# The interplay between sex, time of day, fasting status, and their impact on cardiac mitochondrial structure, function, and dynamics

**DOI:** 10.1038/s41598-023-49018-z

**Published:** 2023-12-07

**Authors:** Mariame S. Kane, Gloria A. Benavides, Edie Osuma, Michelle S. Johnson, Helen E. Collins, Yecheng He, David Westbrook, Silvio H. Litovsky, Kasturi Mitra, John C. Chatham, Victor Darley-Usmar, Martin E. Young, Jianhua Zhang

**Affiliations:** 1 Department of Pathology, University of Alabama at Birmingham, 901 19th Street S., Birmingham, AL BMRII-53435294-0017 USA; 2 Present Address: Birmingham VA Health Care System (BVACS), Birmingham, USA; 3 Baylor College of Medicine, Houston, USA; 4 Present Address: Department of Medicine, University of Louisville, Louisville, USA; 5 Department of Clinical Medicine, Suzhou Vocational Health College, Suzhou, Jiangsu China; 6 Present Address: Department of Genetics, University of Alabama at Birmingham, Birmingham, AL USA; 7 Ashoka University, Sonipat, NCR (Delhi) India; 8 Present Address: Department of Medicine, University of Alabama at Birmingham, 703 19th St. S., ZRB 308, Birmingham, AL 35294 USA

**Keywords:** Biochemistry, Physiology

## Abstract

Mitochondria morphology and function, and their quality control by mitophagy, are essential for heart function. We investigated whether these are influenced by time of the day (TOD), sex, and fed or fasting status, using transmission electron microscopy (EM), mitochondrial electron transport chain (ETC) activity, and mito-QC reporter mice. We observed peak mitochondrial number at ZT8 in the fed state, which was dependent on the intrinsic cardiac circadian clock, as hearts from cardiomyocyte-specific BMAL1 knockout (CBK) mice exhibit different TOD responses. In contrast to mitochondrial number, mitochondrial ETC activities do not fluctuate across TOD, but decrease immediately and significantly in response to fasting. Concurrent with the loss of ETC activities, ETC proteins were decreased with fasting, simultaneous with significant increases of mitophagy, mitochondrial antioxidant protein SOD2, and the fission protein DRP1. Fasting-induced mitophagy was lost in CBK mice, indicating a direct role of BMAL1 in regulating mitophagy. This is the first of its kind report to demonstrate the interactions between sex, fasting, and TOD on cardiac mitochondrial structure, function and mitophagy. These studies provide a foundation for future investigations of mitochondrial functional perturbation in aging and heart diseases.

## Introduction

Mitochondria are a major constituent of the heart and play a key role in ATP synthesis, redox signaling, and homeostasis of calcium and metabolites. Underscoring this central role, deleterious mitochondrial structural and functional changes are associated with aging and various cardiac pathologies^1,2^. Moreover, both sex and nutrition are established modifiers of mitochondrial function, as well as health and disease outcomes^3–5^. Optimal mitochondrial performance is dependent on several integrated pathways that maintain organelle quality, including fission, fusion, and mitophagy^6–9^. Cardiac oxidative metabolism fluctuates dramatically over the course of the day in parallel with contractility and energetic/nutritional demands, all of which are regulated in part by the intrinsic cardiomyocyte circadian clock^10–19^. Whether mitochondrial function or the associated mitochondrial quality control pathways exhibit diurnal variations in the heart dependent on the circadian clock is not known.

Using mouse models in which the cardiomyocyte circadian clock is impaired, we have demonstrated that the heart exhibits time-of-day changes in non-oxidative metabolism, including glycogen and triglyceride synthesis, protein turnover, cellular signaling, electrophysiology, and contractility, which are all regulated by the intrinsic cardiomyocyte clock. This is exemplified by the fluctuation of protein quality control, protein synthesis, and autophagy over the 24 h period (both peaking during the first 4 h of the sleep phase), secondary to the combined effects of daily feeding-fasting cycles and the cardiomyocyte circadian clock^10,19^. Genetic disruption of the cardiomyocyte circadian clock has shown that this molecular mechanism temporally governs a broad range of processes that are essential for cardiac function (including metabolism, signaling, and electrophysiology)^10–17^. Indeed, mouse models in which the cardiomyocyte circadian clock is impaired develop an age-dependent decrease in cardiac function and exhibit shortened lifespans^17^.

In hearts from mice where the cardiomyocyte circadian clock has been disrupted, mitochondrial state 3 respiration is attenuated and a decrease in mitochondrial protein content and complex I and IV activities have been reported^10,19^. Deletion of BMAL1 (Basic Helix-Loop-Helix ARNT Like 1) in human embryonic stem cell-derived cardiomyocytes increased mitochondrial area, decreased mitochondrial respiration (e.g., basal, ATP-linked, maximal and reserve capacity), and abundance of mitophagosomes in vitro^20^. Collectively, these observations suggest that genetic disruption of the cardiomyocyte circadian clock impairs mitochondrial function. However, whether mitochondrial function and quality control fluctuate over the course of the day in the heart in vivo, and the extent to which this is regulated by the cardiomyocyte has not yet been determined.

Mitophagy, a key mechanism involved in mitochondrial quality control, allows for whole or segments of damaged mitochondria to be removed^6,21–23^. Prior studies have shown that deficits in mitophagy result in impaired cardiac function^9,24^. Nutrient deprivation or starvation has been shown to induce mitophagy in several species and specific organs ranging from rodent livers^25^, yeast^26^, worms^27^, and mammalian cell cultures^28^. Whether mitophagy in the heart may also be regulated by TOD and the time course of fasting from a fixed ZT has not been previously studied in the heart.

To address these fundamental questions, mitochondrial structural, functional, and mitophagy parameters were assessed in hearts collected from both male and female mice every 4 h throughout a 24 h period. Studies were also performed in ad libitum fed mice and mice fasted for 24 h. We also used cardiomyocyte-specific BMAL1 knockout (CBK) mice to determine whether potential time of the day (TOD)-dependent changes in mitochondria and mitophagy were regulated by the cardiomyocyte circadian clock. We found that mitochondrial number fluctuates across TOD and is regulated by the intrinsic cardiac circadian clock, while mitochondrial electron transport chain (ETC) activities were not significantly altered across TOD. However, ETC activities were significantly decreased in response to fasting in parallel with increased mitophagy. Furthermore, we observe complex interactions between TOD and sex, between TOD and fasting, between sex and fasting, and among TOD, sex and fasting in mitochondrial structure, function, and quality control. These observations demonstrated the existence of different mechanisms of regulating mitochondrial structure and quality control between sexes, of depleting of mitochondrial inner membrane ETC components versus matrix enzymes in response to fasting. These data will provide a foundation for sex-dependent strategies to modulate TOD-dependent genetic, pharmacological and life-style adjustment for disease interventions.

## Materials and Methods

### Mice

Prior studies have used transmission electron microscopy^8^ as well as reporter fluorescent proteins such as mito-QC and mtKeima^29,30^ to analyze mitophagy in vivo. In this study, we have used the mito-QC reporter mice (C57/BL6 background) which contain mCherry-GFP fused to the amino acid sequence of FIS1 which targets the fusion protein to the mitochondria with both red and green fluorescence, and red only when the mitochondrion is translocated to the lysosomes^29^. This model has been used to assess mitophagy in a diverse range of tissues, including the eye^31^, kidney^32^, brain^33^, heart^34^, and cell cultures^35^. The mito-QC transgene encodes for a mitochondrially-targeted protein that emits both red and green fluorescence in mitochondria, but only red fluorescence in the lysosomal environment, indicating mitophagic flux. Cardiomyocyte-specific BMAL1 knockout (CBK) mice (C57/BL6 background) are used to determine whether the cardiac circadian clock impacts TOD-dependent mitochondrial changes in the heart. Because prior studies focused on male CBK phenotypes^17,36–38^, this study only used male CBK mice to allow comparison with the prior literature. CBK mice that are heterozygous for the mito-QC transgene are used to determine whether the cardiac circadian clock impacts TOD-dependent mitophagy changes.

**Figure 1:**
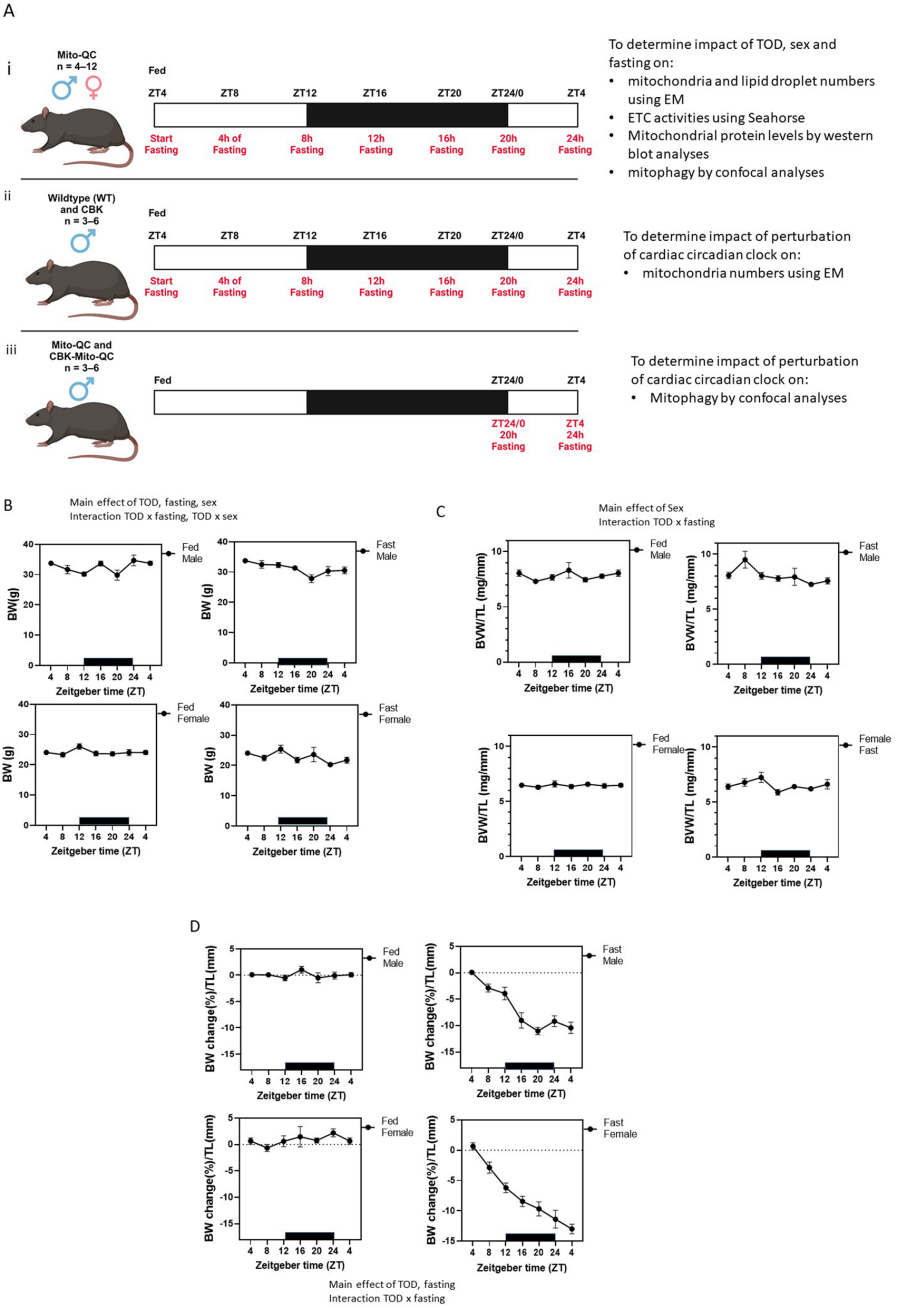
Time-of-the-day (TOD), fasting and sex-dependent changes of body weight and heart weight. (**A**) Diagram of the different groups of mice either fed ad libitum and sacrificed at ZT4, 8, 12, 16, 20, 24 and 28/4 (n = 6 each sex, each ZT for either fed or fasting), or were undergoing fasting starting ZT4 and sacrificed at ZT8, 12, 16, 20, 24 and 28 (with 4–24 h. fasting, respectively, n = 6 or more, each sex). (**A-i**): Determine impact of TOD, sex and fasting on: (1) mitochondria and lipid droplet numbers using transmission electron microscopy (EM); (2) ETC activities using XF extracellular flux analyzer; (3) mitochondrial protein levels determined by western blot analyses; (4) mitophagy quantified by confocal analyses. Male and female mito-QC mice^29^ (heterozygous for the mito-QC transgene) (n = 4–12 at each time of day) were used in all studies. Mice were either fed ad libitum or fasted at ZT4 for up to 24 h. Fed and fasted mice were sacrificed every 4 h starting at ZT4. (**A-ii**) To determine whether cardiac circadian clock is important for mitochondrial morphology, male cardiomyocyte-specific Bmal1 knockout (CBK)^17^ mice and littermate controls (n = 3–6 each group) were used for EM studies for mitochondrial number. Mice were either fed ad libitum or fasted at ZT4 for up to 24 h. Fed and fasted mice were sacrificed every 4 h starting at ZT4. (**A-iii**) To determine whether cardiac circadian clock is important for mitophagy, male CBK:mito-QC (CBK mice that are heterozygous for the mito-QC transgene) and their littermate control mito-QC mice (n = 3–6 each group) were used for mitophagy studies by confocal analyses. Mice were either fed ad libitum or fasted at ZT4 for 20 or 24 h. Fed and fasted mice were sacrificed at ZT0 and ZT4. Made with BioRender. (**B**) The body weight of each animal at the time of sacrifice are plotted. Three-way ANOVA shows the effect of time-of-day (TOD), sex, fasting, TOD × fasting, and TOD × sex. (**C**) Biventricular heart weight of each animal is normalized to tibia length (BHW/TL) and plotted. Three-way ANOVA shows the effect of sex, as well as TOD × fasting interaction. (**D**) The body weight change was calculated with the body weight at sacrifice minus the body weight at ZT4 the day before, normalized to the body weight at ZT4 the day before, as well as to its tibia length (ΔBW%/TL). Three-way ANOVA shows the effect of TOD, fasting, and TOD × fasting. Data = mean ± SEM.

All mice were 19-week-old housed individually in standard, clear, polycarbonate mouse cages with wire bottom floors on a 12 h light–dark cycle (lights on at 6:00 am, Zeitgeber time (ZT) 0) with access to standard rodent chow and water ad libitum, unless stated otherwise. All mice were allowed to acclimatize to these conditions for 1 week prior to experimentation. For the fasting groups, fasting was initiated in a subset of the experimental mice for a duration of up to 24 h; more specifically, food was removed at 10:00 am (ZT4), but water was kept freely accessible. Mice were euthanized at 20 weeks of age with Fatal-Plus (pentobarbital, obtained through the Animal Resource Program at UAB) at specific times of the day (ZT4, ZT8, ZT12, ZT16, ZT20, or ZT0). Hearts were immediately perfused with 1 × PBS (approximately 5 min to remove excess blood), and cut into sections; one section was frozen in liquid nitrogen for biochemical analysis, one section was fixed with glutaraldehyde for electron microscopy, while the third section was fixed with paraformaldehyde for histology (mitophagy assessment). All procedures were approved by the University of Alabama at Birmingham's Institutional Animal Care and Use Committee. All methods were carried out in accordance with relevant guidelines and regulations, and all methods are reported in accordance with ARRIVE guidelines. Experimental designs are described in Fig. 1A legend.

### Transmission electron microscopy (EM)

Cardiac tissues (apex) were fixed with 2% glutaraldehyde in 0.1 M sodium cacodylate buffer (pH 7.4) at 37 °C^8^. Following fixation, samples were placed in 2% osmium tetroxide in 0.1 M sodium cacodylate buffer (pH 7.4), dehydrated in a graded series of ethyl alcohol and embedded in Epon. Ultrathin sections were cut with an ultramicrotome and placed on grids. Following counterstaining, longitudinal sections of cardiac tissues were acquired at the UAB high-resolution imaging facility using an Olympus VS120 robotic microscope on a BX61 platform that was equipped with an automated slide scanner and CellSens (Olympus Corp., Tokyo, Japan) control software. Images were acquired using two magnifications: (i) ×2100 for mitochondria and lipid number counting and (ii) ×4400 for area and length measurements. Mitochondrial and lipid parameters were assessed blindly using NIH ImageJ software. Six mice (5 images for each mouse) from each sex, each ZT for either fasting or fed animals, were analyzed^8^.

### Mitochondrial electron transport chain (ETC) activities

The top one-third of the bi-ventricular section of the heart was pulverized in liquid nitrogen and subsequently homogenized in MAS buffer (70 mM sucrose, 220 mM mannitol, 5 mM KH2PO4, 5 mM MgCl_2_, 1 mM EGTA, and 2 mM HEPES, pH 7.4; 10 μL/mg tissue) using a glass-glass Dounce homogenizer. Homogenates were centrifuged at 1000 × g for 10 min at 4 °C. Supernatant was collected and protein concentrations were determined by DC Protein Assay (Bio-Rad). Heart homogenates (1 µg protein) were loaded onto Seahorse XF96 microplate (Agilent, Santa Clara, CA) in 20 µL of MAS buffer. The loaded plate was centrifuged at 2000 × g for 20 min at 4 °C, followed by the addition of 160 µL of MAS prepared with cytochrome c (10 µM) and alamethicin (10 µg/ml). To measure complex activities, the following substrates were added: for complex I: 1 mM NADH; for complex II: 10 mM succinate with 2 μM rotenone; for complex III: 0.5 mM duroquinol; and for complex IV: 2 mM ascorbate with 0.5 mM TMPD. The following complex inhibitors were used: to inhibit complex I: 2 µM rotenone; to inhibit complex II and III: 10 µM antimycin A (AA); and to inhibit complex IV: 20 mM azide^39,40^.

### Citrate synthase activity assay

Biochemical assays were performed by incubating protein lysates with oxaloacetate, acetyl CoA, and DTNB. The reaction product TNB-CoA was measured at 412 nm, and the activity as nmol/min/mg protein was calculated from the rate of absorbance change, as described^40,41^.

### LDHA activity assay:

S previously described^40,41^, we incubated protein lysates with NADH and Pyruvate. The decrease of NADH absorption at 340 nm was measured and used to calculate the activity as nmol/min/mg protein.

### Western blot analysis

Protein extracts (10 μg) made in MAS buffer (see above Mitochondrial electron transport chain activities) were further diluted in RIPA buffer, then separated on 4–12% Criterion™ XT Bis–Tris Protein Gels (Biorad 3,450,124) and transferred to nitrocellulose membranes according to standard procedures^39^. Every gel was randomized for TOD or fasting status, and every gel for the same antibody contained a common anchor lane from an identical extract for normalization purposes to allow comparisons of protein levels from different gels. Antibodies used: complex I subunit NDUFA9 (dilution: 1:2500, Invitrogen, 459100), complex III subunit UQCRC1 (dilution: 1:2000, Invitrogen, 459140), complex IV subunit MTCO1 (dilution: 1:2500, Abcam, ab14705), complex V ATP5A1 (dilution: 1/1000, Invitrogen 459240), DRP1 (dilution: 1:1000, Abcam, ab56788), FIS1 (dilution: 1:500, Abcam, ab229969), OPA1 (dilution: 1:1000, BD Biosciences, 612606), VDAC (dilution: 1:1000, ab14734), citrate synthase (dilution: 1:1000, Abcam, ab129095), SOD2 (dilution: 1:2500, BD Biosciences, 611580), and SOD1 (dilution: 1:1000, Abcam, ab13499). Anti‐mouse and anti‐rabbit peroxidase‐linked secondary antibodies (GE Healthcare, Life Sciences) were used at 1:5000, and signals were detected using a chemiluminescence system (Super signal^®^ West Dura, Thermo Scientific, #34076; (Super signal^®^ Femto, Thermo Scientific, # 34095) (Thermo Fisher Scientific). Image Quant software was used to analyze the acquired images. Ponceau-S staining was used as a loading control. All original uncropped western blot images are in a Supplemental file, each lane from each mouse.

### Mitophagy assessment

The middle one-third of the bi-ventricular section of the heart was immersed in 4% Paraformaldehyde in 200 mM Hepes buffer, pH 7.2 for 12–16 h at 4 °C. The fixed cardiac tissues were washed in 1 × PBS at 4 °C before density-dependent cryoprotection in filtered 30% sucrose at 4 °C for 24 h. Cryoprotected tissues were embedded in OCT (Thermo Fisher Scientific) and slowly frozen via controlled immersion in chilled 2-Methylbutane (Fisher Chemical) on liquid nitrogen and sectioned on a cryostat (CM1520; Leica Biosystems). The 20-µm sections were mounted on Fisherbrand superfrost plus slides with antifade mountant with DAPI (Invitrogen). Images were acquired with Plan apochromat 40× oil, 1.4 NA objective on Zeiss LSM 700 AxioObserver. All images were acquired with identical settings, including laser intensity, gain, magnification, and exposure time. ZEISS Zen Black was used to convert the 3D images into maximal projection intensity images and ZEISS Zen Blue was used to detect mitophagy events in the cardiac tissues. For image analyses, all images in each experimental group were processed as a batch using identical protocols. Mitophagy was quantitated using applied thresholding and morphological filtering to the brightest mCherry fluorescence signal (areas brighter than the background at low resolution) in order to separate and count the signals. Using the Image Analysis module in Zen-Blue, we were able to count mitophagy events based *on: (i) intensity ranges resulting from set thresholds and (ii) minimum continuous size. The image was smoothed using the “Low Pass method*”, which is useful in cases of noisier edges of objects, resulting in a better representation of the quantified biological event and optimized identification of mitophagy events. In the segmentation step, edge enhancement method was applied to tighten up the edge of the threshold around the object and we excluded tiny objects counts by setting a minimum area in pixels for the area being segmented and included in the measurement. The mitophagy events were identified using the automated *Otsu* method^42^. Object identification was further refined by separating identified objects using the morphology method^42,43^. The features analyzed were the number of mitophagy events and the area for each spot identified. Blinded experimenter manually counted nuclei number and data is presented as total mitophagy area normalized by number of nuclei in image.

### Statistical analyses

Statistical analyses were performed using GraphPad Prism7, with three-way ANOVA (for Fig. 1B–D) and mixed effect analyses (for all other figures) to determine the main effect, followed by *Sidak*’s post hoc test. Two-way ANOVA was performed to determine TOD and fasting effect in each sex, and one-way ANOVA to determine TOD effect. The data shown represent mean ± SEM, *p* < 0.05 were considered significant. Results of the statistics are included in Supplementary Tables 1, 2. Data were reviewed for normality using QQ plot and Shapiro tests prior to statistical analysis. Data points that were more than two-standard deviations from the mean were considered an outlier and excluded from the statistical analyses. The mean of all parameters from each ZT point was standardized to Z-Score values before JMP analysis. Analysis of unsupervised hierarchical clustering heat map and dendrogram was done by JMP using the ward method (which defines the distance between two clusters as the ANOVA sum of squares between the two clusters summed over all the variables) and the unstandardized option (Z-score data), the constellation plot was constructed with the dendrogram clusters (Fig. 9). To observe how different parameters were associated with each other in each individual mouse, we performed a Spearman correlation in R-studio using all raw data and corrected the comparisons with a false discovery rate (FDR) to adjust for the actual p-value distribution and balance type II errors (Fig. 10).

## Results

### Impact of sex, fasting status, and time-of-day on murine gravimetric parameters

To determine whether time-of-day (TOD) influenced mitochondrial parameters in the murine heart, we initially single-housed ad libitum fed 20-week-old heterozygous mito-QC mice and collected hearts at 4 h intervals over a 24 h period, starting at ZT4 (Fig. 1A). Figure 1B, C shows TOD effect on body weight in only males ((Fig. 1B, C, Supplementary Table 1 one-way ANOVA). TOD did not affect biventricular heart weight in both sexes. Three-way ANOVA shows TOD, fasting, sex, TOD × fasting, and TOD × sex effects on body weight (Fig. 1B, Supplementary Table 2). When subjected to fasting, females had lower biventricular heart weight/tibia length and body weight. Fasting effects on biventricular heart weight/tibia length also vary at different TOD (Supplementary Table 2, three-way ANOVA, Fig. 1C). To determine the effect of fasting on the percent change in body weight, the body weight of each animal was measured the day before fasting at ZT4, and then again immediately before sacrifice at the indicated ZTs (Fig. 1D). There is a clear TOD and fasting effect, with higher body weight during the active phase and the expected decrease in body weight after fasting. Altogether, these findings support distinct sex differences in the TOD regulation of gravimetric parameters and in responses to nutrition status.

### Regulation of mitochondrial numbers in the heart

**Figure 2:**
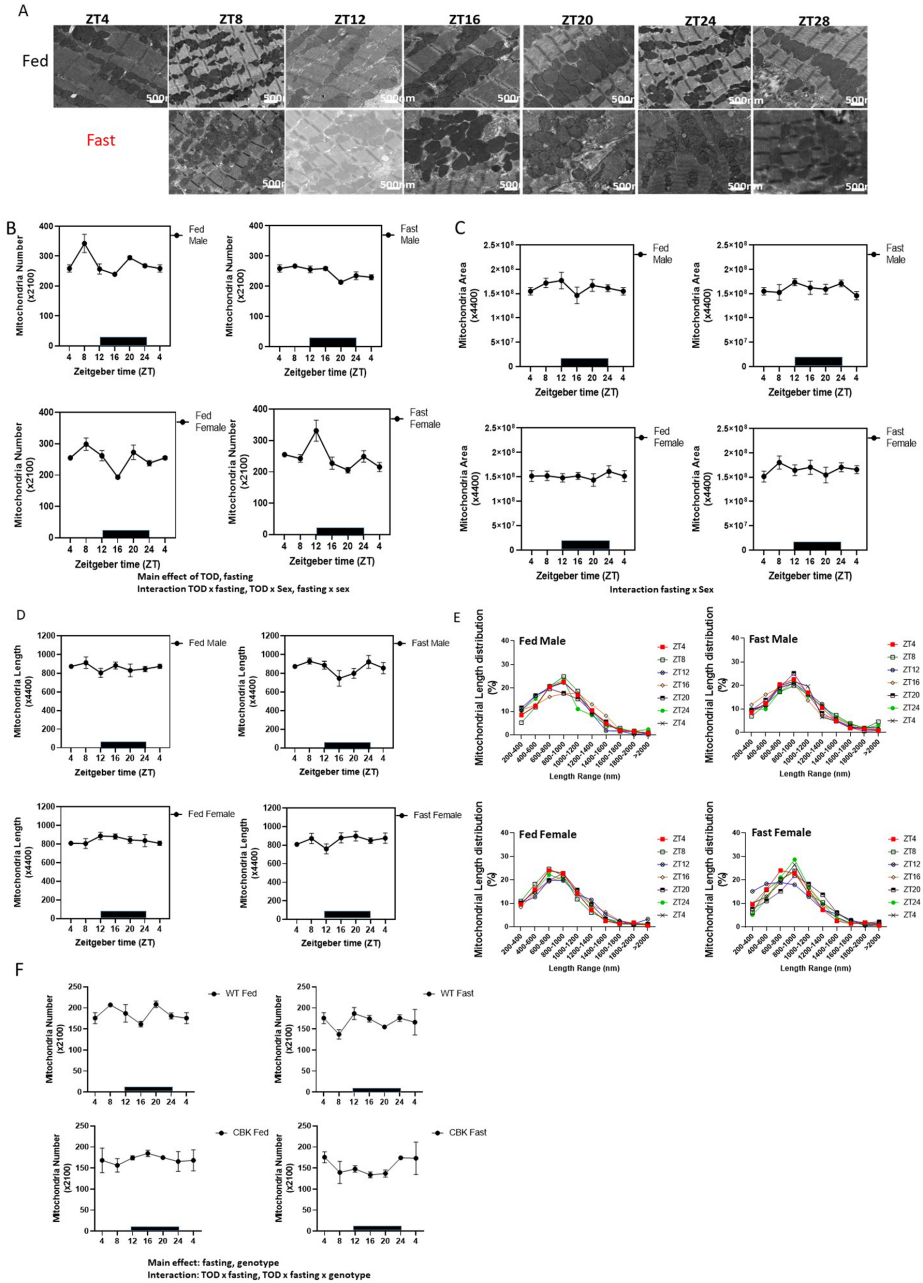
Mitochondrial number is regulated by both time-of-day (TOD) and fasting. Transmission electron microscopy images taken from cardiac tissue isolated from fed or fasted 20-week-old mito-QC male and female mice. (**A**) Representative images for the male mice at the indicated ZT. Scale bar = 500 nm. (**B**) Mitochondrial number was calculated from an average of 5 images for each mouse (×2100 magnification). Three-way ANOVA analyses indicate that there are main effects of TOD and fasting. In addition, there are effects of TOD × fasting, and TDO × sex interactions. (**C**) The relative mitochondrial area was calculated from a cumulative of the mitochondrial sectional area from 5 images (×4400 magnification) for each mouse using image J. Three-way ANOVA mixed effect analyses identified no significance. (**D**) Mitochondrial length was computed as the major axis of mitochondria from an average of 5 images for each mouse (×4400 magnification) using image J. Three-way ANOVA mixed effect analyses identified no significance among comparisons. (**E**) The percent distribution of the numbers of mitochondria between 200–400 nm, 400–600, 600–800, 800–1000, 1000–1200, 1200–1400, 1400–1600, 1600–1800, 1800–2000, and > 2000 nm were plotted for each ZT, each sex, at fed or fasting conditions. (**F**) Comparing mitochondrial number in males of CBK mice with littermate control mice. We found that there is a significant effect of fasting and genotype in 3-way ANOVA, and effects of TOD × fasting, and TOD × fasting × genotype. (**B**–**E**) n = 4–6 mice for each experimental group. (**F**) n = 3–4 mice. Data = mean ± SEM, 5 images each mouse.

Mitochondria are highly dynamic organelles capable of modulating their structure by fusion or fission events. This structural plasticity is inherent to mitochondrial health and function. We used transmission electron microscopy (EM) to determine whether mitochondrial number or morphology oscillates across TOD (Fig. 2A). We found that mitochondrial numbers showed TOD effects (Supplementary Table 1, one-way ANOVA, in male/female fed mice), along with a significant impact of fasting and an interaction of TOD × fasting and TOD × sex (Fig. 2B). Post-hoc one-way ANOVA analyses found that male fed mice exhibited higher mitochondrial number at ZT8 when mice are inactive, and female fed mice exhibit lower mitochondrial number at ZT16 when the mice are active. Fasting for 16 h decreased mitochondrial number in males, while fasting for 8 h increased mitochondrial number in females (Supplementary Table 1). In contrast, while mitochondrial numbers change with TOD and fasting, the mitochondrial cross-sectional area and the average mitochondrial length were not significantly different across TOD, between sexes, or between fasting and fed (Fig. 2C, D). There was an interaction of fasting × sex for mitochondrial area, with females having higher mitochondrial area at ZT8 and ZT4 after 4 and 24 h of fasting (Fig. 2C). In addition, we plotted the distribution of mitochondrial diameter at different ZTs (Fig. 2E). Interestingly, looking at the distribution of different mitochondrial size populations in male fed mice, at ZT8, when mitochondrial numbers were highest, there were fewer mitochondria in the 200–400 nm range. Notably, in female fasting mice, mitochondrial numbers were highest at ZT12 (fasting for 8 h), with the highest percentage of mitochondria in the 200–400 nm range, consistent with more mitochondrial fragmentation. Collectively, these data indicate complex TOD, fasting influence on mitochondrial dynamics with significant interaction with these parameters in different sexes.

Since mitochondrial number varied with TOD with a peak at ZT8, we examined whether mitochondrial number is under the cardiomyocyte circadian control, using the cardiomyocyte-specific BMAL1-knockout mouse (CBK).^17,36–38^ (Fig. 2F). We found a significant main effect of fasting and genotype along with a significant interaction of TOD × fasting and TOD × fasting × genotype. One-way ANOVA found TOD differences in WT-fed and WT-fasting mice, while such TOD differences were lost in CBK mice. Two-way ANOVA in fed mice shows a genotype and TOD × genotype difference. The peak of mitochondrial number is phase-shifted in CBK mice, indicating a circadian regulation of mitochondrial number.

**Figure 3:**
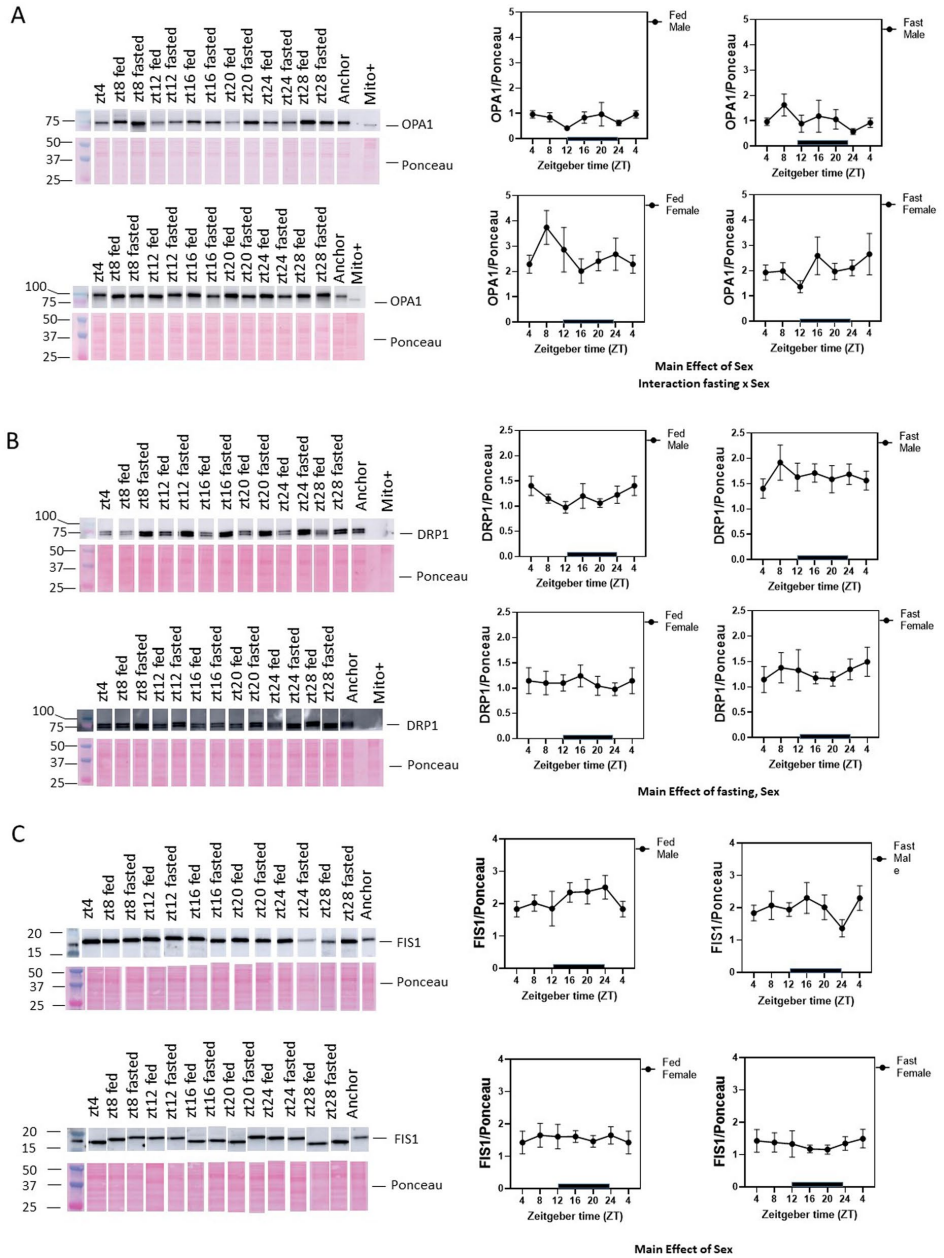
Fission and fusion proteins exhibit sex differences and are regulated by fasting. Western blot analyses were performed to measure the levels of (**A**) OPA1, (**B**) DRP1, and (**C**) FIS1 proteins. As shown OPA1, DRP1, and FIS1 have the main effect of sex. In addition, OPA1 has significance in fasting × sex, and DRP1 has the main effect of fasting. n = 4–6 each group. Data = mean ± SEM.

To further understand the mechanisms by which mitochondrial numbers were changed by TOD and fasting, we measured the levels of fission and fusion proteins. We found that OPA1, DRP1, and FIS1 all exhibited sex-dependent differences with lower fusion protein OPA1 in males (both fed and fasted) and higher fission proteins DRP1 (during fasting) and FIS1 (both fed and fasted) in males (Fig. 3A–C). OPA1 also exhibited a fasting × sex difference with higher OPA1 in females after fasting (Fig. 3A). DRP1 exhibited a fasting effect, with an increase in males after 4 h fasting (Fig. 3B, Supplementary Table 1). These data indicate that different regulatory mechanisms exist in males and females to maintain mitochondrial morphology and size.

### Regulation of lipid droplet numbers in the heart

It has been shown that mitochondria can be associated with lipid droplets, and this association plays a role in regulating electron transport chain activities^44^. In addition, the association between mitochondria and lipid droplets can also affect lipid utilization^44^, providing bioenergetic resources to the heart. It has been shown that increased palmitate can increase mitochondrial fission, and transgenic mice overexpressing ACSL1 (long-chain acyl-CoA synthase 1) in cardiomyocytes exhibit decreased mitochondrial diameter^45^.

**Figure 4:**
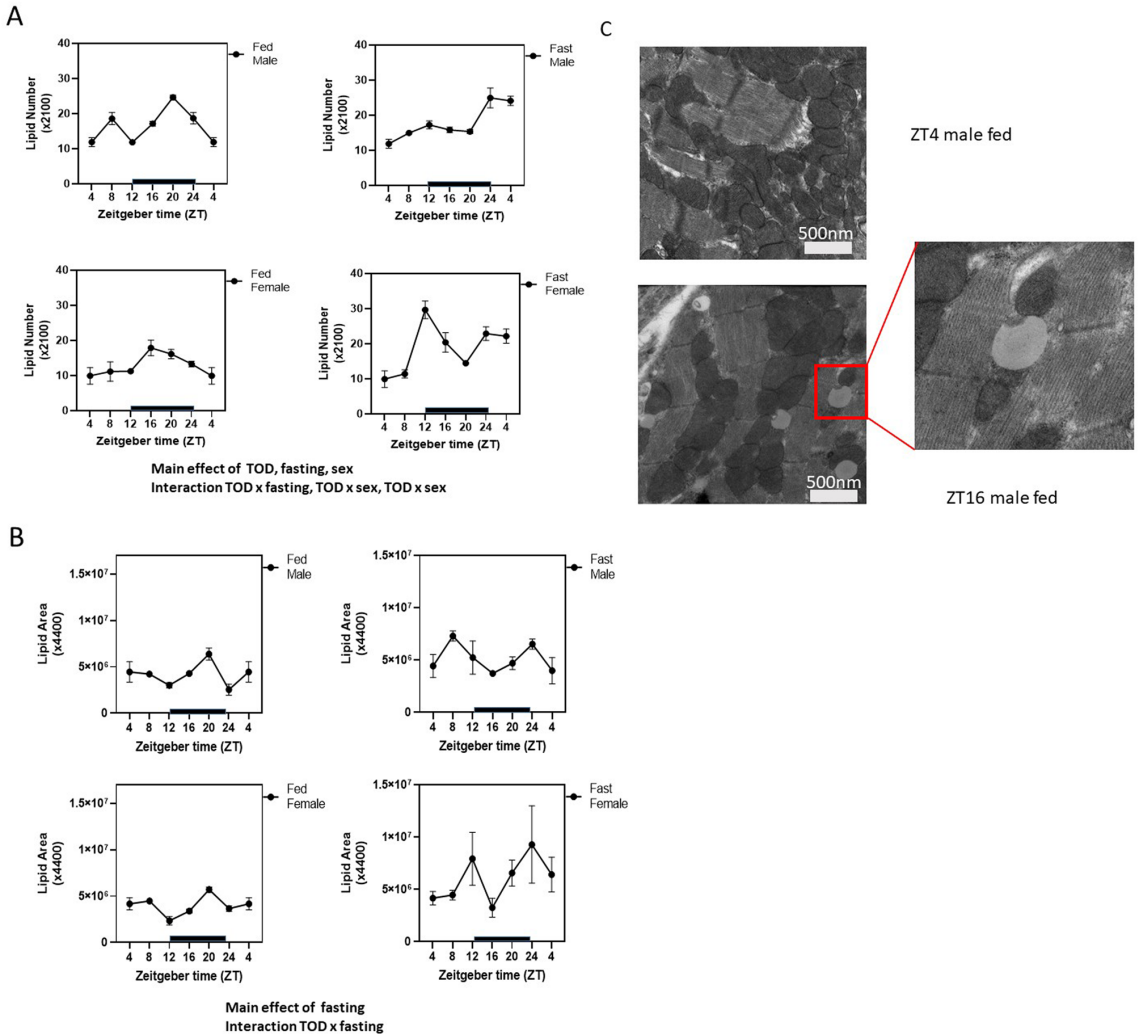
Time-of-day (TOD) and fasting regulation of lipid droplet area and number. Transmission electron microscopy images taken from cardiac tissue isolated from fed or fasted 20-week-old mito-QC male and female mice. (**A**) Lipid number was analyzed from an average of 5 images for each mouse (×2100 magnification). Three-way ANOVA analyses indicate that effect of TOD and fasting; as well as the interaction TOD × fasting, and TOD × sex. (**B**) Relative lipid area was calculated blindly from a total of 5 images for each mouse (×4400 magnification) using Image J. Three-way ANOVA analyses indicate that there is an effect of fasting, and an effect of TOD × fasting interaction. n = 4–6 mice for each experimental group. Data = mean ± SEM. (**C**) Representative images from ZT4 male fed and ZT16 male fed.

To examine whether lipid droplet numbers were changed in parallel to the changes in mitochondrial numbers, we measured lipid droplet numbers and areas using EM (Fig. 4). We found a significant TOD effect on lipid droplet numbers in both male and female fed hearts and *posthoc* analyses identified more specific ZT time-dependent changes in the male heart, including higher lipid numbers at ZT20 in males and ZT16 in females (Supplementary Table 1).

Three-way ANOVA analyses indicate that both the numbers of lipid droplets (Fig. 4A) and the area of each lipid droplet (Fig. 4B) are significantly increased by fasting (Supplementary Table 2). Furthermore, lipid droplet numbers were regulated by TOD, TOD × fasting interaction, and TOD × sex interaction (Fig. 6A, Supplementary Tables 1, 2). In fed males, lipid droplet numbers at ZT8, ZT20, and ZT0 were higher than ZT4 (one-way ANOVA) (an example of a lipid droplet is shown in Fig. 4C). In response to fasting, males exhibited increased lipid droplet numbers at ZT0/fasting for 20 h, and ZT4/fasting for 24 h, compared to ZT4/fasting for 0 h. (one-way ANOVA). In response to fasting, females exhibited increased lipid droplet numbers at ZT12/fasting for 8 h, ZT16/fasting for 12 h, ZT0/fasting for 20 h, and ZT4/fasting for 24 h (one-way ANOVA). Our data suggest that lipid regulation is differentially regulated between males and females, which is consistent with previous reports of gender-related differences in substrate utilization and metabolism, especially in response to fasting.

For lipid droplet area, there is no sex-dependent effect with both male and female fed mice peaking at ZT20. However, there is an effect of TOD × fasting. In particular, male mice exhibited a higher lipid area at ZT8 (4 h after fasting), while females exhibited a higher lipid area at ZT12 (8 h after fasting).

### Sex-dependent increases of citrate synthase (CS) and decreases  of LDH activities in the heart in response to fasting

**Figure 5:**
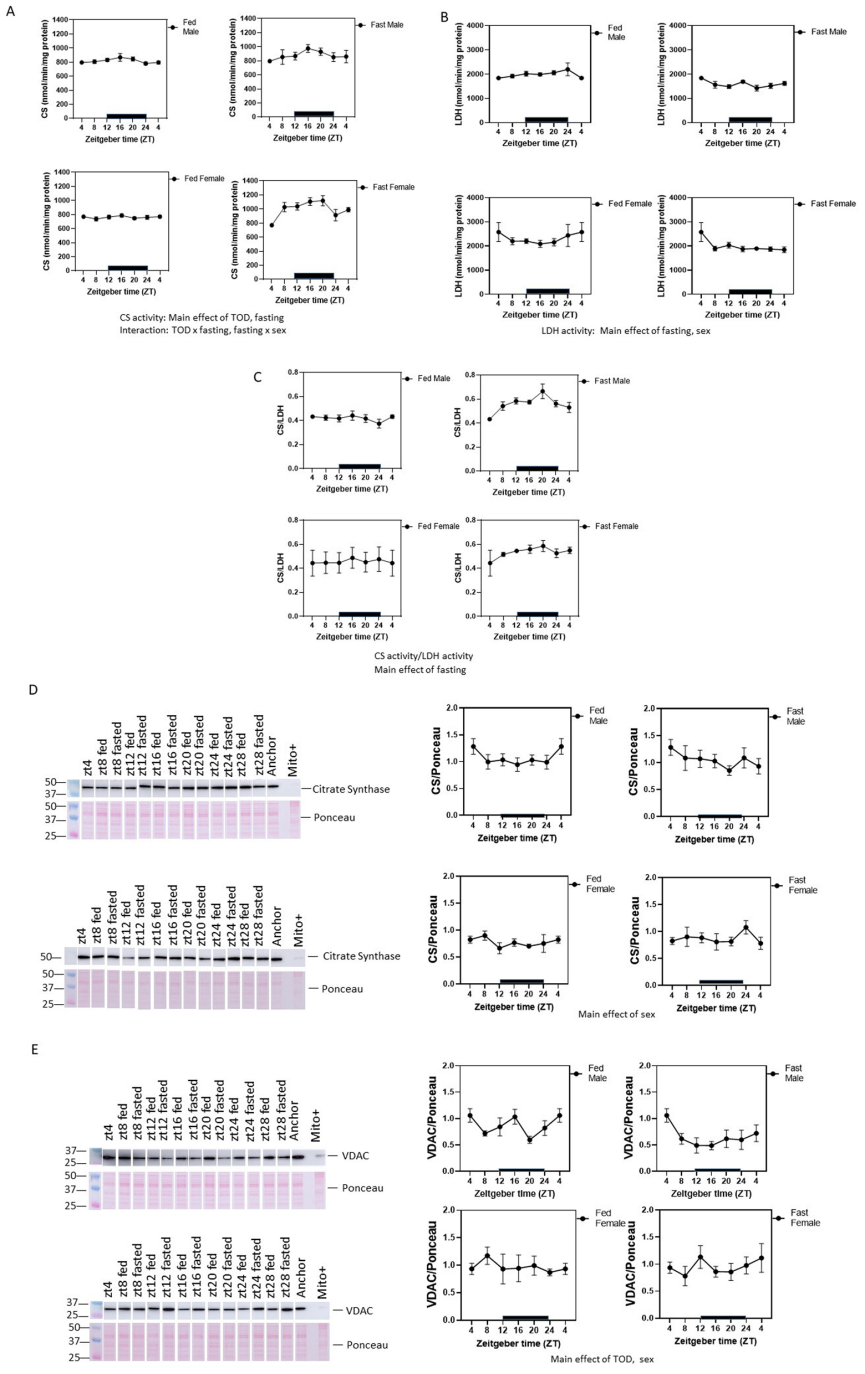
Citrate synthase (CS) activities are regulated by time-of-the-day (TOD) and are increased in response to fasting while LDH activities are decreased in response to fasting. (**A**) CS activities were measured, normalized the protein, and plotted according to the ZT of their harvest. Three-way ANOVA indicates the main effects of TOD and fasting; as well as interaction of TOD × fasting, and fasting × sex. LDH activities were measured across ZT in both males and females, and both the fed ad libitum and the fasting groups (**B**), and citrate synthase (CS) activity: LDH activity ratio were plotted for these groups (**C**). Western blots were also performed to measure the levels of (**C**) CS (mitochondrial matrix protein), and (**D**) VDAC (mitochondrial outer membrane protein). CS protein level shows a significant difference between sexes (three-way ANOVA) but no effects of fasting. VDAC protein levels are significantly changed by sex. Data = mean ± SEM, n = 4–6 per group.

Mitochondrial matrix enzyme citrate synthase (CS) activities and cytosolic lactate dehydrogenase (LDH) activities, were assessed in hearts isolated at the different ZTs (Fig. 5A, B, Supplementary Table 1). Significant TOD-dependent fluctuations of CS or LDH activities were not detected (one-way ANOVA) in either male or female fed mice. However, when taking TOD, sex, and fasting all into consideration, three-way ANOVA analyses of CS activity found an effect of TOD, fasting, and interaction of TOD × fasting, fasting × sex (Fig. 5A). CS activities are higher in the active phase in both fed and fasting animals in both sexes. Fasting elevated CS activity and was more evident in females compared to males. In contrast, LDH activity was decreased by fasting, and LDH activity in males was lower than females in response to fasting (Fig. 5B). Overall, in response to fasting, the ratio of CS/LDH activity was increased after fasting (Fig. 5C). Although CS activities do not exhibit a sex effect, CS protein levels are higher in males (Fig. 5D). The outer membrane protein VDAC level had a sex and a TOD effect, with higher levels in males than females. Furthermore, only in fed males was VDAC lower in ZT20 (Fig. 5E).

### Regulation of mitochondrial electron transport chain (ETC) activities in the heart

**Figure 6:**
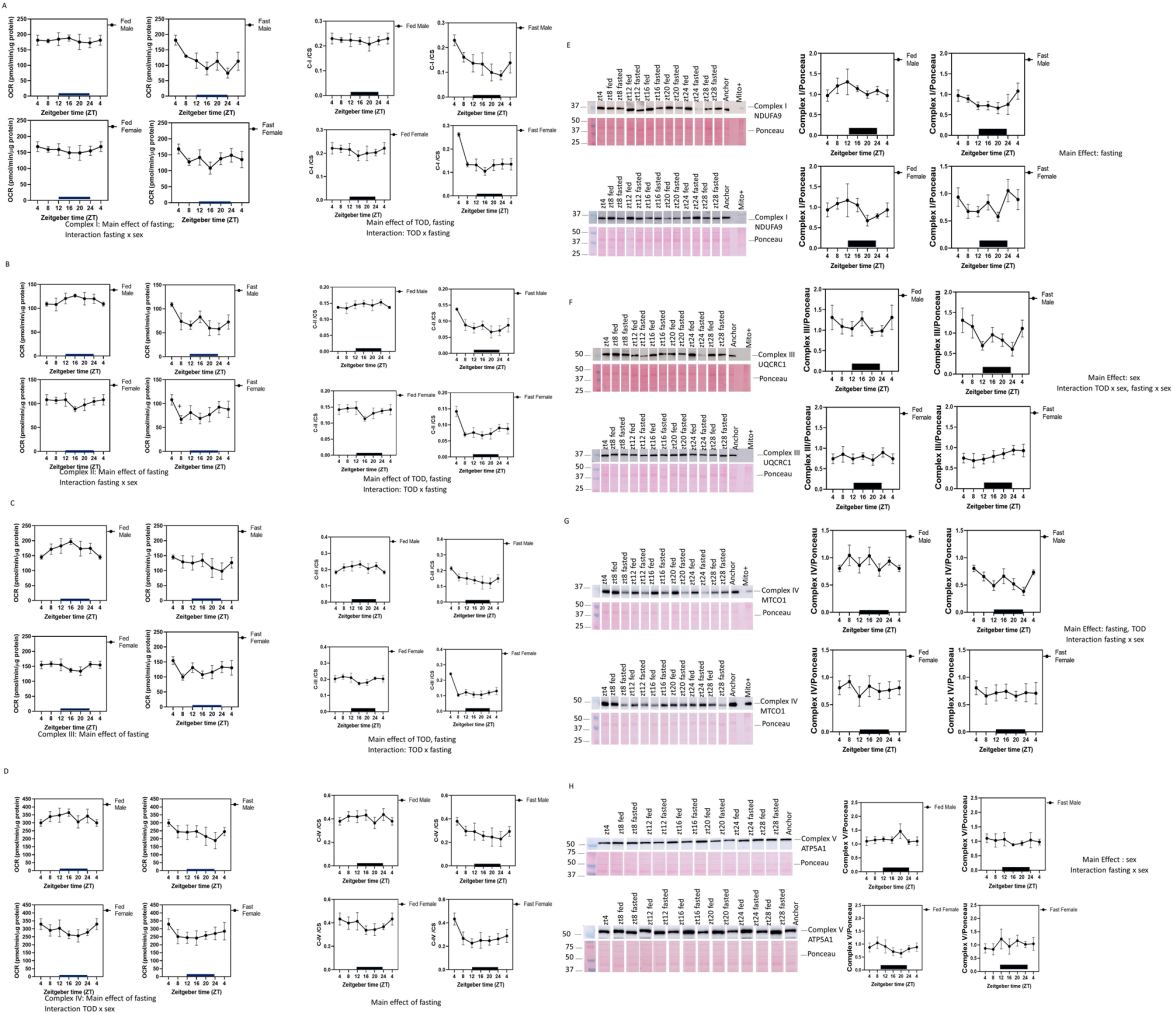
Mitochondrial electron transport chain (ETC) activities and protein levels are significantly decreased in response to fasting. Complex I (**A**), II (**B**), III (**C**) and IV (**D**) substrate-linked oxygen consumption rates (OCR) were measured, normalized to the protein, and plotted according to the ZT of their harvest. Three-way ANOVA found that fasting significantly changed all complex activities. In addition, complex I (**A**) and complex II (**B**) have significant effect of fasting × sex interaction. Complex IV has a significant effect of time-of-day (TOD) × sex (**D**). TOD effects were observed for complex I/CS, complex II/CS, and complex III/CS. Sex effects were observed for complex III/CS. Western blot analyses were performed to measure the levels of (**E**) complex I subunit NDUFA9, (**F**) complex III subunit UQCRC1, (**G**) complex IV subunit MTCO1 and (**H**) complex V subunit ATP5A1. Three-way ANOVA indicates that for complex I there is a main effect of fasting; for complex III there is a main effect of sex, and effect of TOD × sex, and fasting × sex; for complex IV there is a main effect of fasting and TOD, and interaction of fasting × sex; for complex V there is a main effect of sex and interaction of fasting × sex. Data = mean ± SEM, n = 4–6 per group.

To determine whether mitochondrial ETCs in the heart are regulated in a similar manner with mitochondrial number or CS activity, we measured complex I, II, III, and IV substrate-linked oxygen consumption rate (OCR) in hearts isolated at the different ZTs (Fig. 6A–D). ETC activities (normalized to protein) exhibited no significant TOD-dependent fluctuation in hearts from either male or female mice (Fig. 6A–D, Supplementary Table 1). Taking TOD, sex, and fasting all into consideration, there were significant decreases in complexes I-IV substrate-linked OCR in response to fasting. Furthermore, there were significant differences in complex I and II activities with fasting × sex with males exhibiting a more pronounced decrease. There were also significant differences in complex IV activities with TOD × sex, with higher complex IV activities in males than females in the dark phase.

Since CS activities were increased by fasting and CI-IV activities were decreased by fasting, not surprisingly, fasting-induced decreases of ETC CI-IV activities are more pronounced after CI-IV activities are normalized to CS activities. Complex I-III activities/CS activities all have a TOD effect. There were also TOD × fasting effects on complex I-III activities/CS activities (Fig. 6A–D).

To examine whether the regulation of mitochondrial ETC activities was associated with their protein levels or changes in the mitochondrial mass, we performed western blot analyses (Fig. 6E–H). Immunoblot analyses revealed that complex I (NDUFA9) and IV (MTCO1) subunit protein levels were decreased by fasting. There is a main effect of sex on complex III (UQCRC1) levels with fed males having higher levels of UQCRC1, and a main effect of TOD on complex IV (MTCO1) levels. Fasting × sex interaction was significant for protein levels of complex III and IV subunits, with males exhibiting a higher fold decrease compared to females. Sex × TOD was significant for complex III subunit UQCRC1 protein levels. There is a sex effect for complex V ATP5A1 with higher levels in males compared to females, and a sex × fast effect indicating the males and females respond to fasting differently with males trending down and female trending up.

### Regulation of mitophagy in the heart

**Figure 7:**
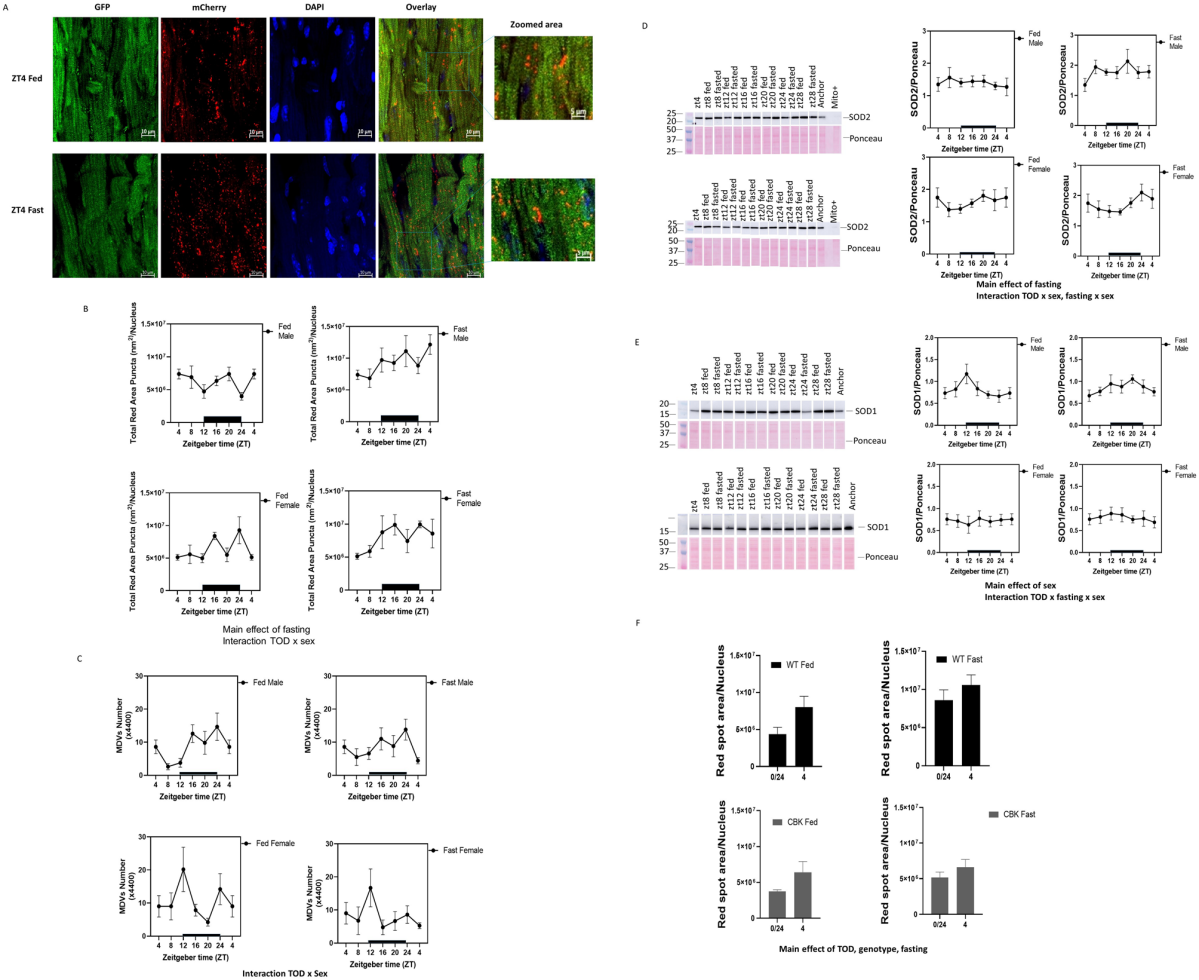
Mitophagy is increased by fasting in the heart. Using confocal analyses, mitophagy is calculated as the total red spot area normalized by the number of nuclei in the mito-QC hearts. Analyses were obtained blindly from 5 images from each mouse using Zen Black (for maximal projection intensity) and Zen Blue (for spot detection from the maximal projection images). n = 4–6 mice per experimental group. (**A**) Representative images of one ZT4 fed male mouse, and one male mouse after fasting for 24 h. and harvested at ZT4. (**B**) Graphs summarize the data analyses. There are main effects of fasting, and sex × TOD by three-way ANOVA analyses. One way ANOVA also revealed TOD differences in fed male and female. (**C**) Transmission electron microscopy images taken from cardiac tissue isolated from fed or fast 20-week-old mito-QC male and female mice as in Fig. 2 enabled us to analyze the mitochondrial-derived vesicles (MDVs, those are 50–200 nm and attached to mitochondria) across TOD, for each sex, at fed or fasting conditions. There is an effect of TOD × sex. Western blot analyses were performed to measure the levels of (**D**) SOD2, and (**E**) SOD1. SOD2 protein levels are significantly changed by fasting, and had significance for sex × TOD, and fasting × sex. SOD1 levels are significantly different between sexes, and is significance for fasting × sex × TOD. (**F**) Mitophagy was measured at ZT0 and ZT4 for male CBK mice either fed ad libitum or fasted for 20 h (ZT0) and 24 h (ZT4). There are effects of TOD, sex and fast by three-way ANOVA.

To determine whether mitochondrial function is coordinately regulated by mitophagy in the heart, we performed confocal microscopy analyses with the mito-QC mice (Fig. 7A, B). Using red-only spot areas normalized to the number of nuclei as an indicator of mitophagy, we found that fasting increased mitophagy (Fig. 7B), consistent with decreased mitochondrial ETC activities (Fig. 6). There was also a sex × TOD effect by three-way ANOVA. One-way ANOVA indicated a TOD effect in both female and male fed mice in ZT4 versus ZT0, with mitophagy higher at ZT4 than ZT0 in males and lower at ZT4 than ZT0 in females (Supplementary Table 1). Mitophagy showed a bimodal distribution in the ad libitum fed male mice, with troughs at ZT12 and ZT0 at the intersections between active and inactive phases, consistent with a boost of conservation of mitochondria. Ad libitum fed females exhibit lower mitophagy compared to males; while lower at ZT12 compared to their male counterparts, females display higher mitophagy at ZT24/0 when the dark phase turns into the light phase, consistent with an immediate start of repair (Fig. 7A, B). Overall, our data shows that TOD regulates basal mitophagy (fed state) with distinct responses in males and females. Fasting abrogated the TOD regulation of mitophagy at ZT12, and increased mitophagy with longer fasting times. While males peak at ZT4/fasting 24 h, females peak at ZT24 (Fig. 7B). Irrespective of TOD, fold increase of mitophagy levels in response to fasting is smaller compared to males.

To determine whether the level of mito-QC protein is altered by TOD, sex or fasting, we measured dim red signals (as representing total mito-QC rather than those in the lysosome). There was no sex difference at ZT4 (Supplementary Fig. 1). However, there was a sex effect but no TOD or fasting effect, if analyzed across all TOD. Mitochondrial-derived vesicles (MDVs) are small vesicles that bud from mitochondria to mediate mitophagy independent of autophagosomes (with a size of 50–200 nm) that can be visualized using EM^46^. Analyses of MDV showed that there was a TOD × sex effect (three-way ANOVA) (Fig. 7C), with higher numbers of MDVs in males in the dark phase and lower numbers of MDVs in females in the dark phase.

Fasting-induced mitophagy is associated with decreased ETC activities and mitochondrial ETC complex I and IV subunit proteins (Fig. 6), and increased DRP1 protein (Fig. 3), without changing outer member VDAC protein or matrix CS protein levels (Fig. 5). The increase in DRP1 is consistent with prior studies indicating that DRP1 may be a key mitophagy regulator^25,47^. To further examine mechanisms of mitophagy regulation, we assessed whether fasting increased mitophagy in the heart is associated with changes in antioxidant protein levels. We found that mitochondrial matrix superoxide dismutase SOD2 protein levels were changed by fasting, TOD × sex, and fasting × sex, consistent with the observation that nutrient deprivation significantly elevates reactive oxygen species (ROS) production, which triggers the elevation of antioxidants^48,49^ (Fig. 7D). The fasting-induced SOD2 increase is more significant and immediate in males compared to females. Cytosolic superoxide dismutase (SOD1) level was also higher in males, and there is a significant fasting × sex × TOD interaction, with males exhibiting a more significant increase in the active phase (Fig. 7E).

To determine whether the cardiac circadian clock regulates fasting-induced mitophagy, we further bred the mito-QC mice with CBK mice (cardiac-specific Bmal1 disruption). We analyzed mitophagy at ZT0 and ZT4 and fasting for 20 and 24 h (harvested at ZT0 and ZT4) for the males. We found that mitophagy is regulated by TOD, fasting, and BMAL1 (Fig. 7F). Both WT and CBK mice had higher mitophagy at ZT4 when fed compared to ZT0, and while fasting increased mitophagy in WT mice, it no longer induced mitophagy in CBK mice.

### Coordinately regulated parameters.

**Figure 8:**
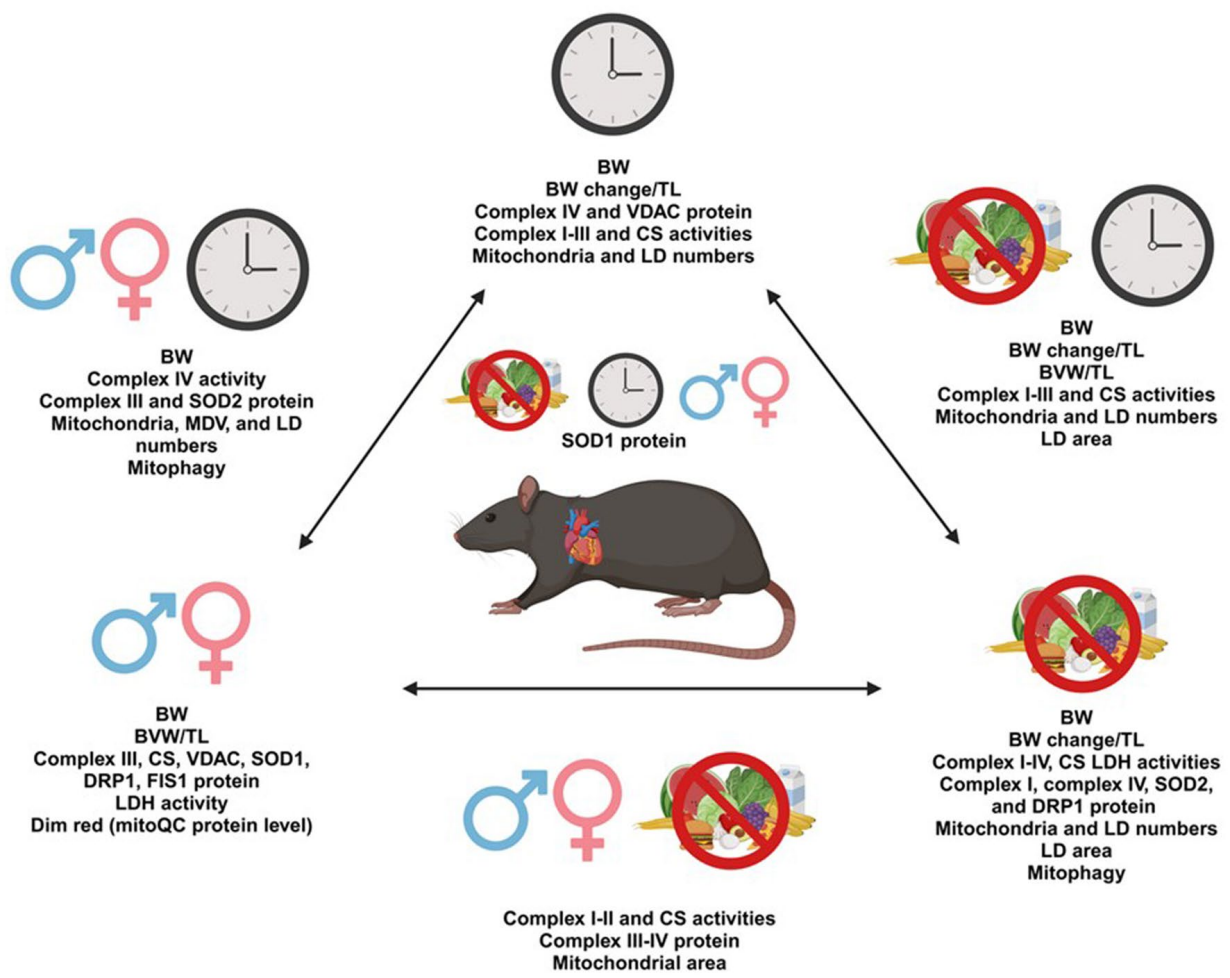
Impact of Time-of-day (TOD), sex, fasting and their interactions on cardiac mitochondrial function and mitophagy. Diagram summarizing the relative impact on cardiac mitochondrial morphology, function and protein levels, and mitophagy by TOD (depicted by a clock), sex (depicted by ♂♀), fasting (depicted by blockade of food) and by the complicated interactions TOD × sex, TOD × fasting, Sex × fasting, and TOD × sex × fasting. Listed are all significant parameters by 3-way ANOVA. Figure generated with BioRender.

As summarized in Supplementary Tables 1, 2 and Fig. 8, we identified the impact of TOD, sex and fasting on mitochondrial homeostasis, morphology, function, and quality control. These are based on 1-way ANOVA across TOD and 3-way ANOVA across TOD × sex × fasting.

**Figure 9:**
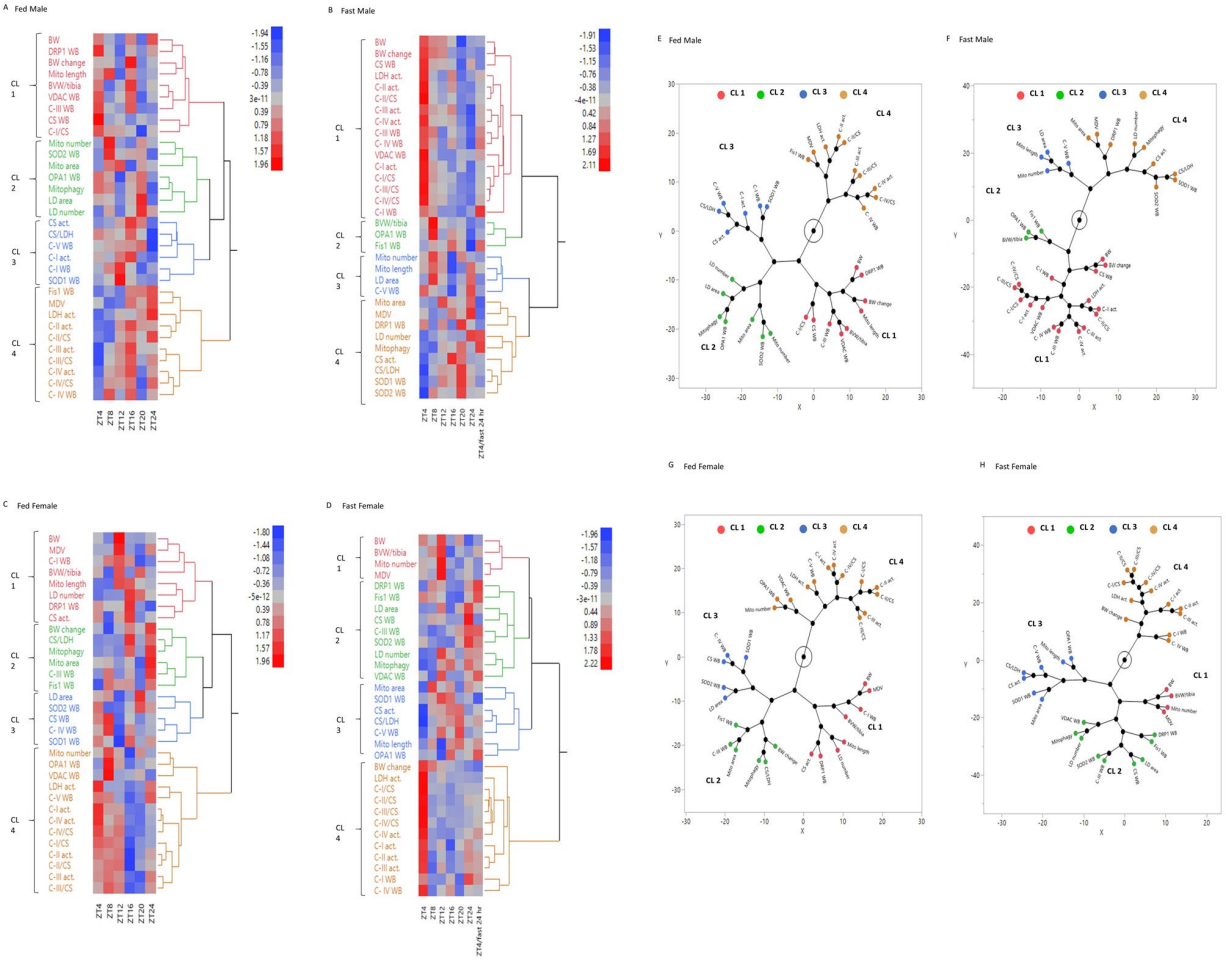
Heat map data visualization shows difference by sex and fasting over TOD. (**A**–**D**) A hierarchical clustered heat map showing the time dependent (ZT) changes of the 32 parameters measured (Z-score) in 4 distinct clusters (CL1 to CL4) for the (A) fed male (B) fast male (C) fed female and (D) fast female. (**E**–**H**) Constellation plot of the cluster (CL1 to CL4) parameters from the dendrogram in the (A) fed male (B) fast male (C) fed female and (D) fast female.

To analyze in greater depth the TOD-dependent interactions between the bioenergetic parameters, heat maps and constellation plots were constructed for male/female with and without fasting (Fig. 9). To reveal the changes of each parameter over the 24-h period the data in each row were converted to a Z score which represents variation from the mean (ZT4–24) for each time point. This was used to generate the heat maps shown in Fig. 9A–D for ZT4–ZT24 with hierarchical clustering of the different parameters regardless of whether the changes reached statistical significance. In the fed male, cluster 1 which includes higher BW, DRP1, VDAC, CIII, CS and CI/CS activity at ZT4. Cluster 2 and 3 parameters, which include mitophagy, lipid droplet area, complex I activity, CS parameters, and complex V protein, are the lowest at ZT24. Cluster 4 which includes mitochondrial parameters related to complex II, III and IV activities are the lowest at ZT4 and an increase to peaks at ZT16 or ZT24. Notably, the lowest mitophagy at ZT24 coincides with the highest complex II, III, and IV activities, and the highest mitophagy at ZT4 coincides with the lowest mitochondrial complex II, III, and IV activities.

In contrast to the fed male, the fed female cluster 1 has peaks at ZT12 or ZT16, while cluster 2 has the highest Z score at ZT24, and cluster 3 has the lowest at ZT12. The highest Z score for mitochondrial complex I-IV activities in cluster 4 are now at ZT4 and lowest at ZT16 in contrast to the males. As found with the fed male low mitophagy Z score occurs at the ZT when mitochondrial activity Z scores are high.

As expected with fasting the majority of the mitochondrial parameters and BW (cluster 1 in male, cluster 4 in female) show a progressive decrease in Z score from ZT4 (fasting for 0 h) to ZT4/fasting 24 h. With increasing fasting time, mitophagy progressively increases to the highest score at ZT4/fasting for 24 h for the male, and at ZT24/fasting for 20 h for the female. In the males, OPA1 is decreased while both DRP1 and SOD2 are increased with fasting with their maximum changes occurring earlier than the mitophagy peak. In the females, although mitophagy and SOD2 peak at ZT24/fasting for 20 h, DRP1 and Fis1 peak at ZT4/fasting 24 h.

The constellation plots (Fig. 9E–H) are an alternative representation of the hierarchical clustering allowing relationships to become more evident. In both the fed Male and female groups there are essentially four clusters but with slightly different elements in each group (Fig. 9E, G). In males, the mitophagy cluster 2 is associated with LD number and area, OPA1 and SOD2 levels and mitochondrial area and number. Cluster 3 is linked to the mitophagy cluster and includes the levels of mitochondrial proteins and mitochondrial CS and complex I activity. In females, LD number and area are no longer in cluster 2 but LD area is distantly related to mitophagy in cluster 3, while Fis1 protein levels are in cluster 2 with mitophagy. In fed males cluster 4 contains a majority of mitochondrial electron transport activities and Fis 1 protein levels and cluster 1 contains both mitochondrial protein levels and activities and body weight related parameters. Clusters 1 and 2 have a similar distribution in the fed female to male animals with more members in cluster 4 for females. Fasting introduces an extensive reorganization of the clusters for both males and females. The two main clusters in both the male and female groups now show a strong association between the majority of the mitochondrial protein levels and activities in cluster 1 and mitophagy cluster 4 in males, and in cluster 4 and mitophagy cluster 2 in females. Clusters with OPA1 level, mitochondrial number and length clusters are dissociated from mitochondrial activity and mitophagy clusters. Overall these analyses show that mitochondrial structure, function and mitophagy are differentially regulated by TOD, sex and fasting.

**Figure 10:**
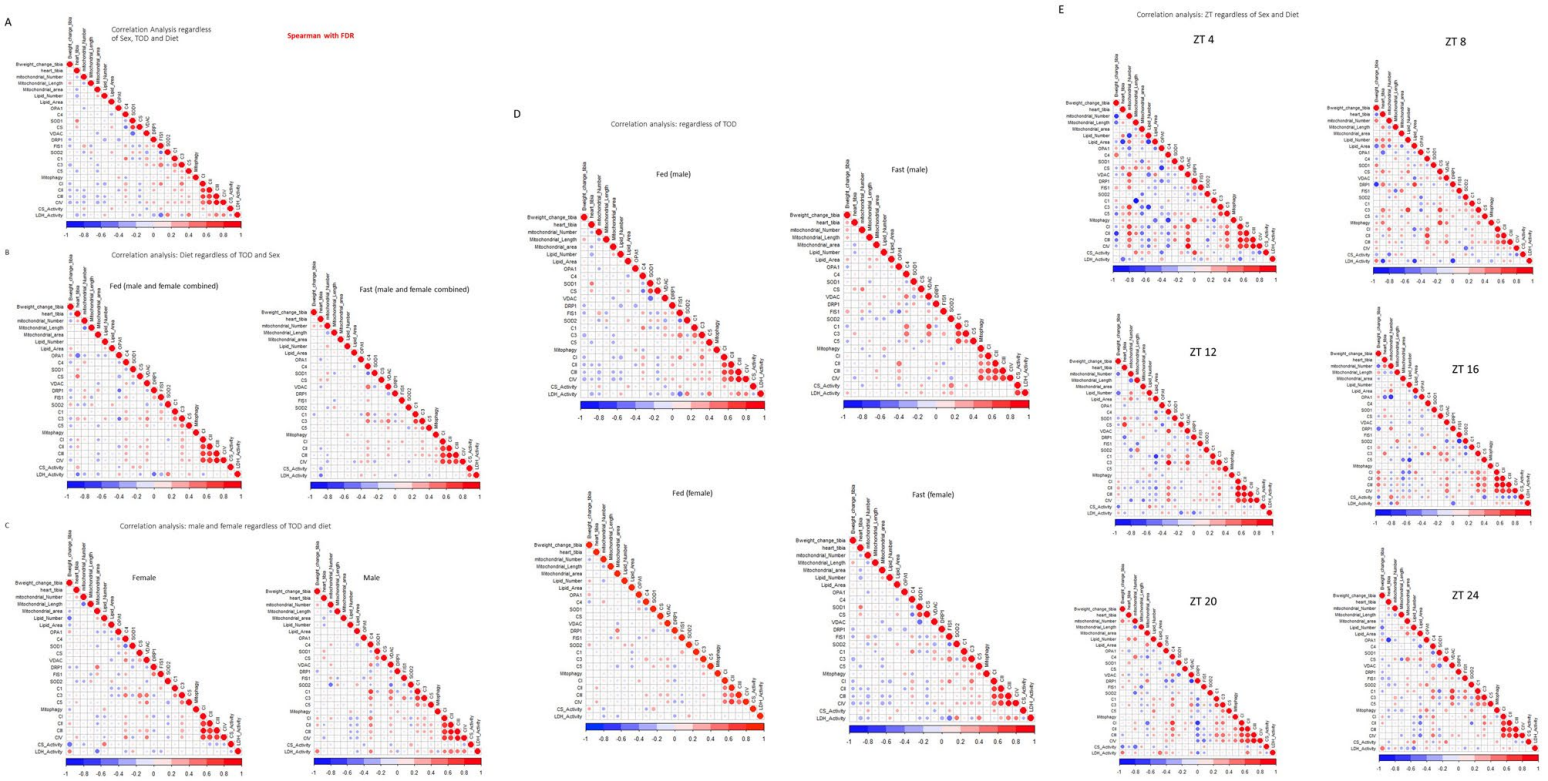
Bi-variant correlation analyses. To examine whether some of the measured parameters of mitochondrial function, mitochondrial morphology, mitochondrial quality control, and levels of protein related to mitochondrial structure and function, we performed Spearman correlation study with FDR. Bweight_change_tibia is the BW change (%)/TL (mm) from Fig. 1D. Heart_tibia is BVW/TL in Fig. 1C. C1, C3, C4 and C5 are the protein levels of Fig. 6E–H. CI, CII, CIII and CIV are the ETC activities of Fig. 6A–D. Mitochondrial_Length, Mitochondrial_area, Lipid_Number, and Lipid-Area are from the EM analyses. The color scheme under the graph indicates R^2^. (**A**) Correlations when all animals, both sexes, with and without fasting, and harvested at different ZTs are included. (**B**) Correlations in all fed versus all fasting animals (regardless of TOD and sex). (**C**) Correlations in all male versus all female animals (regardless of TOD or fasting). (**D**) Correlations in male fed, male fast, female fed, and female fast. (**E**) Correlations in different ZTs (regardless of sex or fasting).

To further analyze how different parameters are coordinately integrated to modulate mitochondrial quality control in each individual mouse, we performed bi-variant analysis as previously performed by our group^41,50–53^. Parameters for each mouse were plotted and multiple comparisons analysis (spearman) was performed, correcting for false discoveries. First, we analyzed how different parameters were coordinately regulated irrespective of TOD, sex, and fasting status. To determine whether individual animal has any of the parameters coordinately regulated, we performed bi-variant analyses with the measured parameters of mitochondrial function, mitochondrial morphology, mitochondrial quality control, and levels of protein related to mitochondrial structure and function, with Spearman correlation study with FDR. We found that mitochondrial complexes III and IV are always positively coordinately regulated with a high correlation coefficient (Fig. 10A). Complex I and II are also positively coordinately regulated with complex III and IV albeit slightly lower correlation coefficient. SOD1 is positively coordinated with citrate synthase protein levels and negatively regulated with VDAC protein levels. LDH activity is negatively coordinated with Fis1 protein level. Mitophagy flux level does not correlate with any of the parameters.

Next, we analyzed the impact of diet in how these parameters are coordinately regulated. OPA1 is negatively correlated with heart weight/tibia in the fed but not the fasted animals. There is a modest correlation between complex IV subunit MTCO1 with complex I subunit NDUFA9 and complex III subunit UQCRC1 levels in the fasting animals. The coordinate regulation among mitochondrial complexes I-IV still remains as the strongest correlated parameters after separating fed versus fast (Fig. 10B). Likewise, separating male versus female animals, the coordinate regulation among mitochondrial complexes I-IV remains the strongest correlated parameters (Fig. 10C). Body weight is negatively correlated with lipid droplet numbers and citrate synthase activity in the female. Mitochondrial area is positively correlated with DRP1 protein levels in the female. Complex III subunit UQCRC1 is positively correlated with complex IV subunit MTCO1, citrate synthase, and VDAC protein levels in the female. In the male, complex IV subunit MTCO1 is positively correlated with complex I NDUFA9 protein level.

Further separating male fed, male fasting, female fed and female fasted groups, the coordinate regulation among mitochondrial complexes I-IV remains as the strongest correlated parameters (Fig. 10D). LDH activity is positively correlated with citrate synthase activity in fasting males, while negatively correlated with Fis1 protein levels in all groups except fasting males. Sod1 proteins levels are positively correlated with citrate synthase protein levels and negatively correlated with VDAC protein levels in all groups except fasting males.

Analyzing each ZT, there are more correlations between parameters measured (Fig. 10E). The coordinate regulation among mitochondrial complexes I-IV remains strong at all ZTs except ZT8.

At ZT4, body weight is negatively correlated with mitochondrial number, and complex II activity. Heart weight/tibia length is negatively correlated with lipid droplet number. Mitochondrial number is negatively correlated with lipid droplet number, but positively correlated with VDAC protein level, Complex III subunit UQCRC1 level, and complex I, II, III and IV activities. Lipid droplet number is also negatively correlated with lipid area and complex III subunit UQCRC1 protein level. OPA1 protein level is negatively correlated with citrate synthase protein level. Complex IV subunit MTCO1 subunit protein level is negatively correlated, and Drp1 protein level is positively correlated with mitophagy. VDAC protein level is positively correlated with complex I, II, III, and IV activities.

At ZT8, the coordinated regulation among mitochondrial complexes I-III to complex IV is weakened. Positive correlations include CII activity with complex IV MTCO1 subunit protein level; citrate synthase protein level with complex III UQCRC1 protein level and mitophagy flux; complex III UQCRC1 protein level and lipid droplet number. Negative correlations include body weight with DRP1 protein level, LDH activity with heart weight, lipid area and DRP1 protein level.

As with ZT4, at ZT12, 16, 20, and 24, mitochondrial complexes I-IV activities are positively correlated. There were new positive correlations at ZT12 for complex III UQCRC1 and IV MTCO1 subunit protein levels; negative correlation at ZT16 for OPA1 protein level and mitochondrial number; negative correlations at ZT20 between DRP1 and complex I activity; and a negative correlation at ZT24 between heart weight and OPA1 protein level. Overall, these multiple comparison analyses show that ETC activities are the most coordinately regulated irrespective of fasting, and across most of the TOD except ZT8, albeit the strength of interactions between individual complexes are sex dependent.

## Discussion

The goal of this study was to determine the effects of TOD, fasting, and sex on mitochondrial structure, function, and mitophagy in the heart. To achieve this, we measured the sizes and number of mitochondria, mitochondrial ETC complexes, CS and LDH activities, and levels of proteins related to mitochondrial function and mitophagy. We found that mitochondrial number is dependent on TOD, while mitochondrial function and mitophagy are affected by sex, fasting, and TOD, in mouse hearts. Furthermore, we demonstrated that mitochondrial numbers and mitophagy regulation are dependent on the circadian clock transcription factor BMAL1 in cardiomyocytes. Fasting significantly decreased mitochondrial complexes I-IV activities and levels of key mitochondrial proteins, and increased mitophagy flux. Significant sex-dependent parameters are demonstrated with LDH activity, and levels of VDAC, SOD1, and fission and fusion protein. We further demonstrated significant sex × fasting, TOD × fasting, or sex × TOD interactions on many of the mitochondrial/metabolic activities and protein levels measured. Our findings are summarized in Supplementary Tables 1, 2 and Fig. 8.

Mice show a TOD regulation regarding their body weight, food intake, physical activity, energy expenditure, and respiratory exchange ratio, which are higher during the dark phase and lower during the light phase^54^. As prior studies demonstrated^54^, we found a body weight difference during the dark phase versus the light phase in the males (One-way ANOVA ZT12 versus ZT0 p = 0.0471). We did not find such differences in the females, which is consistent with previous studies depicting differences in metabolic rates between males and females^55^. In addition to body weight, mitochondrial and lipid droplet numbers are dependent on TOD in both males and females. Other parameters that are significant across TOD with 3-way ANOVA are due to combined fasting duration effects that are concurrent with different TOD. Although mitochondrial complexes I-IV activities were not dependent on TOD, complexes I-III activities normalized to CS activities were dependent on TOD, when analyzing both sexes and both fed and fasting groups together. We performed Spearman with FDR correlation analyses and found a modest positive correlation of CS activity with mitochondrial number in fed males, fed females and fasted females, and a modest negative correlation with mitochondrial number in fasted males. This may reflect changes in relative mitochondrial protein content, which may partially involve transcription factors implicated in mitochondrial biogenesis or involve mitochondrial protein degradation.

Sex-differences are increasingly recognized in heart physiology and diseases^56,57^. Sex-dependent differences have been reported in ATP production rate in skeletal muscle mitochondria^58,59^, and in state 3 and state 4 respiration in cardiac mitochondria isolated from the interfibrillar (IFM) and subsarcolemmal (SSM) structure^60^. In cardiac tissue samples of human inflammatory dilated cardiomyopathy patients, there are both age and sex effects observed in the levels of mRNAs of NDUSF1, ND4, and COX1, and proteins of AMPK, p-AMPK, Tom40 and PGC1α^61^. Furthermore, metabolomics in the heart exhibits significant differences in sedentary mice and in response to exercise between sexes, even though the same exercise regime did not affect respiration in isolated mitochondria^62^. We have also reported sex-dependent network interactions between parameters of mitochondrial function and autophagy components^41^. In the current study, we found significant sex-dependent differences in multiple parameters. Indeed, heart weight/tibia were lower in females than males. There was also a marked sex difference in LDH activities. Mitochondrial complex I and II substrate-dependent activities and CS activities were differentially affected by fasting × sex, indicating male and female hearts exhibit different responses to fasting, likely related to energy storage^63,64^. Mitochondrial complex IV substrate-dependent activities, mitochondrial number, lipid droplet numbers, and mitophagy are differentially affected by TOD × sex. Furthermore, complex V ATP5A1 protein is higher in males than females. Interestingly, OPA1, DRP1, and FIS1 protein levels are all different between sexes with higher OPA and lower DRP1 and FIS1 in female hearts, suggesting that females have a higher capacity for fusion and a lower capacity for fission. Interestingly, mitochondrial fragmentation was greater in the females at ZT12 (8 h after fasting) (Fig. 2E), a time point when in females OPA1 was significantly decreased. Although one limitation of our study was that we did not assess the estrus stage for the female mice, the standard deviation in the females is not significantly bigger than in the males, suggesting that it had little effect on our measurements. With regard to the potential mechanisms by which sex regulates mitochondrial structure and function in the heart, hormonal effects, sex-dependent gene and protein expression, as well as network interactions of multiple biochemical parameters, have all been proposed to play a role^3,41,65–67^.

Complex relationships between sex and TOD were found to exist for body weight, complex IV substrate-linked activities, complex III subunit UQCRC1 and SOD2 protein level, mitochondrial number, mitochondrial-derived vesicle numbers (MDVs), lipid droplet number, and mitophagy. These interactions may underlie different responses of males and females to starvation, TOD, or both. There have been no prior studies investigating sex differences in fission and fusion proteins in the heart across different TOD. Our results demonstrate the importance of sex, food intake and TOD interactions, as sex differences become relevant in the context of TOD or food intake.

In response to fasting, in addition to body weight, mitochondrial activities, mitochondrial ETC proteins, and outer membrane VDAC protein levels were all decreased. The dramatic and immediate (within 4 h) response of loss of mitochondrial ETC activities cannot be explained by a loss of mitochondrial mass, as citrate synthase activities were rather paradoxically increased, potentially due to post-translational modification. Furthermore, the magnitude of decrease in ETC activities far exceeds the loss of mitochondrial number, and to a certain extent parallels with loss of ETC proteins, suggesting that the rapid response to fasting in mitochondrial protein and activities may be mediated by degradation of the outer and inner membrane sparing the matrix by mitophagy or by mitochondrial specific proteases. The significant increase of antioxidant response protein SOD2, fission protein DRP1, and mitophagy may contribute to the loss of ETC activities or possibly in response to the loss of the ETC activities. The increase in DRP1 is consistent with a mechanism where fission facilitates mitophagy^25^. The increase in SOD2 is consistent with the observation that nutrient deprivation significantly increases the production of reactive oxygen species (ROS), which triggers the response of elevation of antioxidants^48,49^. Noted that MDVs are not sensitive to fasting, consistent with prior study that MDVs occurs in the heart under normal healthy conditions^46,68^. It has been proposed that decreased food intake can account for some of the metabolic changes across TOD. On the other hand, under normal conditions, mice never experience true fasting as some food consumption remains even during the inactive phase. This may partially explain why more dramatic changes in ETC activities, mitochondrial proteins, and mitophagy occur in response to fasting, while TOD-dependent changes are restricted to parameters related to mitochondrial number.

Further, TOD × fasting is significant for body weight, % body weight change, biventricular heart weight change, CS activity, complexes I-III activities when normalized to CS activities, mitochondrial number, lipid droplet number, and lipid droplet area. Finally, TOD × sex × fasting is significant for SOD1 protein level. The importance of the cardiomyocyte circadian clock in regulating mitochondrial number and mitophagy is further supported by our observation that with BMAL1 knockout in cardiomyocytes (CBK mice), mitochondrial number and mitophagy regulation are significantly different from those in the wildtype heart. One limitation of the current study was that we only used male CBK mice in these specific studies. Further studies will investigate if mitophagy is differentially regulated in female CBK mice.

The use of mito-QC mice facilitated our analyses of mitophagy in vivo. Although we detected a significant sex difference in FIS1 protein levels, and the mitochondrial targeting sequence from FIS1 was used to express mito-QC, we did not see a significant difference in mito-QC level according to the dim red signal between sexes at ZT4, but there was a sex difference across all TOD (Supplemental Fig. 1). This may reflect that mito-QC protein levels may be dependent on mitochondrial targeting, although at the present we cannot exclude the possibility that mito-QC synthesis and degradation may be sex dependent. In addition, a recent study has indicated that mt-Keima transgenic mice may be more sensitive for mitophagy analyses due to the expression of the transgene in the mitochondrial matrix rather than the outer membrane^69,70^. Thus, our data may be an underestimation of the oscillation of the mitophagy changes induced by fasting or the interaction of TOD × sex.

The determination of mitochondrial function, number, and mitophagy in the same animals allowed us to analyze whether these are correlated events. In contrast to our previous studies^41^, we did not find many correlations except that there was in general a close positive correlation of mitochondrial complex I-IV activities except for ZT8. As we found significant sex, fasting and TOD-dependent regulations on multiple parameters of mitochondrial structure and function, metabolism-related protein levels and lipid droplet accumulation, as well as mitophagy, suggesting that some of these parameters are independently regulated. For example, even though mitochondrial complex activities and the protein levels of their respective representative subunits were decreased by fasting, they are not directly proportional to each other. Alternatively, this study has only n = 3–6 mice for each sex, fasting status and ZT, and correlations may be more evident if the number of animals were higher. Future studies will be necessary to further explore mechanisms of sex, fasting, and TOD-dependent regulation of metabolic events and mitophagy.

In conclusion, this is the first extensive study on mitochondrial ultrastructure, ETC activities, and mitophagy across TOD, with both sexes, and in response to fasting. This study provides valuable insights into how mitochondrial function and quality control are not only regulated by TOD, sex, and fasting, but also by their interactions in the heart under physiological conditions, which will aid future studies of how these interactions are regulated. We found that in response to fasting, significant depletion of mitochondrial ETC activities are associated with LDH activity decreases, and not associated with significant morphological or matrix enzyme CS activity changes. This observation calls for further studies of the mechanisms of how different compartment of the mitochondria are degraded in response to nutrient availability. These cell biological and biochemical differences across TOD, between sexes and in response to fasting, even though were under non-disease conditions, may provide a foundation to better understand the mechanisms of the complex sex-differences between male and female hearts, including size, cardiac output, ejection fraction and myocardial oxygen consumption^57^. In addition, since most research was tailored to the male heart, cardiac disease prevalence, severity and mortality in the female population will need to be better investigated. We demonstrated that many gravimetric, mitochondrial, and mitophagy parameters are with different basal levels in males and females, and peak and trough at different ZTs in males and females. The extents of responses to fasting also show divergence between sexes. These findings further emphasize the need to consider sex-related differences in mitochondrial function, quality control and metabolism in a broader sense in health and disease. The wealth of the information gathered in this study highlight the importance that we are in urgent need to identify and determine the specifics and breadth of sex differences in physiology, in response to nutrient abundance, and in response to circadian clock. Taken together, the observations presented in this study is unprecedented in its kind and lay the foundation for identification of the mechanisms of how TOD, sex, and fasting impact mitochondrial structure, function and quality control, and how they change in physiological stress, aging, diseases, and responses to pharmacological interventions.

### Supplementary Information

Supplementary Legends.

Supplementary Information.

Supplementary Figure S1.

Supplementary Tables.

## Data Availability

All data generated or analyzed during this study are included in this published article [and its supplementary information files].

## References

[1] Lu JY, Simon M, Zhao Y, Ablaeva J, Corson N, Choi Y (2022). Comparative transcriptomics reveals circadian and pluripotency networks as two pillars of longevity regulation. Cell Metab..

[2] Lopaschuk GD, Karwi QG, Tian R, Wende AR, Abel ED (2021). Cardiac energy metabolism in heart failure. Circ. Res..

[3] Oliva M, Munoz-Aguirre M, Kim-Hellmuth S, Wucher V, Gewirtz ADH, Cotter DJ (2020). The impact of sex on gene expression across human tissues. Science.

[4] Humphries KH, Izadnegahdar M, Sedlak T, Saw J, Johnston N, Schenck-Gustafsson K (2017). Sex differences in cardiovascular disease - Impact on care and outcomes. Front. Neuroendocrinol..

[5] Austad SN, Fischer KE (2016). Sex differences in lifespan. Cell Metab..

[6] Redmann M, Dodson M, Boyer-Guittaut M, Darley-Usmar V, Zhang J (2014). Mitophagy mechanisms and role in human diseases. Int. J. Biochem. Cell Biol..

[7] Ma X, McKeen T, Zhang J, Ding WX (2020). Role and mechanisms of mitophagy in liver diseases. Cells..

[8] Collins H, Kane M, Litovsky S, Darley-Usmar V, Young M, Chatham J (2021). Mitochondrial morphology and mitophagy in heart diseases: qualitative and quantitative analyses using transmission electron microscopy (TEM). Frontier in Aging..

[9] Liang WJ, Gustafsson AB (2020). The aging heart: Mitophagy at the center of rejuvenation. Front. Cardiovasc. Med..

[10] Bray MS, Shaw CA, Moore MW, Garcia RA, Zanquetta MM, Durgan DJ (2008). Disruption of the circadian clock within the cardiomyocyte influences myocardial contractile function, metabolism, and gene expression. Am. J. Physiol .Heart Circ. Physiol..

[11] Chatham JC, Young ME (2013). Regulation of myocardial metabolism by the cardiomyocyte circadian clock. J. Mol. Cell Cardiol..

[12] Durgan D, Trexler N, Egbejimi O, McElfresh T, Suk H, Petterson L (2006). The circadian clock within the cardiomyocyte is essential for responsiveness of the heart to fatty acids. J. Biol. Chem..

[13] Durgan DJ, Pat BM, Laczy B, Bradley JA, Tsai JY, Grenett MH (2011). O-GlcNAcylation, novel post-translational modification linking myocardial metabolism and cardiomyocyte circadian clock. J. Biol. Chem..

[14] Zhang J, Chatham JC, Young ME (2020). Circadian regulation of cardiac physiology: rhythms that keep the heart beating. Annu. Rev. Physiol..

[15] Kondratov RV, Kondratova AA, Gorbacheva VY, Vykhovanets OV, Antoch MP (2006). Early aging and age-related pathologies in mice deficient in BMAL1, the core componentof the circadian clock. Genes Dev..

[16] Lefta M, Campbell KS, Feng HZ, Jin JP, Esser KA (2012). Development of dilated cardiomyopathy in Bmal1-deficient mice. Am. J. Physiol. Heart Circ. Physiol..

[17] Young ME, Brewer RA, Peliciari-Garcia RA, Collins HE, He L, Birky TL (2014). Cardiomyocyte-specific BMAL1 plays critical roles in metabolism, signaling, and maintenance of contractile function of the heart. J. Biol. Rhythms..

[18] Young ME (2016). Temporal partitioning of cardiac metabolism by the cardiomyocyte circadian clock. Exp. Physiol..

[19] Kohsaka A, Das P, Hashimoto I, Nakao T, Deguchi Y, Gouraud SS (2014). The circadian clock maintains cardiac function by regulating mitochondrial metabolism in mice. PLoS One..

[20] Li E, Li X, Huang J, Xu C, Liang Q, Ren K (2020). BMAL1 regulates mitochondrial fission and mitophagy through mitochondrial protein BNIP3 and is critical in the development of dilated cardiomyopathy. Protein Cell..

[21] Zhang J (2013). Autophagy and mitophagy in cellular damage control. Redox Biol..

[22] Hill BG, Shiva S, Ballinger S, Zhang J, Darley-Usmar VM (2019). Bioenergetics and translational metabolism: Implications for genetics, physiology and precision medicine. Biol. Chem..

[23] Kim I, Rodriguez-Enriquez S, Lemasters JJ (2007). Selective degradation of mitochondria by mitophagy. Arch. Biochem. Biophys..

[24] Liang W, Moyzis AG, Lampert MA, Diao RY, Najor RH, Gustafsson AB (2020). Aging is associated with a decline in Atg9b-mediated autophagosome formation and appearance of enlarged mitochondria in the heart. Aging Cell..

[25] Kim I, Lemasters JJ (2011). Mitochondrial degradation by autophagy (mitophagy) in GFP-LC3 transgenic hepatocytes during nutrient deprivation. Am. J. Physiol. Cell Physiol..

[26] Nguyen TN, Padman BS, Usher J, Oorschot V, Ramm G, Lazarou M (2016). Atg8 family LC3/GABARAP proteins are crucial for autophagosome-lysosome fusion but not autophagosome formation during PINK1/Parkin mitophagy and starvation. J. Cell Biol..

[27] Schiavi A, Maglioni S, Palikaras K, Shaik A, Strappazzon F, Brinkmann V (2015). Iron-starvation-induced mitophagy mediates lifespan extension upon mitochondrial stress in *C. elegans*. Curr. Biol..

[28] Zhen Y, Spangenberg H, Munson MJ, Brech A, Schink KO, Tan KW (2020). ESCRT-mediated phagophore sealing during mitophagy. Autophagy.

[29] McWilliams TG, Prescott AR, Allen GF, Tamjar J, Munson MJ, Thomson C (2016). mito-QC illuminates mitophagy and mitochondrial architecture in vivo. J. Cell Biol..

[30] Katayama H, Kogure T, Mizushima N, Yoshimori T, Miyawaki A (2011). A sensitive and quantitative technique for detecting autophagic events based on lysosomal delivery. Chem. Biol..

[31] McWilliams TG, Prescott AR, Villarejo-Zori B, Ball G, Boya P, Ganley IG (2019). A comparative map of macroautophagy and mitophagy in the vertebrate eye. Autophagy.

[32] Livingston MJ, Wang J, Zhou J, Wu G, Ganley IG, Hill JA (2019). Clearance of damaged mitochondria via mitophagy is important to the protective effect of ischemic preconditioning in kidneys. Autophagy.

[33] Singh F, Prescott AR, Ball G, Reith AD, Ganley IG (2020). Pharmacological rescue of impaired mitophagy in Parkinson’s disease-related LRRK2 G2019S knock-in mice. Elife.

[34] McWilliams TG, Prescott AR, Montava-Garriga L, Ball G, Singh F, Barini E (2018). Basal mitophagy occurs independently of PINK1 in MOUSE TISSUES OF HIGH METABOLIC DEMANd. Cell Metab..

[35] Zhao JF, Rodger CE, Allen GFG, Weidlich S, Ganley IG (2020). HIF1alpha-dependent mitophagy facilitates cardiomyoblast differentiation. Cell Stress..

[36] Mia S, Kane MS, Latimer MN, Reitz CJ, Sonkar R, Benavides GA (2020). Differential effects of REV-ERBalpha/beta agonism on cardiac gene expression, metabolism, and contractile function in a mouse model of circadian disruption. Am. J. Physiol. Heart Circ. Physiol..

[37] McGinnis GR, Tang Y, Brewer RA, Brahma MK, Stanley HL, Shanmugam G (2017). Genetic disruption of the cardiomyocyte circadian clock differentially influences insulin-mediated processes in the heart. J. Mol. Cell Cardiol..

[38] Durgan DJ, Tsai JY, Grenett MH, Pat BM, Ratcliffe WF, Villegas-Montoya C (2011). Evidence suggesting that the cardiomyocyte circadian clock modulates responsiveness of the heart to hypertrophic stimuli in mice. Chronobiol. Int..

[39] Benavides GA, Mueller T, Darley-Usmar V, Zhang J (2022). Optimization of measurement of mitochondrial electron transport activity in postmortem human brain samples and measurement of susceptibility to rotenone and 4-hydroxynonenal inhibition. Redox Biol..

[40] Acin-Perez R, Benador IY, Petcherski A, Veliova M, Benavides GA, Lagarrigue S (2020). A novel approach to measure mitochondrial respiration in frozen biological samples. EMBO J..

[41] Huynh VN, Benavides GA, Johnson MS, Ouyang X, Chacko BK, Osuma E, Mueller T, Chatham J, Darley-Usmar VM, Zhang J (2022). Acute inhibition of OGA sex-dependently alters the networks associated with bioenergetics, autophagy, and neurodegeneration. Mol. Brain.

[42] Shihan MH, Novo SG, Le Marchand SJ, Wang Y, Duncan MK (2021). A simple method for quantitating confocal fluorescent images. Biochem. Biophys. Rep..

[43] Grigoryan AM, Dougherty ER, Kononen J, Bubendorf L, Hostetter G, Kallioniemi O (2002). Morphological spot counting from stacked images for automated analysis of gene copy numbers by fluorescence in situ hybridization. J. Biomed. Opt..

[44] Benador IY, Veliova M, Mahdaviani K, Petcherski A, Wikstrom JD, Assali EA (2018). Mitochondria bound to lipid droplets have unique bioenergetics, composition, and dynamics that support lipid droplet expansion. Cell Metab..

[45] Tsushima K, Bugger H, Wende AR, Soto J, Jenson GA, Tor AR (2018). Mitochondrial reactive oxygen species in lipotoxic hearts induce post-translational modifications of AKAP121, DRP1, and OPA1 that promote mitochondrial fission. Circ. Res..

[46] Cadete VJ, Deschenes S, Cuillerier A, Brisebois F, Sugiura A, Vincent A (2016). Formation of mitochondrial-derived vesicles is an active and physiologically relevant mitochondrial quality control process in the cardiac system. J. Physiol..

[47] Fang X, Gustafsson AB (2023). DRP1-mediated mitophagy: Safeguarding obese hearts from cardiomyopathy. Circ. Res..

[48] Lee J, Giordano S, Zhang J (2012). Autophagy, mitochondria and oxidative stress: Cross-talk and redox signalling. Biochem. J..

[49] Li L, Chen Y, Gibson SB (2013). Starvation-induced autophagy is regulated by mitochondrial reactive oxygen species leading to AMPK activation. Cell Signal..

[50] Uppal K, Ma C, Go YM, Jones DP, Wren J (2018). xMWAS: A data-driven integration and differential network analysis tool. Bioinformatics..

[51] Chacko BK, Smith MR, Johnson MS, Benavides G, Culp ML, Pilli J (2019). Mitochondria in precision medicine; linking bioenergetics and metabolomics in platelets. Redox Biol..

[52] Go YM, Fernandes J, Hu X, Uppal K, Jones DP (2018). Mitochondrial network responses in oxidative physiology and disease. Free Radic. Biol. Med..

[53] Smith MR, Chacko BK, Johnson MS, Benavides GA, Uppal K, Go YM (2020). A precision medicine approach to defining the impact of doxorubicin on the bioenergetic-metabolite interactome in human platelets. Redox Biol..

[54] Brewer RA, Collins HE, Berry RD, Brahma MK, Tirado BA, Peliciari-Garcia RA (2018). Temporal partitioning of adaptive responses of the murine heart to fasting. Life Sci..

[55] Halsey LG, Careau V, Pontzer H, Ainslie PN, Andersen LF, Anderson LJ (2022). Variability in energy expenditure is much greater in males than females. J. Hum. Evol..

[56] Stolfo D, Uijl A, Vedin O, Stromberg A, Faxen UL, Rosano GMC (2019). Sex-based differences in heart failure across the ejection fraction spectrum: Phenotyping, and prognostic and therapeutic implications. JACC Heart Fail..

[57] St Pierre SR, Peirlinck M, Kuhl E (2022). Sex matters: A comprehensive comparison of female and male hearts. Front. Physiol..

[58] Karakelides H, Irving BA, Short KR, O'Brien P, Nair KS (2010). Age, obesity, and sex effects on insulin sensitivity and skeletal muscle mitochondrial function. Diabetes.

[59] Guevara R, Santandreu FM, Valle A, Gianotti M, Oliver J, Roca P (2009). Sex-dependent differences in aged rat brain mitochondrial function and oxidative stress. Free Radic. Biol. Med..

[60] Ribeiro RF, Ronconi KS, Morra EA, Do Val Lima PR, Porto ML, Vassallo DV (2016). Sex differences in the regulation of spatially distinct cardiac mitochondrial subpopulations. Mol. Cell Biochem..

[61] Barcena ML, Tonini G, Haritonow N, Breiter P, Milting H, Baczko I (2023). Sex and age differences in AMPK phosphorylation, mitochondrial homeostasis, and inflammation in hearts from inflammatory cardiomyopathy patients. Aging Cell..

[62] Fulghum K, Collins HE, Jones SP, Hill BG (2022). Influence of biological sex and exercise on murine cardiac metabolism. J. Sport Health Sci..

[63] Soeters MR, Sauerwein HP, Groener JE, Aerts JM, Ackermans MT, Glatz JF (2007). Gender-related differences in the metabolic response to fasting. J. Clin. Endocrinol. Metab..

[64] Mauvais-Jarvis F (2015). Sex differences in metabolic homeostasis, diabetes, and obesity. Biol. Sex Differ..

[65] Austad SNBS, Buford TW, Carter CS, Smith DL, Darley-Usmar V, Zhang J (2021). Targeting whole body metabolism and mitochondrial bioenergetics in the drug development for Alzheimer’s disease. Acta Pharm. Sin. B.

[66] Nguyen TTL, Wang M, Liu D, Iyer S, Gonzalez Bonilla HM, Acker N (2022). Proteomic biomarkers of sacubitril/valsartan treatment response in heart failure with preserved ejection fraction: Molecular insights into sex differences. Circ Heart Fail..

[67] Kwan A, Demosthenes E, Salto G, Ouyang D, Nguyen T, Nwabuo CC (2022). Cardiac microstructural alterations measured by echocardiography identify sex-specific risk for heart failure. Heart.

[68] Dengjel J, Kristensen AR, Andersen JS (2008). Ordered bulk degradation via autophagy. Autophagy.

[69] Liu YT, Sliter DA, Shammas MK, Huang X, Wang C, Calvelli H (2021). Mt-Keima detects PINK1-PRKN mitophagy in vivo with greater sensitivity than mito-QC. Autophagy.

[70] Liu YT, Sliter DA, Shammas MK, Huang X, Wang C, Calvelli H (2021). Comment on "mt-Keima detects PINK1-PRKN mitophagy in vivo with greater sensitivity than mito-QC". Autophagy.

