# Supplementary material for: The interplay between sex, time of day, fasting status, and their impact on cardiac mitochondrial structure, function, and dynamics
Source: Sci Rep. 2023 Dec 7;13:21638. doi: 10.1038/s41598-023-49018-z (PMC10703790; doi:10.1038/s41598-023-49018-z)
Supplement: Supplementary file 1 — Supplementary Legends. [file 41598_2023_49018_MOESM1_ESM.docx]

**Supplementary File: Original western blots and associated Ponceau-S staining images as loading controls.** All odd gel numbers are from female mice. All even gel numbers are male mice. Each lane is from an individual mouse, except a ZT4 anchor lane is always included to aid normalization between gels. Antibodies used are indicated. Randomized loading was performed to randomize potential position effects on western blots. Dates of the experiments are provided as the same samples were electrophoresed and probed on different dates. Lanes that are technical or statistical outliers which are excluded from statistics are indicated.

**Supplementary Figure 1**: **Level of mito-QC protein in fed or fast male and female MQC cardiac tissues.** Confocal images from cardiac tissues isolated from 20-week-old fed male and female MQC mice at different time of day. Expression of the transgene is evaluated by the global background red intensity reported here as dim red normalized by the total tissue area. Data are reported for 4-6 mice per experimental group. ZT4 fed male and female are not significantly different by Student t-test. However there is a sex effect (lower in female) if all time-of-day are included (p<0.0001, three-way ANOVA).

**Supplementary Table 1: One-way ANOVA *post hoc* table for TOD effect after one-way ANOVA.** Only those significant are listed.

**Supplementary Table 2: Summary of all significant regulations by 3-way ANOVA.**
