# Supplementary material for: The interplay between sex, time of day, fasting status, and their impact on cardiac mitochondrial structure, function, and dynamics
Source: Sci Rep. 2023 Dec 7;13:21638. doi: 10.1038/s41598-023-49018-z (PMC10703790; doi:10.1038/s41598-023-49018-z)
Supplement: Supplementary file 2 — Supplementary Information. [file 41598_2023_49018_MOESM2_ESM.pdf]

Week 1

OPA1 (BD T 612606), Complex IV (Abcam, ab14705)
March 1-4, 2021

all odd gel numbers are female and all even gel numbers are male

Gel 1

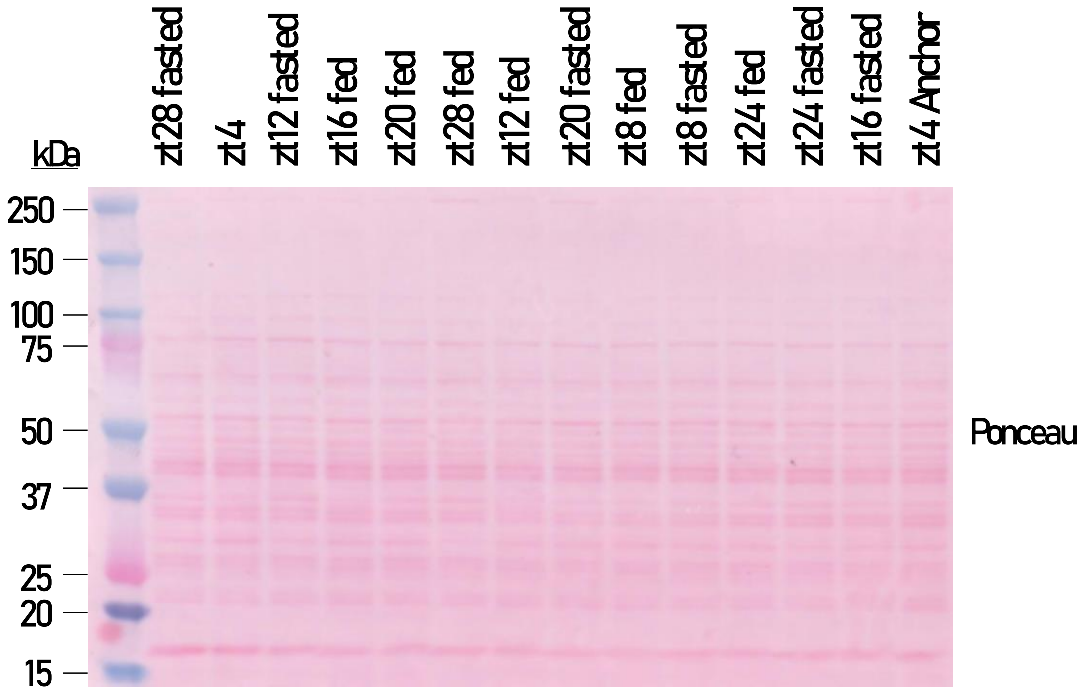
kDa
250
150
100
75
50
37
25
20
15
zt28 fasted
zt4
zt12 fasted
zt16 fed
zt20 fed
zt28 fed
zt12 fed
zt20 fasted
zt8 fed
zt8 fasted
zt24 fed
zt24 fasted
zt16 fasted
zt4 Anchor
Ponceau

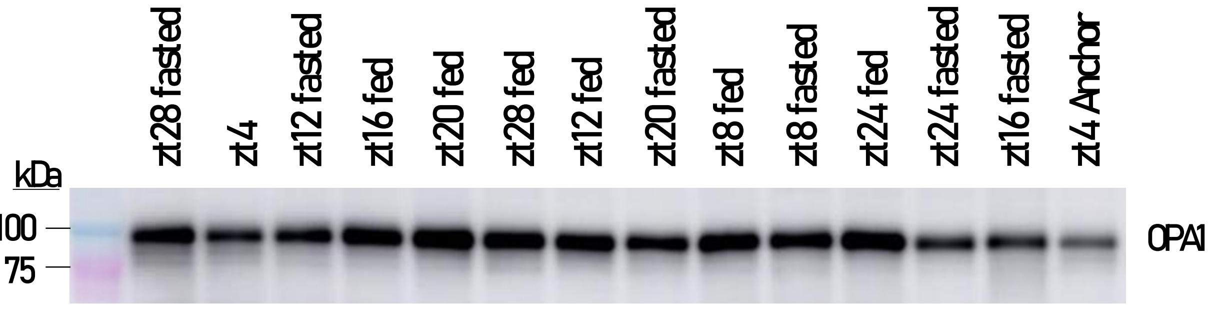
kDa
100
75
zt28 fasted
zt4
zt12 fasted
zt16 fed
zt20 fed
zt28 fed
zt12 fed
zt20 fasted
zt8 fed
zt8 fasted
zt24 fed
zt24 fasted
zt16 fasted
zt4 Anchor
OPA1

Gel 1b

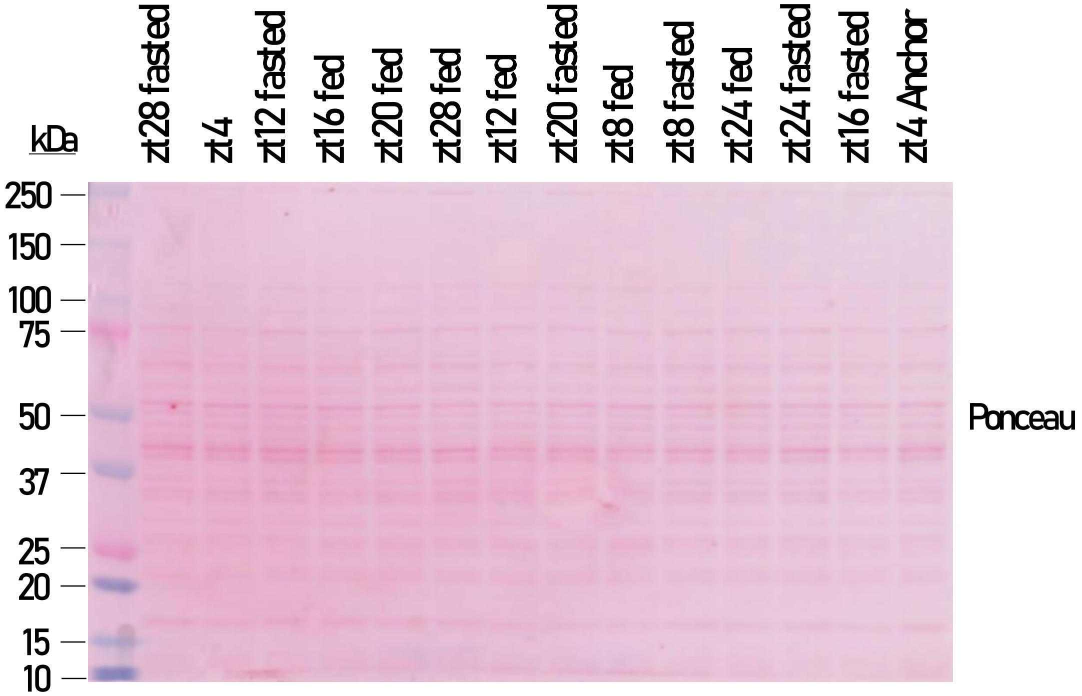
zt28 fasted
zt4
zt12 fasted
zt16 fed
zt20 fed
zt28 fed
zt12 fed
zt20 fasted
zt8 fed
zt8 fasted
zt24 fed
zt24 fasted
zt16 fasted
zt4 Anchor
kDa
250
150
100
75
50
37
25
20
15
10
Ponceau

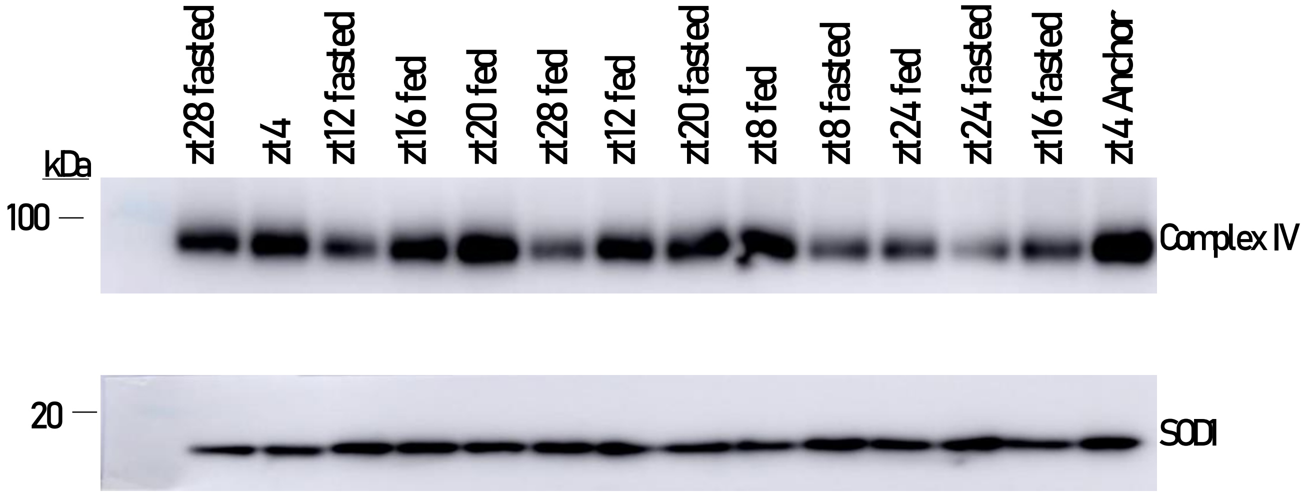
zt28 fasted
zt4
zt12 fasted
zt16 fed
zt20 fed
zt28 fed
zt12 fed
zt20 fasted
zt8 fed
zt8 fasted
zt24 fed
zt24 fasted
zt16 fasted
zt4 Anchor
kDa
100
Complex IV
20
SOD1

Gel 2

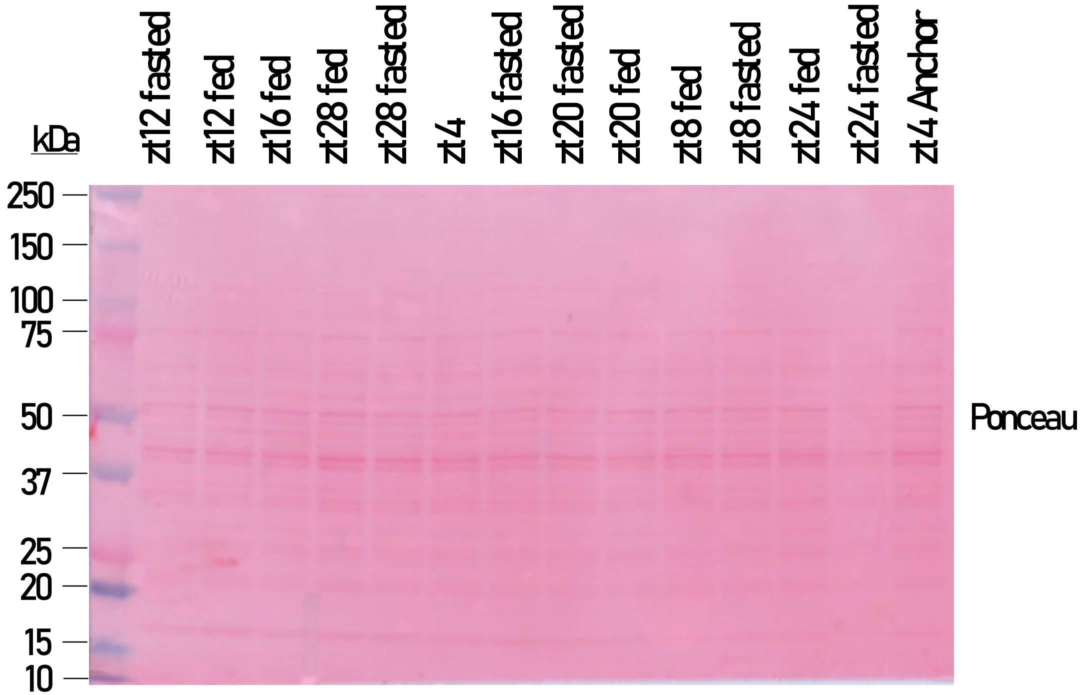
kDa
250
150
100
75
50
37
25
20
15
10
zt12 fasted
zt12 fed
zt16 fed
zt28 fed
zt28 fasted
zt4
zt16 fasted
zt20 fasted
zt20 fed
zt8 fed
zt8 fasted
zt24 fed
zt24 fasted
zt4 Anchor
Ponceau

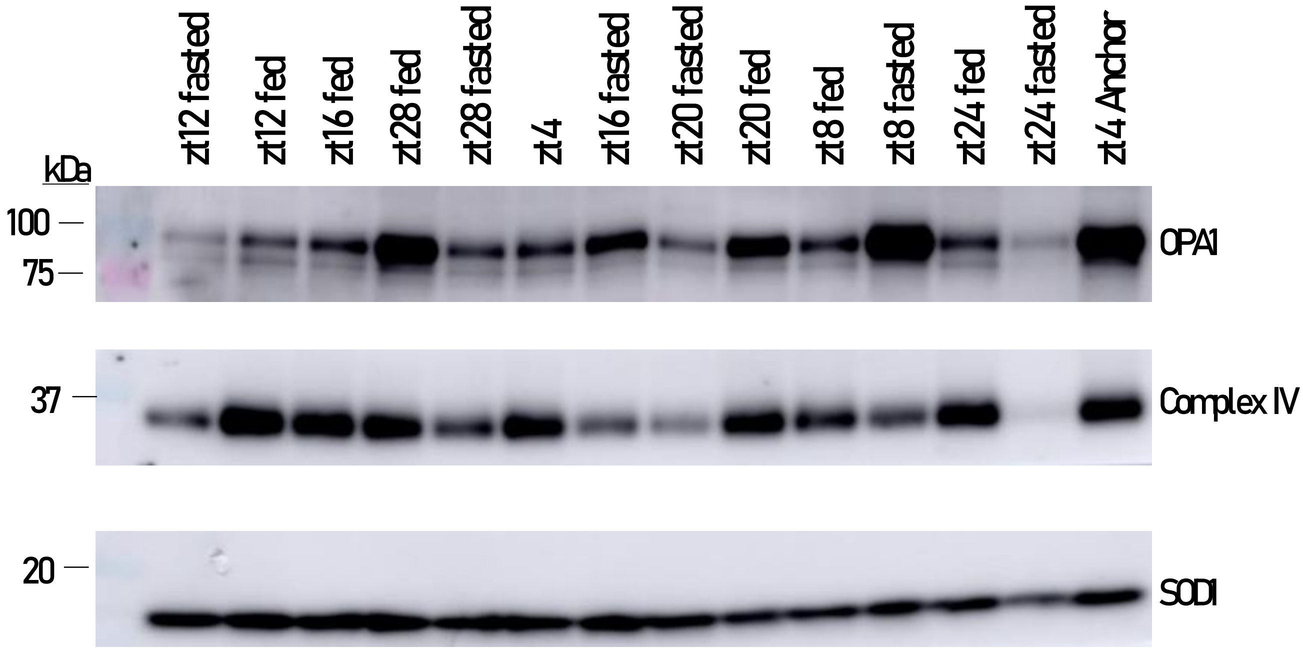
kDa
100
75
37
20
zt12 fasted
zt12 fed
zt16 fed
zt28 fed
zt28 fasted
zt4
zt16 fasted
zt20 fasted
zt20 fed
zt8 fed
zt8 fasted
zt24 fed
zt24 fasted
zt4 Anchor
OPA1
Complex IV
SOD1

Gel 3

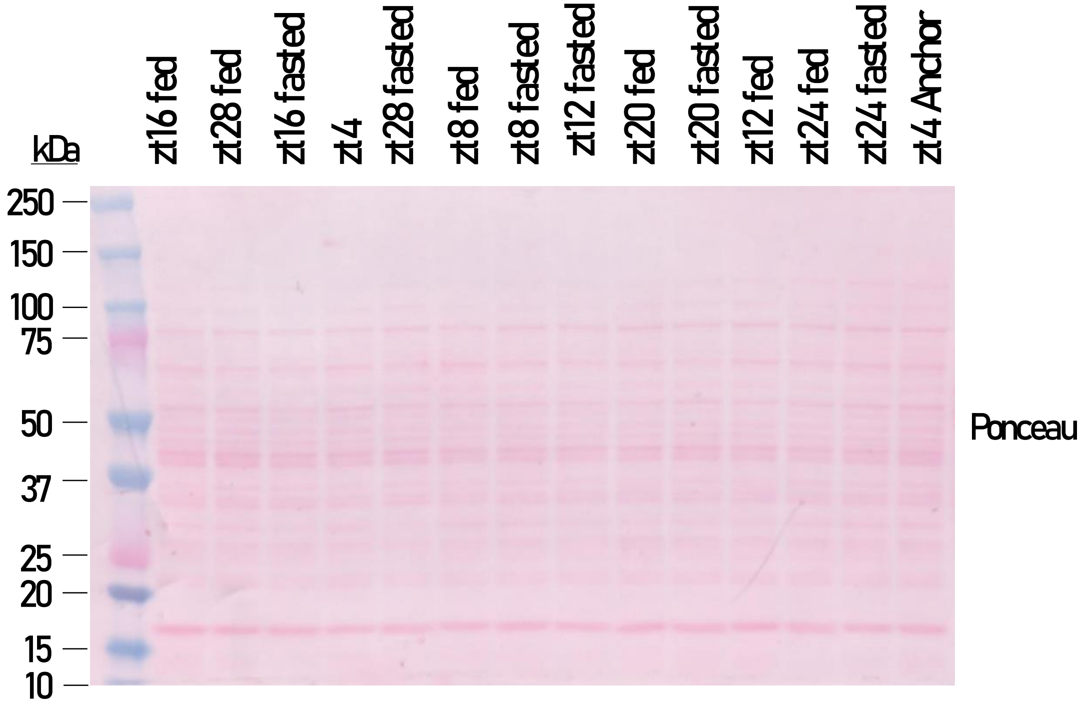

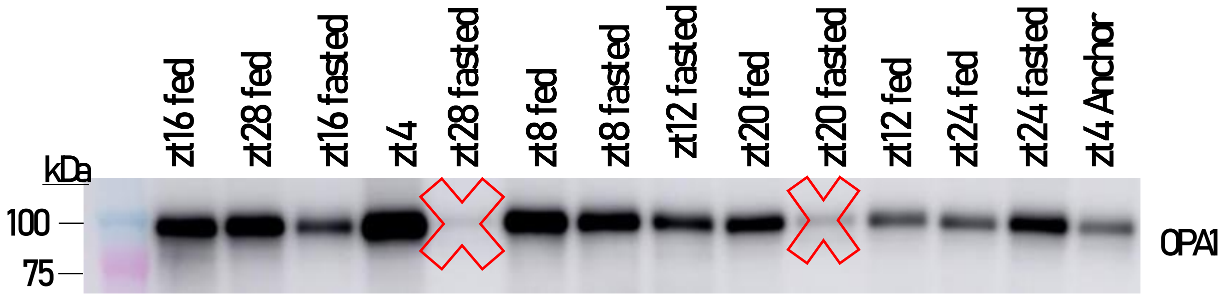

*ZT28 fast and ZT20 fast excluded, signal too low

Gel 3b

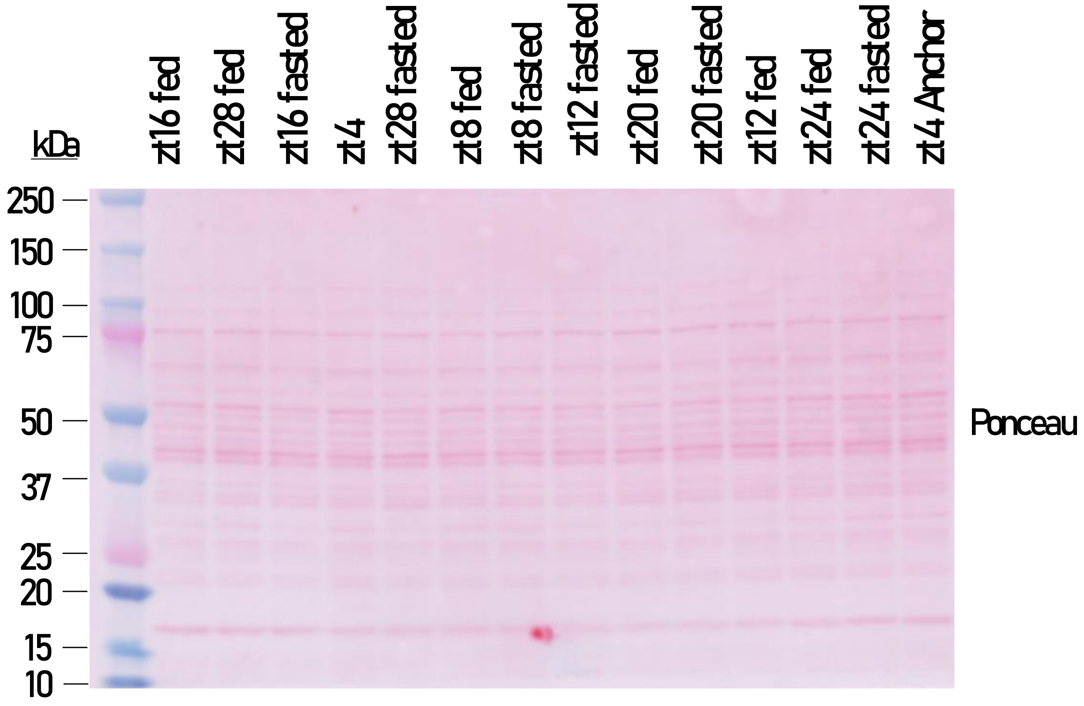

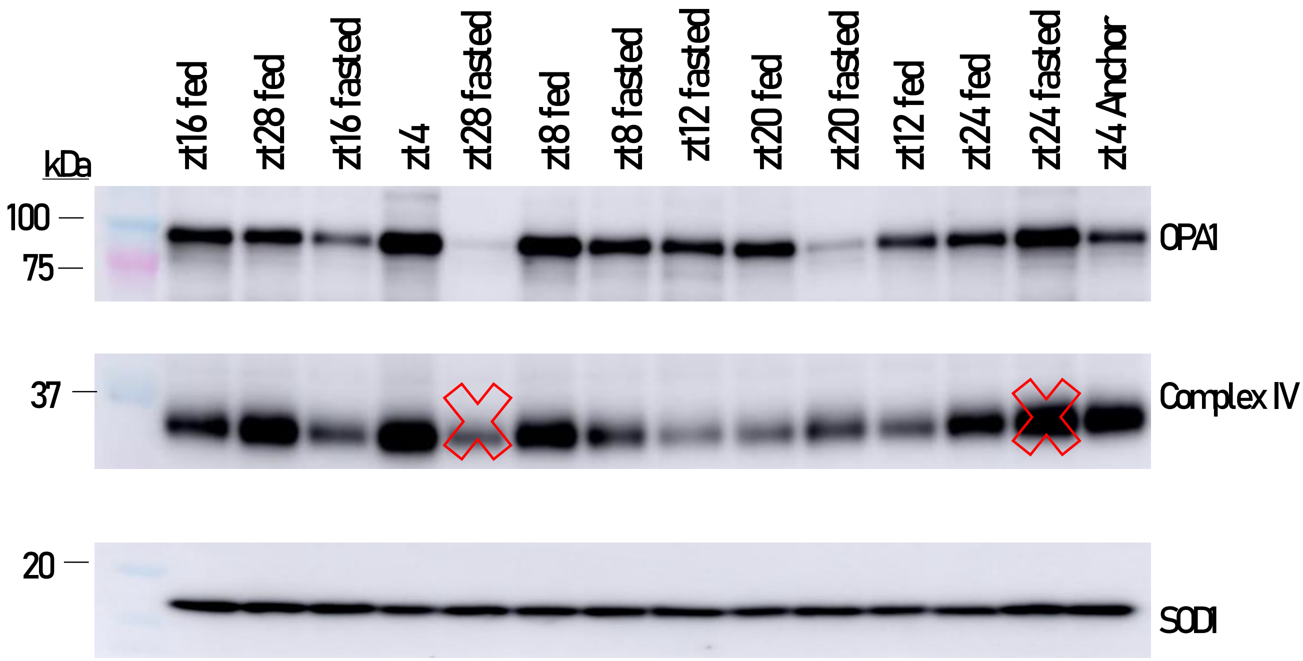

*Complex 4 Zt28 fast excluded, experimental error
*Complex 4 Zt24 fast excluded, 2SD from mean

Gel 4

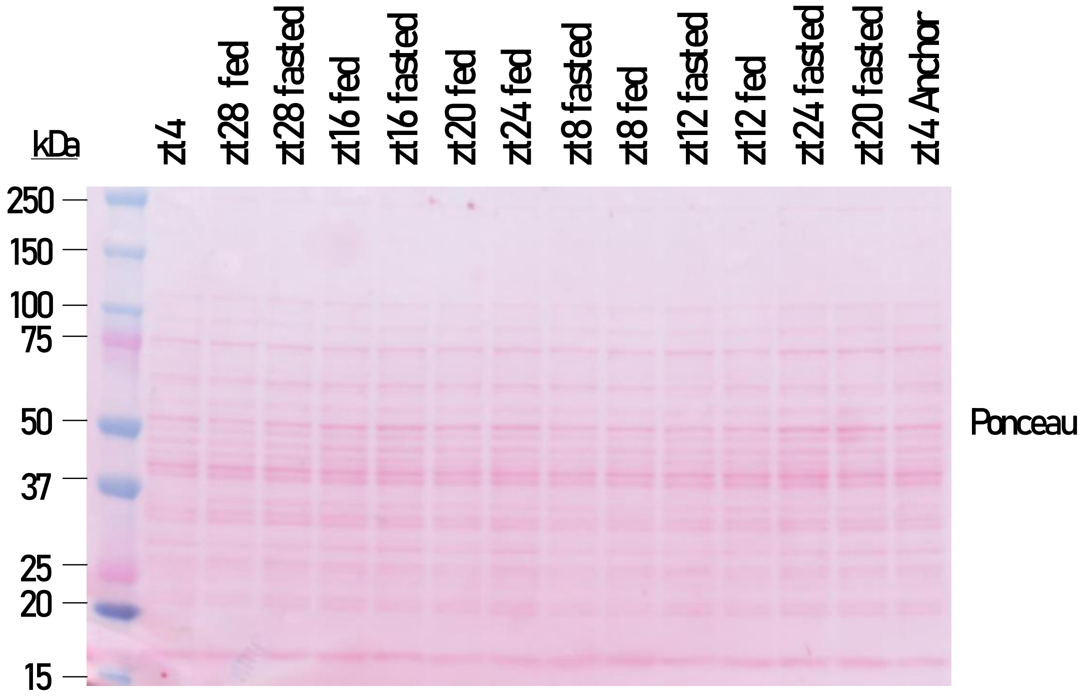

kDa
zt4
zt28 fed
zt28 fasted
zt16 fed
zt16 fasted
zt20 fed
zt24 fed
zt8 fasted
zt8 fed
zt12 fasted
zt12 fed
zt24 fasted
zt20 fasted
zt4 Anchor
250
150
100
75
50
37
25
20
15
Ponceau

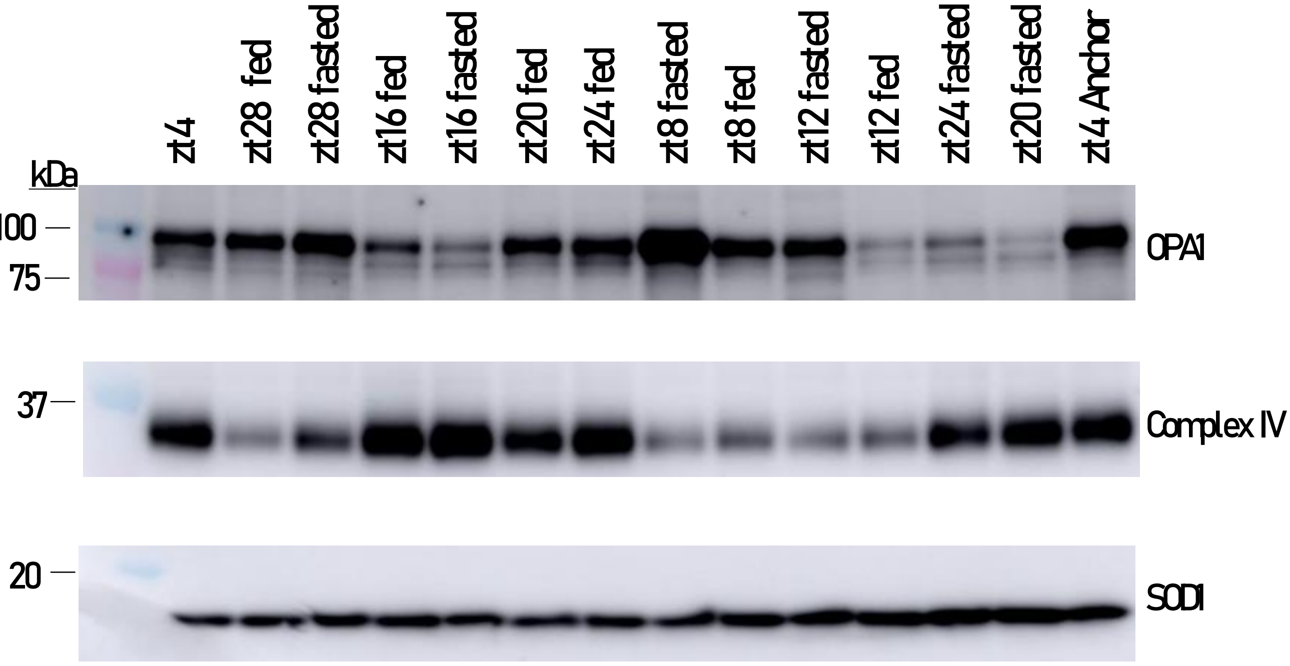

kDa
zt4
zt28 fed
zt28 fasted
zt16 fed
zt16 fasted
zt20 fed
zt24 fed
zt8 fasted
zt8 fed
zt12 fasted
zt12 fed
zt24 fasted
zt20 fasted
zt4 Anchor
100
75
OPA1
37
Complex IV
20
SOD1

Gel 5

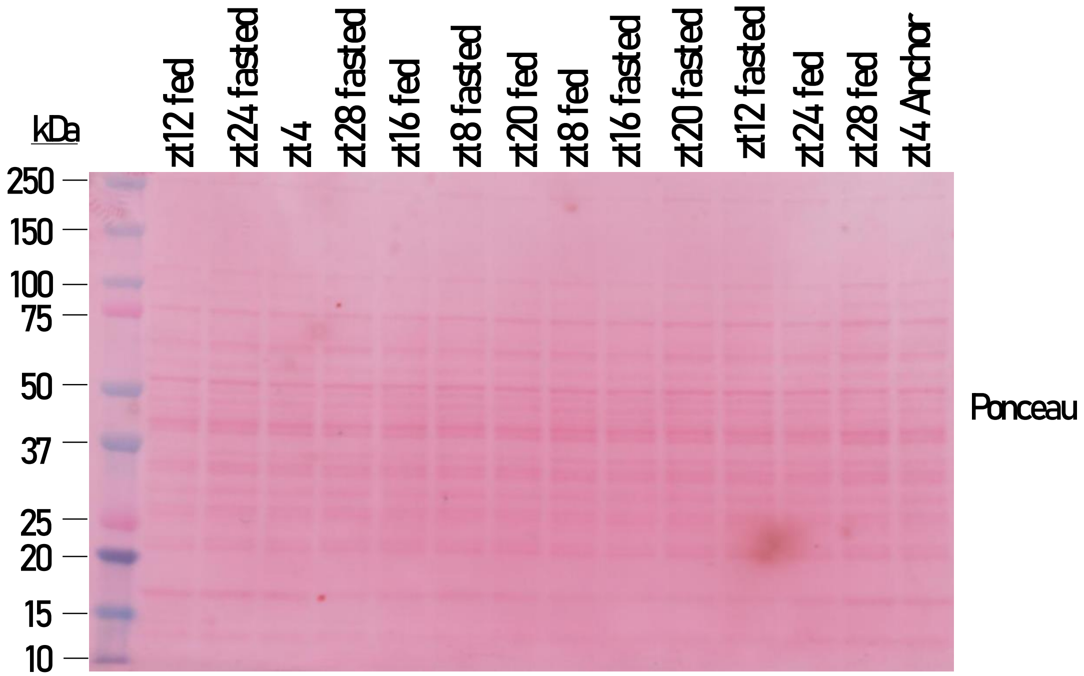
kDa
250
150
100
75
50
37
25
20
15
10
zt12 fed
zt24 fasted
zt4
zt28 fasted
zt16 fed
zt8 fasted
zt20 fed
zt8 fed
zt16 fasted
zt20 fasted
zt12 fasted
zt24 fed
zt28 fed
zt4 Anchor
Ponceau

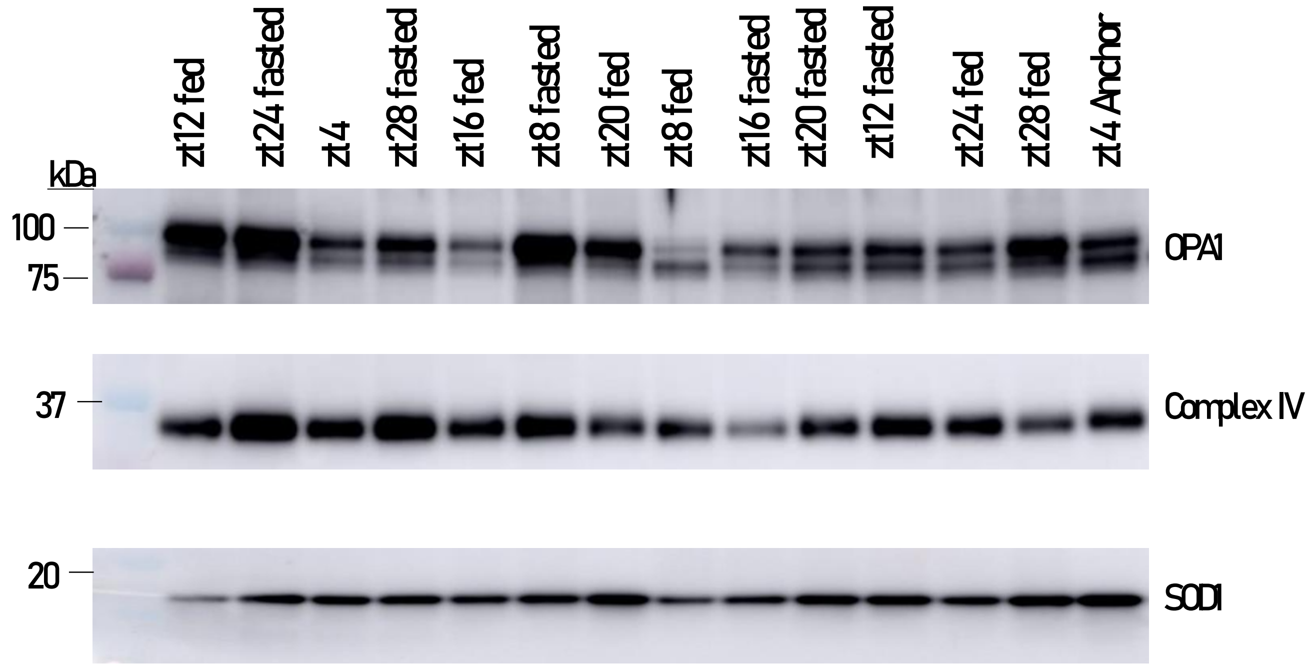
kDa
100
75
37
20
zt12 fed
zt24 fasted
zt4
zt28 fasted
zt16 fed
zt8 fasted
zt20 fed
zt8 fed
zt16 fasted
zt20 fasted
zt12 fasted
zt24 fed
zt28 fed
zt4 Anchor
OPA1
Complex IV
SOD1

Gel 6

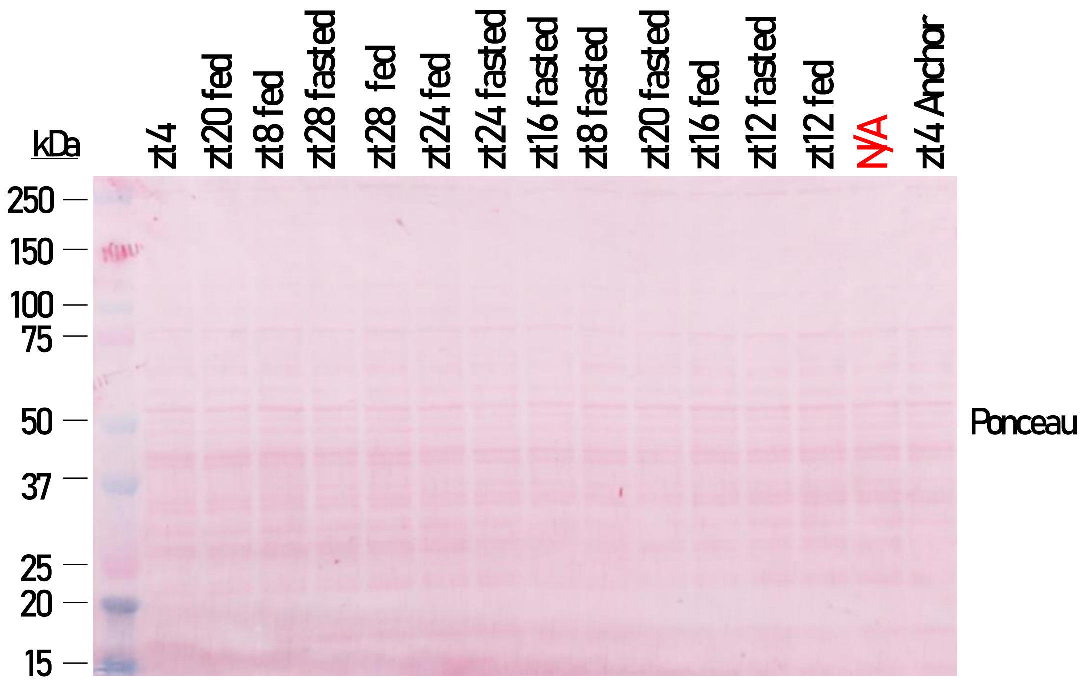

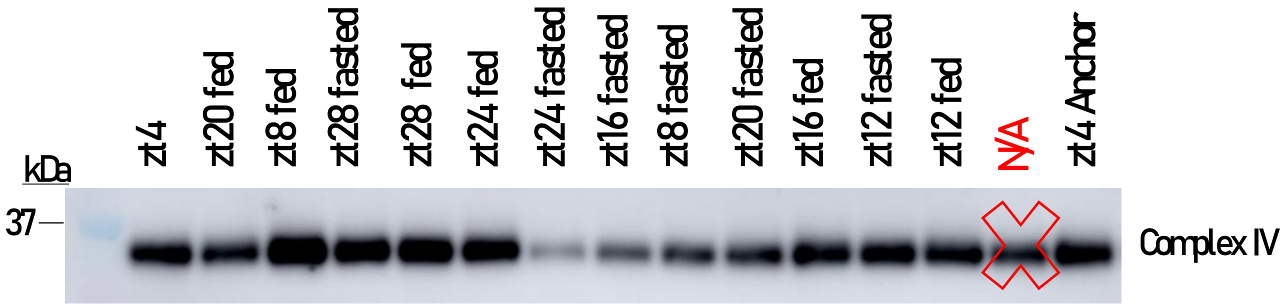

*N/A, wrong sample loaded

Gel 6b

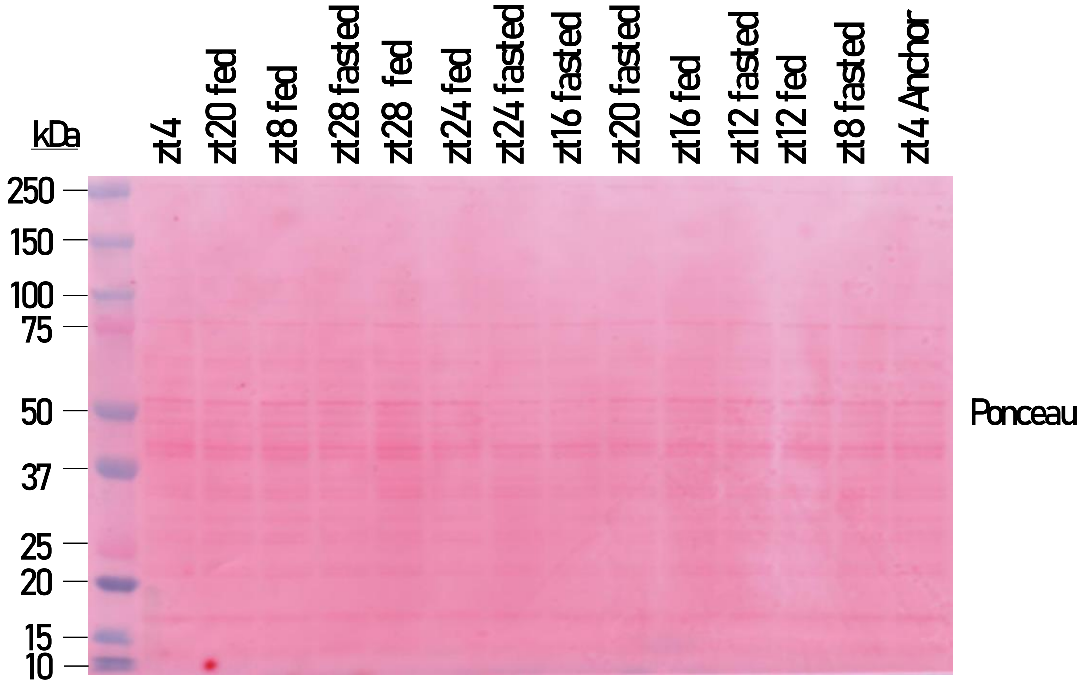

kDa
zt4
zt20 fed
zt8 fed
zt28 fasted
zt28 fed
zt24 fed
zt24 fasted
zt16 fasted
zt20 fasted
zt16 fed
zt12 fasted
zt12 fed
zt8 fasted
zt4 Anchor
250
150
100
75
50
37
25
20
15
10
Ponceau

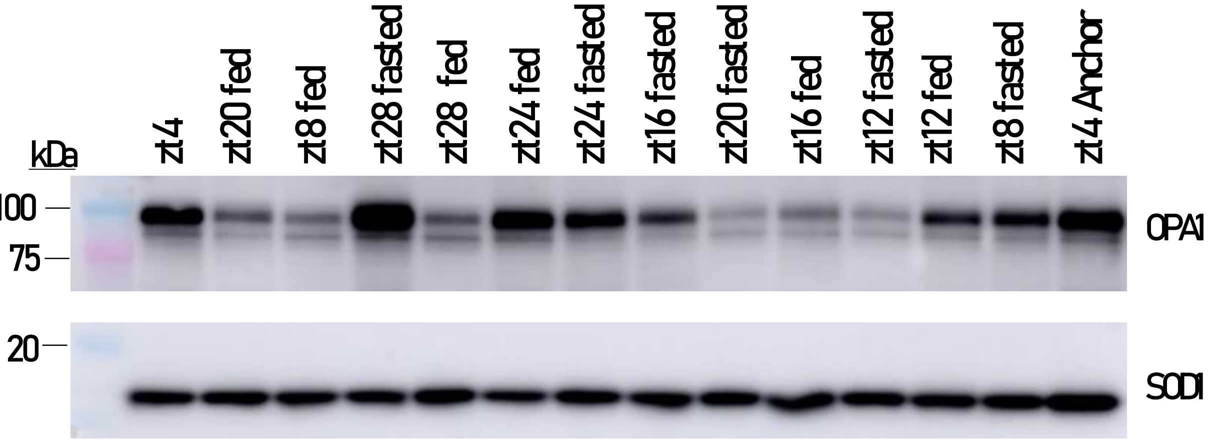

kDa
zt4
zt20 fed
zt8 fed
zt28 fasted
zt28 fed
zt24 fed
zt24 fasted
zt16 fasted
zt20 fasted
zt16 fed
zt12 fasted
zt12 fed
zt8 fasted
zt4 Anchor
100
75
OPA1
20
SOD1

Gel 7

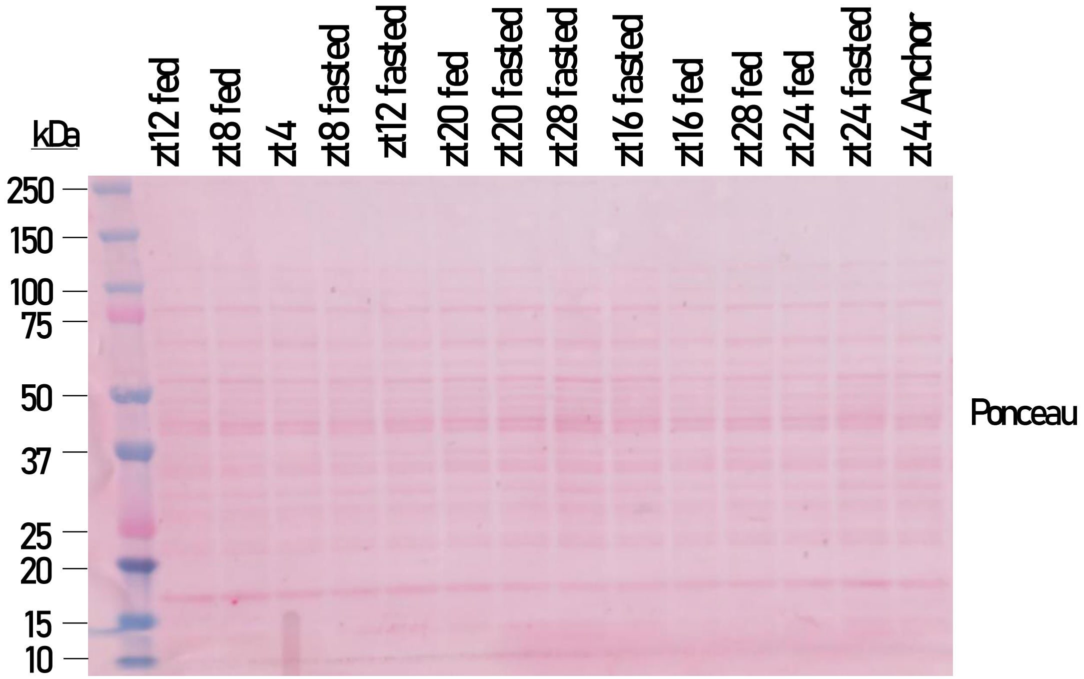
kDa
250
150
100
75
50
37
25
20
15
10
zt12 fed
zt8 fed
zt4
zt8 fasted
zt12 fasted
zt20 fed
zt20 fasted
zt28 fasted
zt16 fasted
zt16 fed
zt28 fed
zt24 fed
zt24 fasted
zt4 Anchor
Ponceau

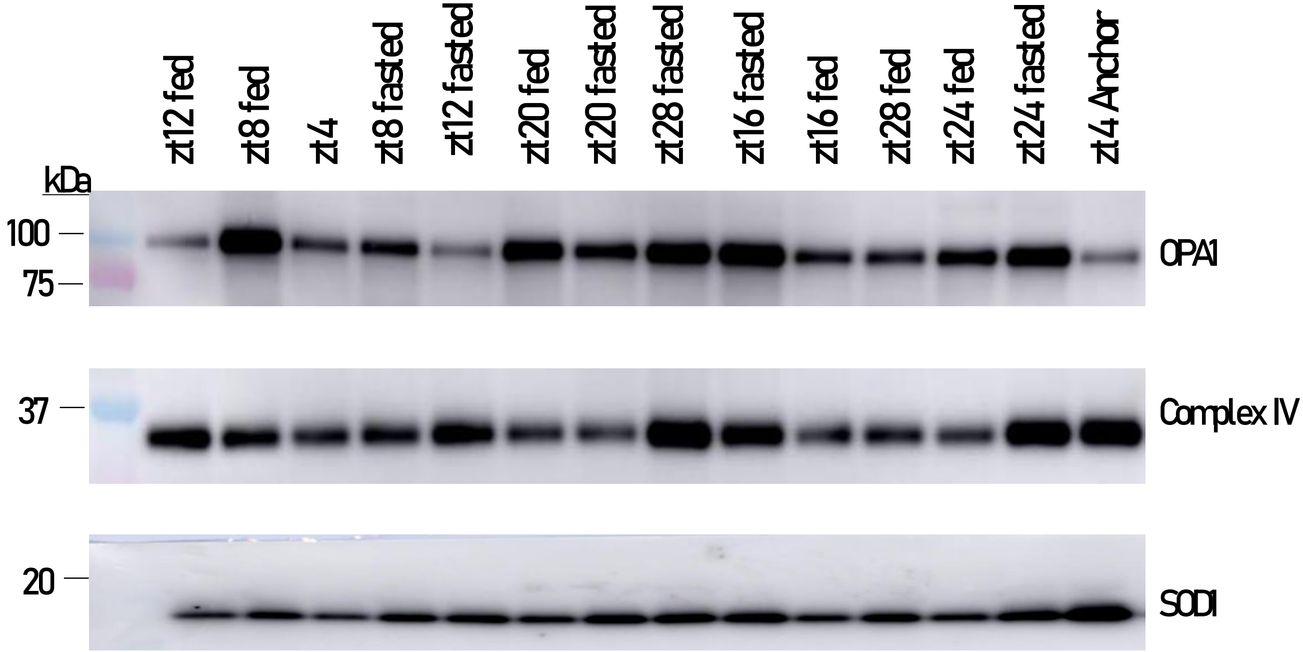
kDa
100
75
37
20
zt12 fed
zt8 fed
zt4
zt8 fasted
zt12 fasted
zt20 fed
zt20 fasted
zt28 fasted
zt16 fasted
zt16 fed
zt28 fed
zt24 fed
zt24 fasted
zt4 Anchor
OPA1
Complex IV
SOD1

Gel 8

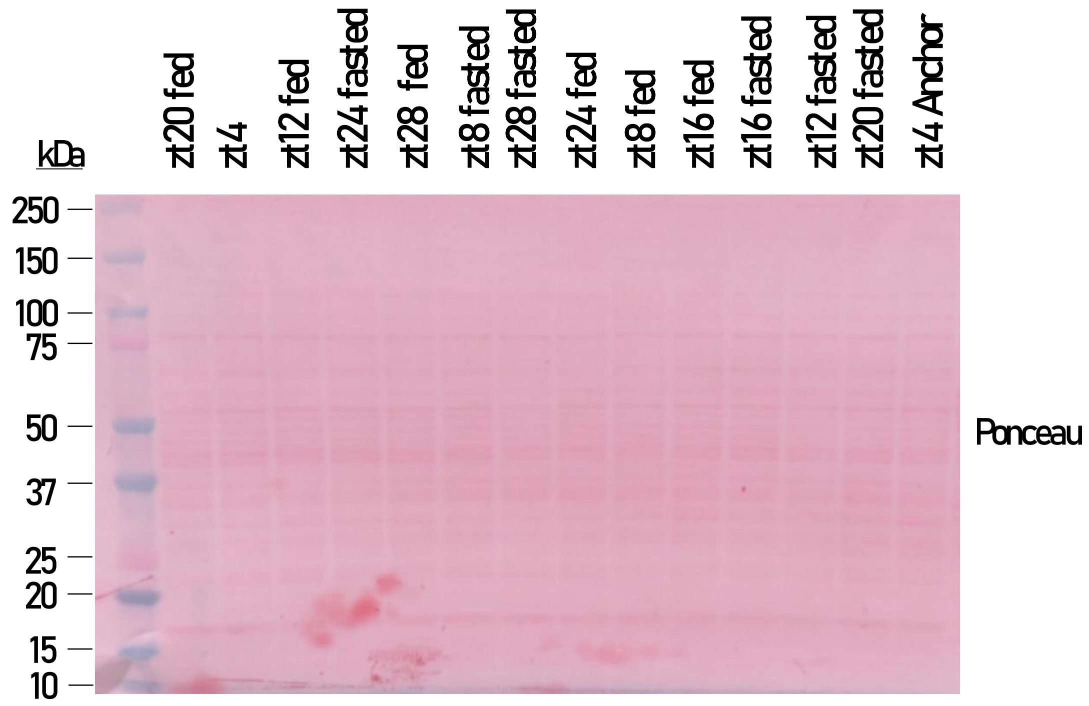
kDa
250
150
100
75
50
37
25
20
15
10
zt20 fed
zt4
zt12 fed
zt24 fasted
zt28 fed
zt8 fasted
zt28 fasted
zt24 fed
zt8 fed
zt16 fed
zt16 fasted
zt12 fasted
zt20 fasted
zt4 Anchor
Ponceau

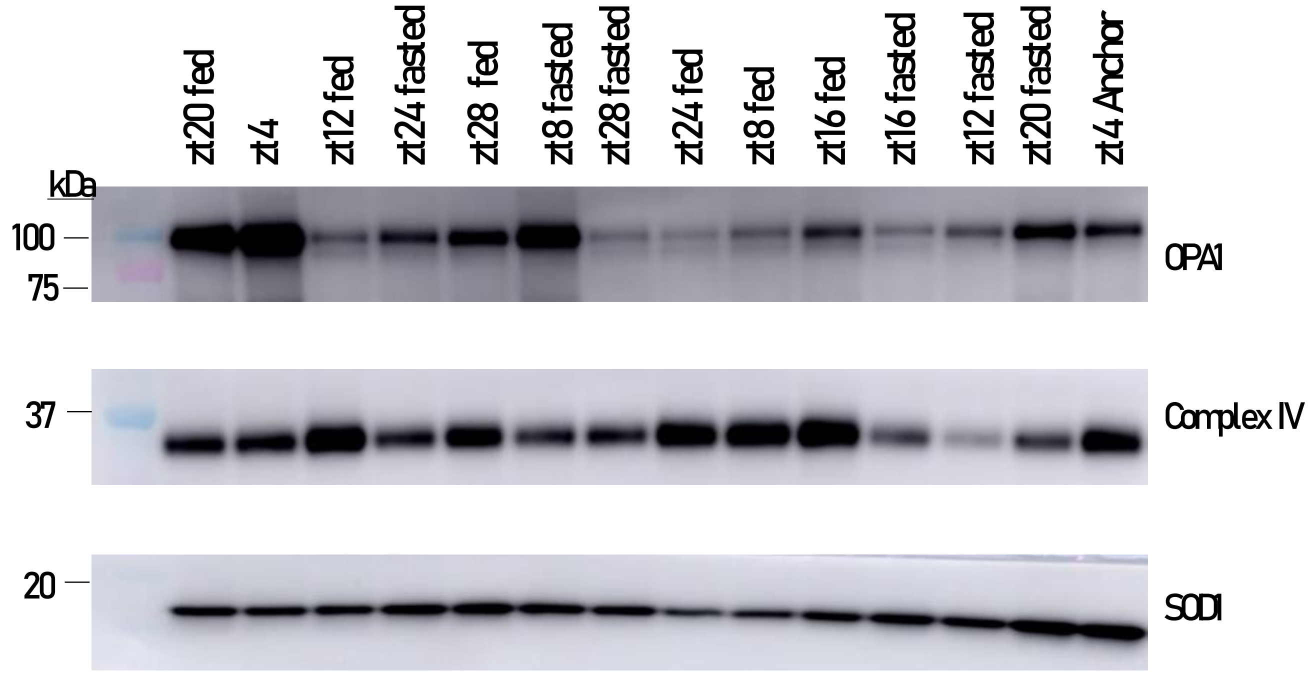
zt20 fed
zt4
zt12 fed
zt24 fasted
zt28 fed
zt8 fasted
zt28 fasted
zt24 fed
zt8 fed
zt16 fed
zt16 fasted
zt12 fasted
zt20 fasted
zt4 Anchor
kDa
100
75
OPA1
37
Complex IV
20
SOD1

Gel 9

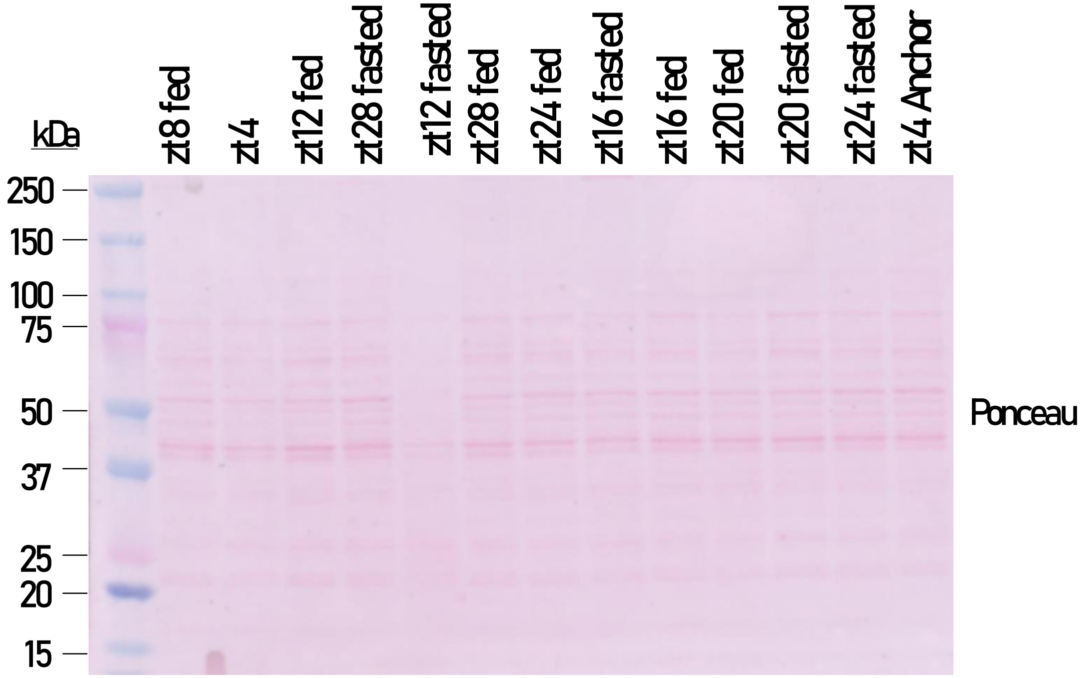
kDa
250
150
100
75
50
37
25
20
15
zt8 fed
zt4
zt12 fed
zt28 fasted
zt12 fasted
zt28 fed
zt24 fed
zt16 fasted
zt16 fed
zt20 fed
zt20 fasted
zt24 fasted
zt4 Anchor
Ponceau

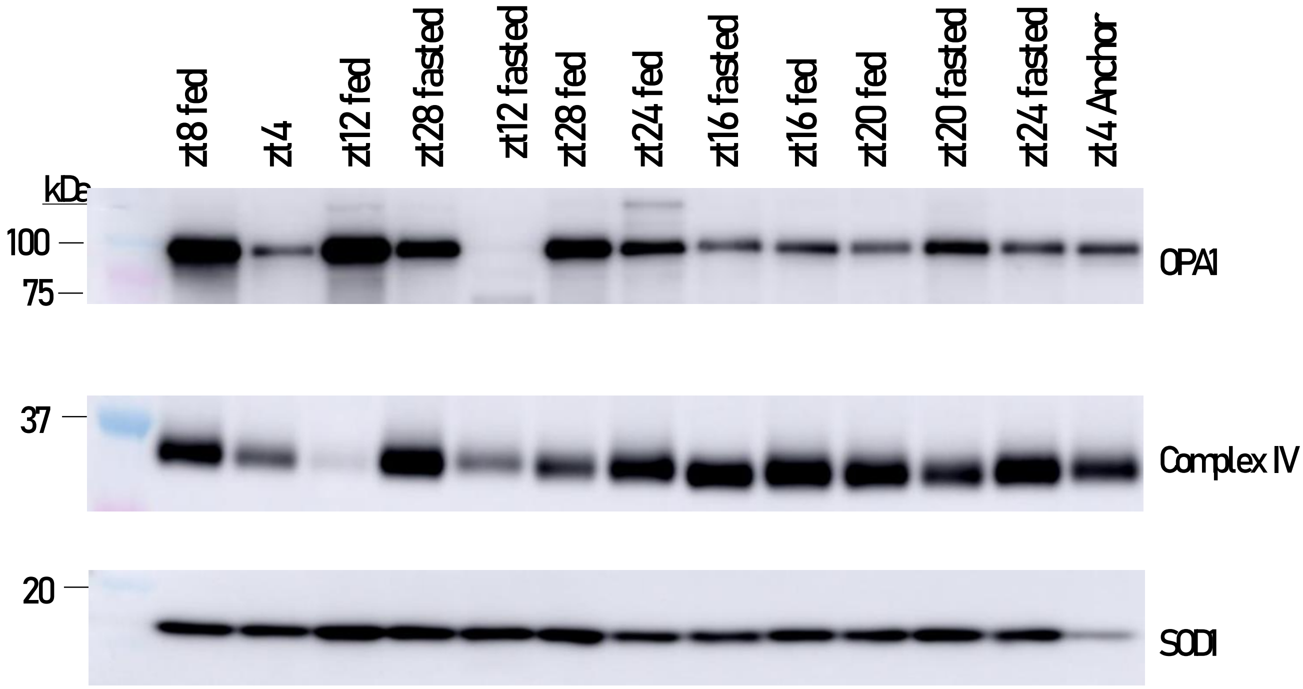
zt8 fed
zt4
zt12 fed
zt28 fasted
zt12 fasted
zt28 fed
zt24 fed
zt16 fasted
zt16 fed
zt20 fed
zt20 fasted
zt24 fasted
zt4 Anchor
kDa
100
75
OPA1
37
Complex IV
20
SOD1

Gel 10

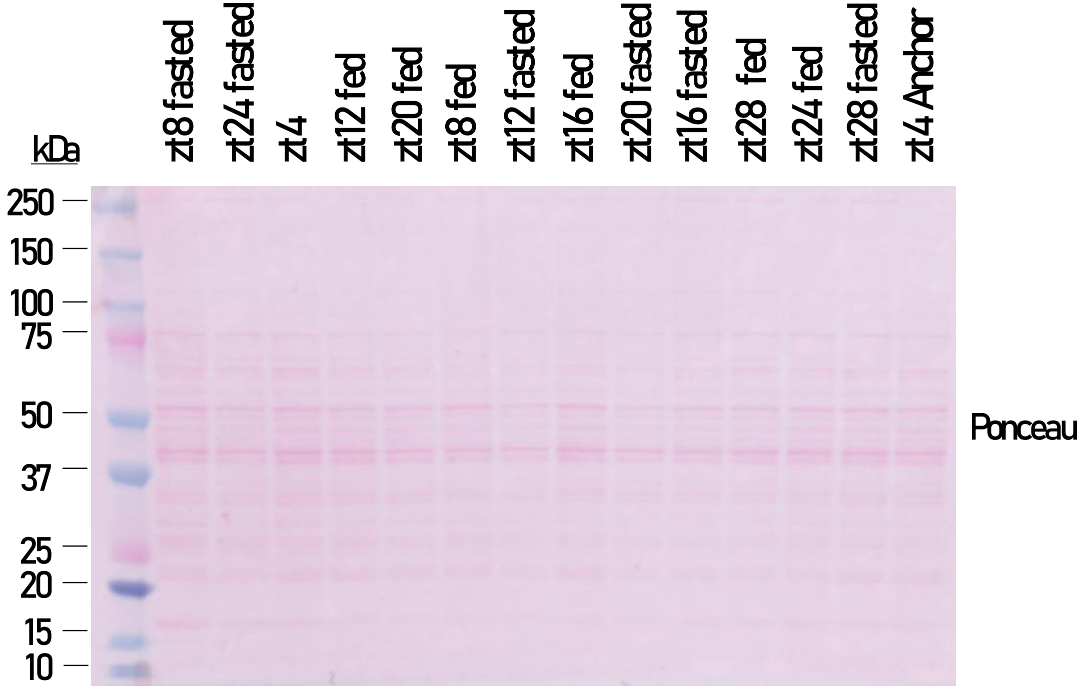
kDa
250
150
100
75
50
37
25
20
15
10
zt8 fasted
zt24 fasted
zt4
zt12 fed
zt20 fed
zt8 fed
zt12 fasted
zt16 fed
zt20 fasted
zt16 fasted
zt28 fed
zt24 fed
zt28 fasted
zt4 Anchor
Ponceau

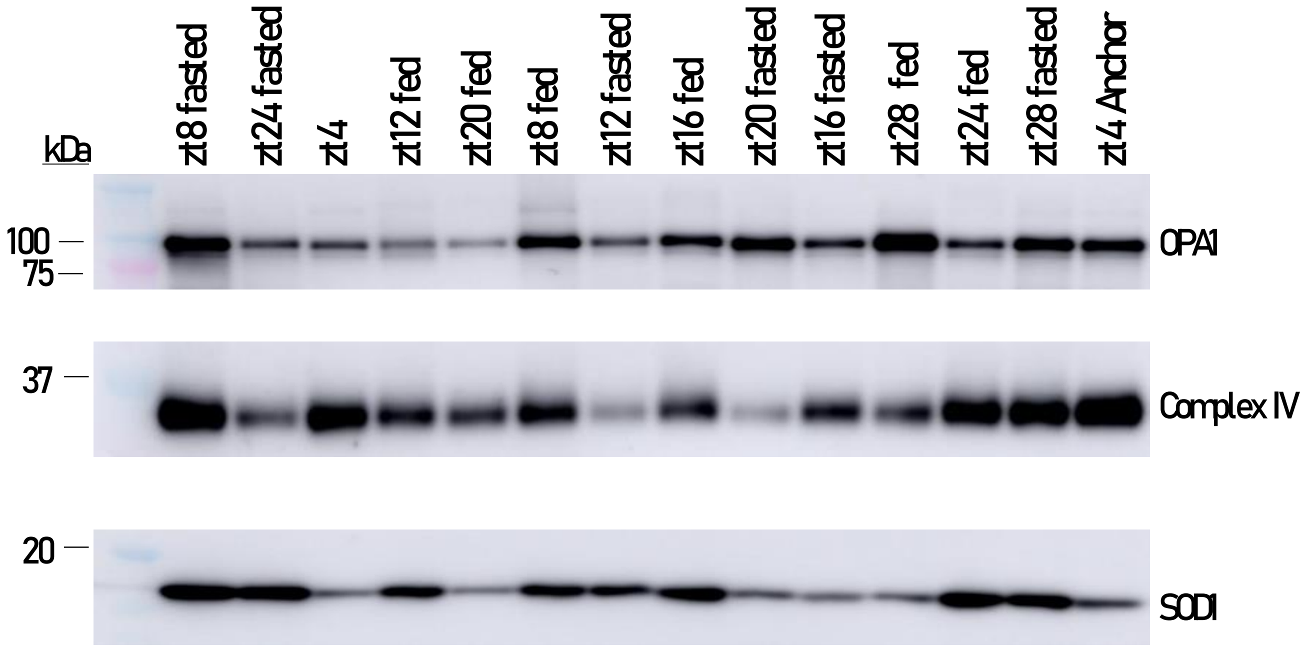
kDa
100
75
37
20
zt8 fasted
zt24 fasted
zt4
zt12 fed
zt20 fed
zt8 fed
zt12 fasted
zt16 fed
zt20 fasted
zt16 fasted
zt28 fed
zt24 fed
zt28 fasted
zt4 Anchor
OPA1
Complex IV
SOD1

Gel 11

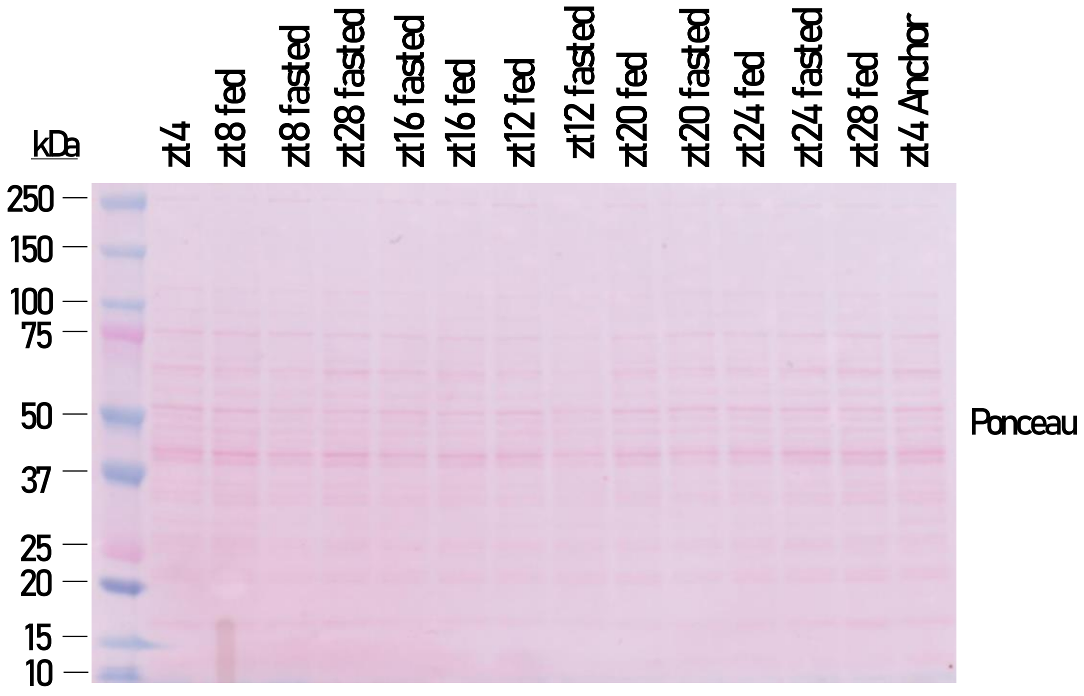

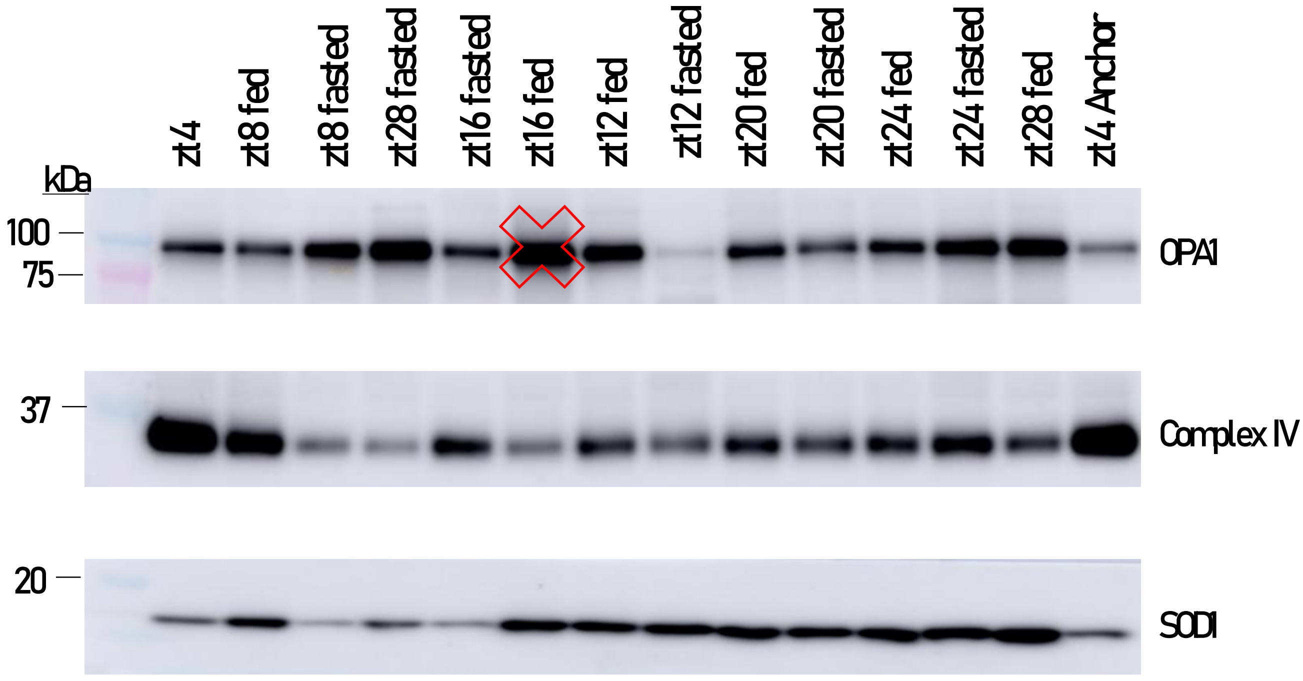

*ZT16 fed excluded, Signal saturating

Gel 12

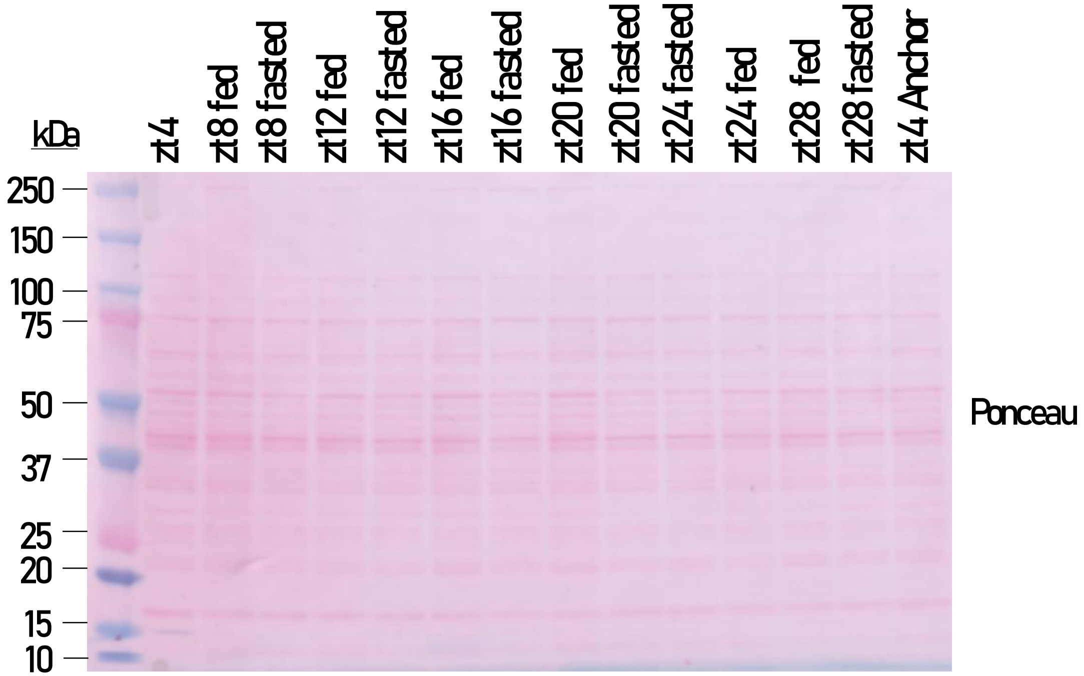

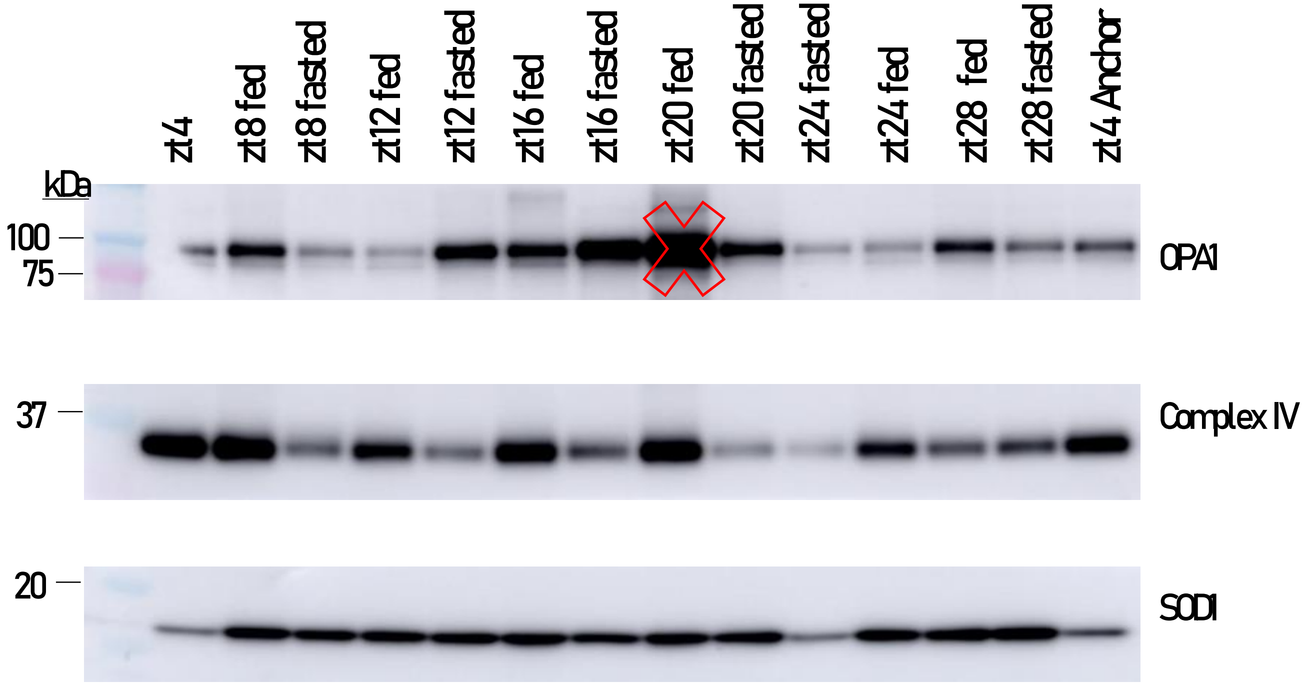

*ZT20 fed excluded, signal saturating

Week 2

CS (Abcam, ab129095), VDAC (Abcam, ab14734)

March 8-12 2021

Gel 1

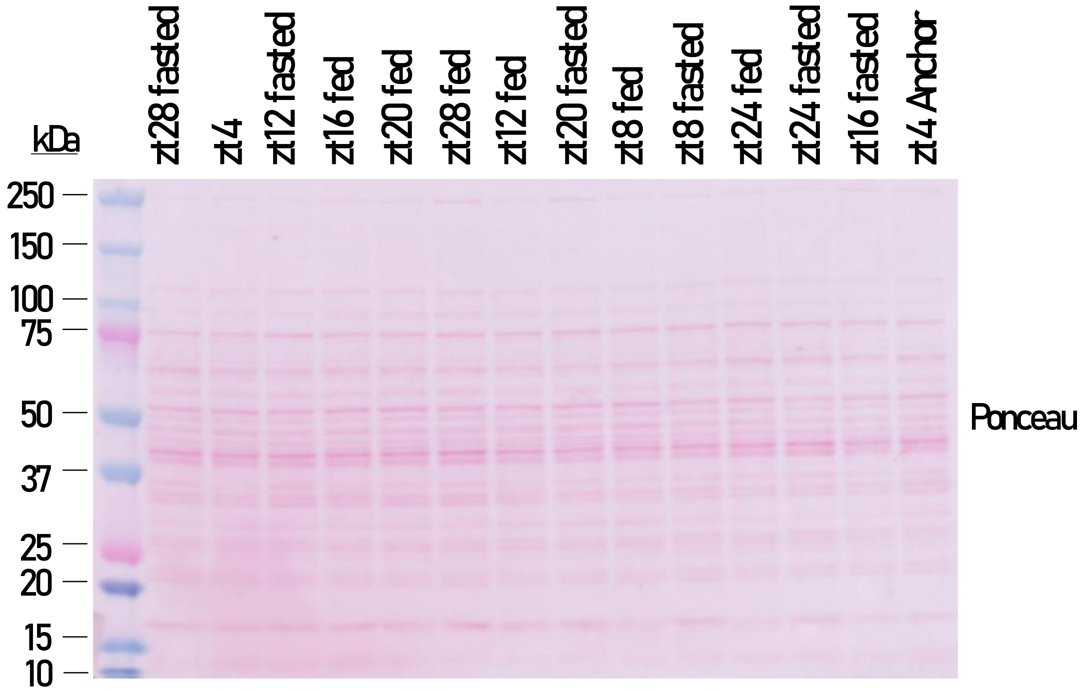
kDa
250
150
100
75
50
37
25
20
15
10
zt28 fasted
zt4
zt12 fasted
zt16 fed
zt20 fed
zt28 fed
zt12 fed
zt20 fasted
zt8 fed
zt8 fasted
zt24 fed
zt24 fasted
zt16 fasted
zt4 Anchor
Ponceau

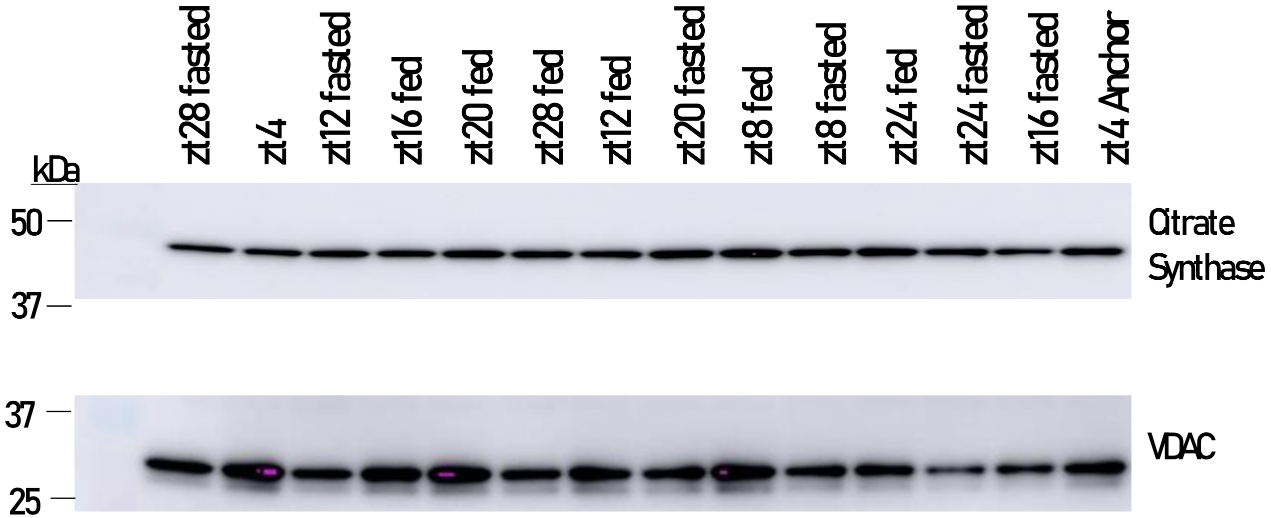
kDa
50
37
37
25
zt28 fasted
zt4
zt12 fasted
zt16 fed
zt20 fed
zt28 fed
zt12 fed
zt20 fasted
zt8 fed
zt8 fasted
zt24 fed
zt24 fasted
zt16 fasted
zt4 Anchor
Citrate Synthase
VDAC

Gel 2

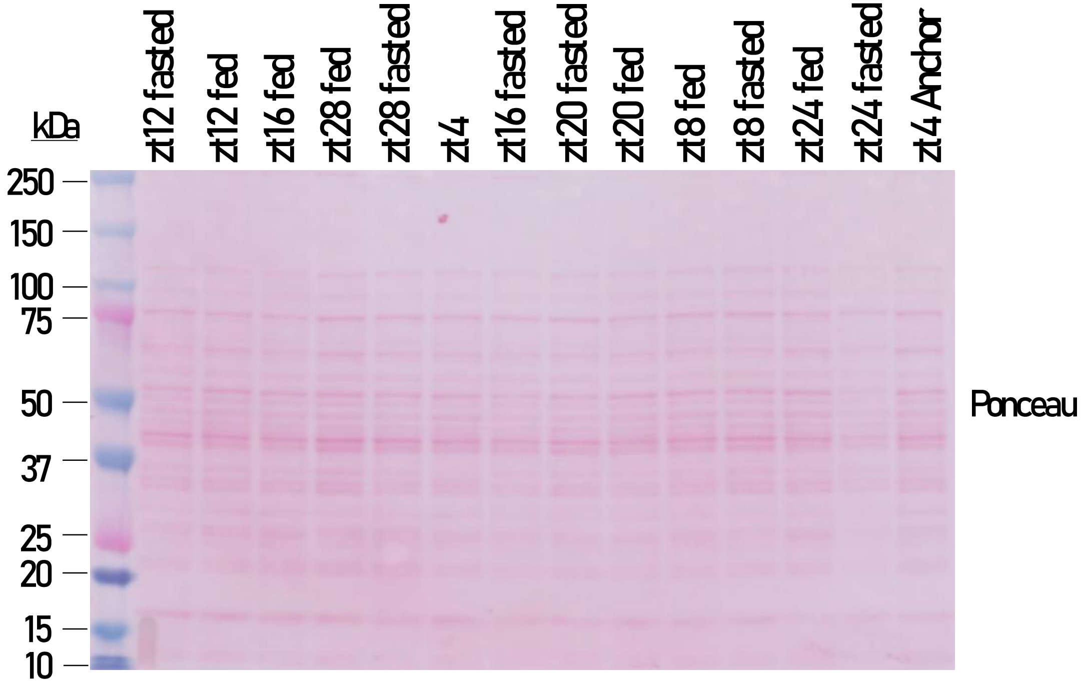
kDa
zt12 fasted
zt12 fed
zt16 fed
zt28 fed
zt28 fasted
zt4
zt16 fasted
zt20 fasted
zt20 fed
zt8 fed
zt8 fasted
zt24 fed
zt24 fasted
zt4 Anchor
250
150
100
75
50
37
25
20
15
10
Ponceau

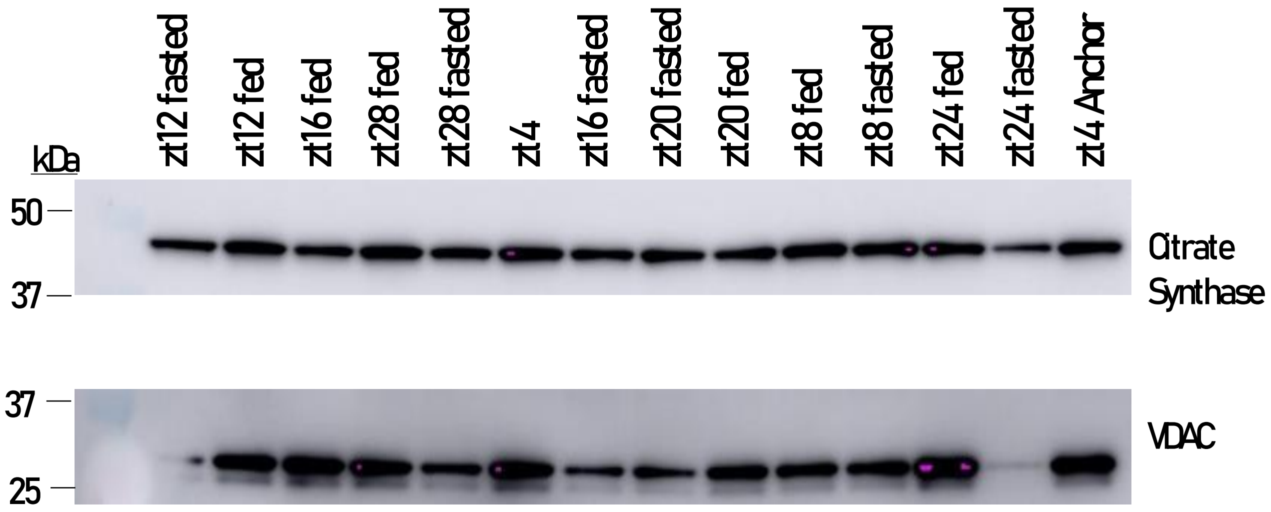
kDa
zt12 fasted
zt12 fed
zt16 fed
zt28 fed
zt28 fasted
zt4
zt16 fasted
zt20 fasted
zt20 fed
zt8 fed
zt8 fasted
zt24 fed
zt24 fasted
zt4 Anchor
50
37
Citrate Synthase
37
25
VDAC

Gel 3

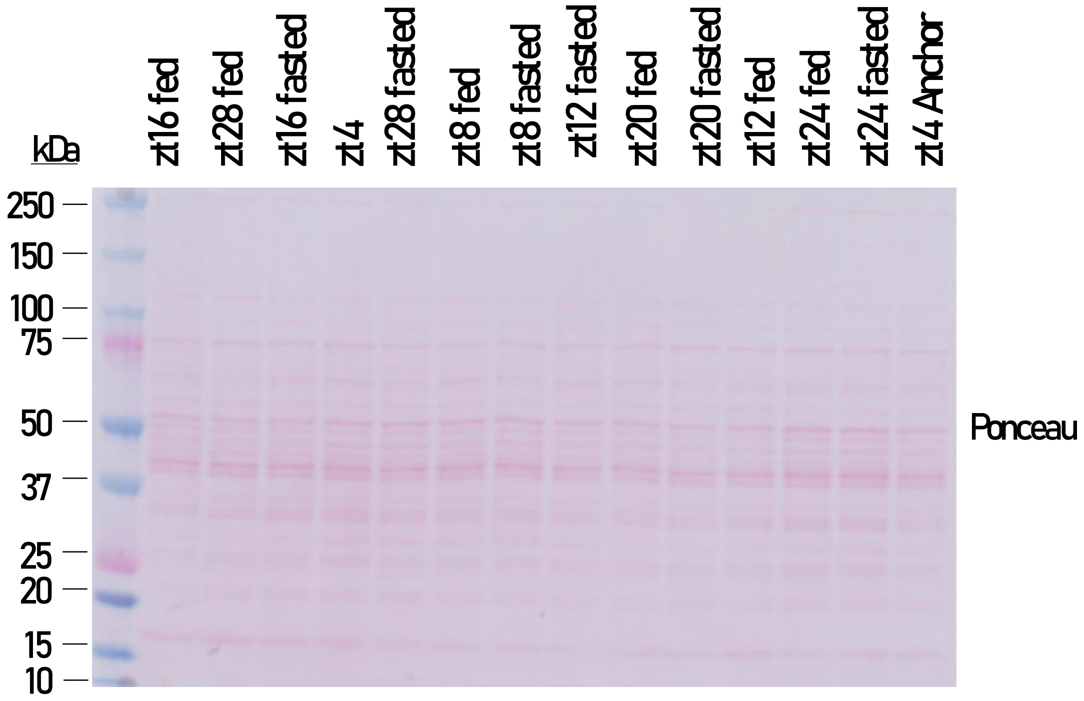

kDa
zt16 fed
zt28 fed
zt16 fasted
zt4
zt28 fasted
zt8 fed
zt8 fasted
zt12 fasted
zt20 fed
zt20 fasted
zt12 fed
zt24 fed
zt24 fasted
zt4 Anchor
250
150
100
75
50
37
25
20
15
10
Ponceau

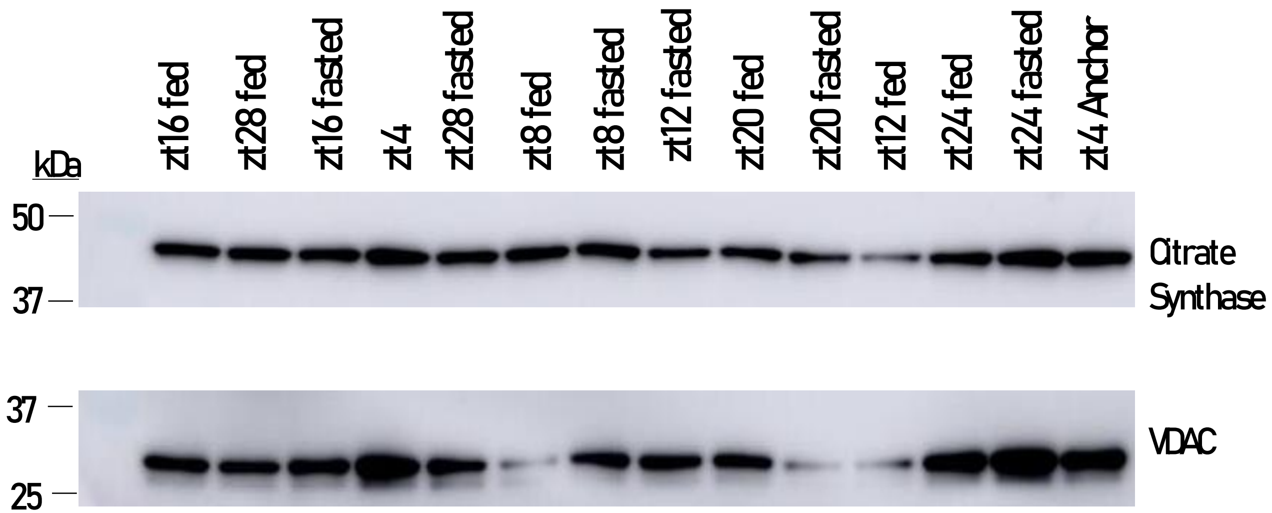

kDa
zt16 fed
zt28 fed
zt16 fasted
zt4
zt28 fasted
zt8 fed
zt8 fasted
zt12 fasted
zt20 fed
zt20 fasted
zt12 fed
zt24 fed
zt24 fasted
zt4 Anchor
50
37
Citrate Synthase
37
25
VDAC

Gel 4

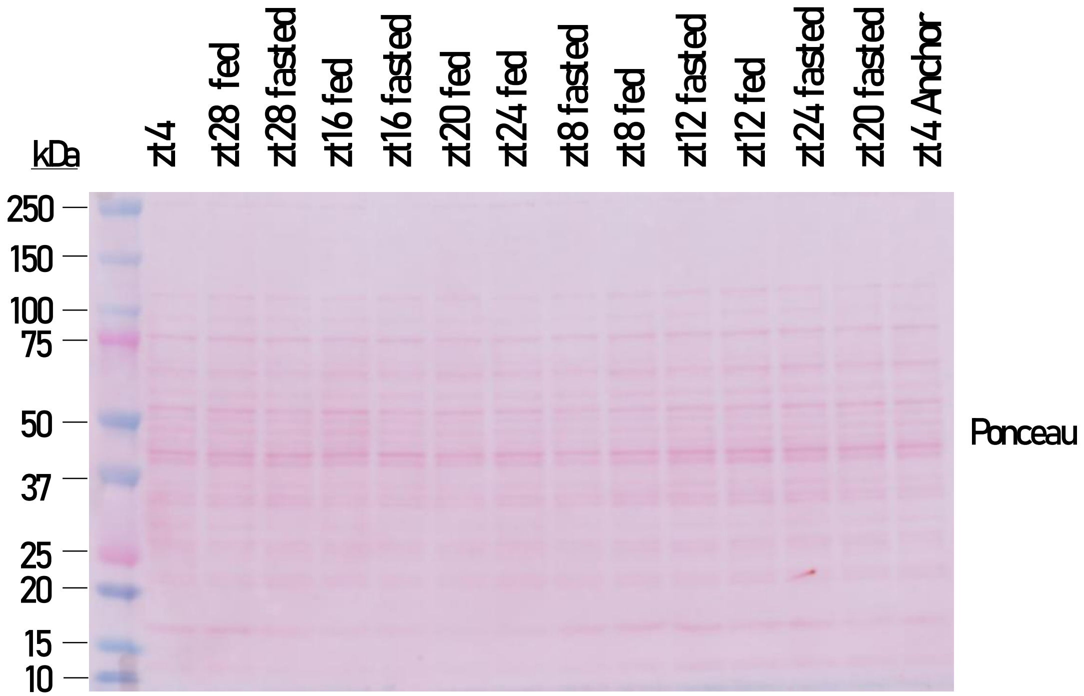
zt4
zt28 fed
zt28 fasted
zt16 fed
zt16 fasted
zt20 fed
zt24 fed
zt8 fasted
zt8 fed
zt12 fasted
zt12 fed
zt24 fasted
zt20 fasted
zt4 Anchor
kDa
250
150
100
75
50
37
25
20
15
10
Ponceau

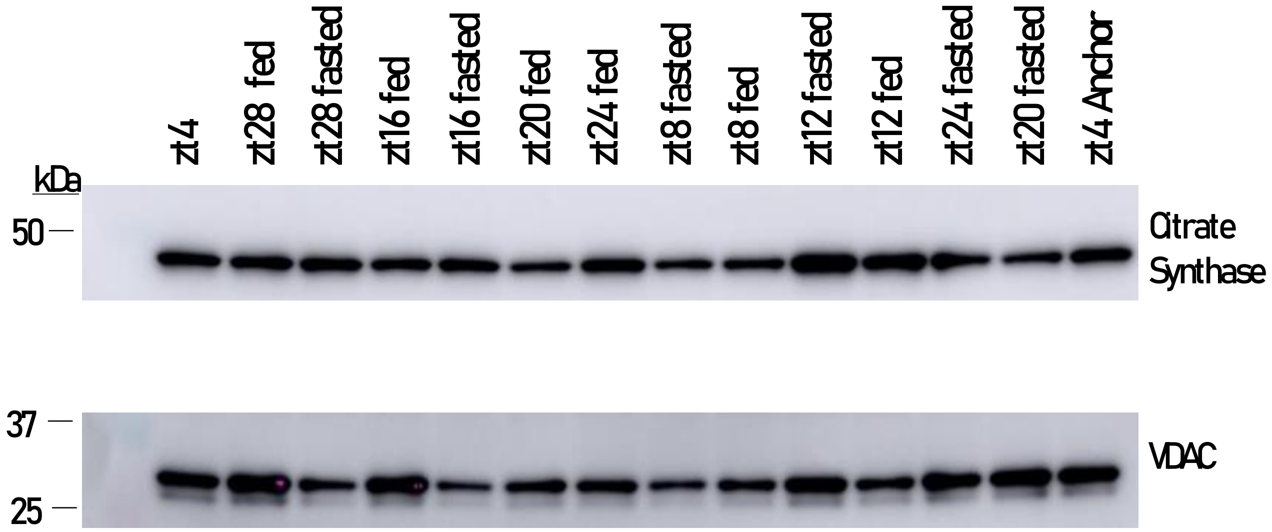
zt4
zt28 fed
zt28 fasted
zt16 fed
zt16 fasted
zt20 fed
zt24 fed
zt8 fasted
zt8 fed
zt12 fasted
zt12 fed
zt24 fasted
zt20 fasted
zt4 Anchor
kDa
50
Citrate Synthase
37
25
VDAC

Gel 5

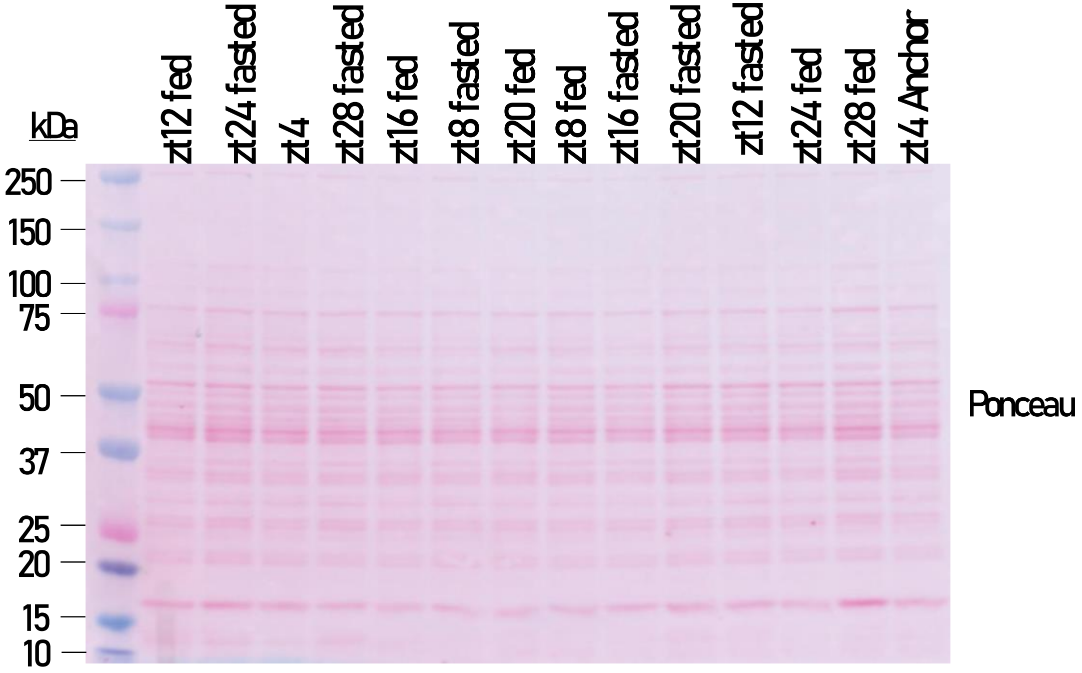

kDa
250
150
100
75
50
37
25
20
15
10
zt12 fed
zt24 fasted
zt4
zt28 fasted
zt16 fed
zt8 fasted
zt20 fed
zt8 fed
zt16 fasted
zt20 fasted
zt12 fasted
zt24 fed
zt28 fed
zt4 Anchor
Ponceau

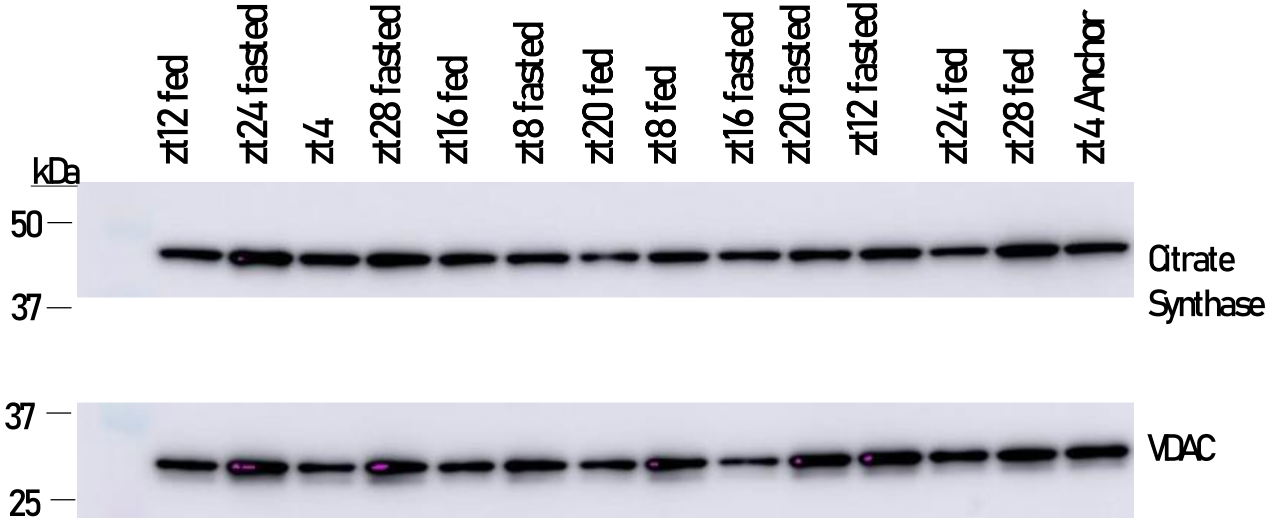

kDa
50
37
37
25
zt12 fed
zt24 fasted
zt4
zt28 fasted
zt16 fed
zt8 fasted
zt20 fed
zt8 fed
zt16 fasted
zt20 fasted
zt12 fasted
zt24 fed
zt28 fed
zt4 Anchor
Citrate Synthase
VDAC

Gel 6

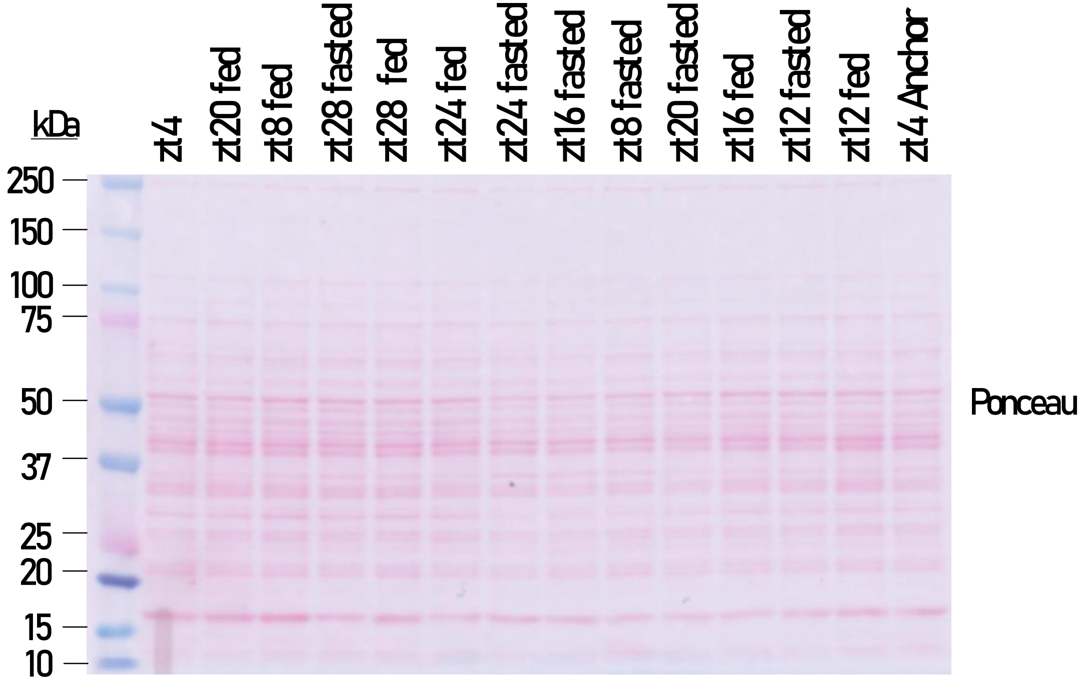

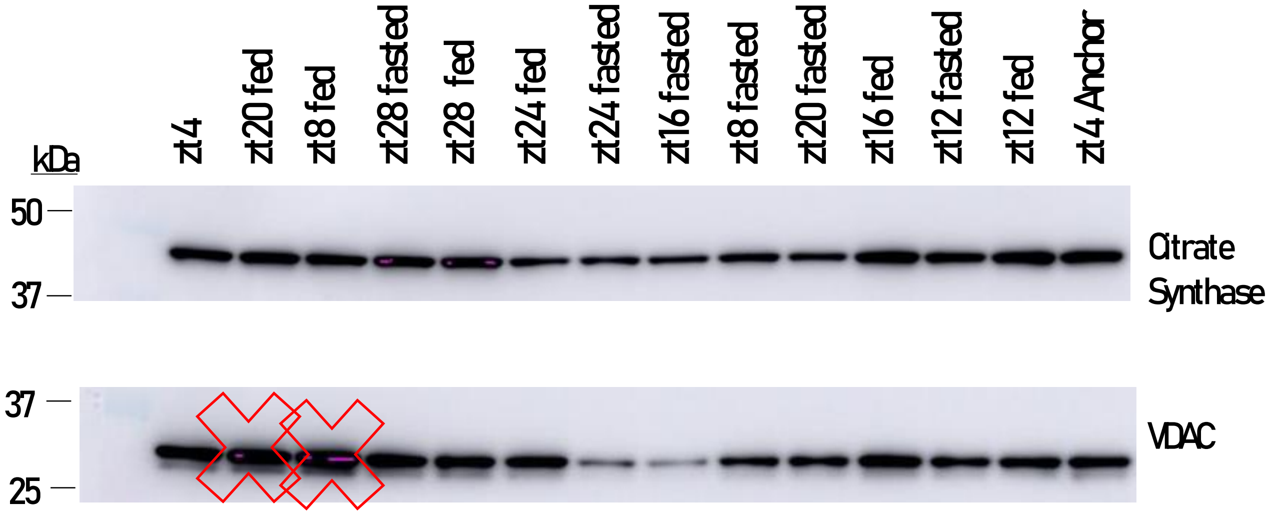

*ZT8 fed excluded from analysis, signal saturated & >2SD from mean

*ZT20 fed signal saturated

Gel 7

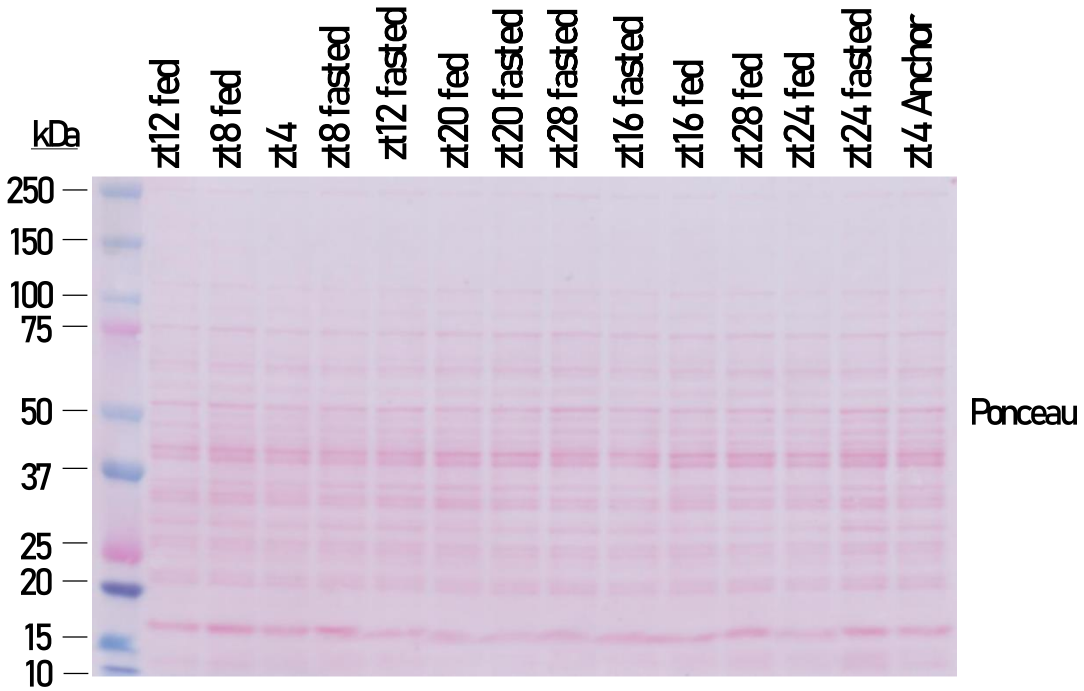
kDa
zt12 fed
zt8 fed
zt4
zt8 fasted
zt12 fasted
zt20 fed
zt20 fasted
zt28 fasted
zt16 fasted
zt16 fed
zt28 fed
zt24 fed
zt24 fasted
zt4 Anchor
250
150
100
75
50
37
25
20
15
10
Ponceau

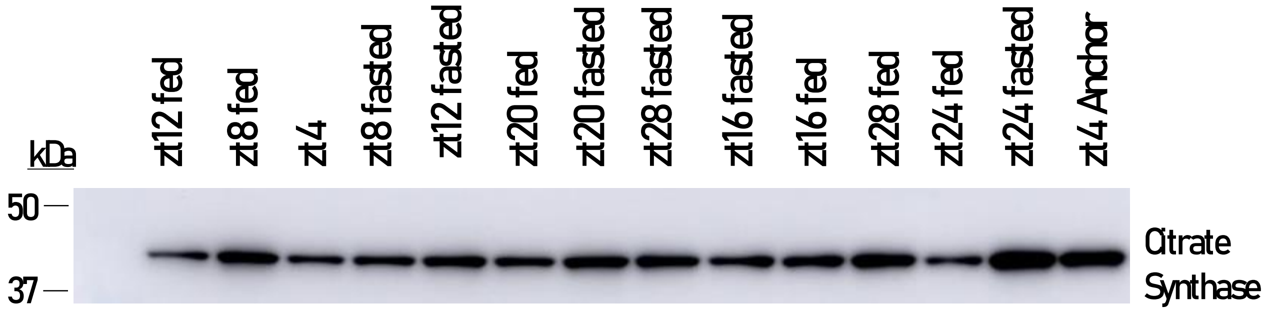
kDa
zt12 fed
zt8 fed
zt4
zt8 fasted
zt12 fasted
zt20 fed
zt20 fasted
zt28 fasted
zt16 fasted
zt16 fed
zt28 fed
zt24 fed
zt24 fasted
zt4 Anchor
50
37
Citrate
Synthase

Gel 7

VDAC rerun

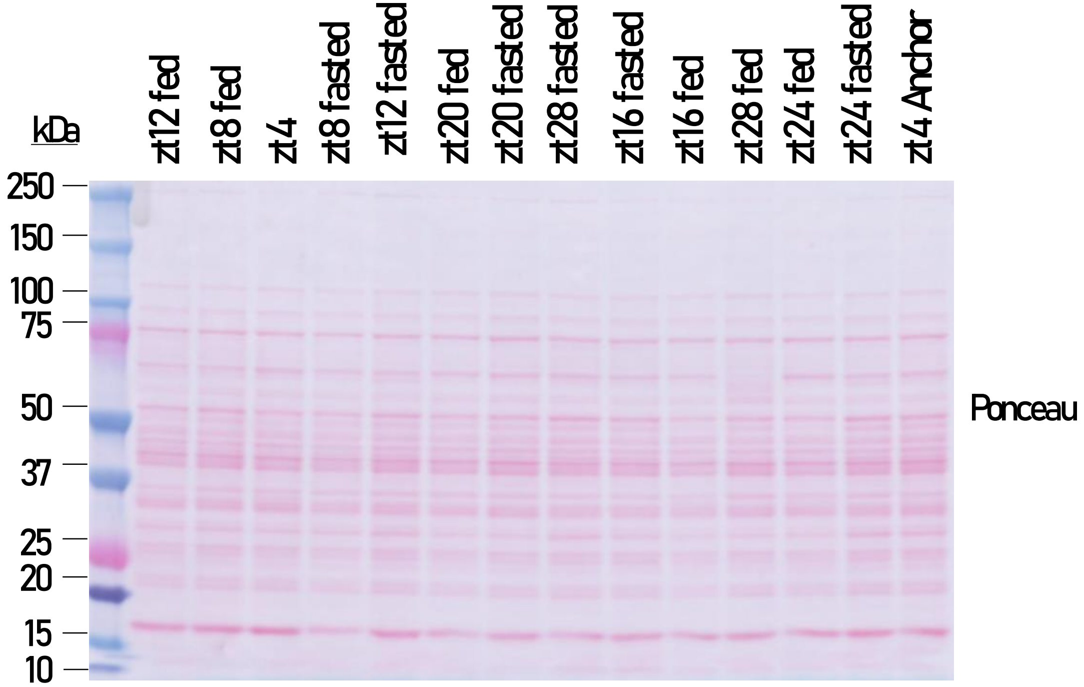

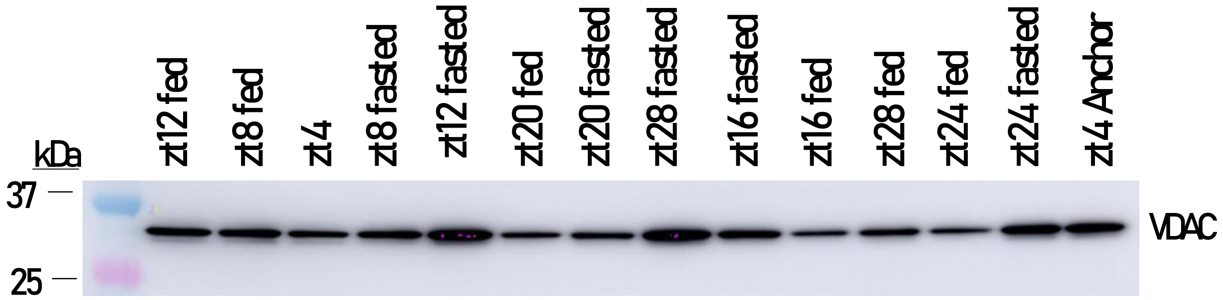

Gel 8

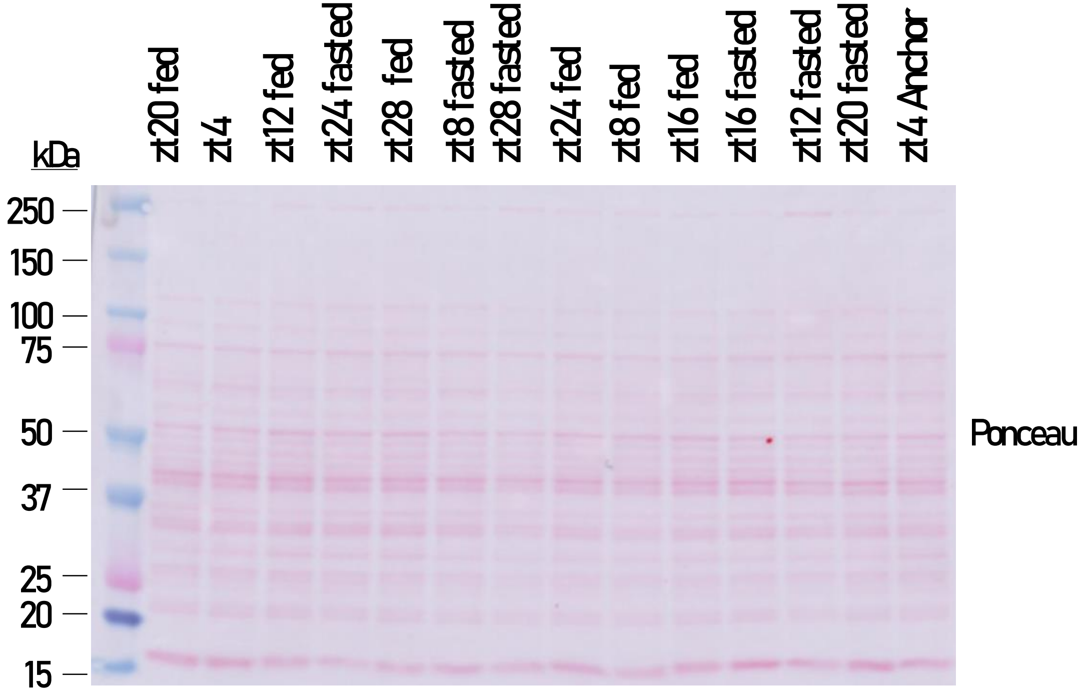

kDa
zt20 fed
zt4
zt12 fed
zt24 fasted
zt28 fed
zt8 fasted
zt28 fasted
zt24 fed
zt8 fed
zt16 fed
zt16 fasted
zt12 fasted
zt20 fasted
zt4 Anchor
250
150
100
75
50
37
25
20
15
Ponceau

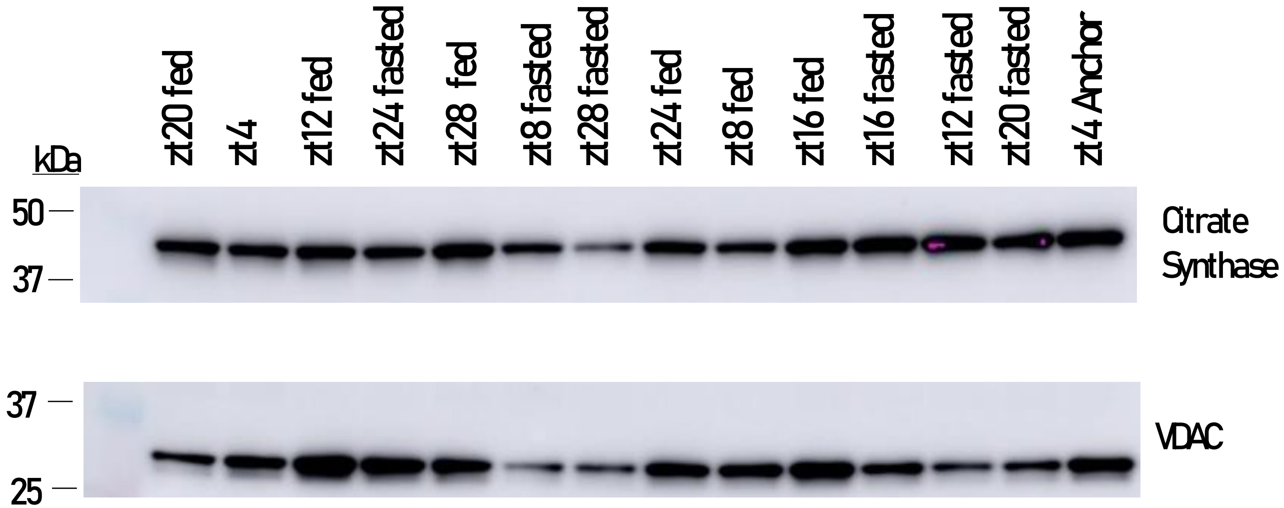

kDa
zt20 fed
zt4
zt12 fed
zt24 fasted
zt28 fed
zt8 fasted
zt28 fasted
zt24 fed
zt8 fed
zt16 fed
zt16 fasted
zt12 fasted
zt20 fasted
zt4 Anchor
50
37
Citrate Synthase
37
25
VDAC

Gel 9

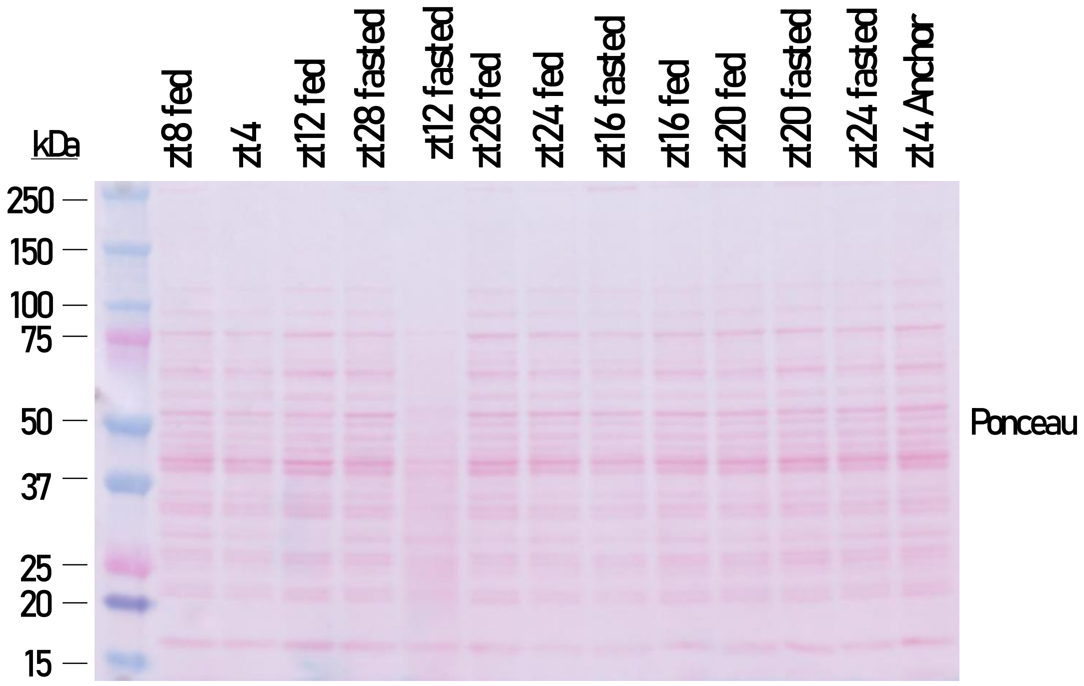
kDa
250
150
100
75
50
37
25
20
15
zt8 fed
zt4
zt12 fed
zt28 fasted
zt12 fasted
zt28 fed
zt24 fed
zt16 fasted
zt16 fed
zt20 fed
zt20 fasted
zt24 fasted
zt4 Anchor
Ponceau

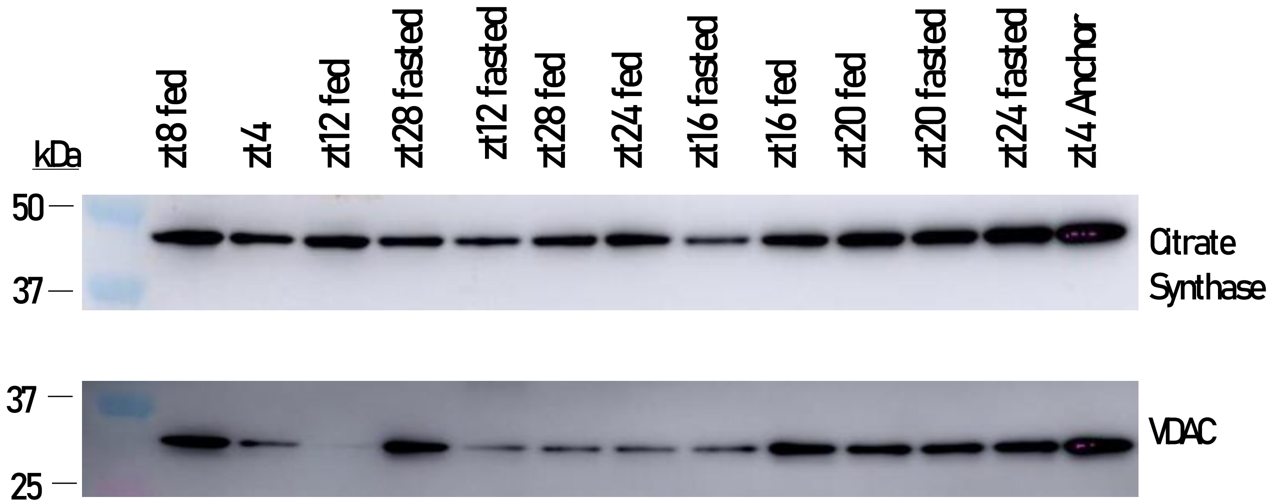
kDa
50
37
37
25
zt8 fed
zt4
zt12 fed
zt28 fasted
zt12 fasted
zt28 fed
zt24 fed
zt16 fasted
zt16 fed
zt20 fed
zt20 fasted
zt24 fasted
zt4 Anchor
Citrate Synthase
VDAC

Gel 10

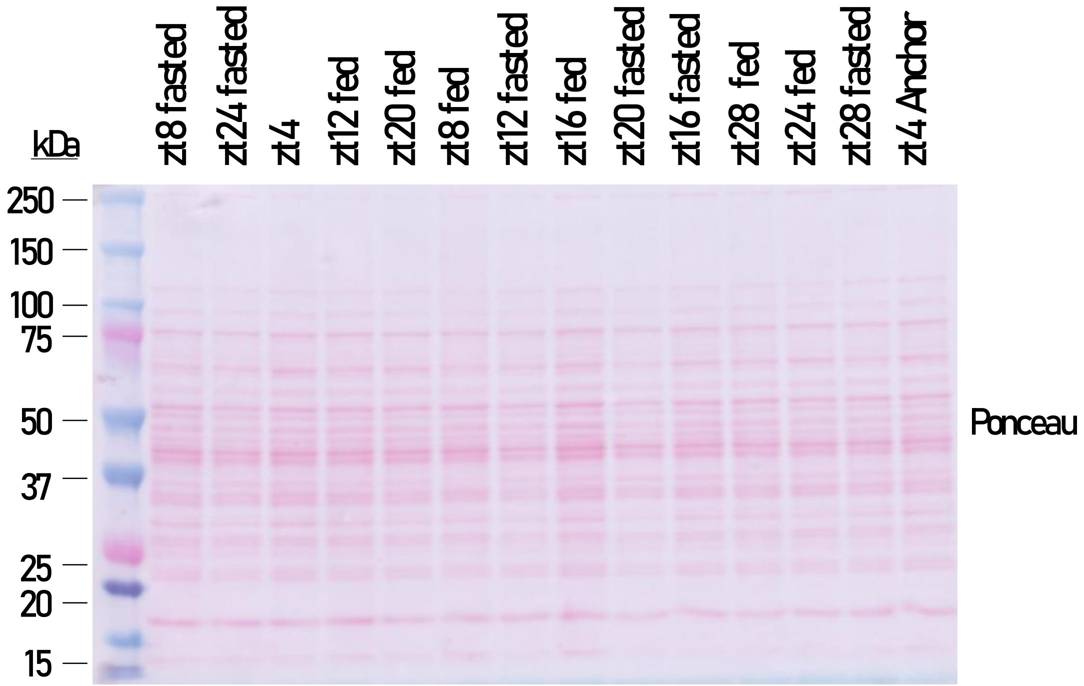
kDa
zt8 fasted
zt24 fasted
zt4
zt12 fed
zt20 fed
zt8 fed
zt12 fasted
zt16 fed
zt20 fasted
zt16 fasted
zt28 fed
zt24 fed
zt28 fasted
zt4 Anchor
250
150
100
75
50
37
25
20
15
Ponceau

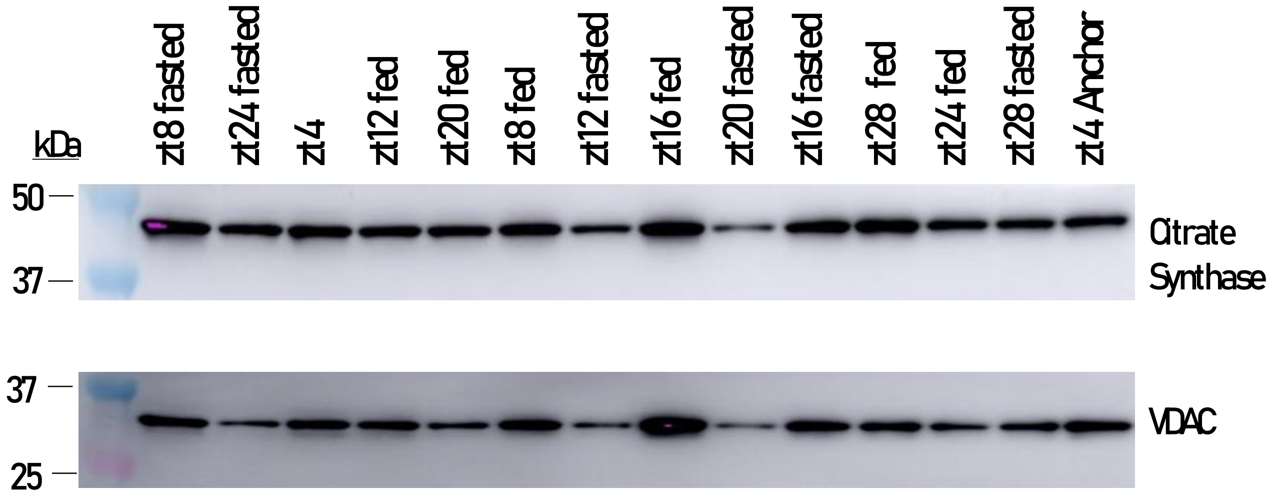
kDa
zt8 fasted
zt24 fasted
zt4
zt12 fed
zt20 fed
zt8 fed
zt12 fasted
zt16 fed
zt20 fasted
zt16 fasted
zt28 fed
zt24 fed
zt28 fasted
zt4 Anchor
50
37
Citrate Synthase
37
25
VDAC

Gel 11

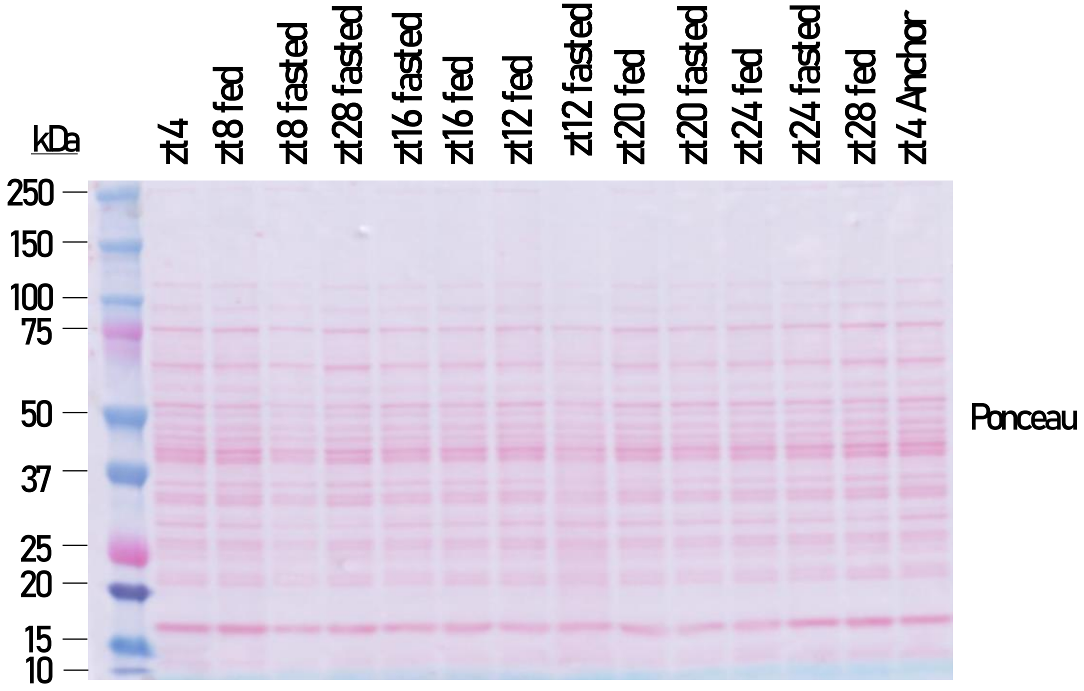
kDa
zt4
zt8 fed
zt8 fasted
zt28 fasted
zt16 fasted
zt16 fed
zt12 fed
zt12 fasted
zt20 fed
zt20 fasted
zt24 fed
zt24 fasted
zt28 fed
zt4 Anchor
250
150
100
75
50
37
25
20
15
10
Ponceau

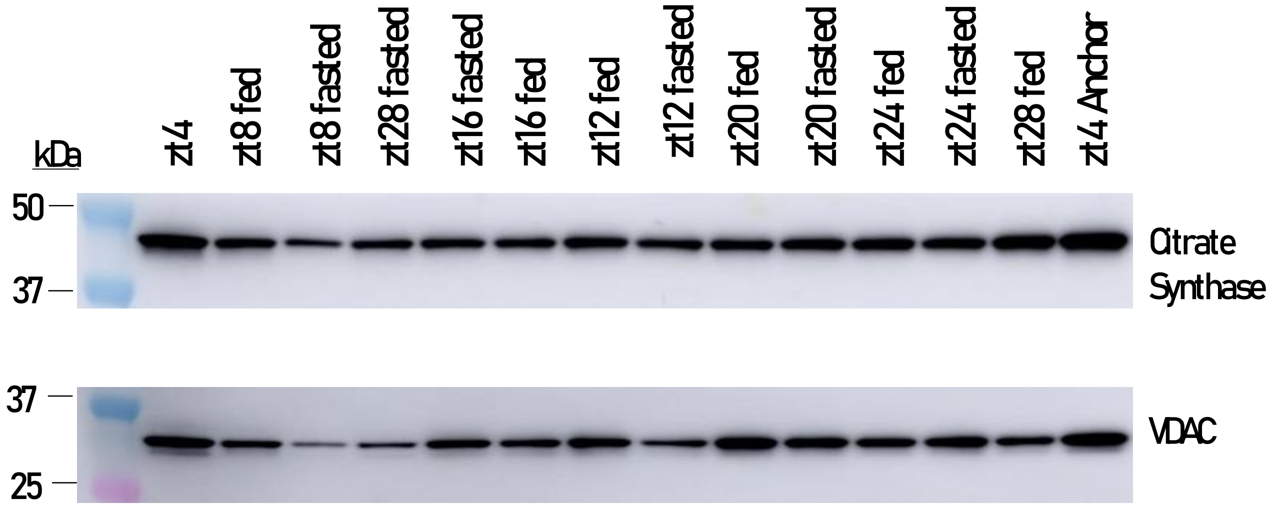
kDa
zt4
zt8 fed
zt8 fasted
zt28 fasted
zt16 fasted
zt16 fed
zt12 fed
zt12 fasted
zt20 fed
zt20 fasted
zt24 fed
zt24 fasted
zt28 fed
zt4 Anchor
50
37
Citrate Synthase
37
25
VDAC

Gel 12

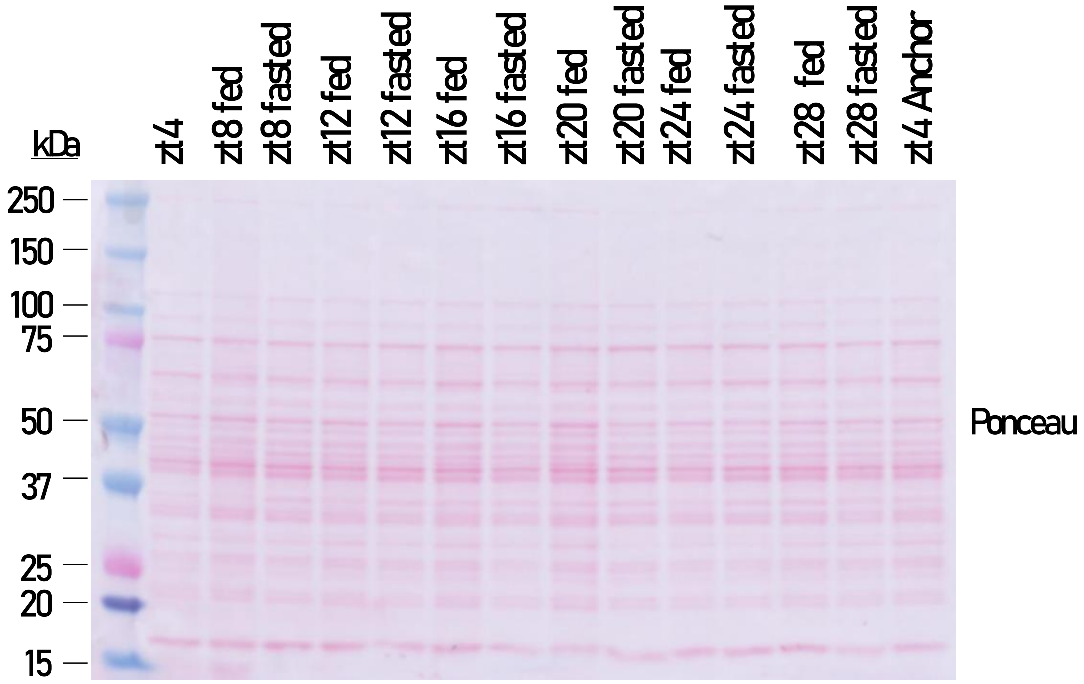
kDa
zt4
zt8 fed
zt8 fasted
zt12 fed
zt12 fasted
zt16 fed
zt16 fasted
zt20 fed
zt20 fasted
zt24 fed
zt24 fasted
zt28 fed
zt28 fasted
zt4 Anchor
250
150
100
75
50
37
25
20
15
Ponceau

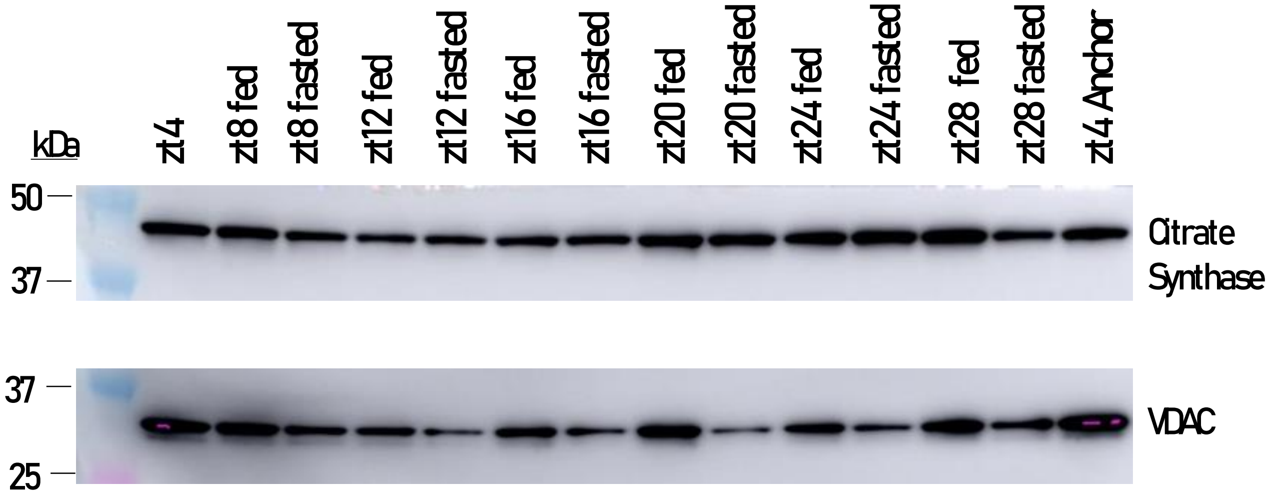
kDa
zt4
zt8 fed
zt8 fasted
zt12 fed
zt12 fasted
zt16 fed
zt16 fasted
zt20 fed
zt20 fasted
zt24 fed
zt24 fasted
zt28 fed
zt28 fasted
zt4 Anchor
50
37
Citrate Synthase
37
25
VDAC

Week 3

SOD2 (BD T 611580)
June 29-July 2 2021

Gel 1

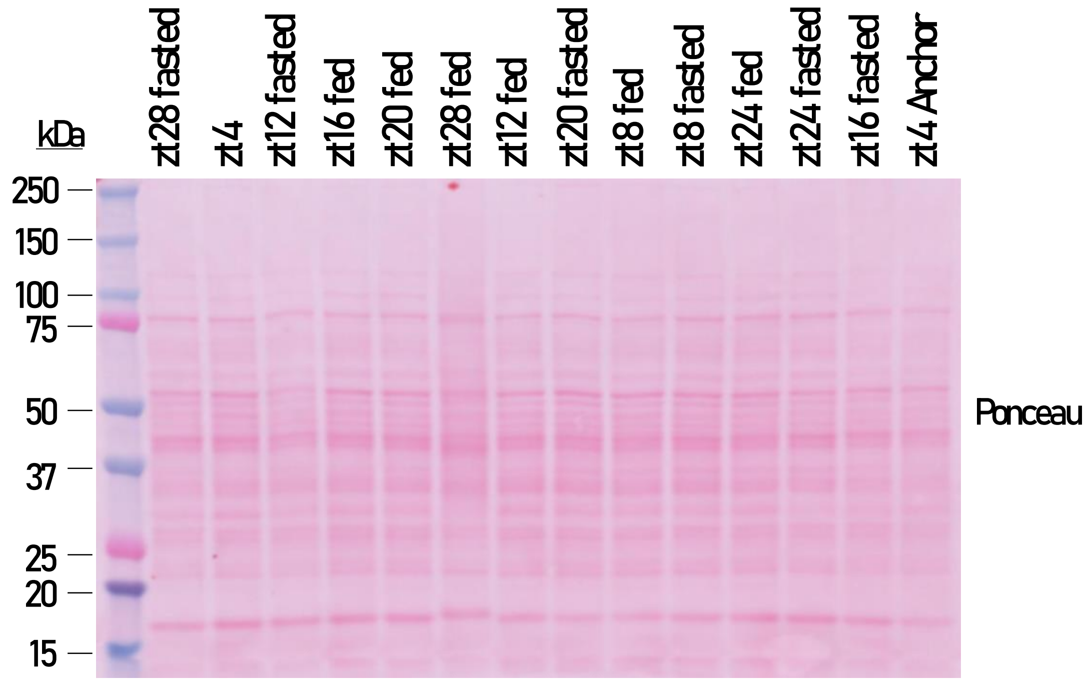
kDa
250
150
100
75
50
37
25
20
15
zt28 fasted
zt4
zt12 fasted
zt16 fed
zt20 fed
zt28 fed
zt12 fed
zt20 fasted
zt8 fed
zt8 fasted
zt24 fed
zt24 fasted
zt16 fasted
zt4 Anchor
Ponceau

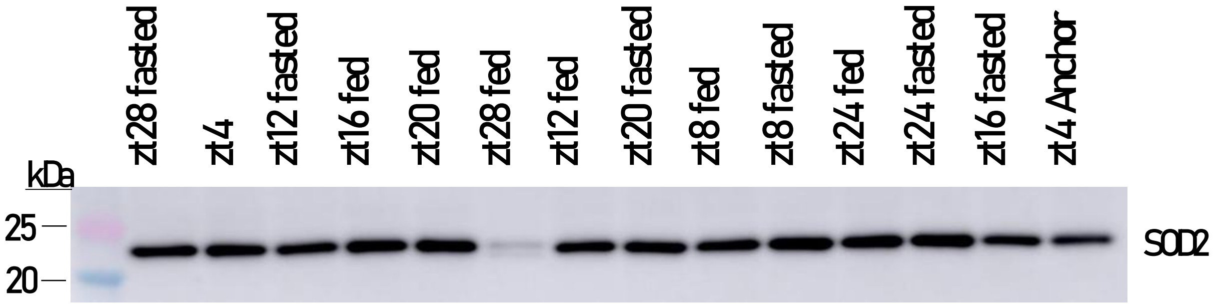
kDa
25
20
zt28 fasted
zt4
zt12 fasted
zt16 fed
zt20 fed
zt28 fed
zt12 fed
zt20 fasted
zt8 fed
zt8 fasted
zt24 fed
zt24 fasted
zt16 fasted
zt4 Anchor
SOD2

Gel 2

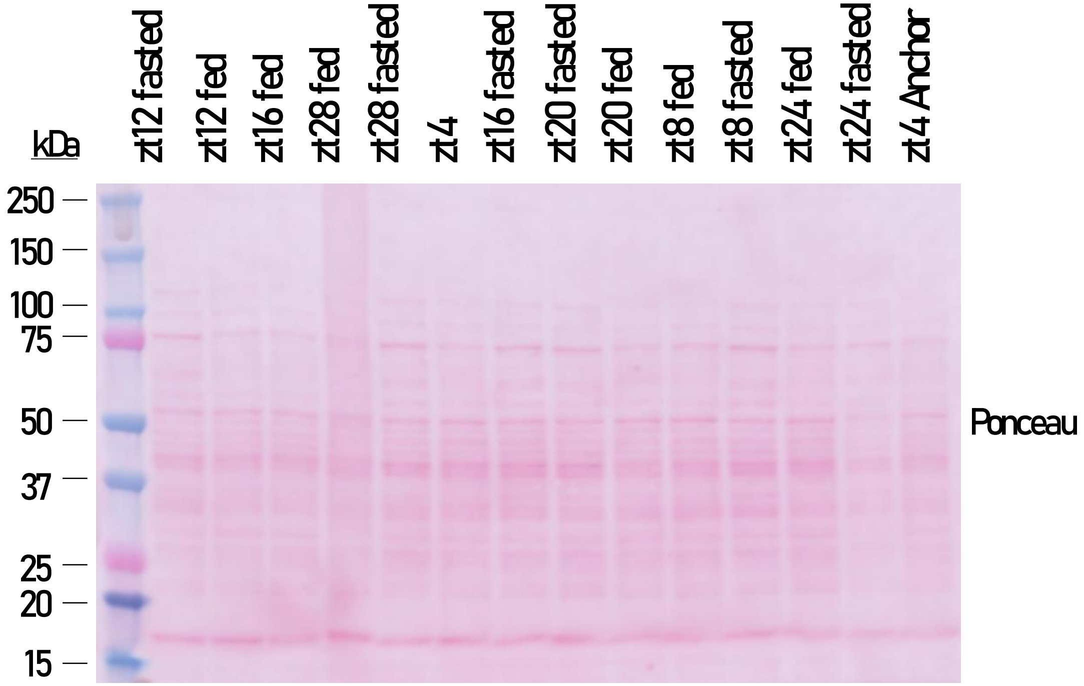
kDa
250
150
100
75
50
37
25
20
15
zt12 fasted
zt12 fed
zt16 fed
zt28 fed
zt28 fasted
zt4
zt16 fasted
zt20 fasted
zt20 fed
zt8 fed
zt8 fasted
zt24 fed
zt24 fasted
zt4 Anchor
Ponceau

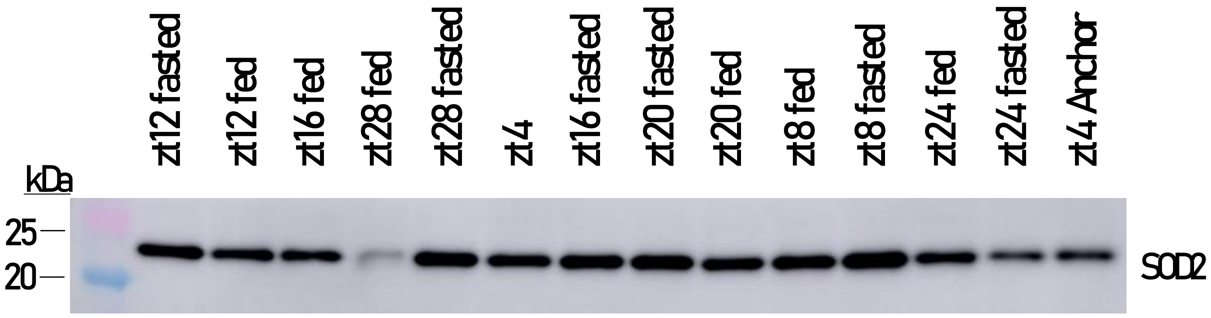
kDa
25
20
zt12 fasted
zt12 fed
zt16 fed
zt28 fed
zt28 fasted
zt4
zt16 fasted
zt20 fasted
zt20 fed
zt8 fed
zt8 fasted
zt24 fed
zt24 fasted
zt4 Anchor
SOD2

Gel 3

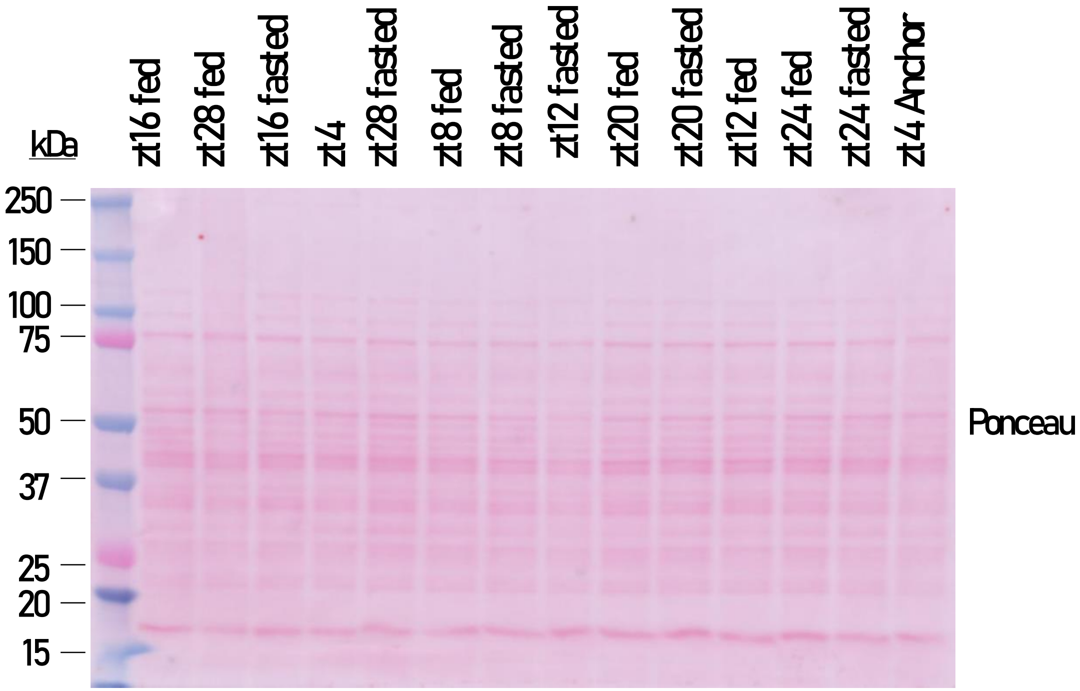
kDa
zt16 fed
zt28 fed
zt16 fasted
zt4
zt28 fasted
zt8 fed
zt8 fasted
zt12 fasted
zt20 fed
zt20 fasted
zt12 fed
zt24 fed
zt24 fasted
zt4 Anchor
250
150
100
75
50
37
25
20
15
Ponceau

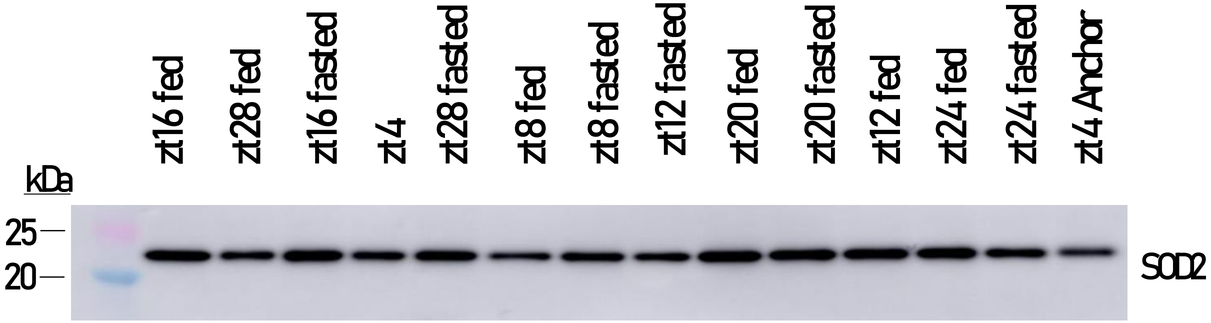
kDa
zt16 fed
zt28 fed
zt16 fasted
zt4
zt28 fasted
zt8 fed
zt8 fasted
zt12 fasted
zt20 fed
zt20 fasted
zt12 fed
zt24 fed
zt24 fasted
zt4 Anchor
25
20
SOD2

Gel 4

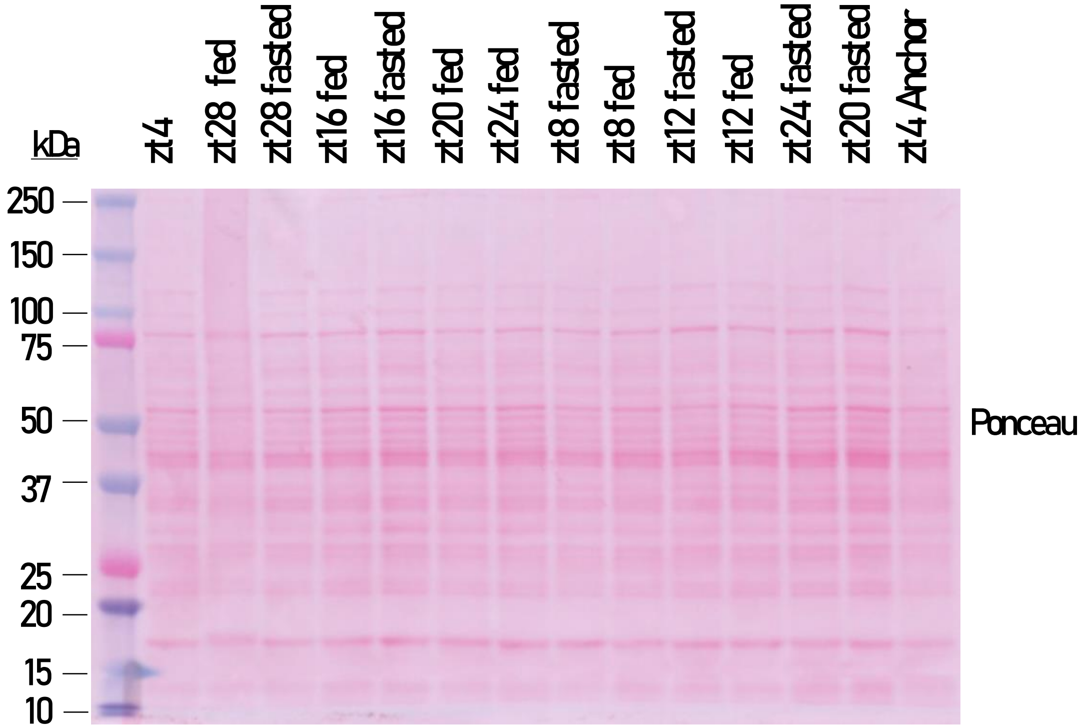
kDa
250
150
100
75
50
37
25
20
15
10
zt4
zt28 fed
zt28 fasted
zt16 fed
zt16 fasted
zt20 fed
zt24 fed
zt8 fasted
zt8 fed
zt12 fasted
zt12 fed
zt24 fasted
zt20 fasted
zt4 Anchor
Ponceau

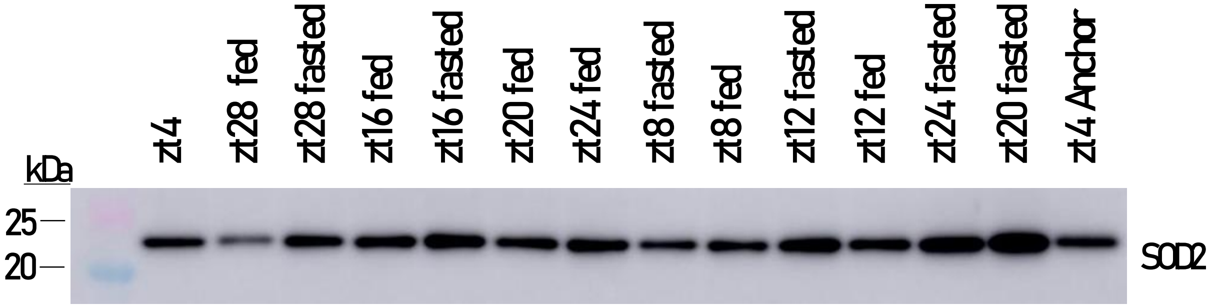
kDa
25
20
zt4
zt28 fed
zt28 fasted
zt16 fed
zt16 fasted
zt20 fed
zt24 fed
zt8 fasted
zt8 fed
zt12 fasted
zt12 fed
zt24 fasted
zt20 fasted
zt4 Anchor
SOD2

Gel 5

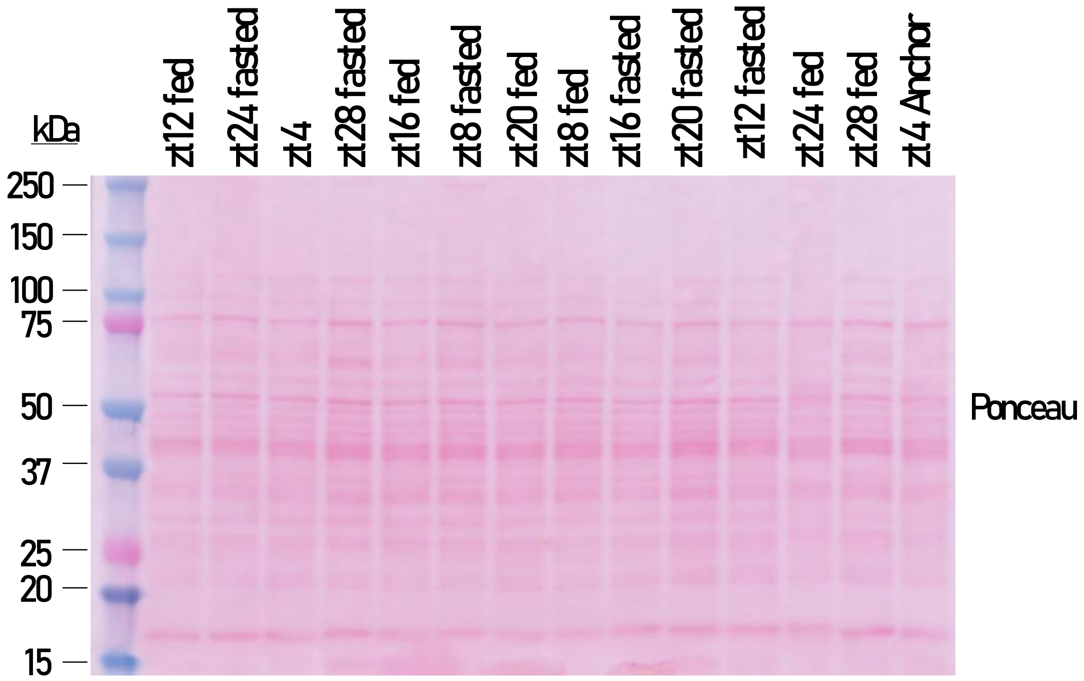
kDa
250
150
100
75
50
37
25
20
15
zt12 fed
zt24 fasted
zt4
zt28 fasted
zt16 fed
zt8 fasted
zt20 fed
zt8 fed
zt16 fasted
zt20 fasted
zt12 fasted
zt24 fed
zt28 fed
zt4 Anchor
Ponceau

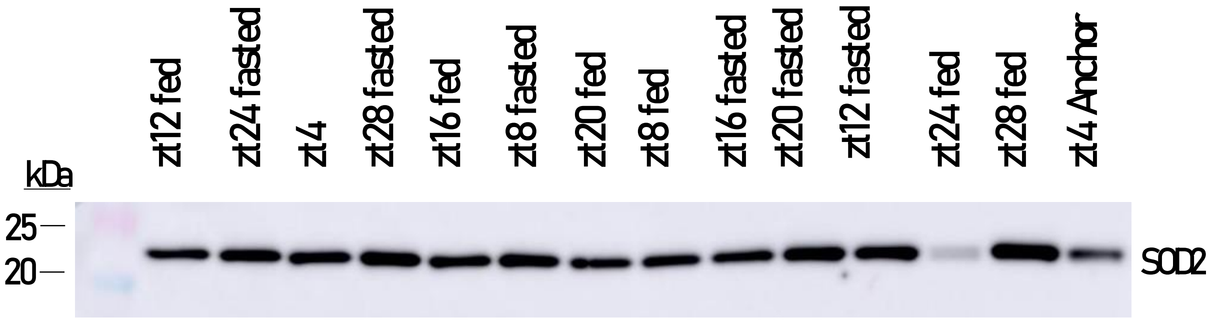
kDa
25
20
zt12 fed
zt24 fasted
zt4
zt28 fasted
zt16 fed
zt8 fasted
zt20 fed
zt8 fed
zt16 fasted
zt20 fasted
zt12 fasted
zt24 fed
zt28 fed
zt4 Anchor
SOD2

Gel 6

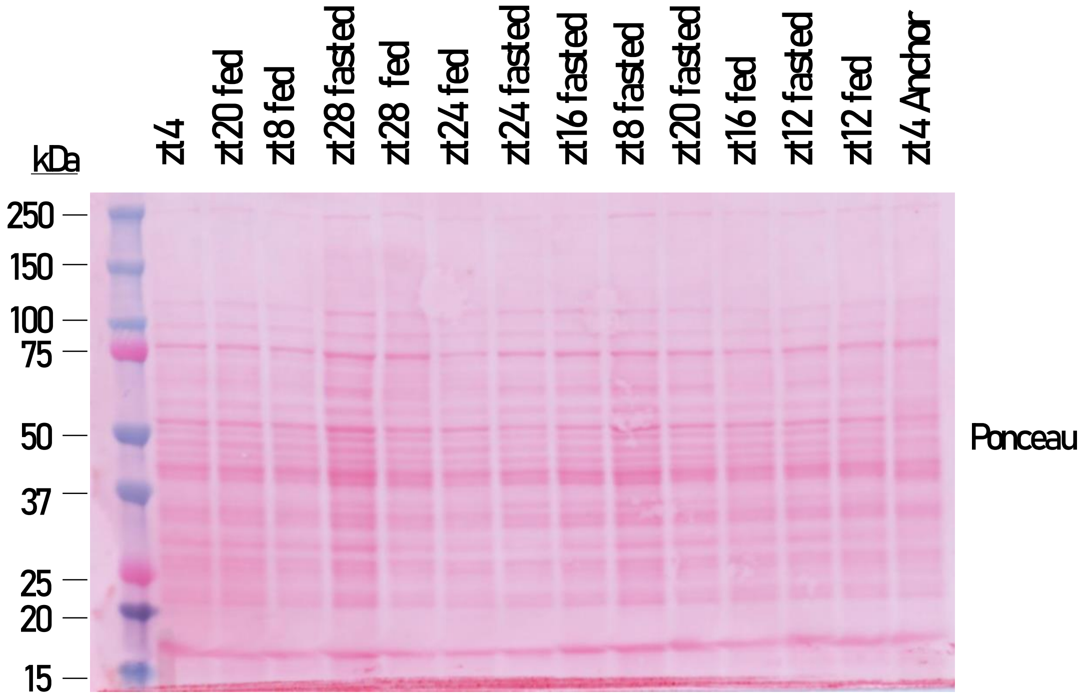

kDa
250
150
100
75
50
37
25
20
15
zt4
zt20 fed
zt8 fed
zt28 fasted
zt28 fed
zt24 fed
zt24 fasted
zt16 fasted
zt8 fasted
zt20 fasted
zt16 fed
zt12 fasted
zt12 fed
zt4 Anchor
Ponceau

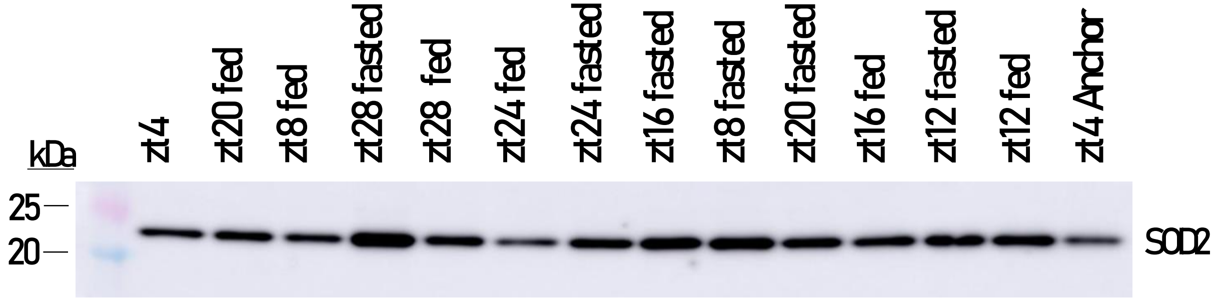

kDa
25
20
zt4
zt20 fed
zt8 fed
zt28 fasted
zt28 fed
zt24 fed
zt24 fasted
zt16 fasted
zt8 fasted
zt20 fasted
zt16 fed
zt12 fasted
zt12 fed
zt4 Anchor
SOD2

Gel 7

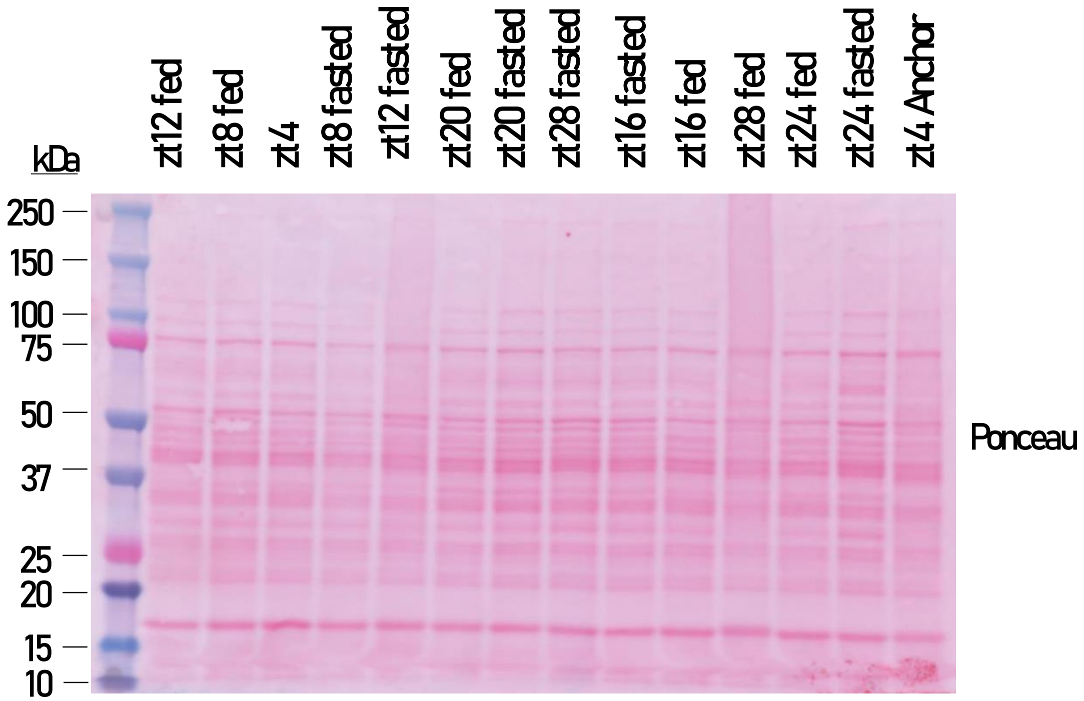

zt12 fed
zt8 fed
zt4
zt8 fasted
zt12 fasted
zt20 fed
zt20 fasted
zt28 fasted
zt16 fasted
zt16 fed
zt28 fed
zt24 fed
zt24 fasted
zt4 Anchor
kDa
250
150
100
75
50
37
25
20
15
10
Ponceau

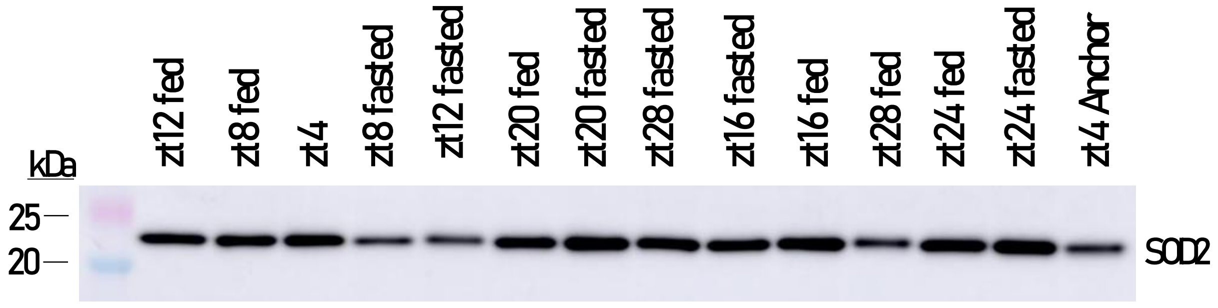

zt12 fed
zt8 fed
zt4
zt8 fasted
zt12 fasted
zt20 fed
zt20 fasted
zt28 fasted
zt16 fasted
zt16 fed
zt28 fed
zt24 fed
zt24 fasted
zt4 Anchor
kDa
25
20
SOD2

Gel 8

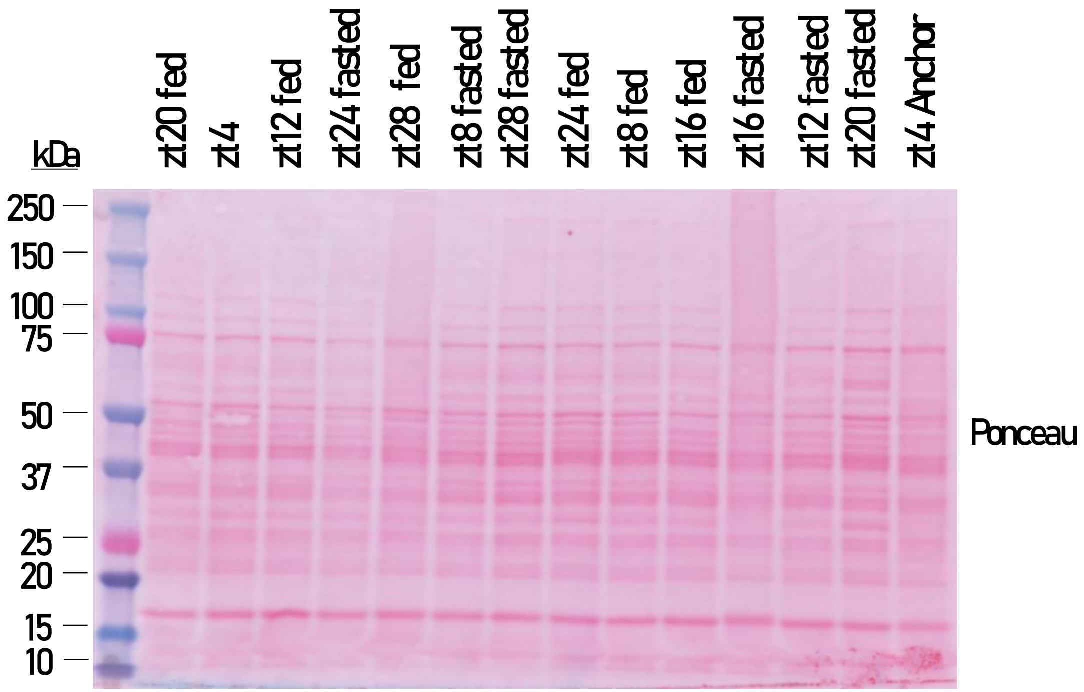
kDa
250
150
100
75
50
37
25
20
15
10
zt20 fed
zt4
zt12 fed
zt24 fasted
zt28 fed
zt8 fasted
zt28 fasted
zt24 fed
zt8 fed
zt16 fed
zt16 fasted
zt12 fasted
zt20 fasted
zt4 Anchor
Ponceau

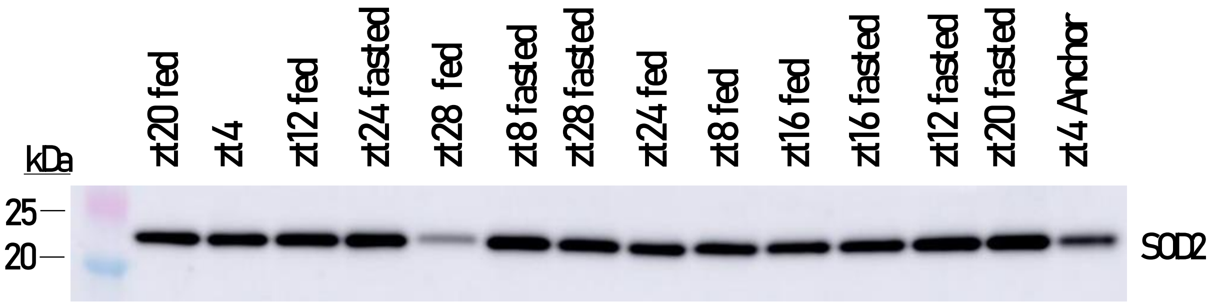
kDa
25
20
zt20 fed
zt4
zt12 fed
zt24 fasted
zt28 fed
zt8 fasted
zt28 fasted
zt24 fed
zt8 fed
zt16 fed
zt16 fasted
zt12 fasted
zt20 fasted
zt4 Anchor
SOD2

Gel 9

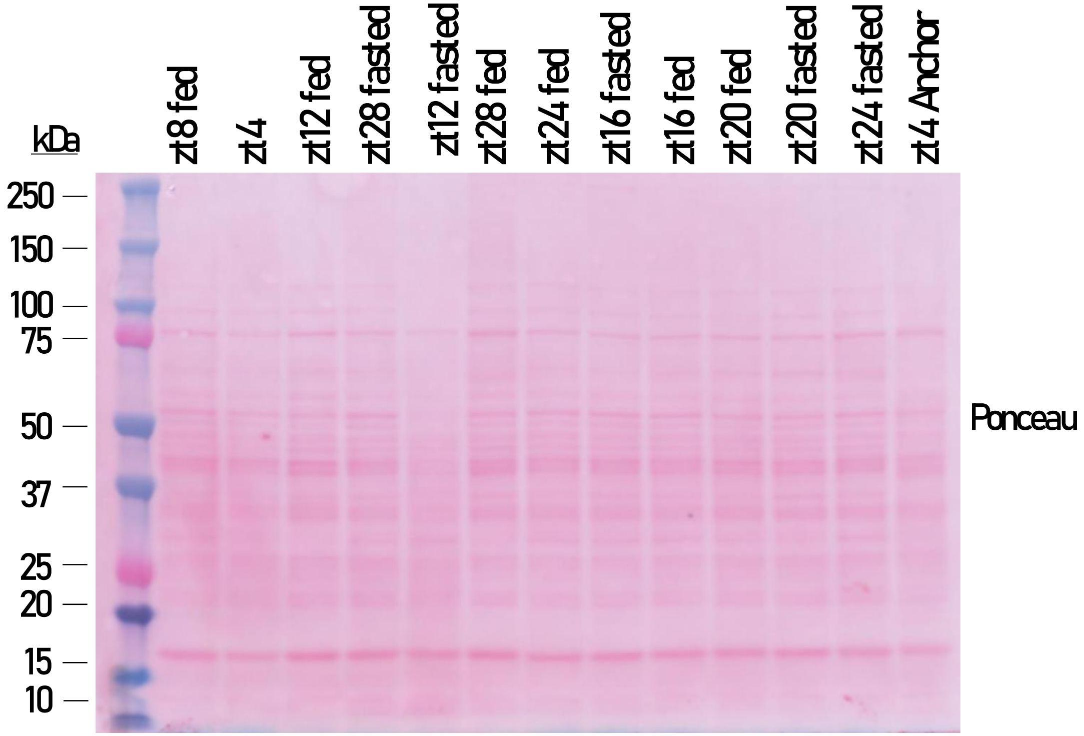
kDa
250
150
100
75
50
37
25
20
15
10
zt8 fed
zt4
zt12 fed
zt28 fasted
zt12 fasted
zt28 fed
zt24 fed
zt16 fasted
zt16 fed
zt20 fed
zt20 fasted
zt24 fasted
zt4 Anchor
Ponceau

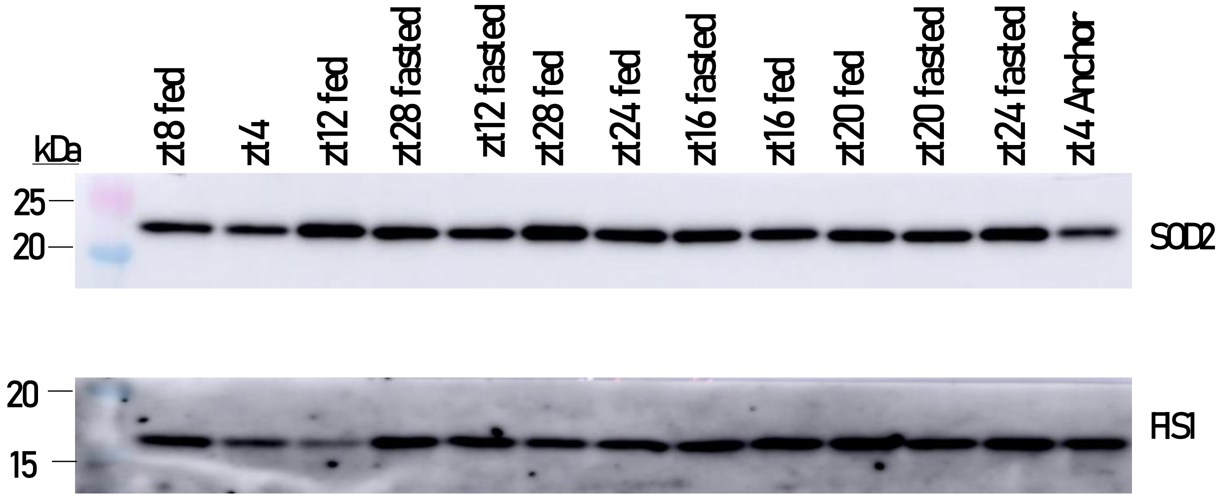
kDa
25
20
20
15
zt8 fed
zt4
zt12 fed
zt28 fasted
zt12 fasted
zt28 fed
zt24 fed
zt16 fasted
zt16 fed
zt20 fed
zt20 fasted
zt24 fasted
zt4 Anchor
SOD2
FIS1

Gel 10

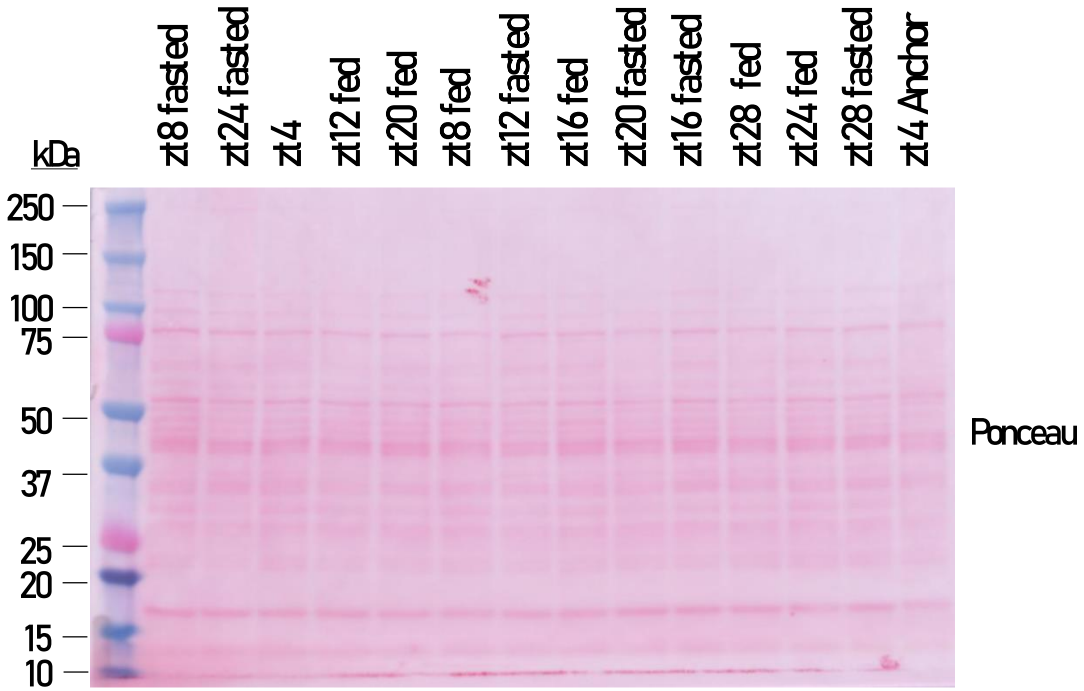
kDa
250
150
100
75
50
37
25
20
15
10
zt8 fasted
zt24 fasted
zt4
zt12 fed
zt20 fed
zt8 fed
zt12 fasted
zt16 fed
zt20 fasted
zt16 fasted
zt28 fed
zt24 fed
zt28 fasted
zt4 Anchor
Ponceau

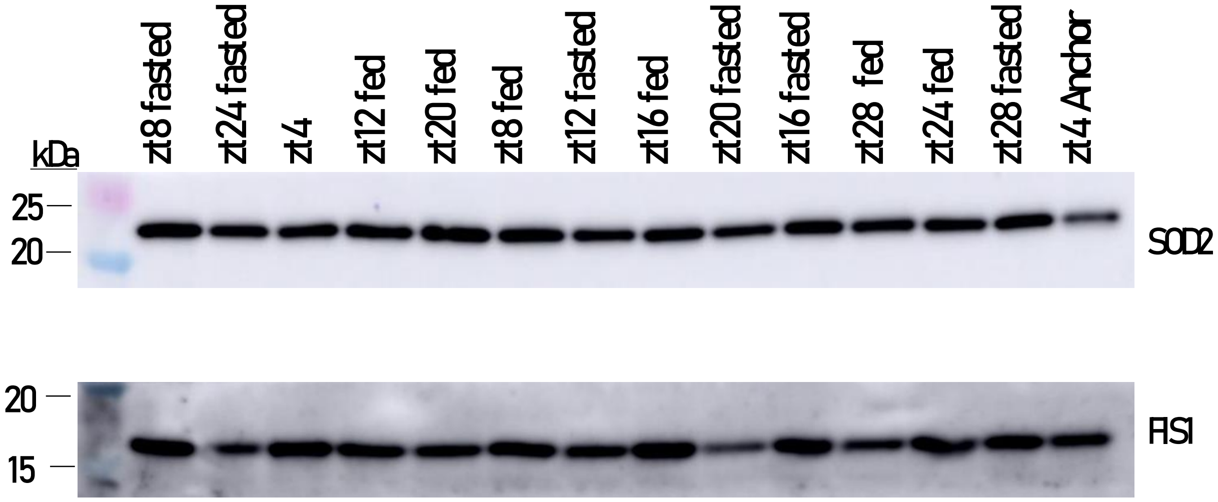
kDa
25
20
20
15
zt8 fasted
zt24 fasted
zt4
zt12 fed
zt20 fed
zt8 fed
zt12 fasted
zt16 fed
zt20 fasted
zt16 fasted
zt28 fed
zt24 fed
zt28 fasted
zt4 Anchor
SOD2
FIS1

Gel 11

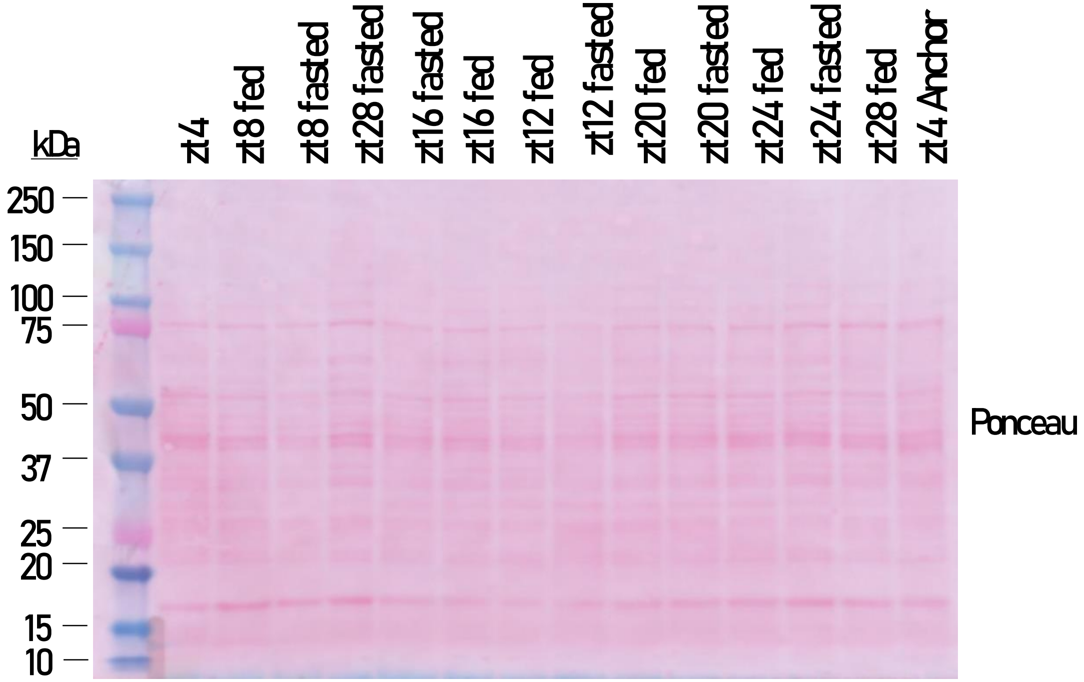
zt4
zt8 fed
zt8 fasted
zt28 fasted
zt16 fasted
zt16 fed
zt12 fed
zt12 fasted
zt20 fed
zt20 fasted
zt24 fed
zt24 fasted
zt28 fed
zt4 Anchor
kDa
250
150
100
75
50
37
25
20
15
10
Ponceau

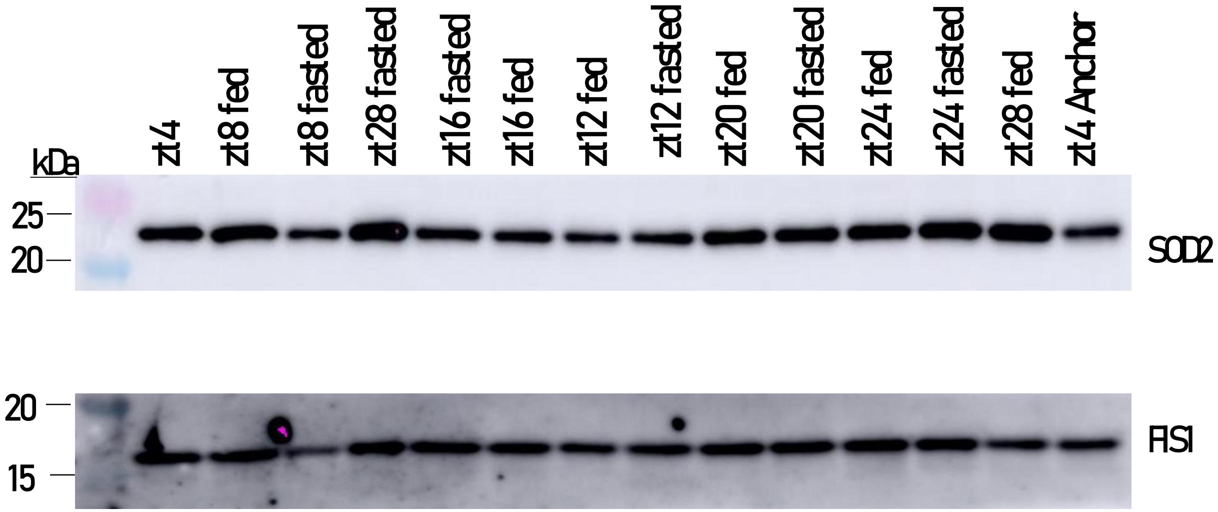
zt4
zt8 fed
zt8 fasted
zt28 fasted
zt16 fasted
zt16 fed
zt12 fed
zt12 fasted
zt20 fed
zt20 fasted
zt24 fed
zt24 fasted
zt28 fed
zt4 Anchor
kDa
25
20
SOD2
20
15
FIS1

Gel 12

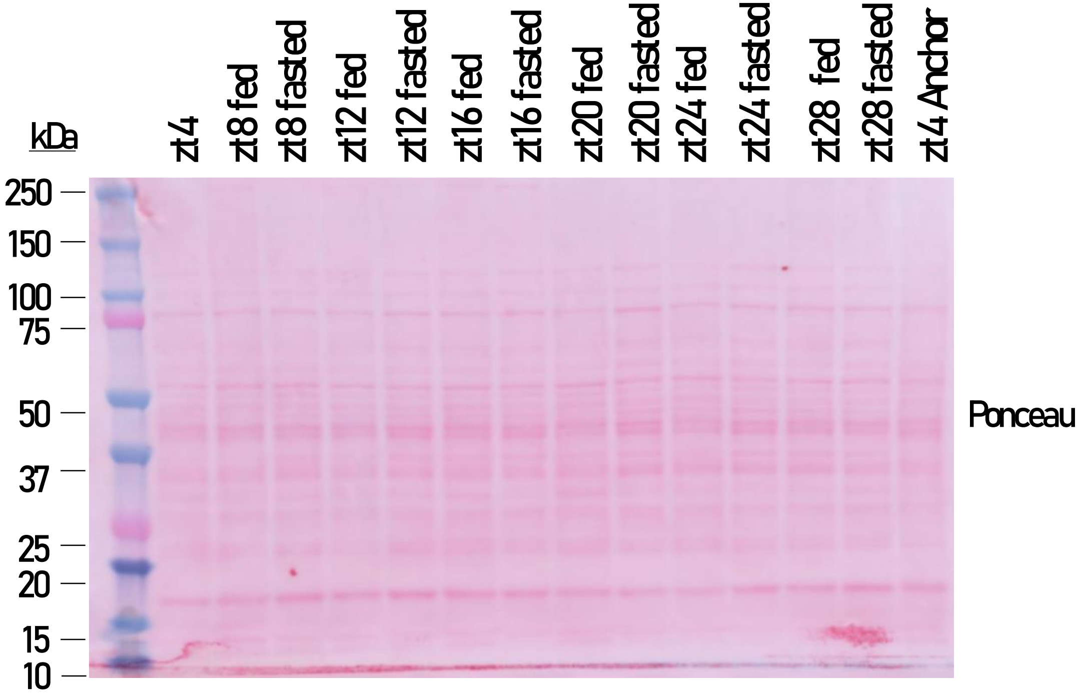
kDa
zt4
zt8 fed
zt8 fasted
zt12 fed
zt12 fasted
zt16 fed
zt16 fasted
zt20 fed
zt20 fasted
zt24 fed
zt24 fasted
zt28 fed
zt28 fasted
zt4 Anchor
250
150
100
75
50
37
25
20
15
10
Ponceau

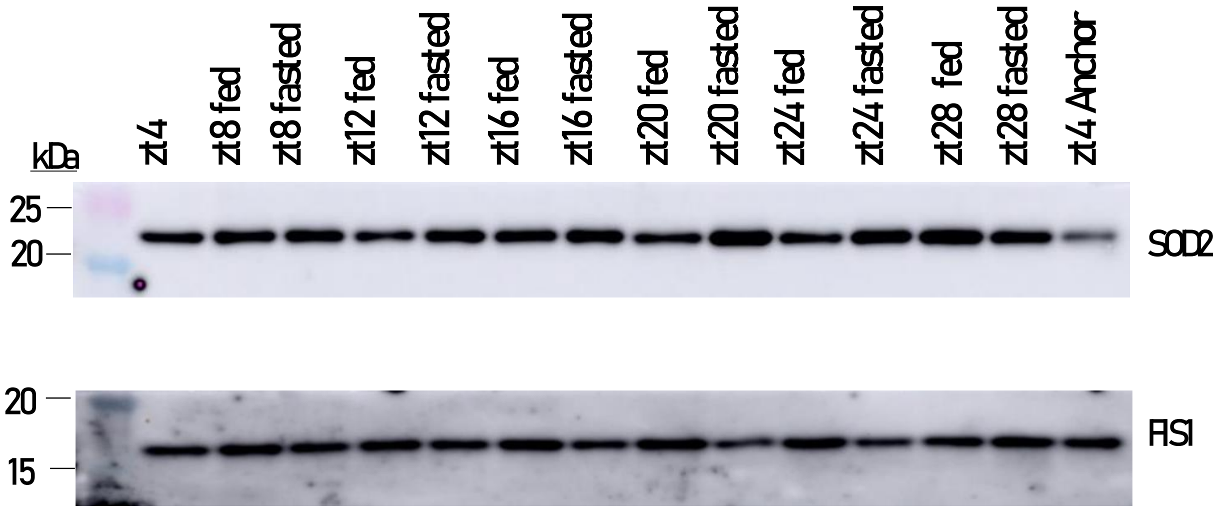
kDa
zt4
zt8 fed
zt8 fasted
zt12 fed
zt12 fasted
zt16 fed
zt16 fasted
zt20 fed
zt20 fasted
zt24 fed
zt24 fasted
zt28 fed
zt28 fasted
zt4 Anchor
25
20
SOD2
20
15
FIS1

Week 4

DRP1 (Abcam, ab56788), FIS1(Abcam, ab229969)
July 5-9 2021

Gel 1

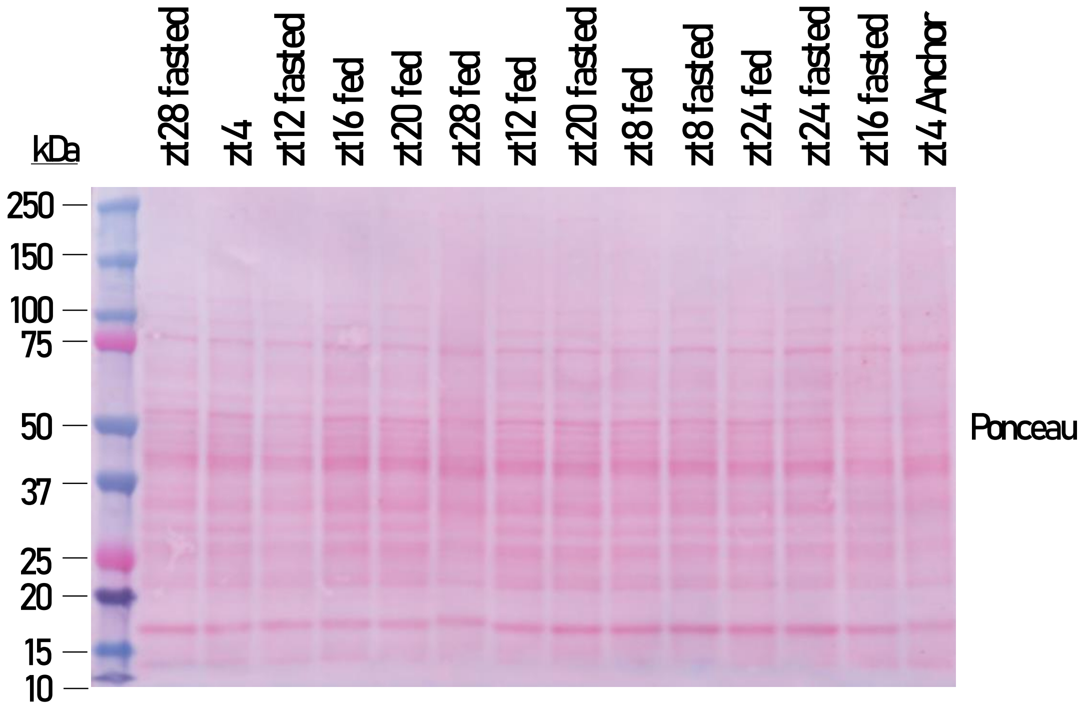
kDa
250
150
100
75
50
37
25
20
15
10
zt28 fasted
zt4
zt12 fasted
zt16 fed
zt20 fed
zt28 fed
zt12 fed
zt20 fasted
zt8 fed
zt8 fasted
zt24 fed
zt24 fasted
zt16 fasted
zt4 Anchor
Ponceau

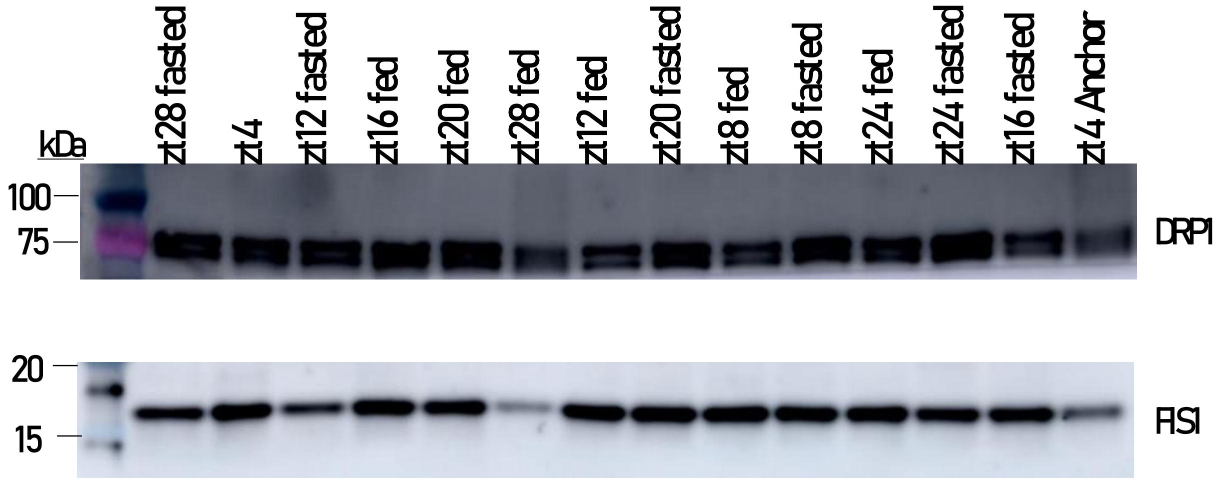
kDa
100
75
20
15
zt28 fasted
zt4
zt12 fasted
zt16 fed
zt20 fed
zt28 fed
zt12 fed
zt20 fasted
zt8 fed
zt8 fasted
zt24 fed
zt24 fasted
zt16 fasted
zt4 Anchor
DRP1
FIS1

Gel 2

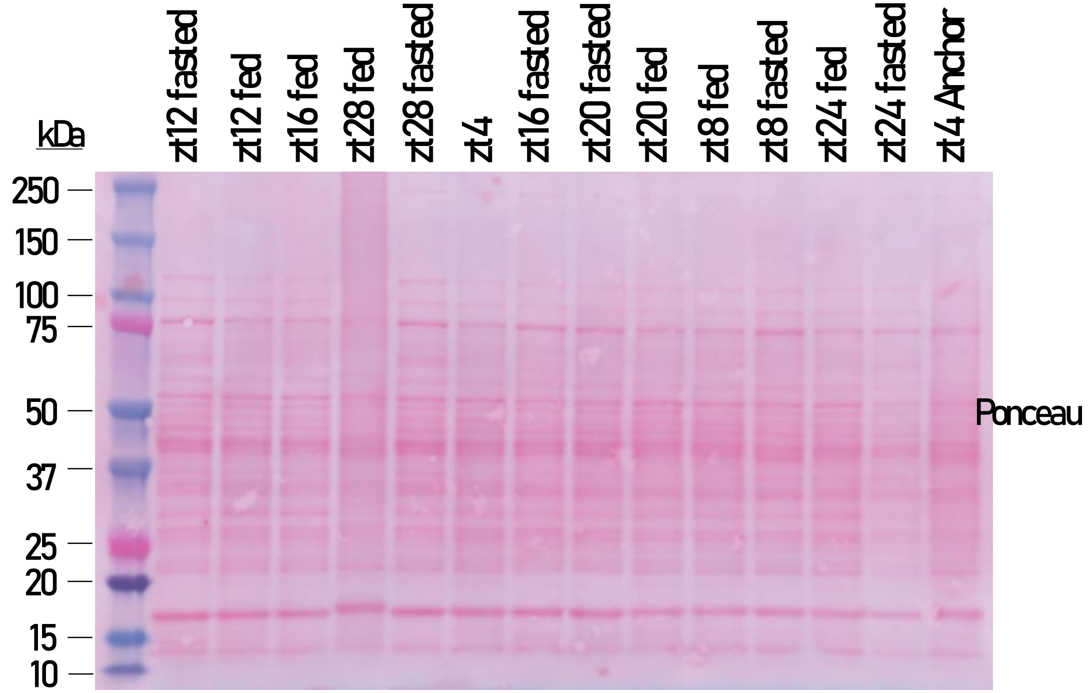
kDa
250
150
100
75
50
37
25
20
15
10
zt12 fasted
zt12 fed
zt16 fed
zt28 fed
zt28 fasted
zt4
zt16 fasted
zt20 fasted
zt20 fed
zt8 fed
zt8 fasted
zt24 fed
zt24 fasted
zt4 Anchor
Ponceau

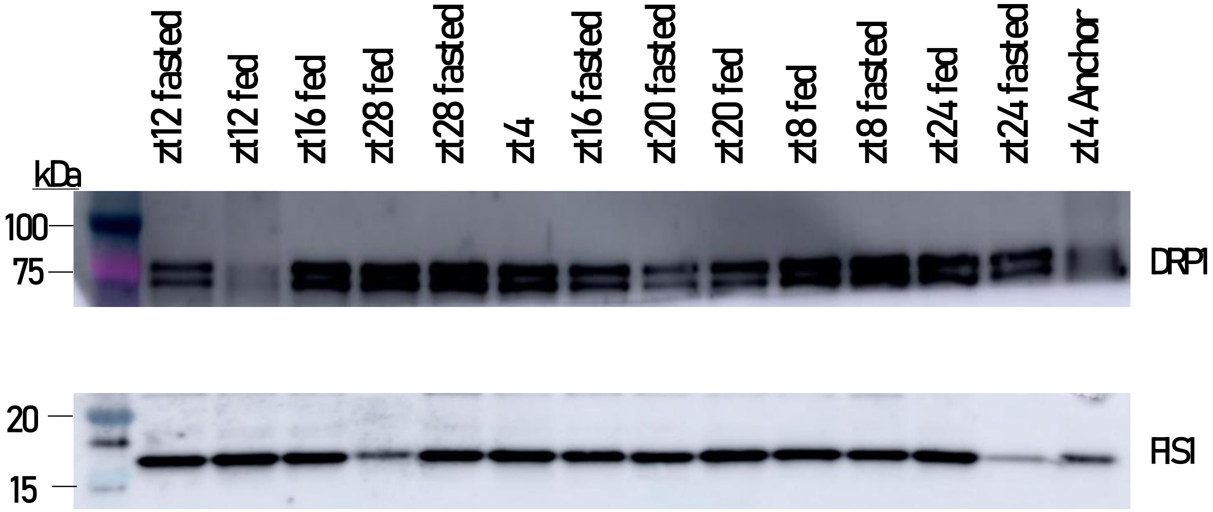
kDa
100
75
20
15
zt12 fasted
zt12 fed
zt16 fed
zt28 fed
zt28 fasted
zt4
zt16 fasted
zt20 fasted
zt20 fed
zt8 fed
zt8 fasted
zt24 fed
zt24 fasted
zt4 Anchor
DRP1
FIS1

Gel 3

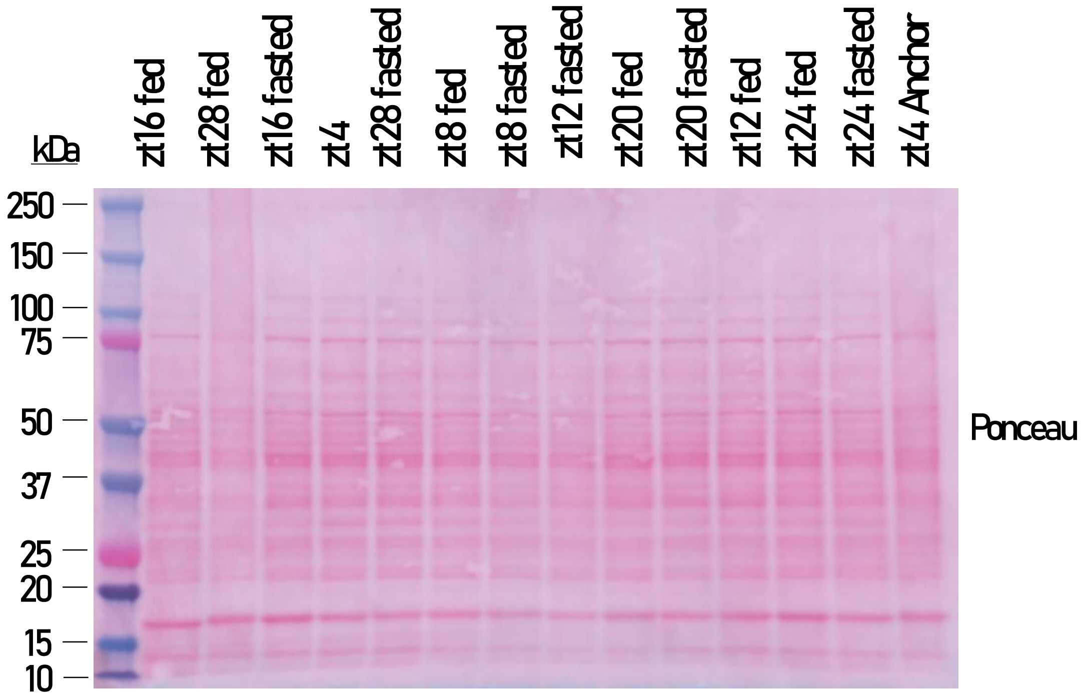
zt16 fed
zt28 fed
zt16 fasted
zt4
zt28 fasted
zt8 fed
zt8 fasted
zt12 fasted
zt20 fed
zt20 fasted
zt12 fed
zt24 fed
zt24 fasted
zt4 Anchor
kDa
250
150
100
75
50
37
25
20
15
10
Ponceau

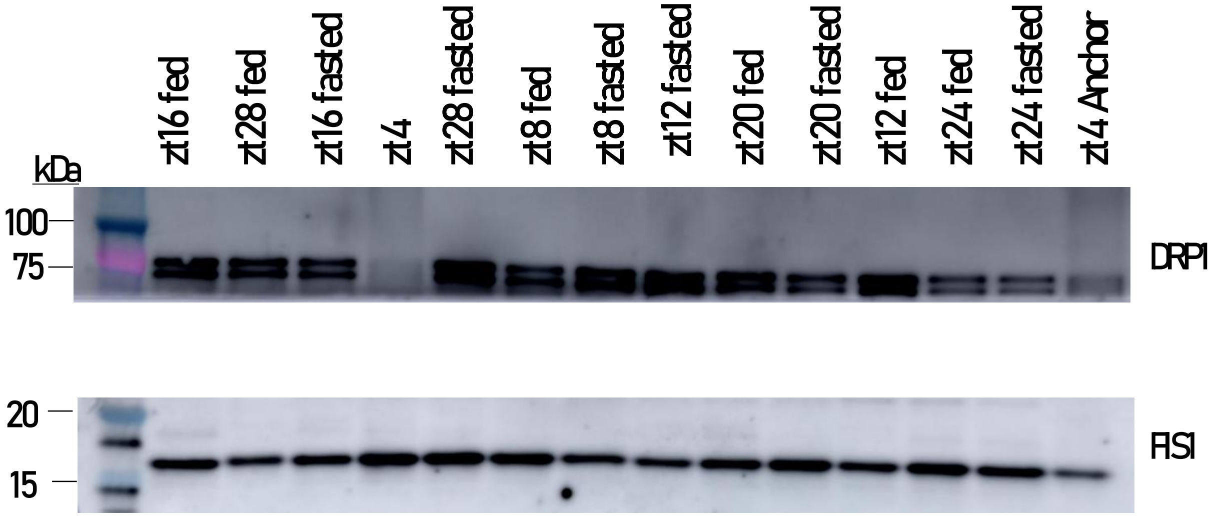
zt16 fed
zt28 fed
zt16 fasted
zt4
zt28 fasted
zt8 fed
zt8 fasted
zt12 fasted
zt20 fed
zt20 fasted
zt12 fed
zt24 fed
zt24 fasted
zt4 Anchor
kDa
100
75
DRP1
20
15
FIS1

Gel 4

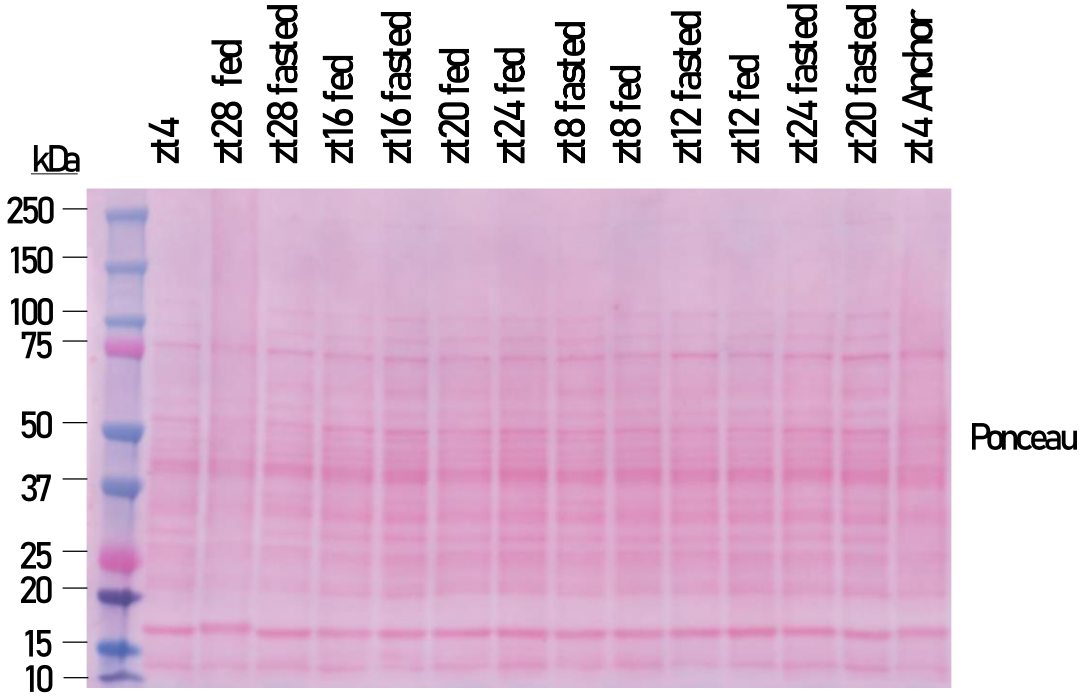
kDa
250
150
100
75
50
37
25
20
15
10
zt4
zt28 fed
zt28 fasted
zt16 fed
zt16 fasted
zt20 fed
zt24 fed
zt8 fasted
zt8 fed
zt12 fasted
zt12 fed
zt24 fasted
zt20 fasted
zt4 Anchor
Ponceau

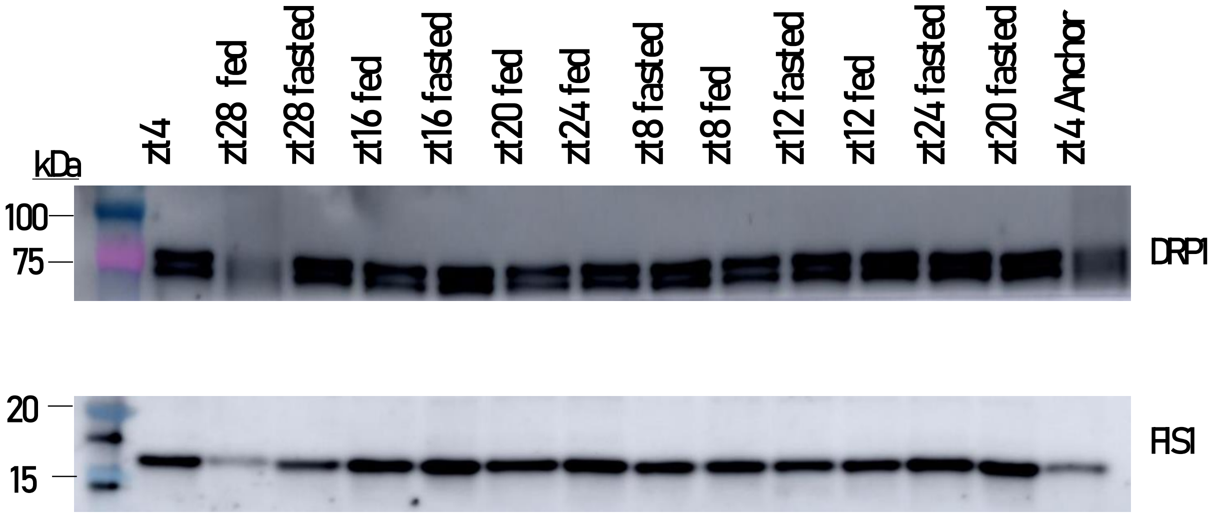
kDa
100
75
20
15
zt4
zt28 fed
zt28 fasted
zt16 fed
zt16 fasted
zt20 fed
zt24 fed
zt8 fasted
zt8 fed
zt12 fasted
zt12 fed
zt24 fasted
zt20 fasted
zt4 Anchor
DRP1
FIS1

Gel 5

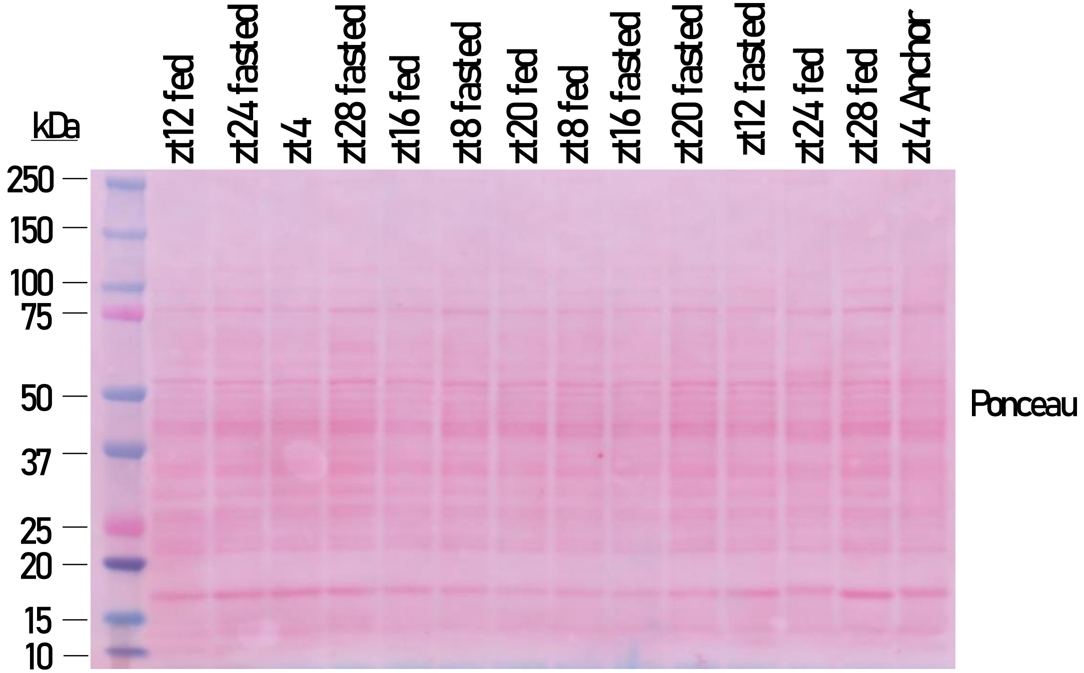
kDa
250
150
100
75
50
37
25
20
15
10
zt12 fed
zt24 fasted
zt4
zt28 fasted
zt16 fed
zt8 fasted
zt20 fed
zt8 fed
zt16 fasted
zt20 fasted
zt12 fasted
zt24 fed
zt28 fed
zt4 Anchor
Ponceau

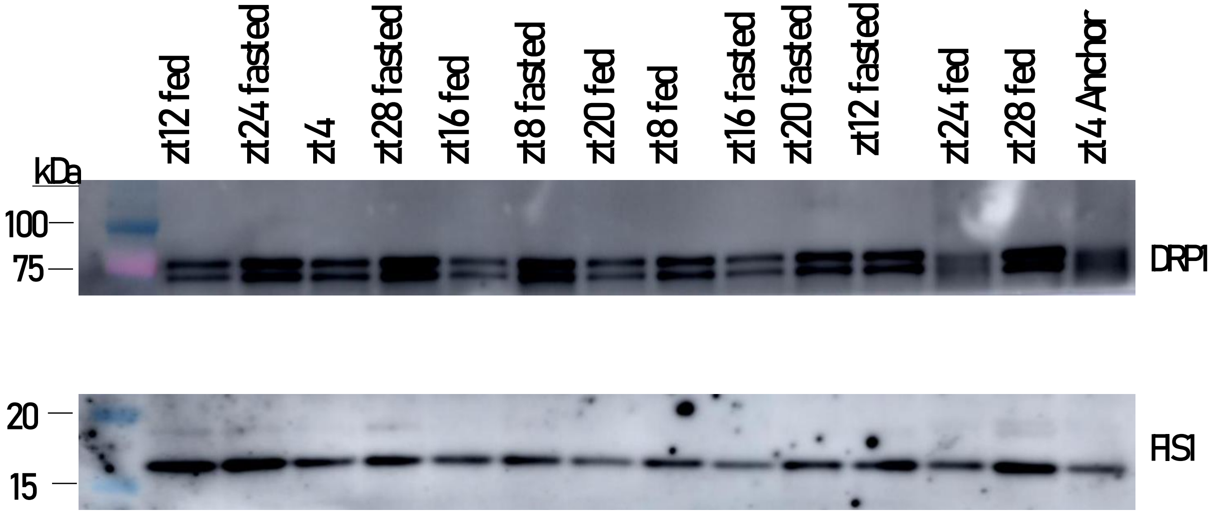
zt12 fed
zt24 fasted
zt4
zt28 fasted
zt16 fed
zt8 fasted
zt20 fed
zt8 fed
zt16 fasted
zt20 fasted
zt12 fasted
zt24 fed
zt28 fed
zt4 Anchor
kDa
100
75
DRP1
20
15
FIS1

Gel 6

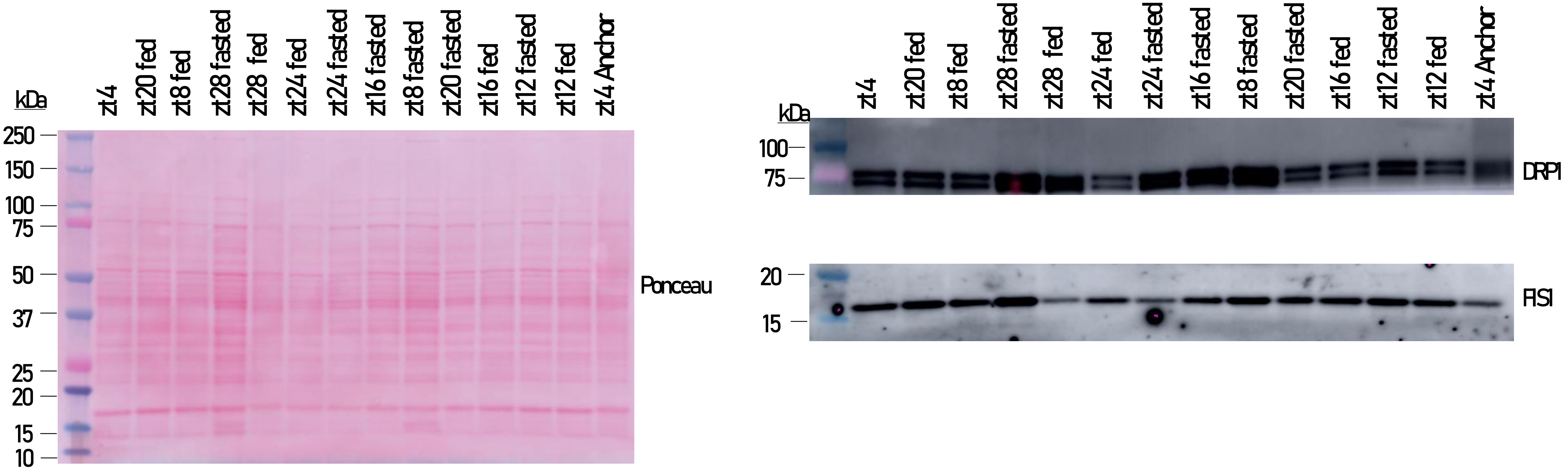
kDa
zt4
zt20 fed
zt8 fed
zt28 fasted
zt28 fed
zt24 fed
zt24 fasted
zt16 fasted
zt8 fasted
zt20 fasted
zt16 fed
zt12 fasted
zt12 fed
zt4 Anchor
250
150
100
75
50
37
25
20
15
10
Ponceau
kDa
100
75
DRP1
20
15
FIS1

Gel 7

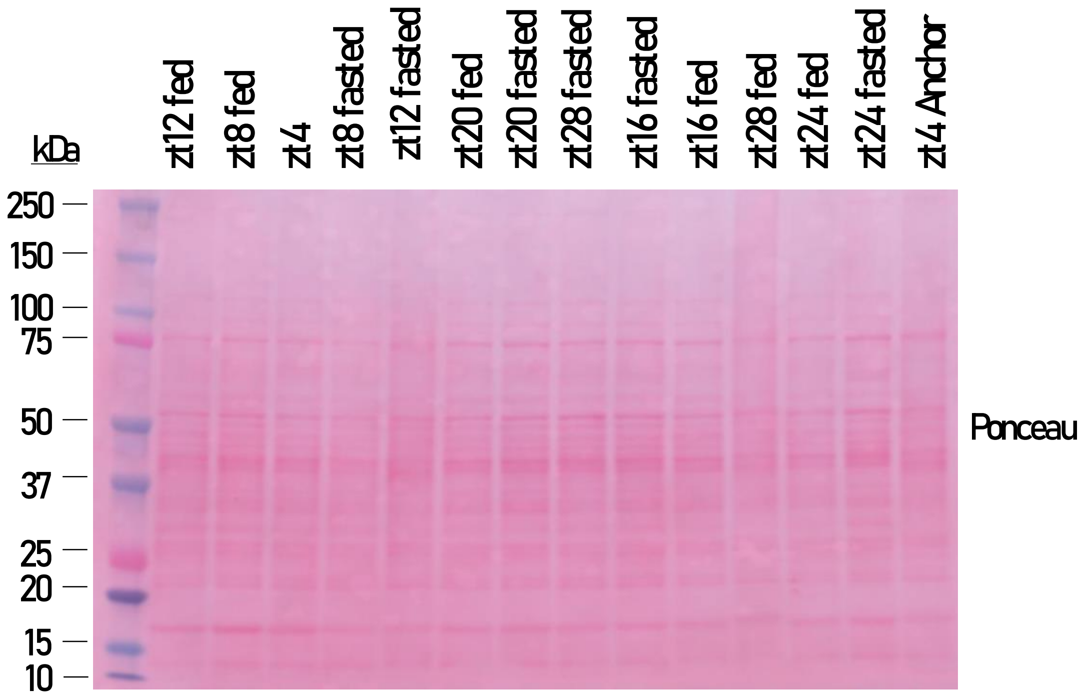
kDa
250
150
100
75
50
37
25
20
15
10
zt12 fed
zt8 fed
zt4
zt8 fasted
zt12 fasted
zt20 fed
zt20 fasted
zt28 fasted
zt16 fasted
zt16 fed
zt28 fed
zt24 fed
zt24 fasted
zt4 Anchor
Ponceau

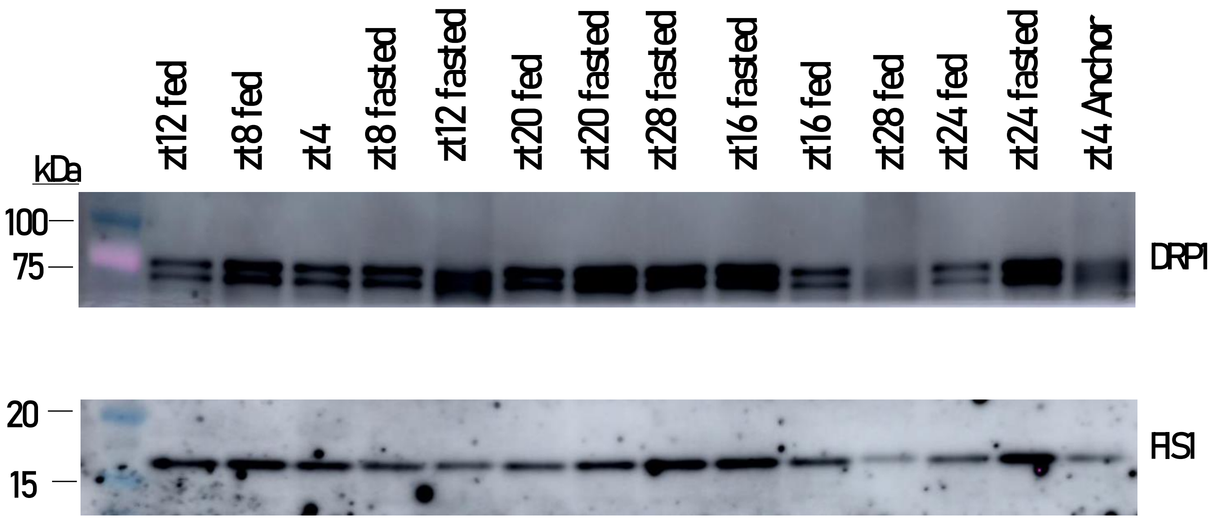
kDa
100
75
20
15
zt12 fed
zt8 fed
zt4
zt8 fasted
zt12 fasted
zt20 fed
zt20 fasted
zt28 fasted
zt16 fasted
zt16 fed
zt28 fed
zt24 fed
zt24 fasted
zt4 Anchor
DRP1
FIS1

Gel 7

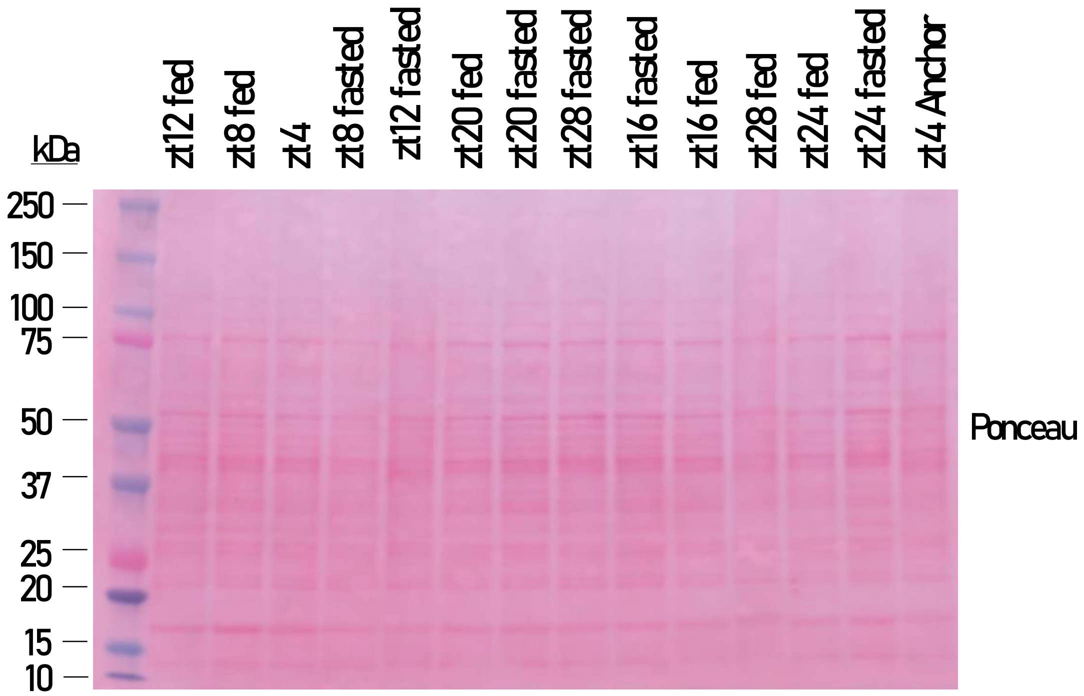
kDa
250
150
100
75
50
37
25
20
15
10
zt12 fed
zt8 fed
zt4
zt8 fasted
zt12 fasted
zt20 fed
zt20 fasted
zt28 fasted
zt16 fasted
zt16 fed
zt28 fed
zt24 fed
zt24 fasted
zt4 Anchor
Ponceau

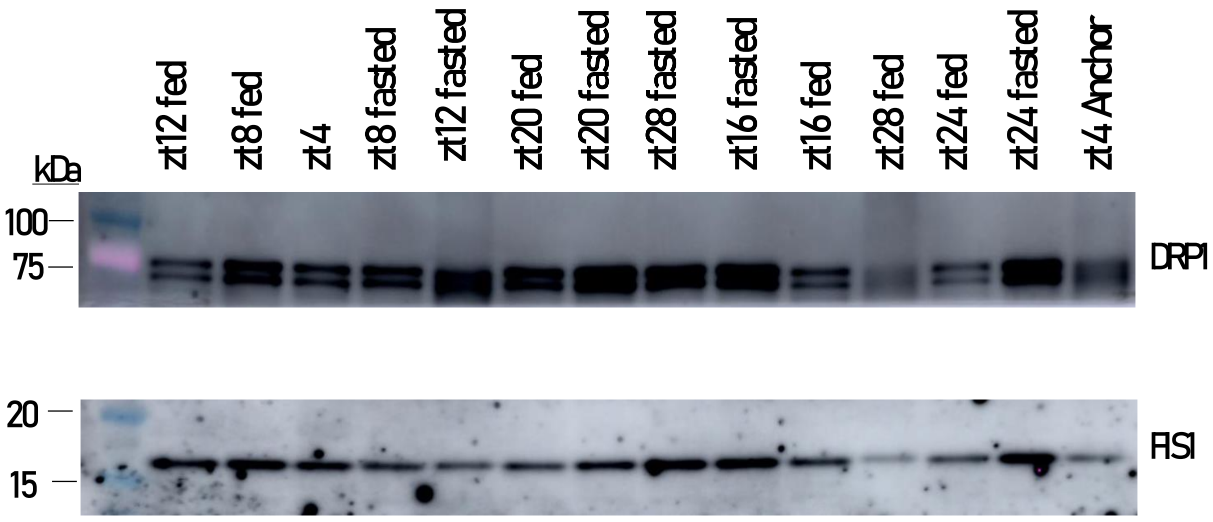
kDa
100
75
20
15
zt12 fed
zt8 fed
zt4
zt8 fasted
zt12 fasted
zt20 fed
zt20 fasted
zt28 fasted
zt16 fasted
zt16 fed
zt28 fed
zt24 fed
zt24 fasted
zt4 Anchor
DRP1
FIS1

Gel 8

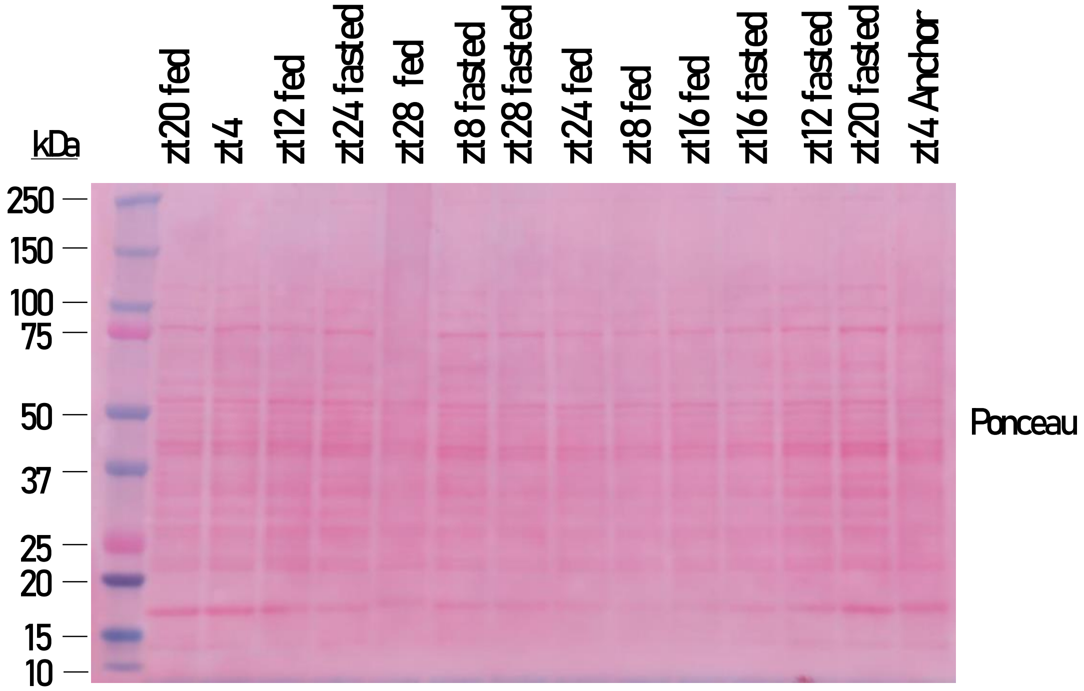
kDa
250
150
100
75
50
37
25
20
15
10
zt20 fed
zt4
zt12 fed
zt24 fasted
zt28 fed
zt8 fasted
zt28 fasted
zt24 fed
zt8 fed
zt16 fed
zt16 fasted
zt12 fasted
zt20 fasted
zt4 Anchor
Ponceau

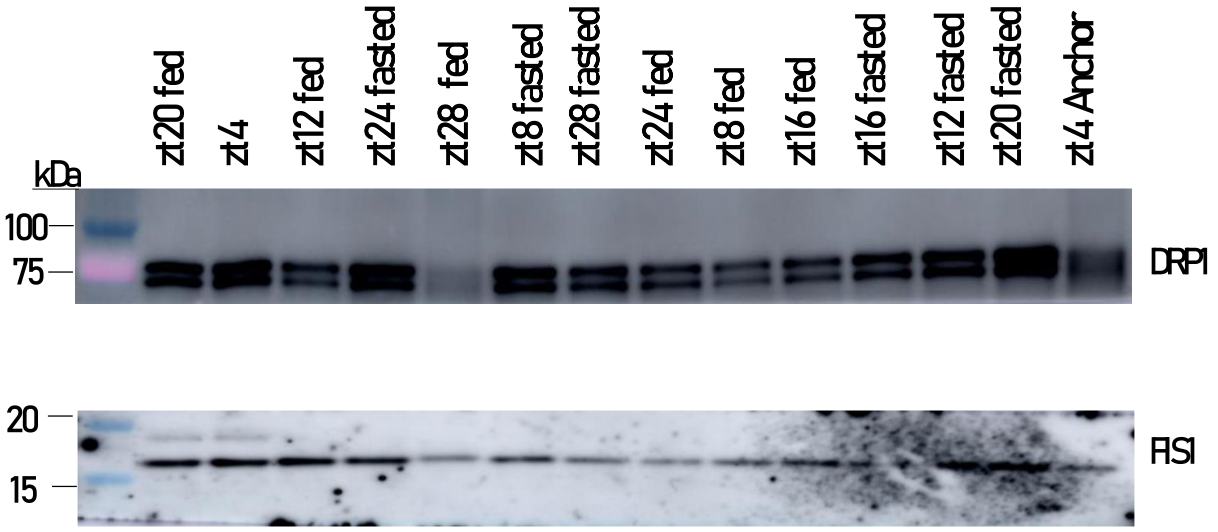
kDa
100
75
20
15
zt20 fed
zt4
zt12 fed
zt24 fasted
zt28 fed
zt8 fasted
zt28 fasted
zt24 fed
zt8 fed
zt16 fed
zt16 fasted
zt12 fasted
zt20 fasted
zt4 Anchor
DRP1
FIS1

Gel 9

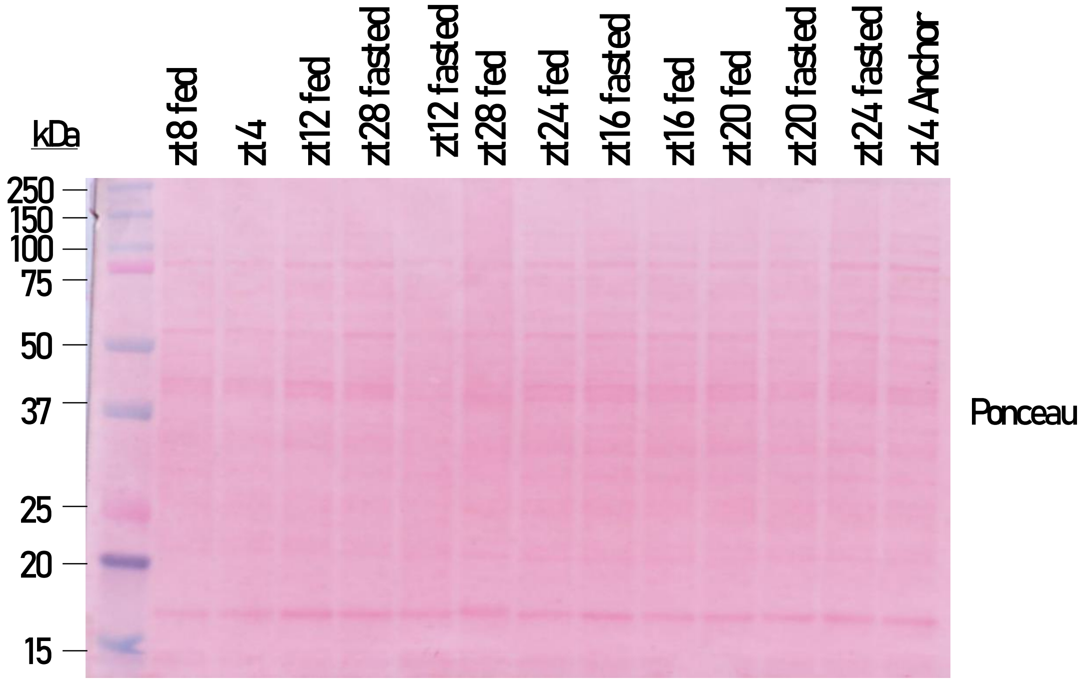

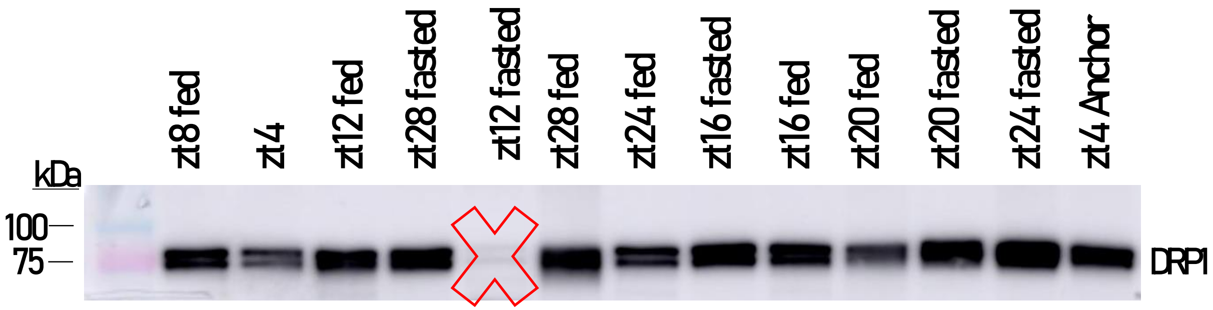

* ZT12 fast, no signal

Gel 10

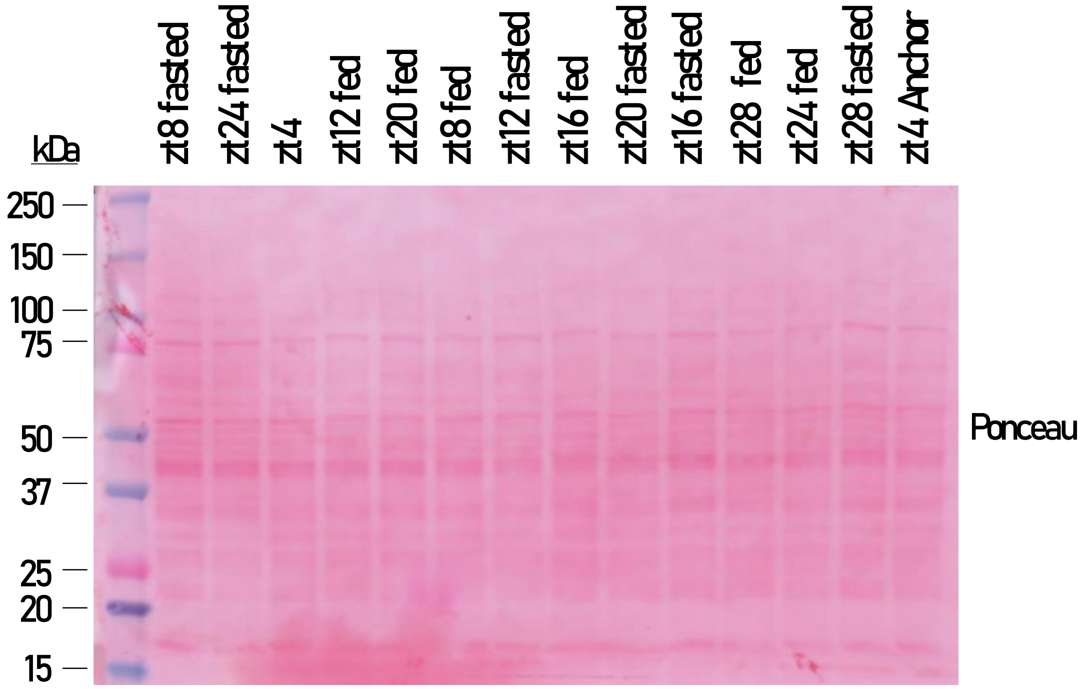
kDa
250
150
100
75
50
37
25
20
15
zt8 fasted
zt24 fasted
zt4
zt12 fed
zt20 fed
zt8 fed
zt12 fasted
zt16 fed
zt20 fasted
zt16 fasted
zt28 fed
zt24 fed
zt28 fasted
zt4 Anchor
Ponceau

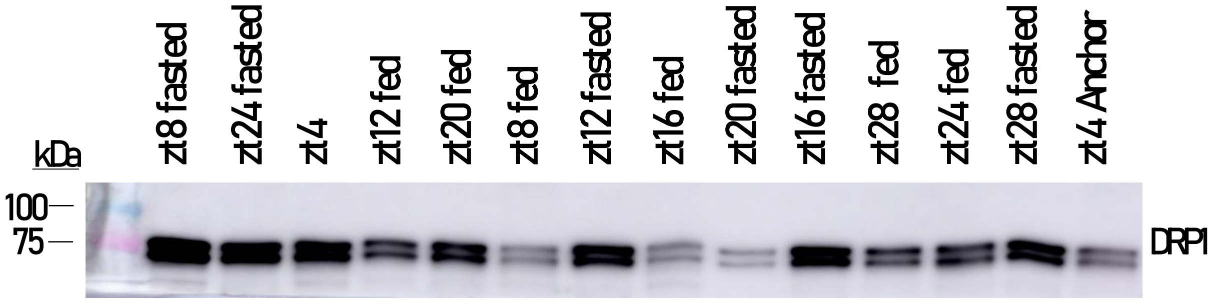
kDa
100
75
zt8 fasted
zt24 fasted
zt4
zt12 fed
zt20 fed
zt8 fed
zt12 fasted
zt16 fed
zt20 fasted
zt16 fasted
zt28 fed
zt24 fed
zt28 fasted
zt4 Anchor
DRP1

Gel 11

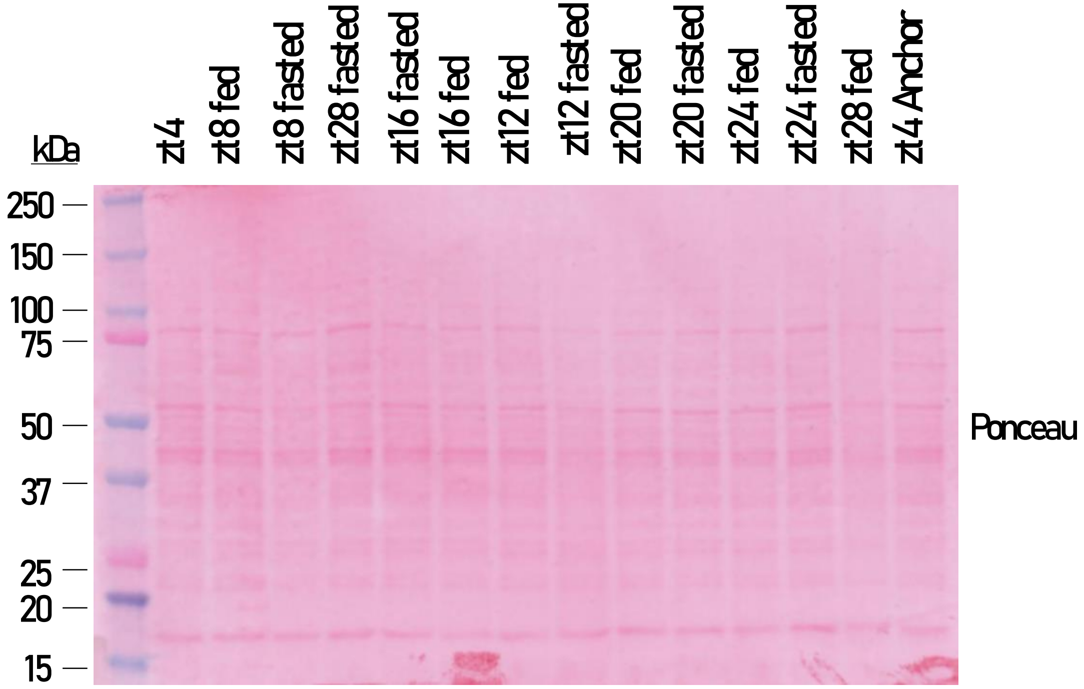
zt4
zt8 fed
zt8 fasted
zt28 fasted
zt16 fasted
zt16 fed
zt12 fed
zt12 fasted
zt20 fed
zt20 fasted
zt24 fed
zt24 fasted
zt28 fed
zt4 Anchor
kDa
250
150
100
75
50
37
25
20
15
Ponceau

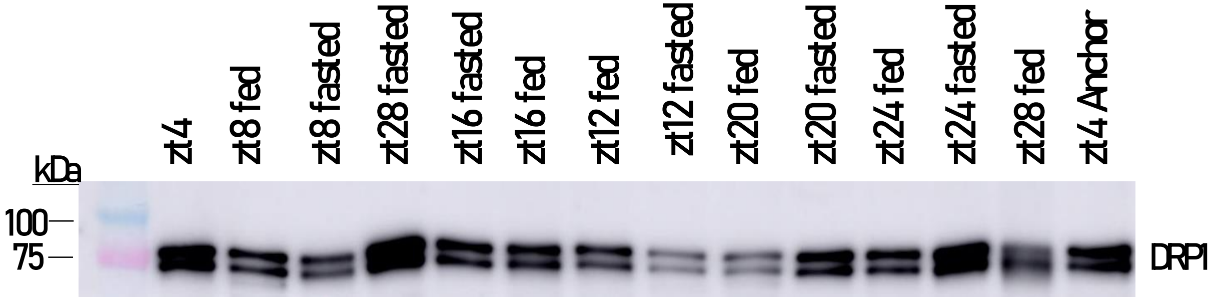
zt4
zt8 fed
zt8 fasted
zt28 fasted
zt16 fasted
zt16 fed
zt12 fed
zt12 fasted
zt20 fed
zt20 fasted
zt24 fed
zt24 fasted
zt28 fed
zt4 Anchor
kDa
100
75
DRP1

Gel 12

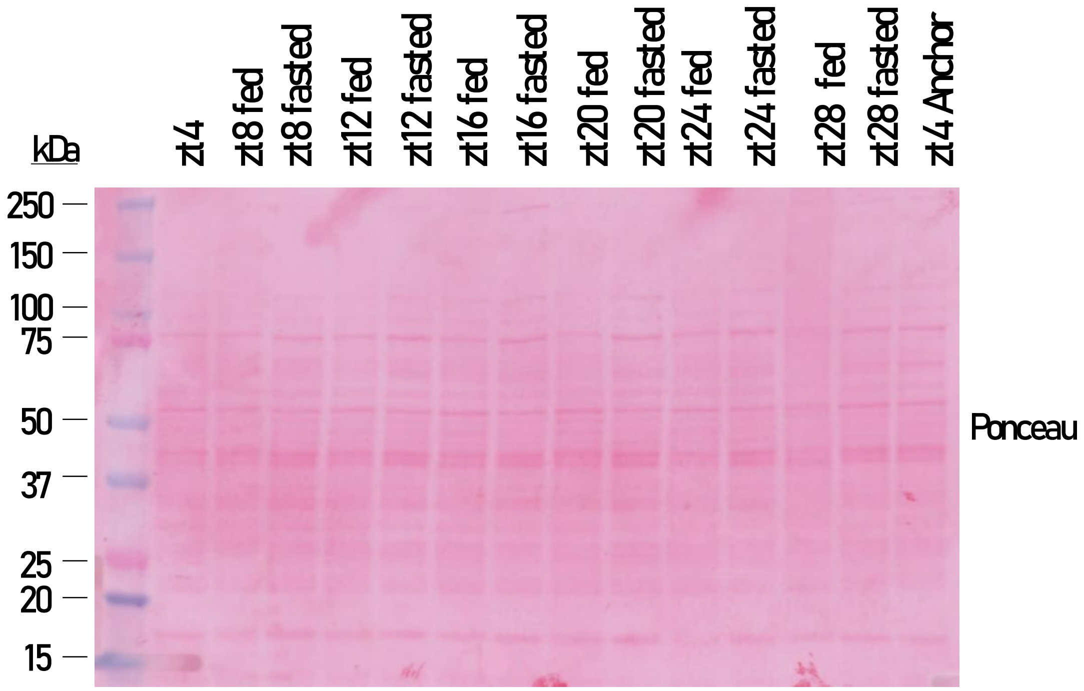

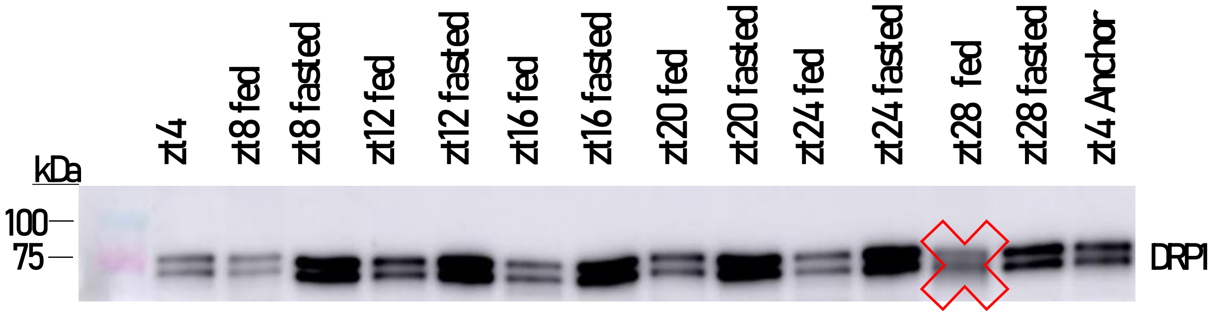

*ZT28 excluded, sample issue (smear in ponceau)

Week 5

Complex I(NDUFA9; Invitrogen, 459100), III (Core 1; Invitrogen, 459140)
September 8-13, 2021

Gel 1

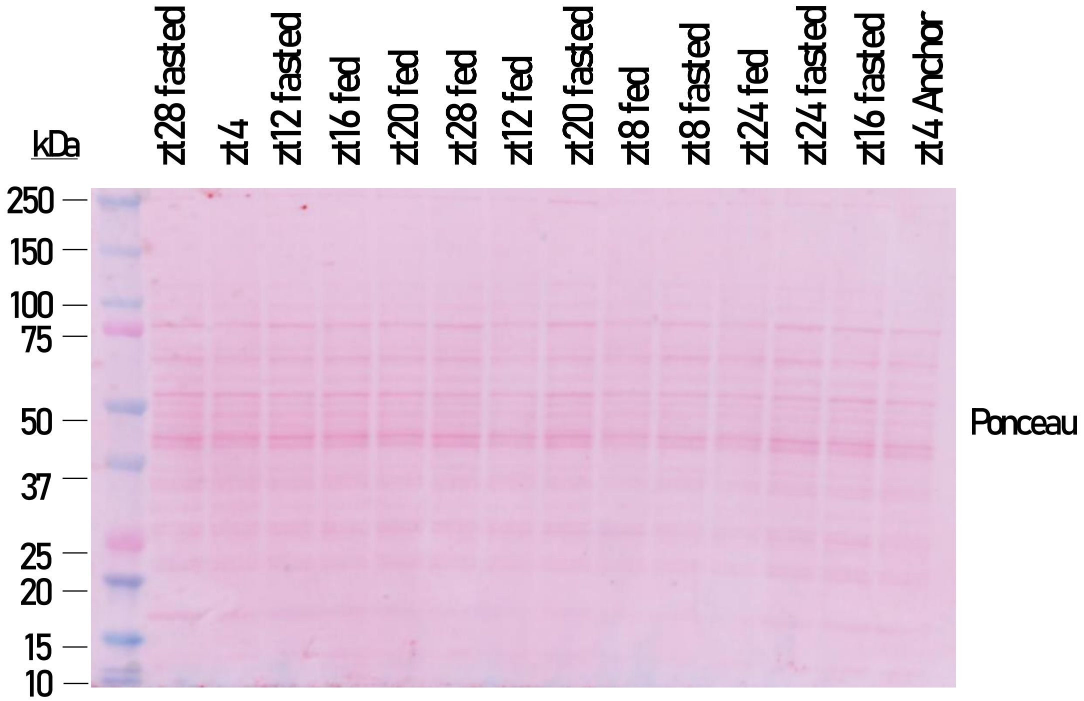
kDa
250
150
100
75
50
37
25
20
15
10
zt28 fasted
zt4
zt12 fasted
zt16 fed
zt20 fed
zt28 fed
zt12 fed
zt20 fasted
zt8 fed
zt8 fasted
zt24 fed
zt24 fasted
zt16 fasted
zt4 Anchor
Ponceau

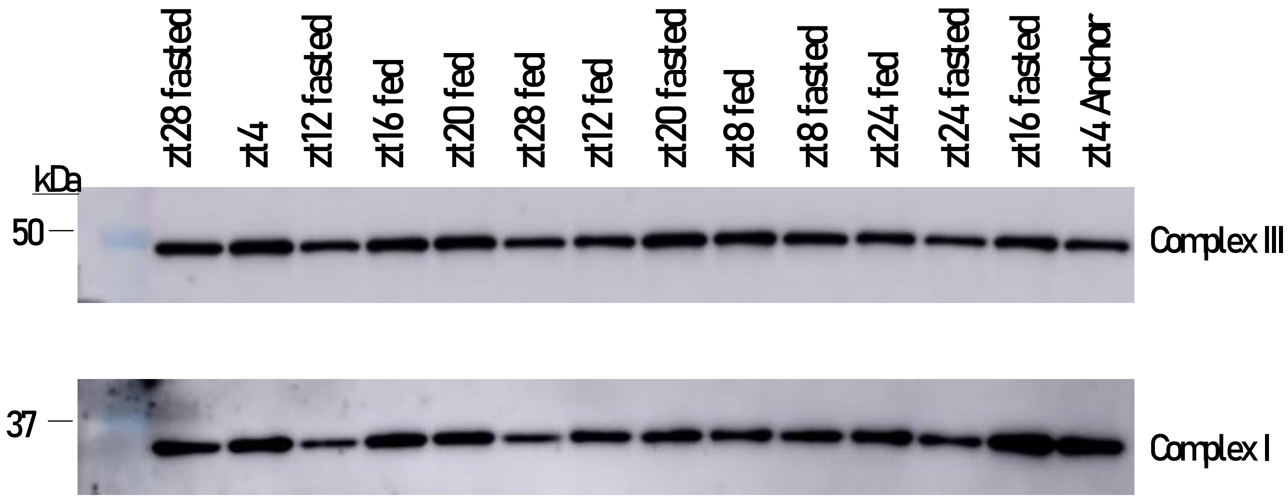
kDa
50
37
zt28 fasted
zt4
zt12 fasted
zt16 fed
zt20 fed
zt28 fed
zt12 fed
zt20 fasted
zt8 fed
zt8 fasted
zt24 fed
zt24 fasted
zt16 fasted
zt4 Anchor
Complex III
Complex I

Gel 2

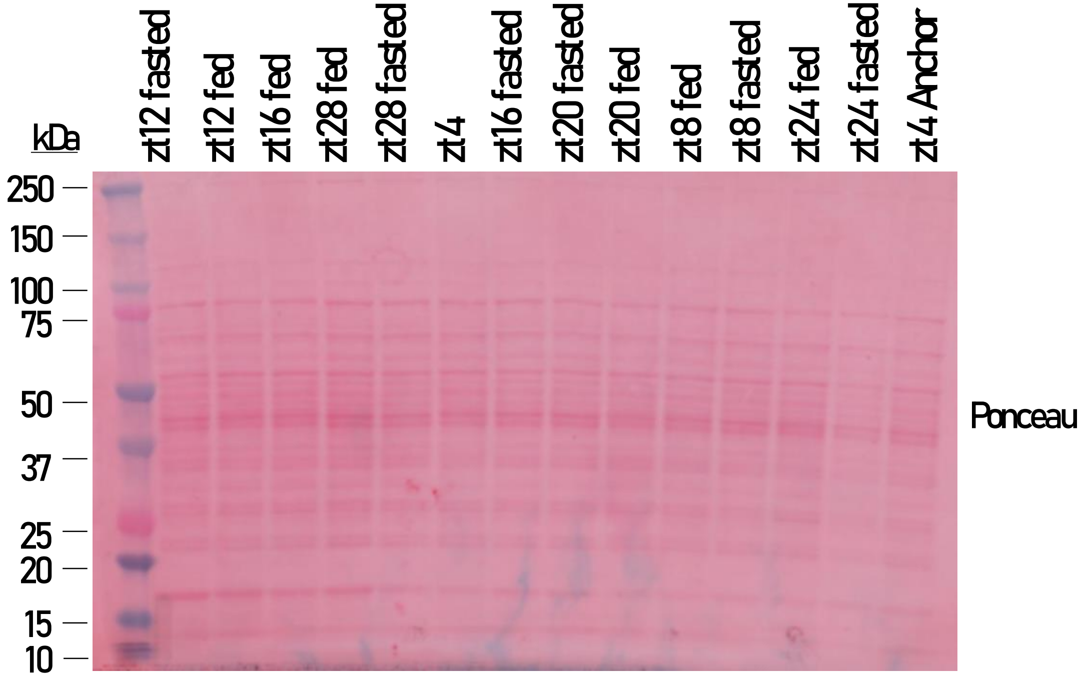

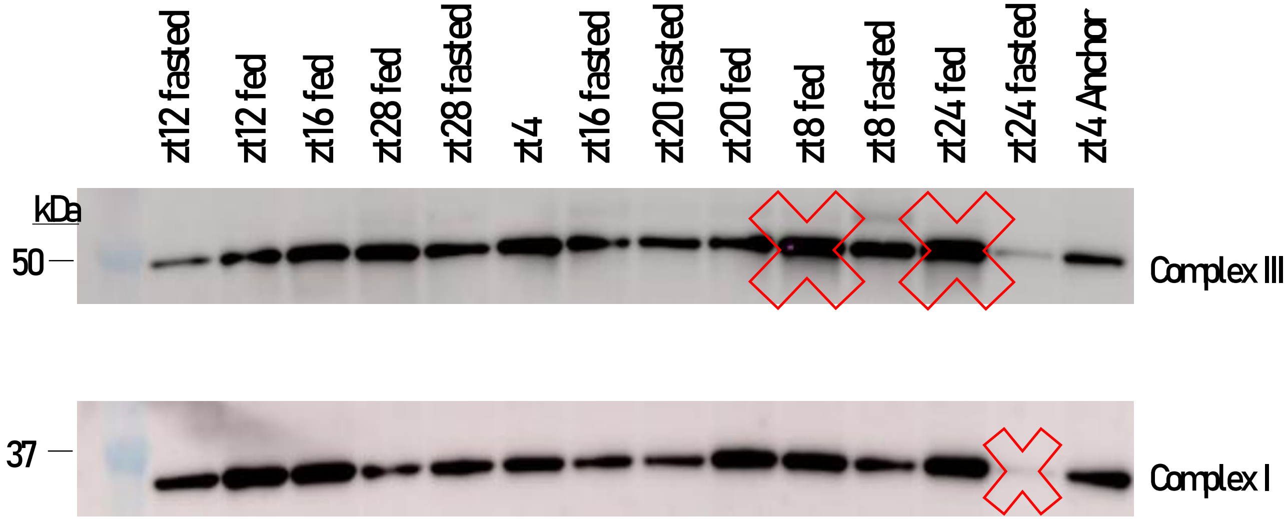

*Complex 1 ZT24 fast excluded, Signal within background signal

*Complex 3 Zt8 and ZT24 fed excluded, saturation of signal (see purple dot)

Gel 3

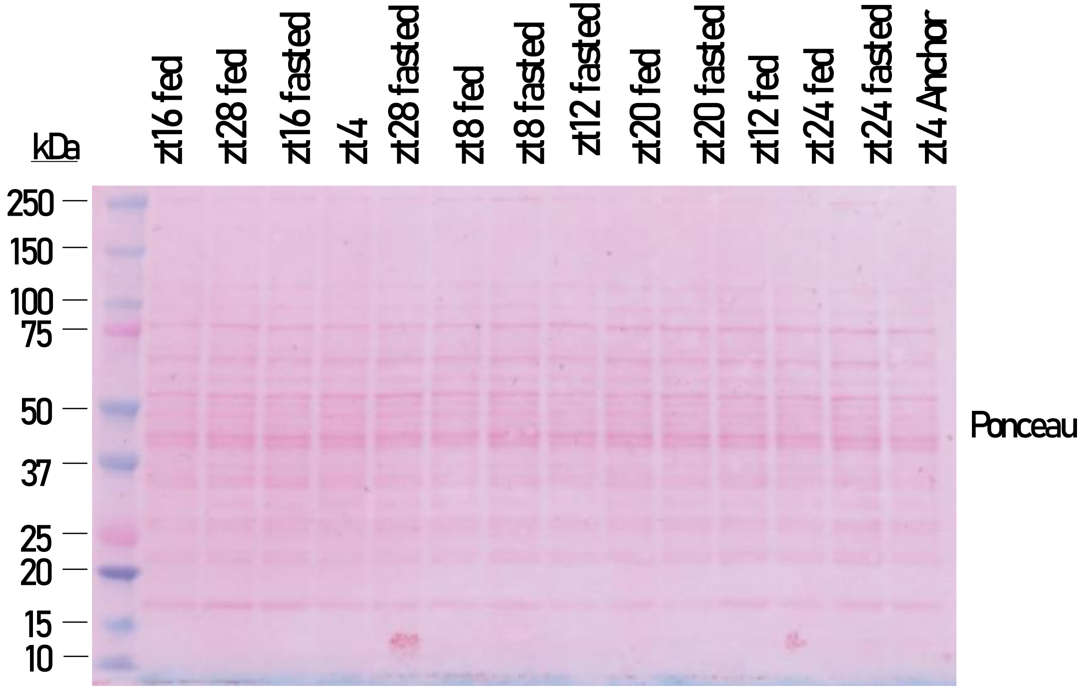
zt16 fed
zt28 fed
zt16 fasted
zt4
zt28 fasted
zt8 fed
zt8 fasted
zt12 fasted
zt20 fed
zt20 fasted
zt12 fed
zt24 fed
zt24 fasted
zt4 Anchor
kDa
250
150
100
75
50
37
25
20
15
10
Ponceau

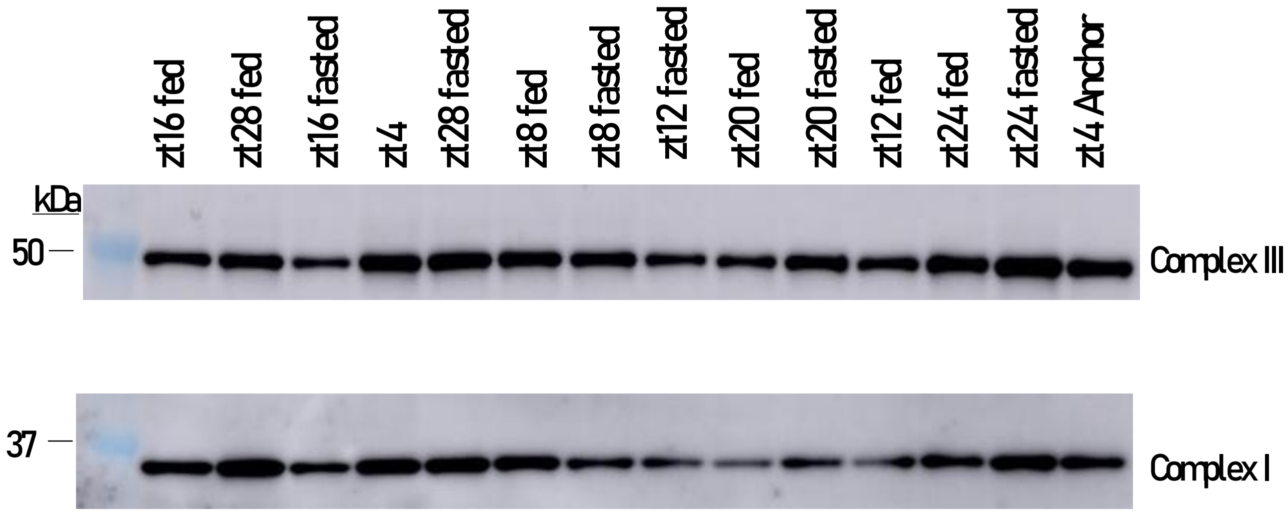
zt16 fed
zt28 fed
zt16 fasted
zt4
zt28 fasted
zt8 fed
zt8 fasted
zt12 fasted
zt20 fed
zt20 fasted
zt12 fed
zt24 fed
zt24 fasted
zt4 Anchor
kDa
50
Complex III
37
Complex I

Gel 4

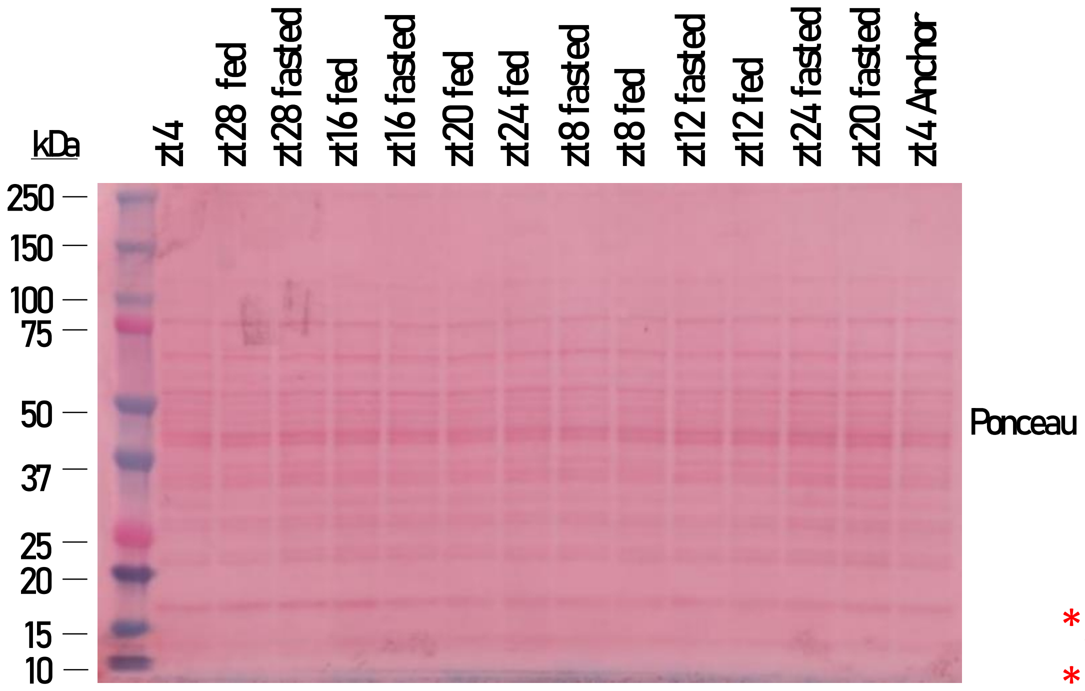

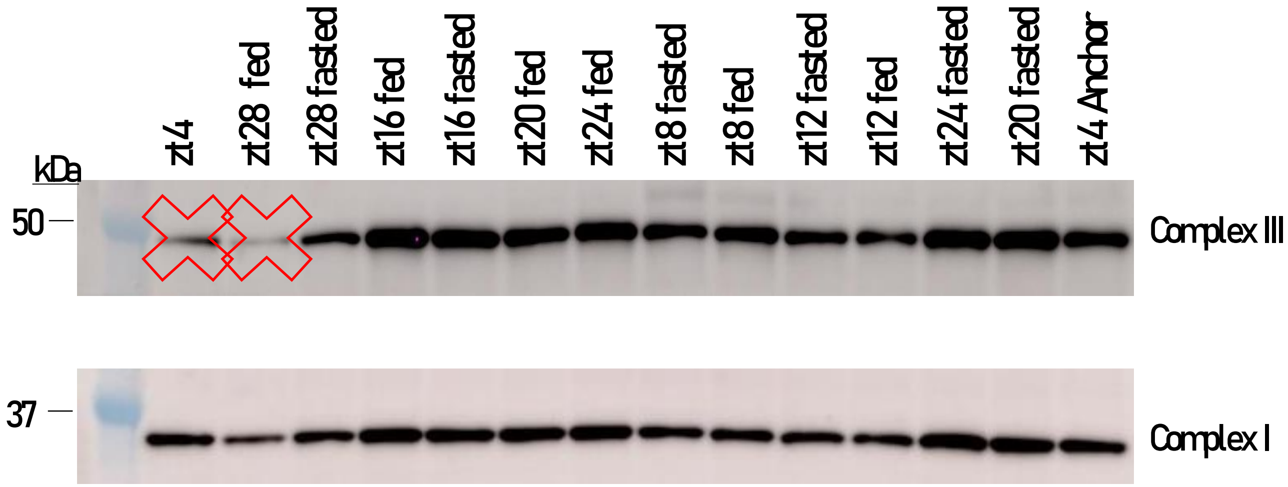

*Zt4 fed excluded, technical issue (signal heterogeneous across band)
*Zt28 fed excluded, signal in background noise signal

Gel 5

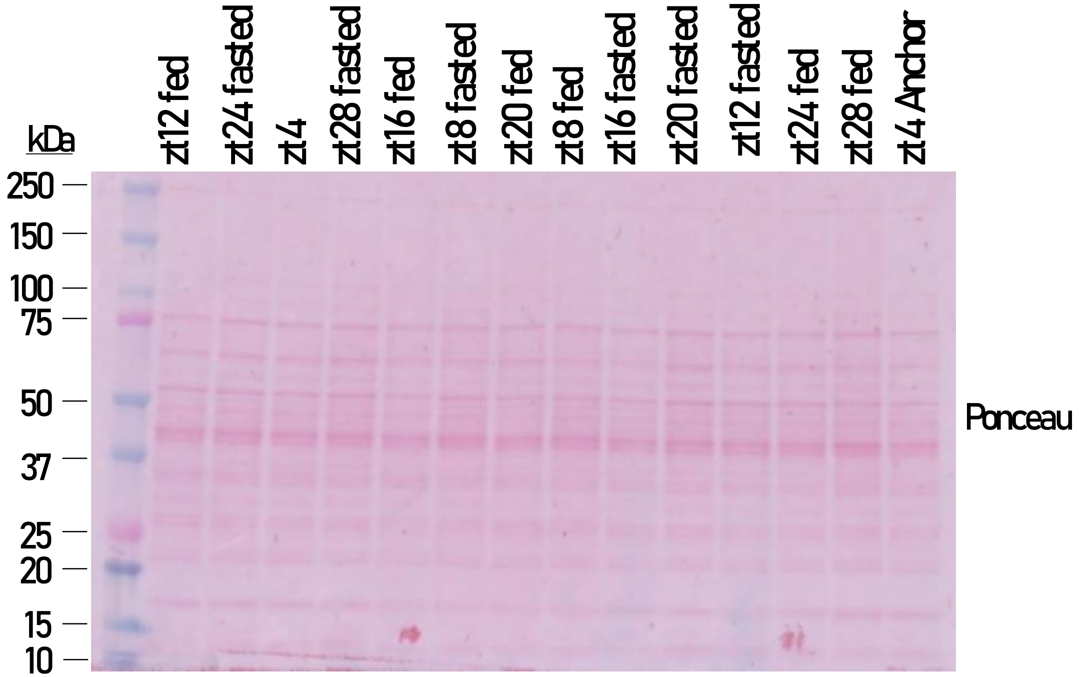
zt12 fed
zt24 fasted
zt4
zt28 fasted
zt16 fed
zt8 fasted
zt20 fed
zt8 fed
zt16 fasted
zt20 fasted
zt12 fasted
zt24 fed
zt28 fed
zt4 Anchor
kDa
250
150
100
75
50
37
25
20
15
10
Ponceau

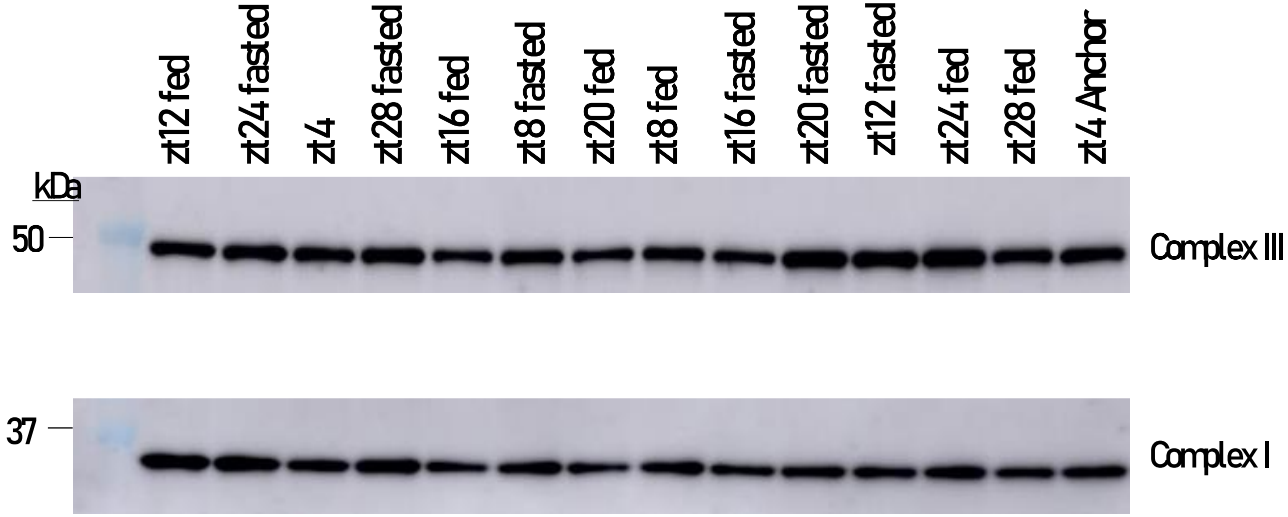
zt12 fed
zt24 fasted
zt4
zt28 fasted
zt16 fed
zt8 fasted
zt20 fed
zt8 fed
zt16 fasted
zt20 fasted
zt12 fasted
zt24 fed
zt28 fed
zt4 Anchor
kDa
50
Complex III
37
Complex I

Gel 6

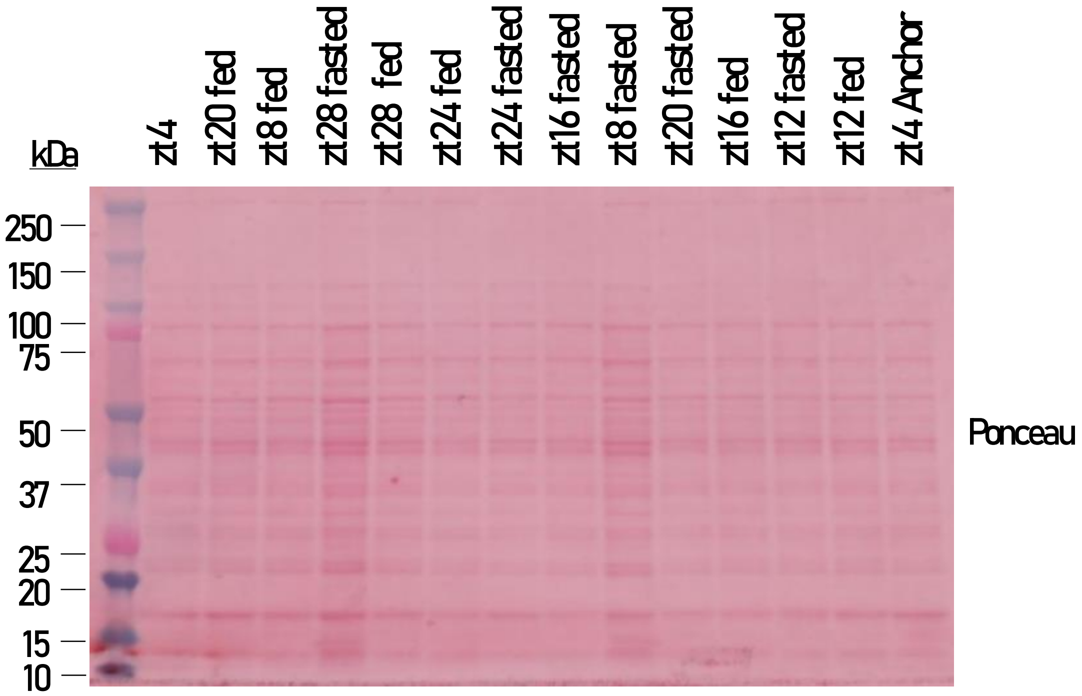
kDa
250
150
100
75
50
37
25
20
15
10
zt4
zt20 fed
zt8 fed
zt28 fasted
zt28 fed
zt24 fed
zt24 fasted
zt16 fasted
zt8 fasted
zt20 fasted
zt16 fed
zt12 fasted
zt12 fed
zt4 Anchor
Ponceau

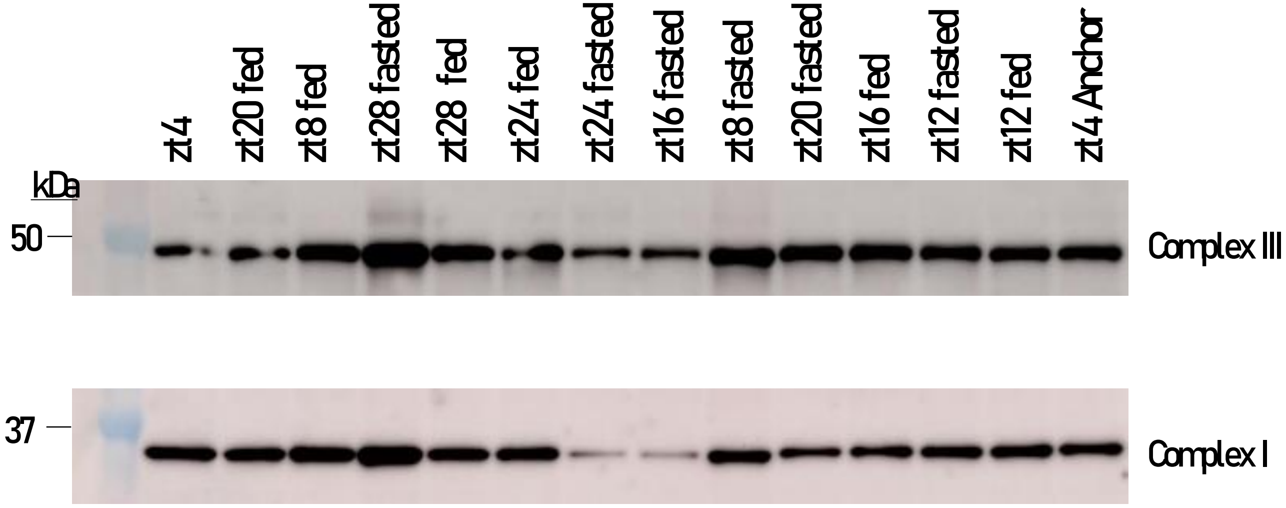
kDa
50
37
zt4
zt20 fed
zt8 fed
zt28 fasted
zt28 fed
zt24 fed
zt24 fasted
zt16 fasted
zt8 fasted
zt20 fasted
zt16 fed
zt12 fasted
zt12 fed
zt4 Anchor
Complex III
Complex I

Gel 7

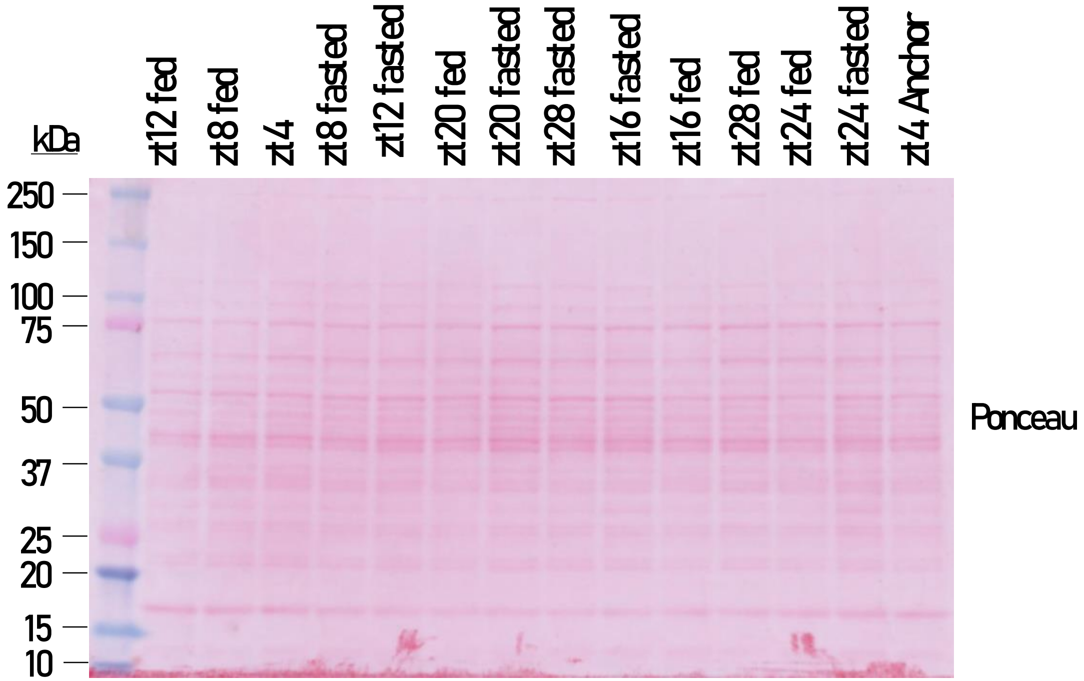
kDa
250
150
100
75
50
37
25
20
15
10
zt12 fed
zt8 fed
zt4
zt8 fasted
zt12 fasted
zt20 fed
zt20 fasted
zt28 fasted
zt16 fasted
zt16 fed
zt28 fed
zt24 fed
zt24 fasted
zt4 Anchor
Ponceau

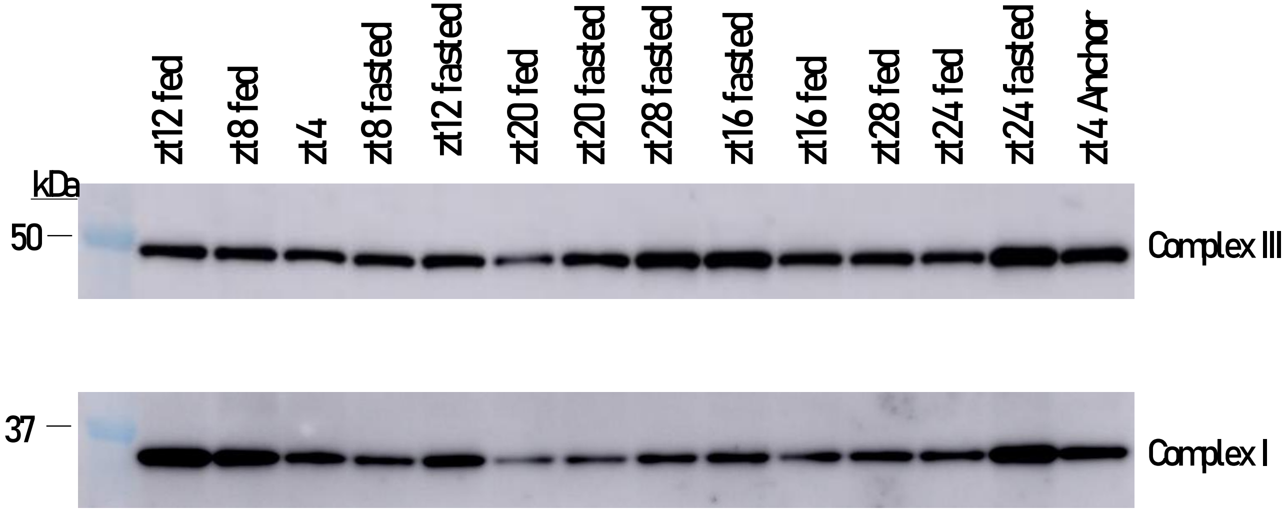
kDa
50
37
zt12 fed
zt8 fed
zt4
zt8 fasted
zt12 fasted
zt20 fed
zt20 fasted
zt28 fasted
zt16 fasted
zt16 fed
zt28 fed
zt24 fed
zt24 fasted
zt4 Anchor
Complex III
Complex I

Gel 8

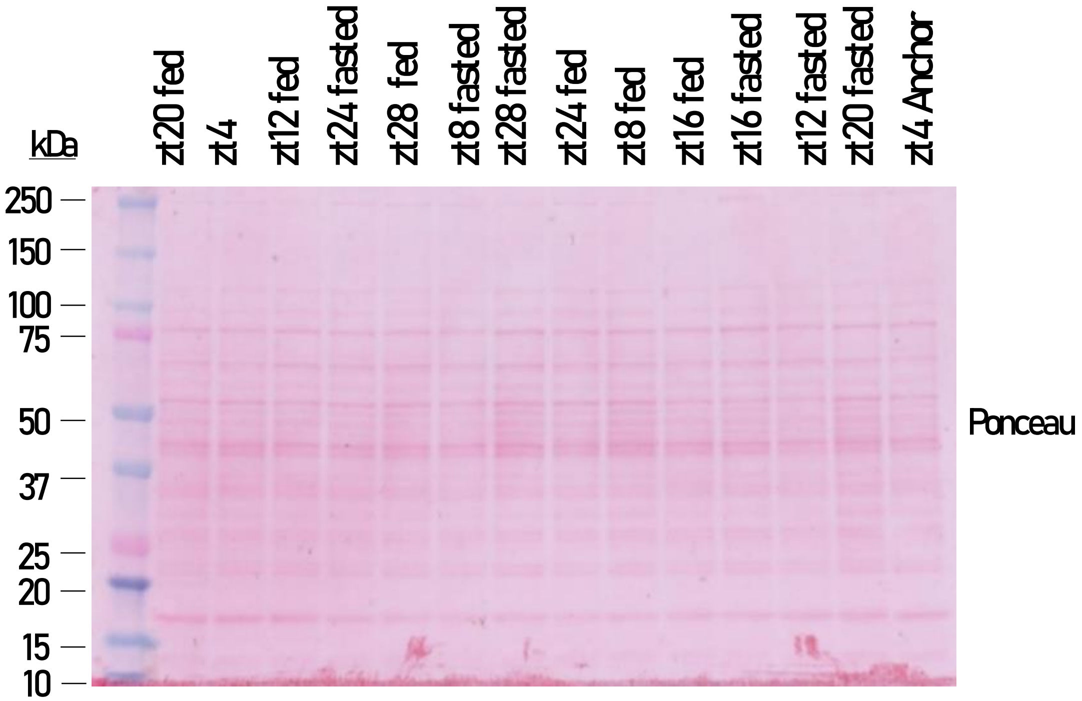
kDa
250
150
100
75
50
37
25
20
15
10
zt20 fed
zt4
zt12 fed
zt24 fasted
zt28 fed
zt8 fasted
zt28 fasted
zt24 fed
zt8 fed
zt16 fed
zt16 fasted
zt12 fasted
zt20 fasted
zt4 Anchor
Ponceau

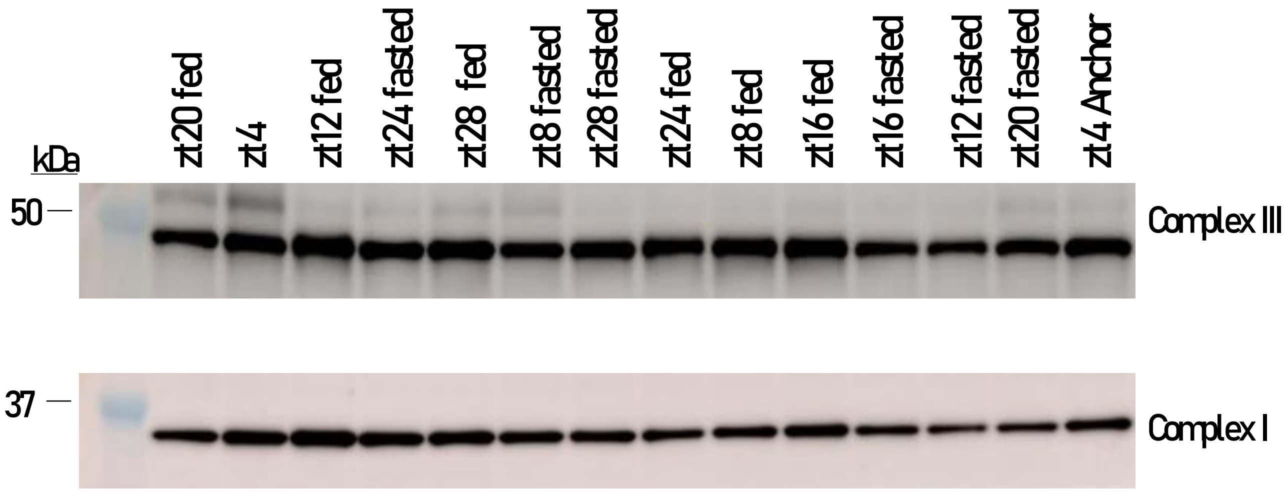
kDa
50
37
zt20 fed
zt4
zt12 fed
zt24 fasted
zt28 fed
zt8 fasted
zt28 fasted
zt24 fed
zt8 fed
zt16 fed
zt16 fasted
zt12 fasted
zt20 fasted
zt4 Anchor
Complex III
Complex I

Gel 9

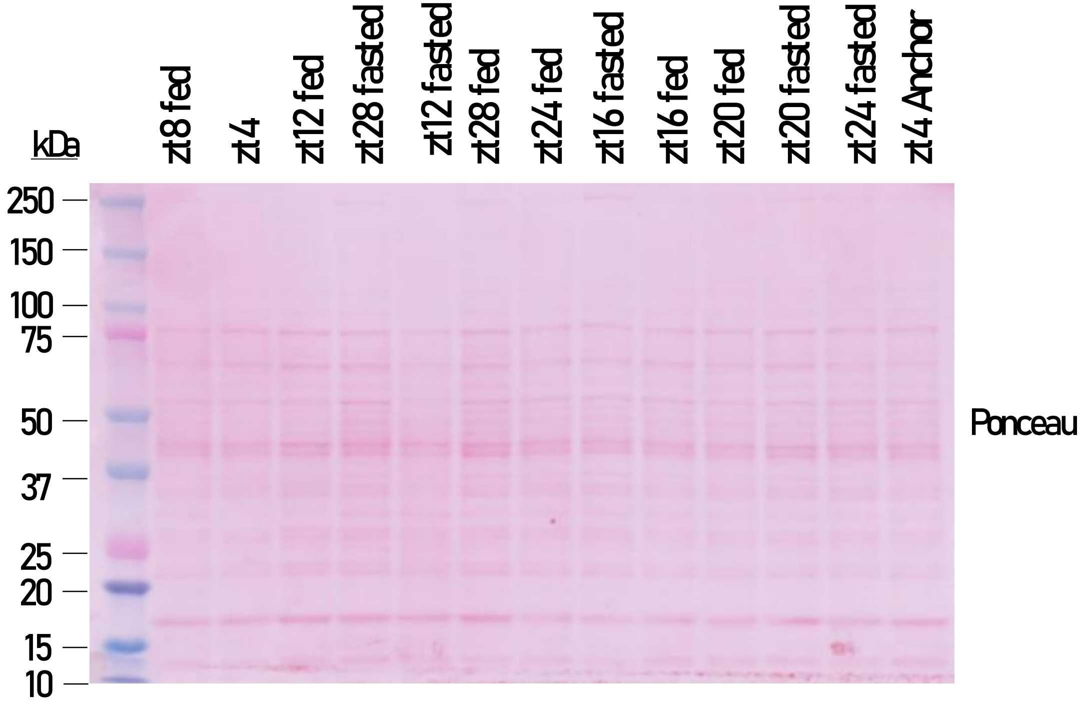

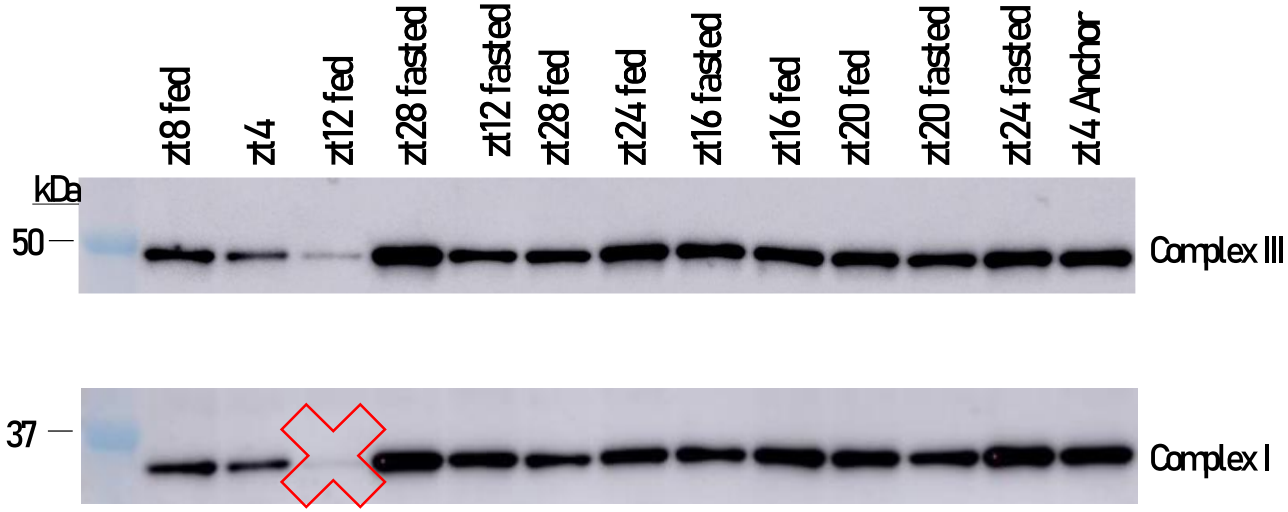

*Zt12 Fed excluded from analysis, signal was within background signal

Gel 10

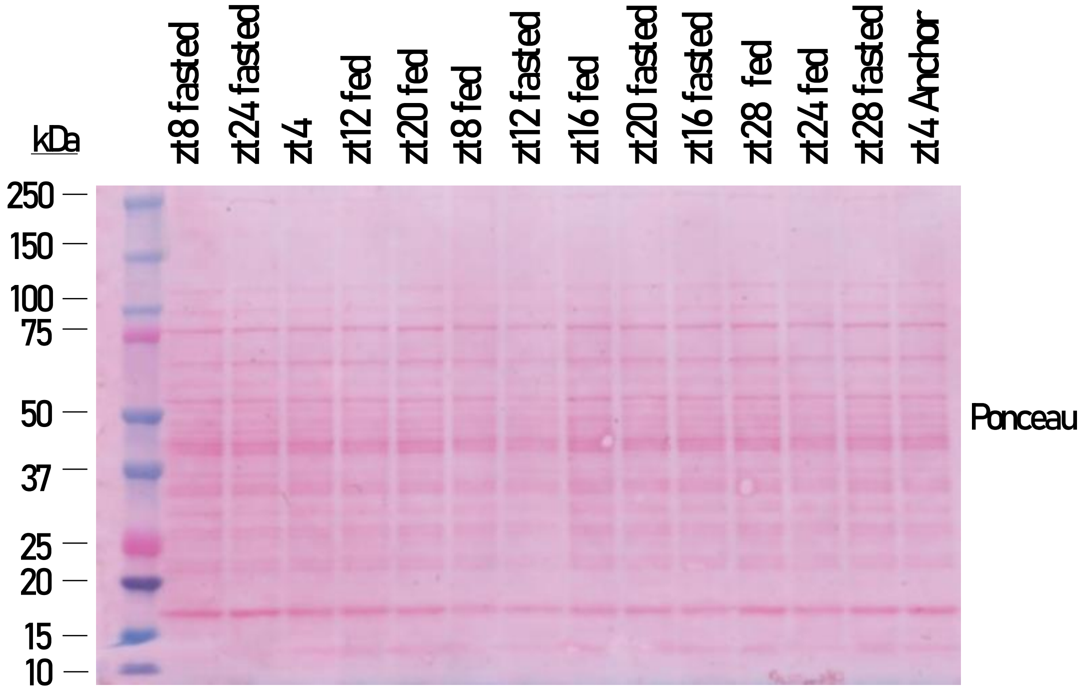

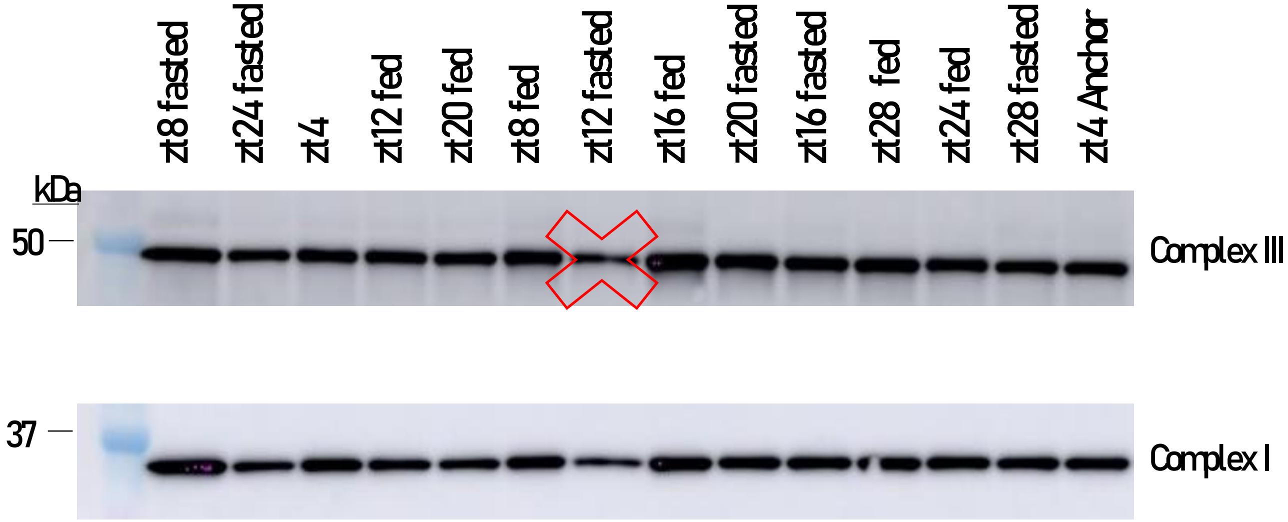

*ZT12 fasted excluded, signal heterogeneous (cut in middle)

Gel 11

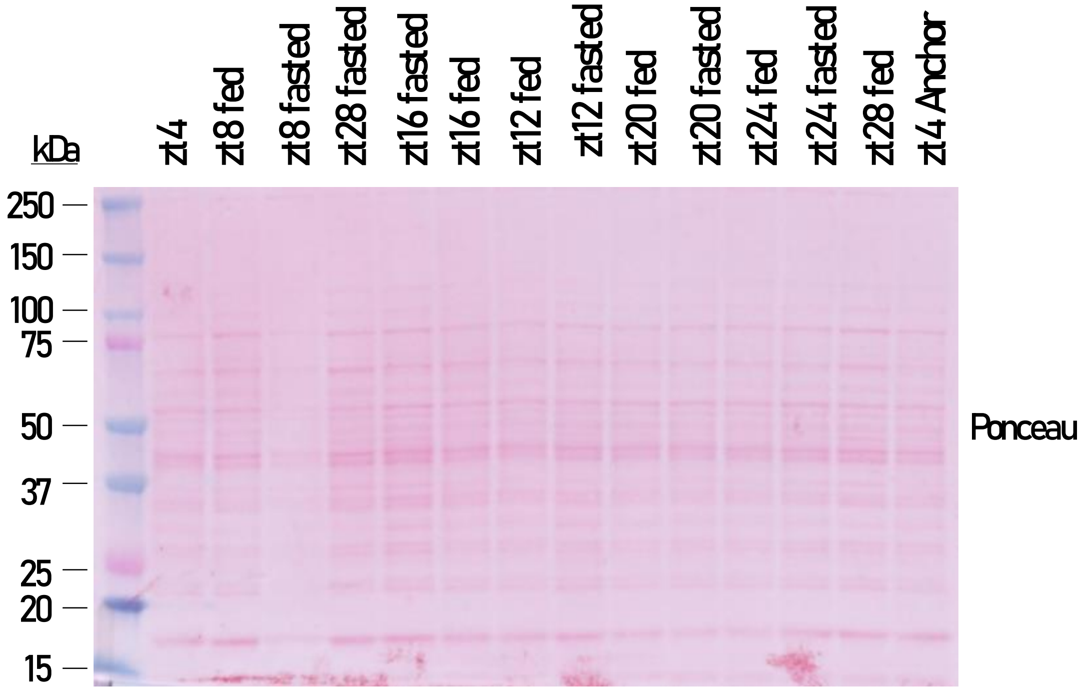
kDa
250
150
100
75
50
37
25
20
15
zt4
zt8 fed
zt8 fasted
zt28 fasted
zt16 fasted
zt16 fed
zt12 fed
zt12 fasted
zt20 fed
zt20 fasted
zt24 fed
zt24 fasted
zt28 fed
zt4 Anchor
Ponceau

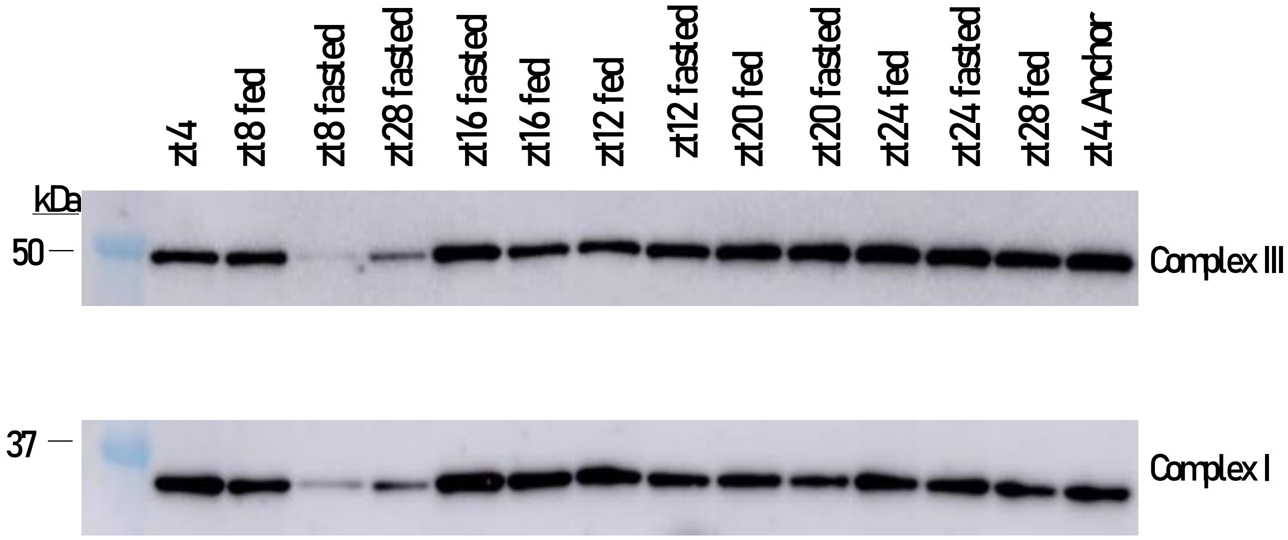
kDa
50
37
zt4
zt8 fed
zt8 fasted
zt28 fasted
zt16 fasted
zt16 fed
zt12 fed
zt12 fasted
zt20 fed
zt20 fasted
zt24 fed
zt24 fasted
zt28 fed
zt4 Anchor
Complex III
Complex I

Gel 12

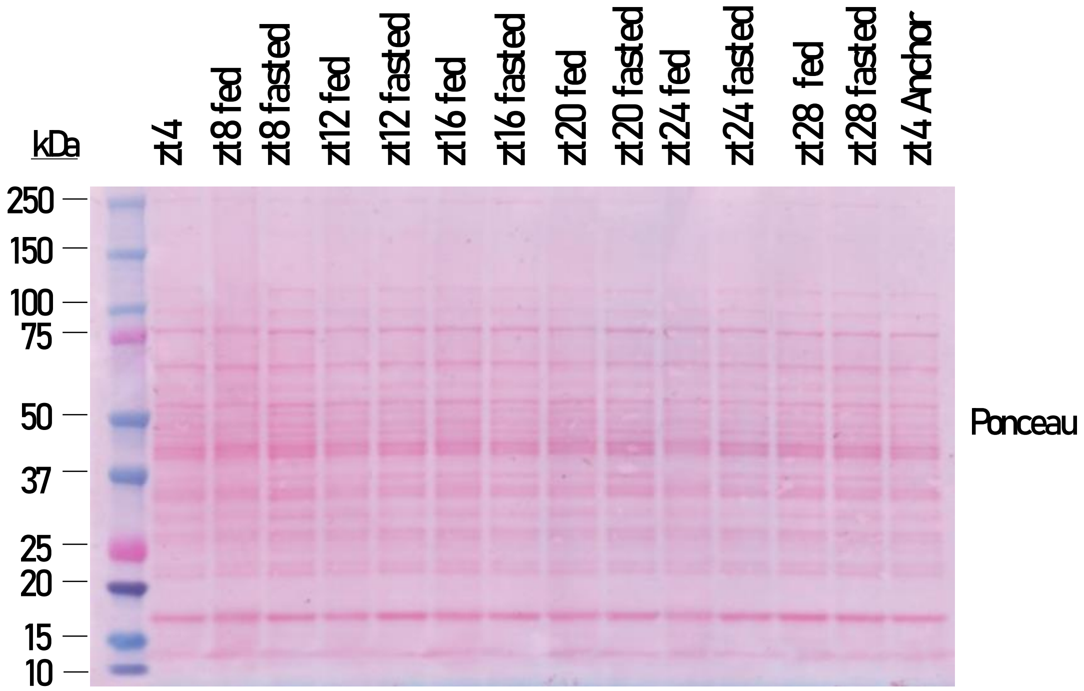

kDa
250
150
100
75
50
37
25
20
15
10
zt4
zt8 fed
zt8 fasted
zt12 fed
zt12 fasted
zt16 fed
zt16 fasted
zt20 fed
zt20 fasted
zt24 fed
zt24 fasted
zt28 fed
zt28 fasted
zt4 Anchor
Ponceau

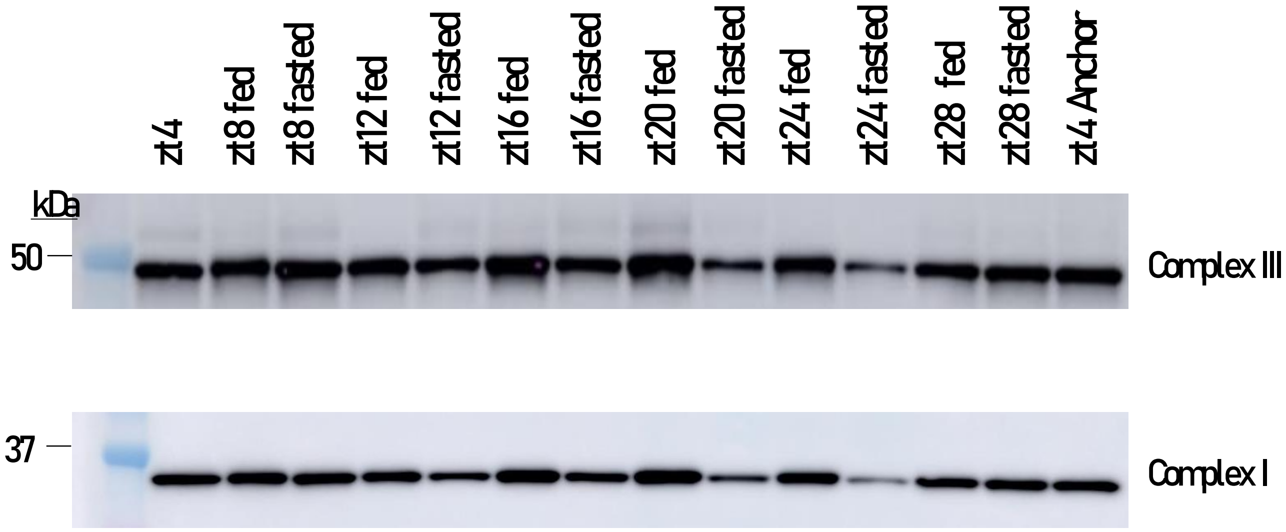

zt4
zt8 fed
zt8 fasted
zt12 fed
zt12 fasted
zt16 fed
zt16 fasted
zt20 fed
zt20 fasted
zt24 fed
zt24 fasted
zt28 fed
zt28 fasted
zt4 Anchor
kDa
50
37
Complex III
Complex I

Gel 12

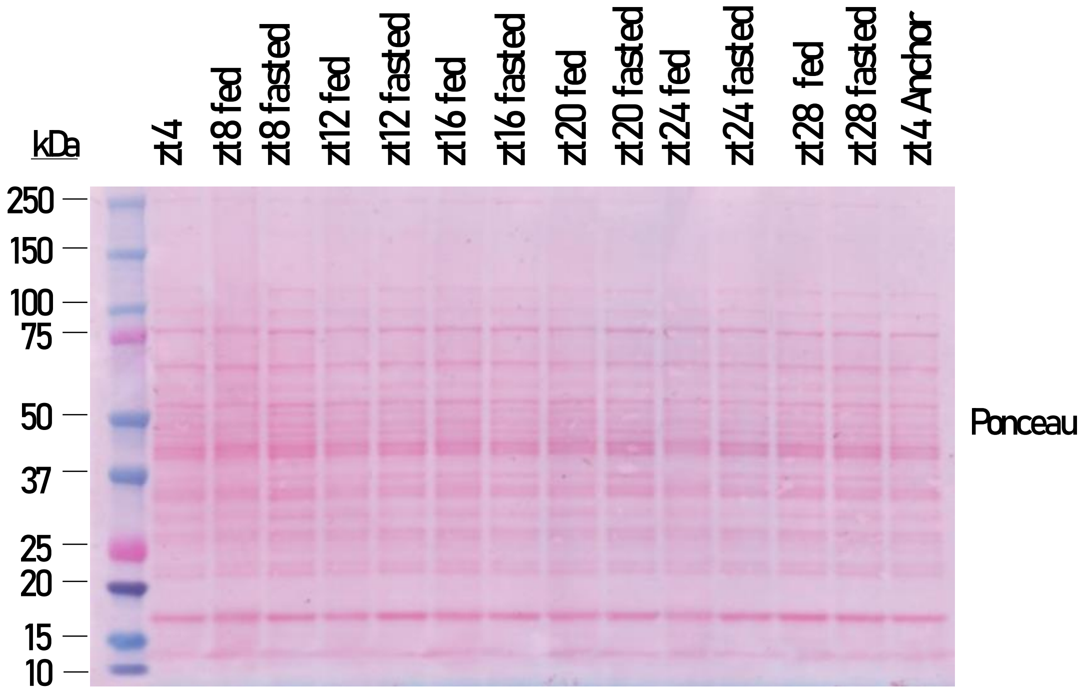
kDa
250
150
100
75
50
37
25
20
15
10
zt4
zt8 fed
zt8 fasted
zt12 fed
zt12 fasted
zt16 fed
zt16 fasted
zt20 fed
zt20 fasted
zt24 fed
zt24 fasted
zt28 fed
zt28 fasted
zt4 Anchor
Ponceau

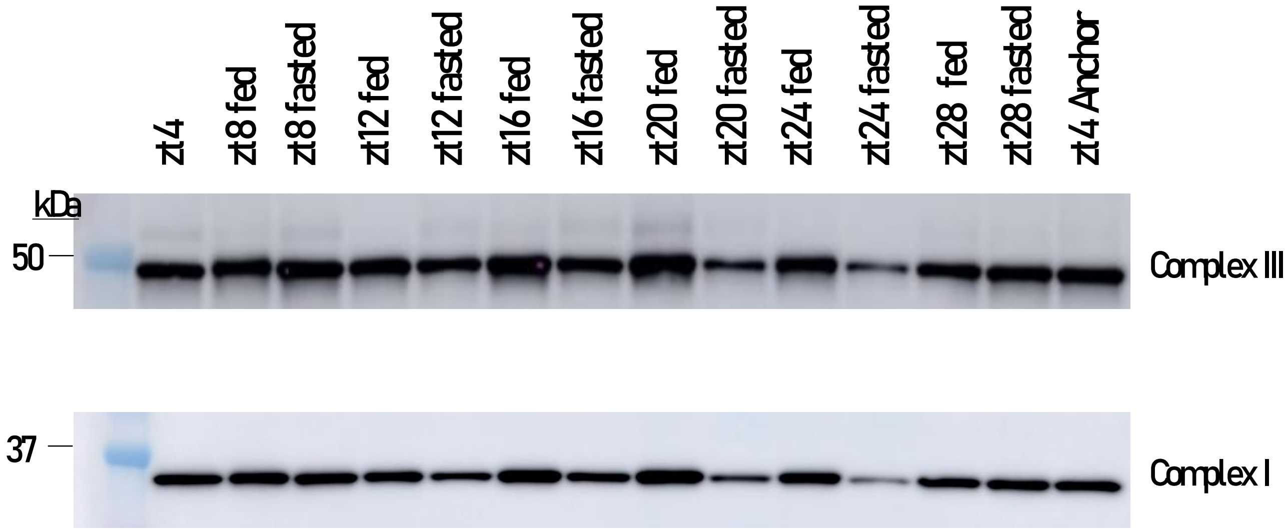
zt4
zt8 fed
zt8 fasted
zt12 fed
zt12 fasted
zt16 fed
zt16 fasted
zt20 fed
zt20 fasted
zt24 fed
zt24 fasted
zt28 fed
zt28 fasted
zt4 Anchor
kDa
50
Complex III
37
Complex I

Week 6

Complex V (Abcam, ab14705)
March 1-4, 2021

all odd gel numbers are female and all even gel numbers are male

Gel 1

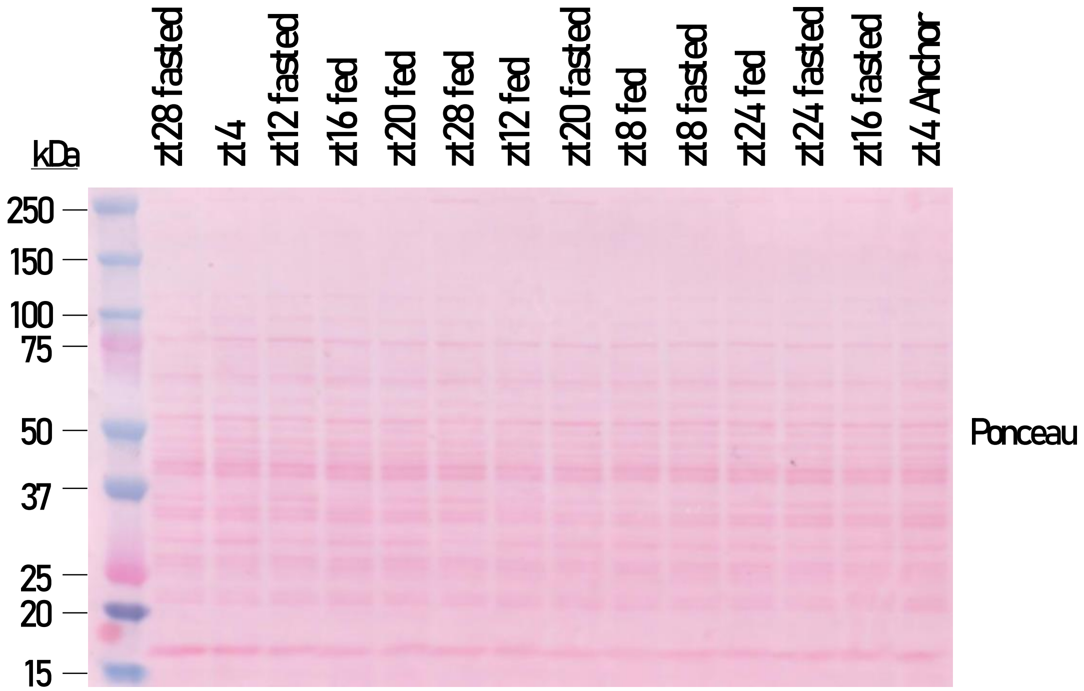

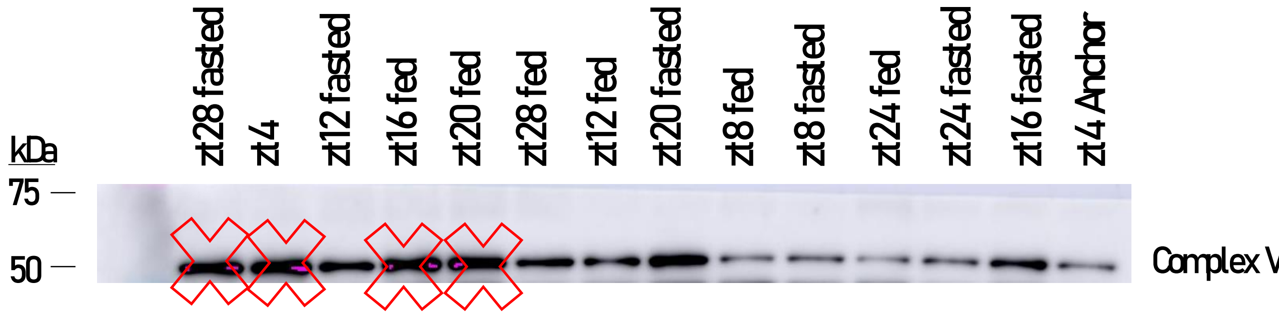

*Complex 5 Zt4,16 and 20 fed and ZT28 fast excluded, saturation of signal (see purple dot)

Gel 2

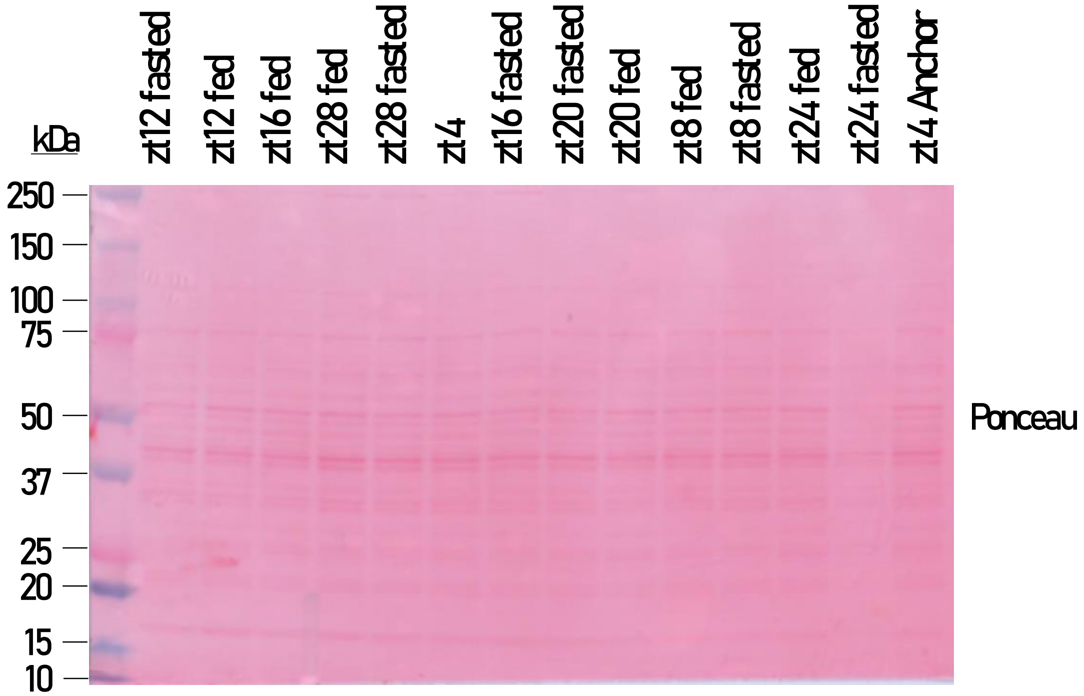
kDa
zt12 fasted
zt12 fed
zt16 fed
zt28 fed
zt28 fasted
zt4
zt16 fasted
zt20 fasted
zt20 fed
zt8 fed
zt8 fasted
zt24 fed
zt24 fasted
zt4 Anchor
250
150
100
75
50
37
25
20
15
10
Ponceau

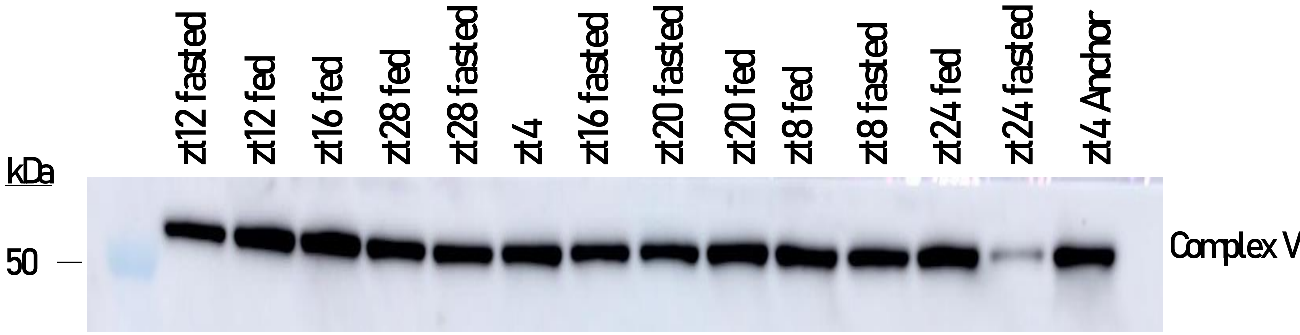
kDa
zt12 fasted
zt12 fed
zt16 fed
zt28 fed
zt28 fasted
zt4
zt16 fasted
zt20 fasted
zt20 fed
zt8 fed
zt8 fasted
zt24 fed
zt24 fasted
zt4 Anchor
50
Complex V

Gel 3

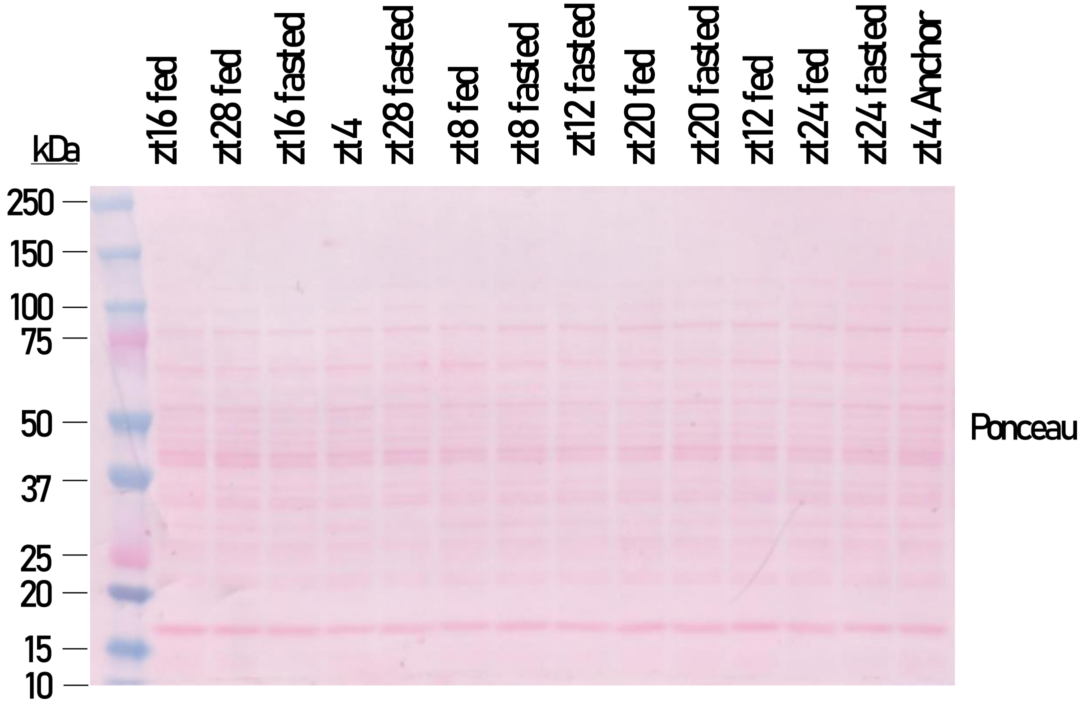

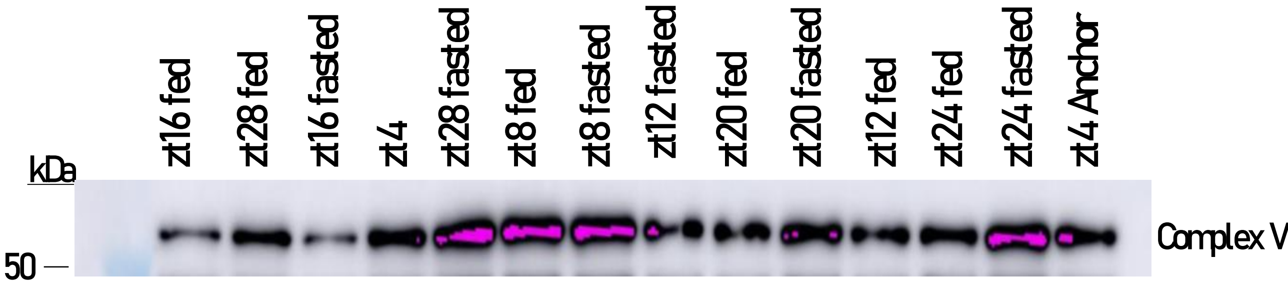

*Complex 5 Zt8 fed Zt8 fast, zt12fast,ZT24 fast excluded, saturation of signal (see purple dot)

Gel 4

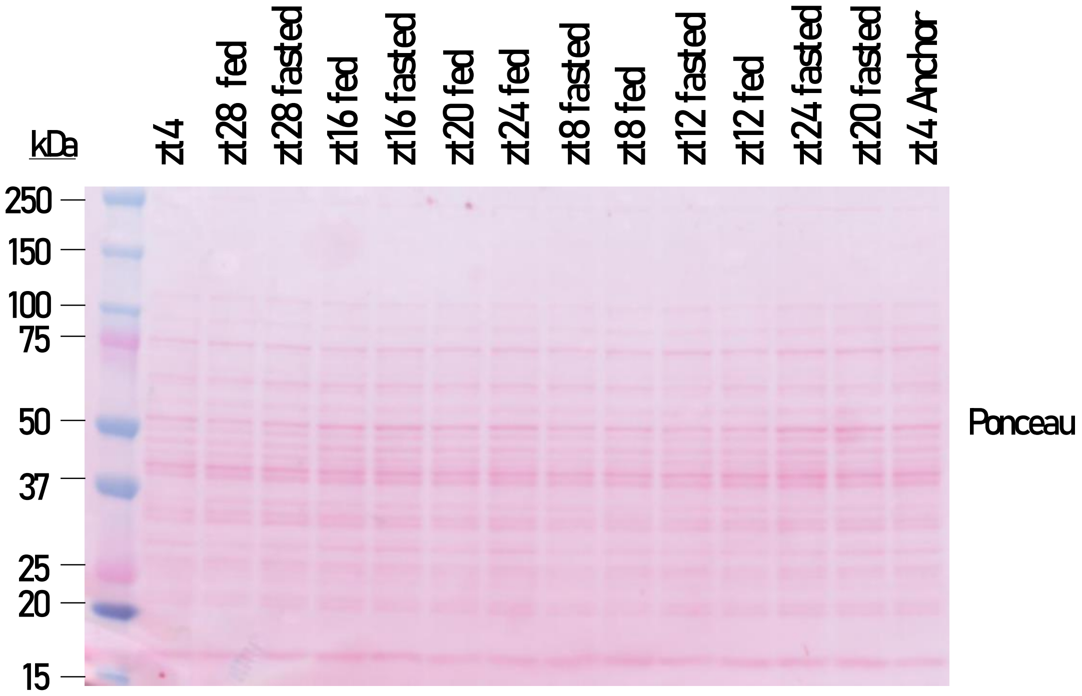
kDa
250
150
100
75
50
37
25
20
15
zt4
zt28 fed
zt28 fasted
zt16 fed
zt16 fasted
zt20 fed
zt24 fed
zt8 fasted
zt8 fed
zt12 fasted
zt12 fed
zt24 fasted
zt20 fasted
zt4 Anchor
Ponceau

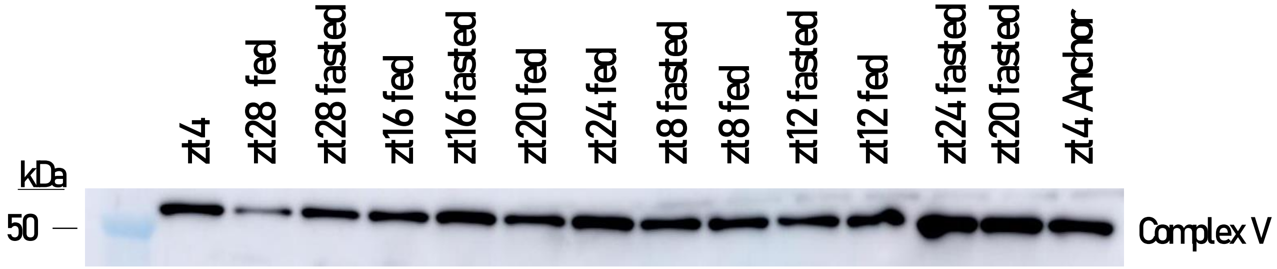
kDa
50
zt4
zt28 fed
zt28 fasted
zt16 fed
zt16 fasted
zt20 fed
zt24 fed
zt8 fasted
zt8 fed
zt12 fasted
zt12 fed
zt24 fasted
zt20 fasted
zt4 Anchor
Complex V

Gel 5

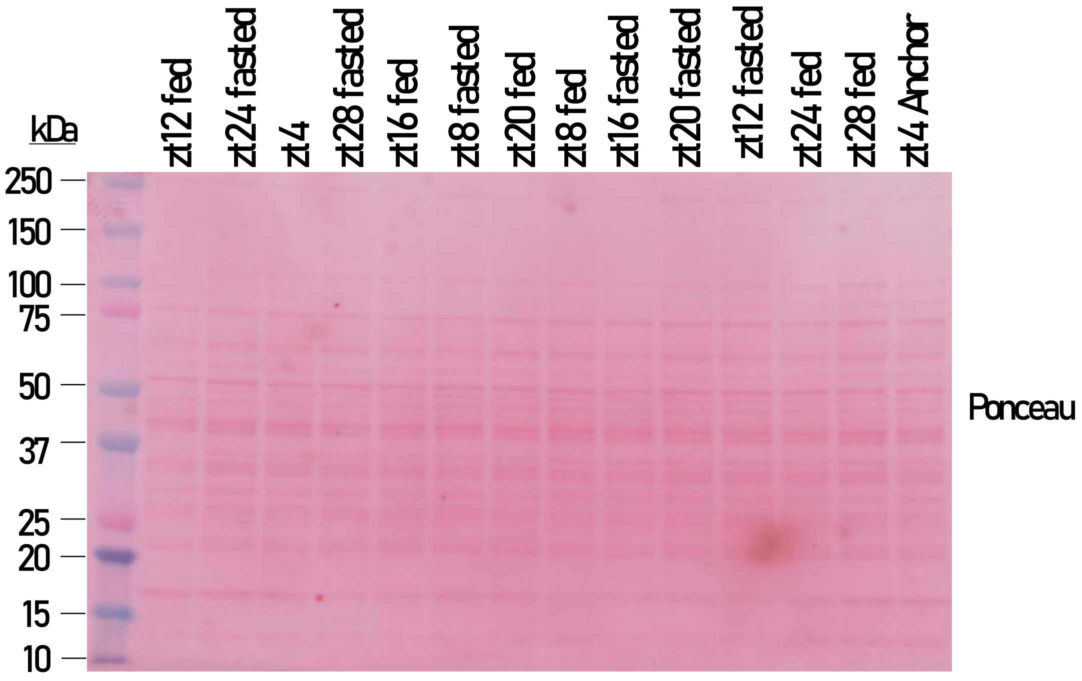

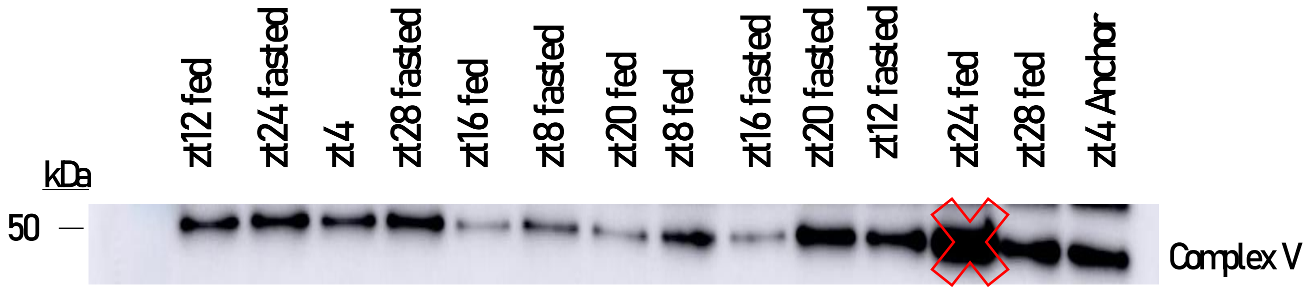

*Complex 5 Zt24 fed saturation of signal

Gel 6

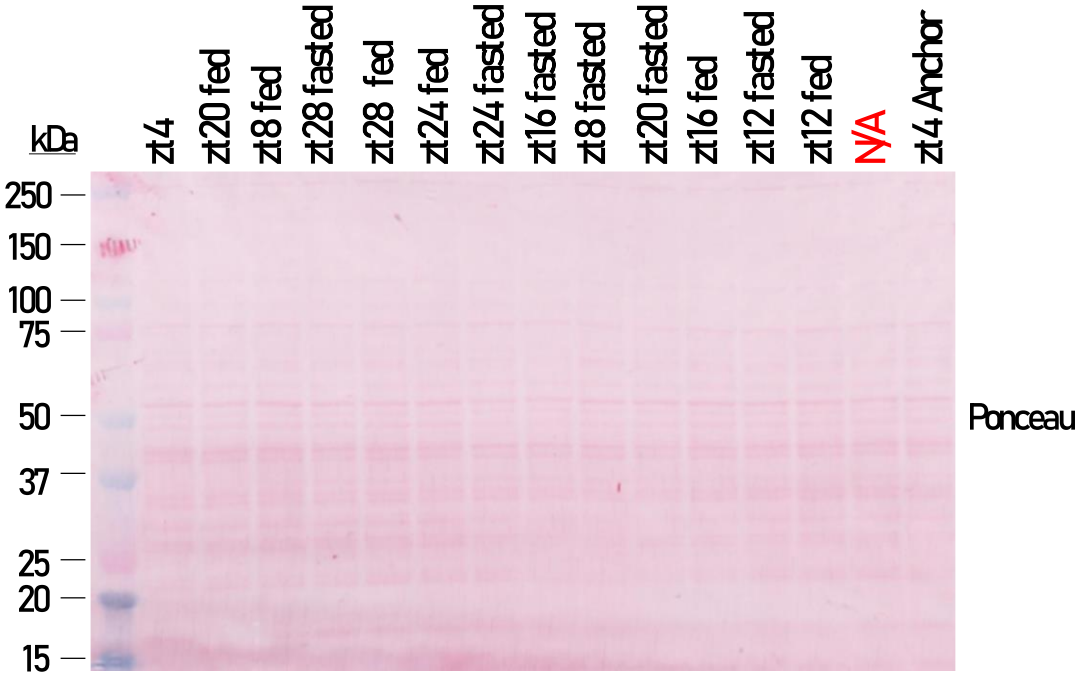

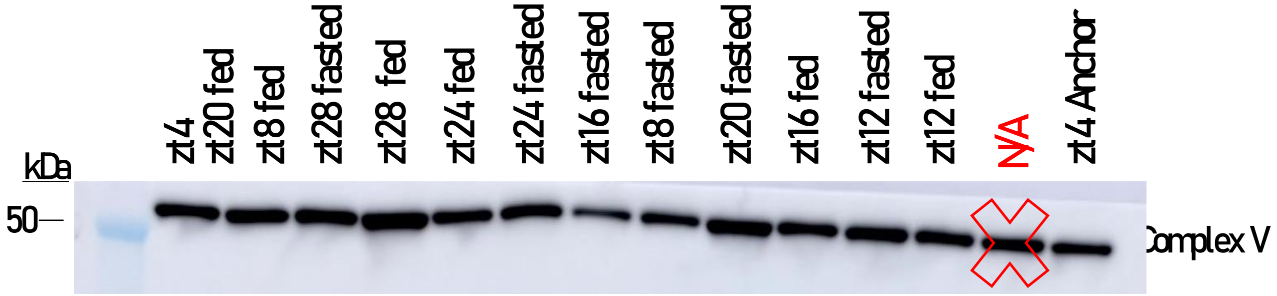

*N/A, wrong sample loaded

Gel 6b

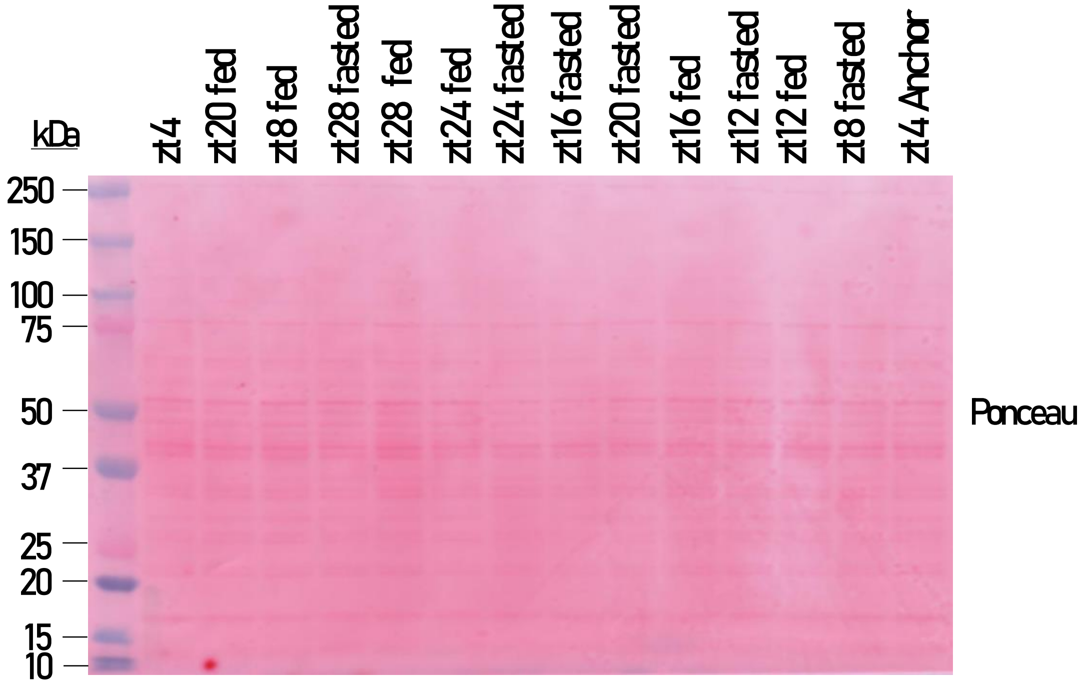
kDa
250
150
100
75
50
37
25
20
15
10
zt4
zt20 fed
zt8 fed
zt28 fasted
zt28 fed
zt24 fed
zt24 fasted
zt16 fasted
zt20 fasted
zt16 fed
zt12 fasted
zt12 fed
zt8 fasted
zt4 Anchor
Ponceau

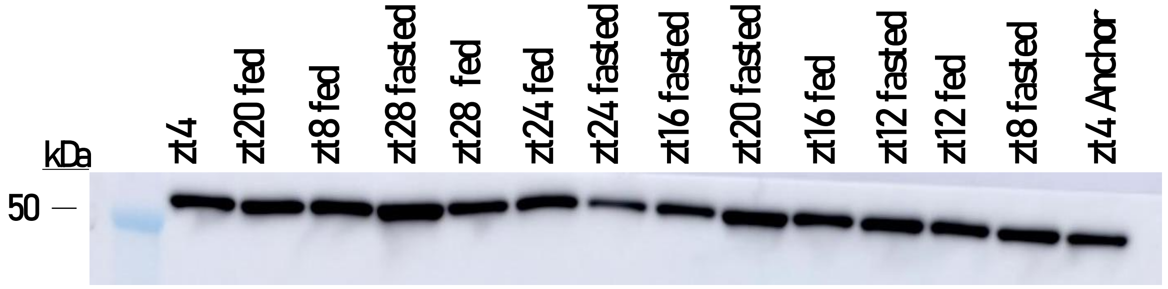
kDa
50
zt4
zt20 fed
zt8 fed
zt28 fasted
zt28 fed
zt24 fed
zt24 fasted
zt16 fasted
zt20 fasted
zt16 fed
zt12 fasted
zt12 fed
zt8 fasted
zt4 Anchor

Gel 7

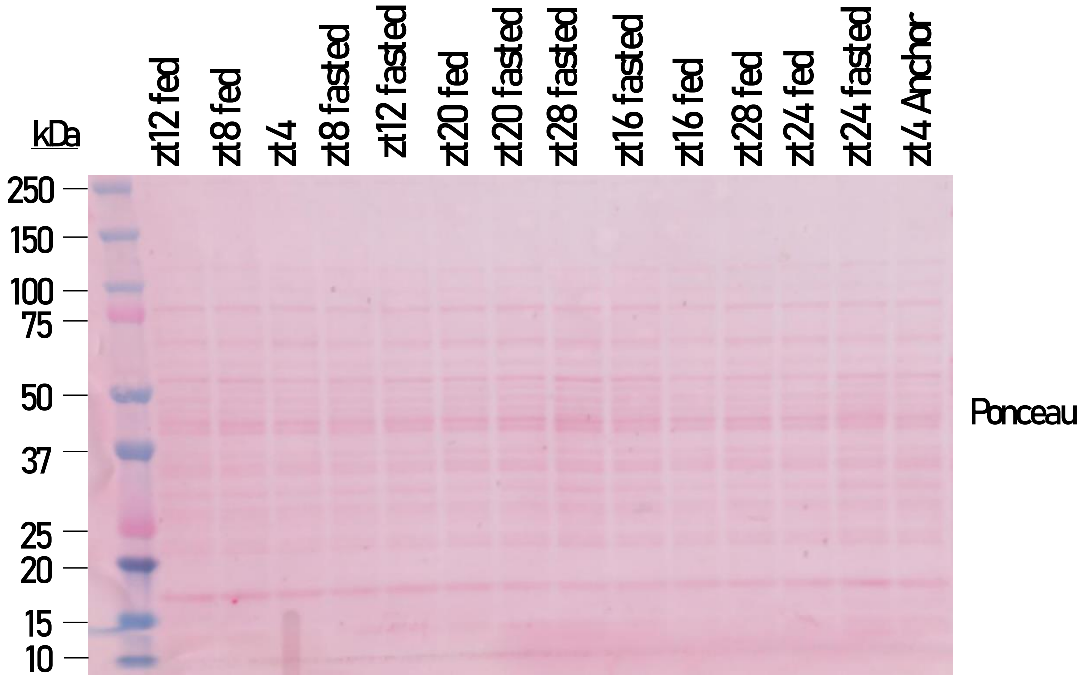
kDa
250
150
100
75
50
37
25
20
15
10
zt12 fed
zt8 fed
zt4
zt8 fasted
zt12 fasted
zt20 fed
zt20 fasted
zt28 fasted
zt16 fasted
zt16 fed
zt28 fed
zt24 fed
zt24 fasted
zt4 Anchor
Ponceau

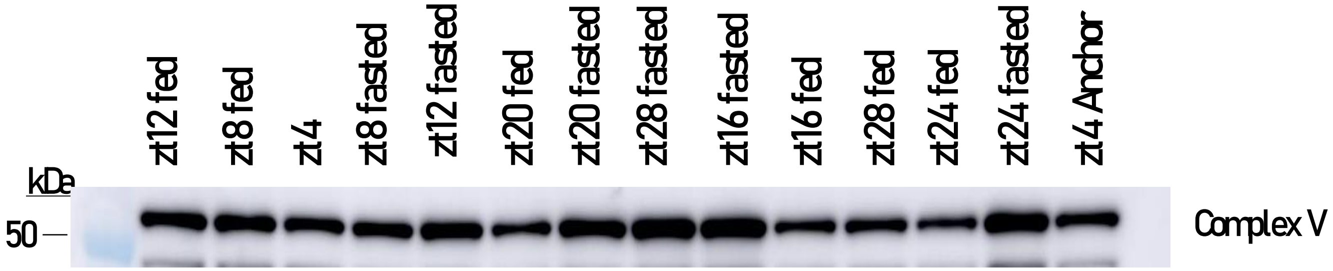
kDa
50
zt12 fed
zt8 fed
zt4
zt8 fasted
zt12 fasted
zt20 fed
zt20 fasted
zt28 fasted
zt16 fasted
zt16 fed
zt28 fed
zt24 fed
zt24 fasted
zt4 Anchor
Complex V

Gel 8

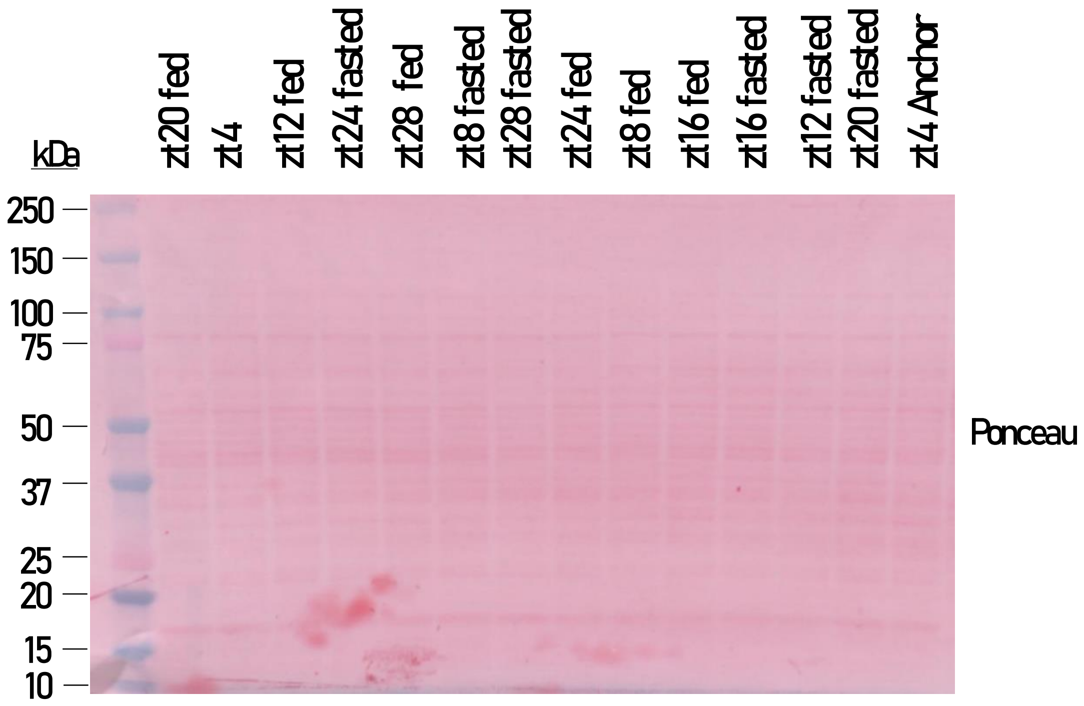
kDa
250
150
100
75
50
37
25
20
15
10
zt20 fed
zt4
zt12 fed
zt24 fasted
zt28 fed
zt8 fasted
zt28 fasted
zt24 fed
zt8 fed
zt16 fed
zt16 fasted
zt12 fasted
zt20 fasted
zt4 Anchor
Ponceau

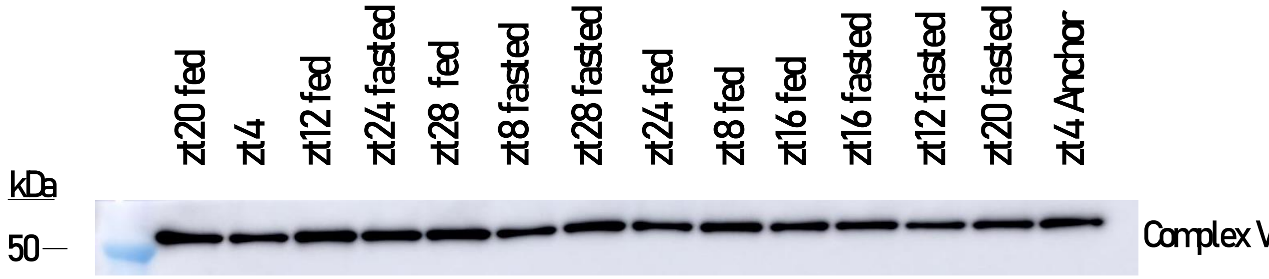
kDa
50
zt20 fed
zt4
zt12 fed
zt24 fasted
zt28 fed
zt8 fasted
zt28 fasted
zt24 fed
zt8 fed
zt16 fed
zt16 fasted
zt12 fasted
zt20 fasted
zt4 Anchor
Complex V

Gel 9

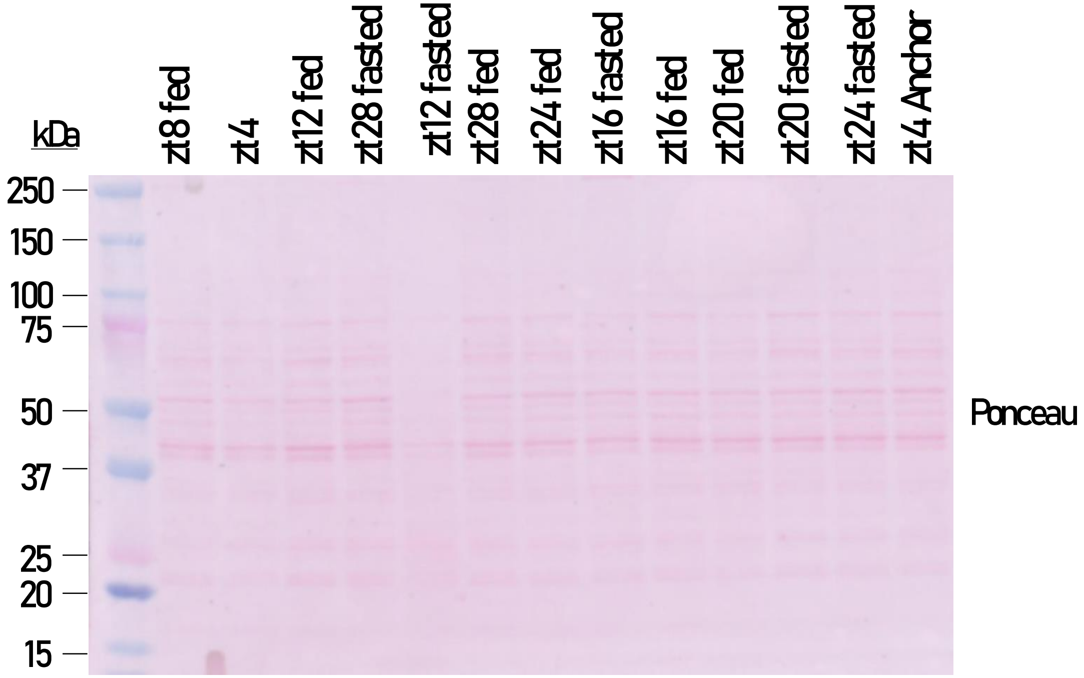

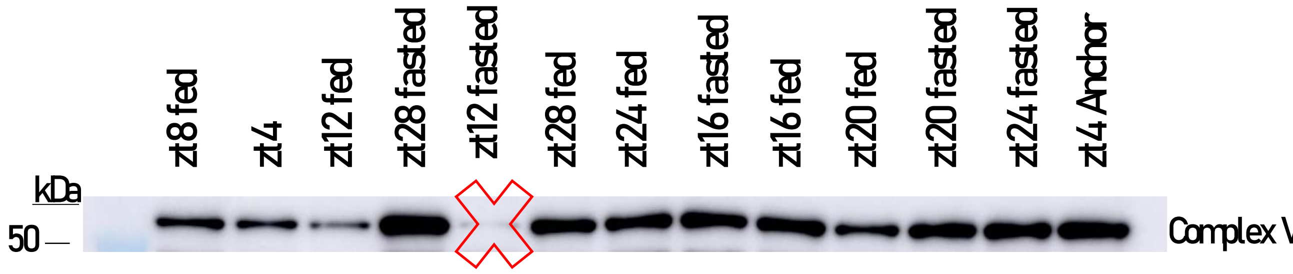

*Complex 5 Zt12fast signal close to background

Gel 10

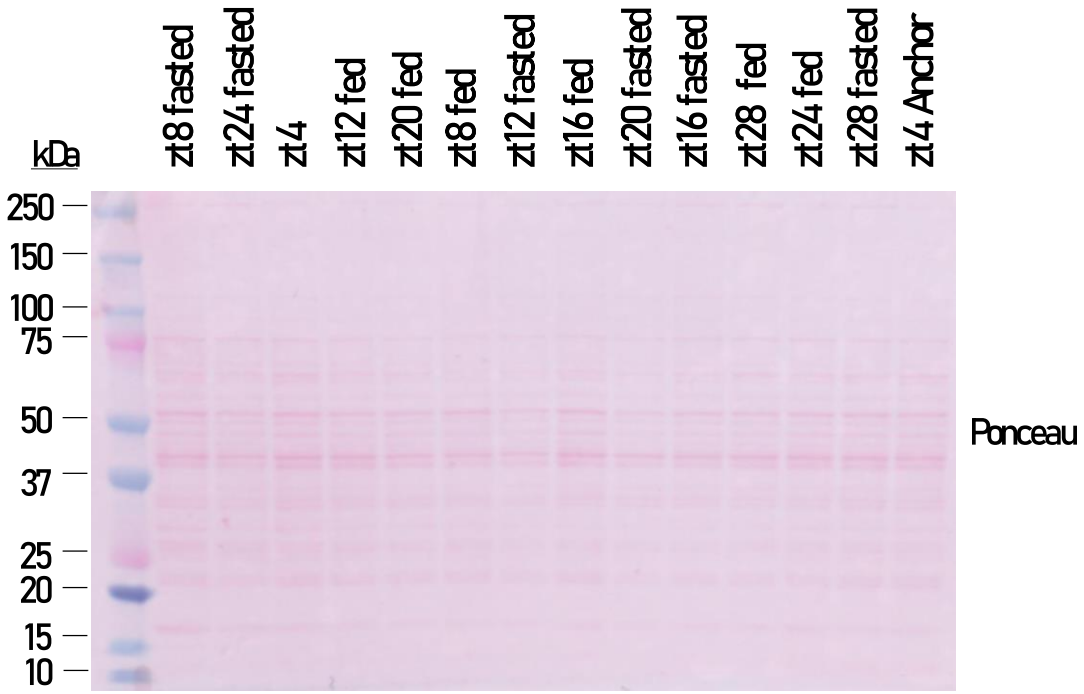
kDa
zt8 fasted
zt24 fasted
zt4
zt12 fed
zt20 fed
zt8 fed
zt12 fasted
zt16 fed
zt20 fasted
zt16 fasted
zt28 fed
zt24 fed
zt28 fasted
zt4 Anchor
250
150
100
75
50
37
25
20
15
10
Ponceau

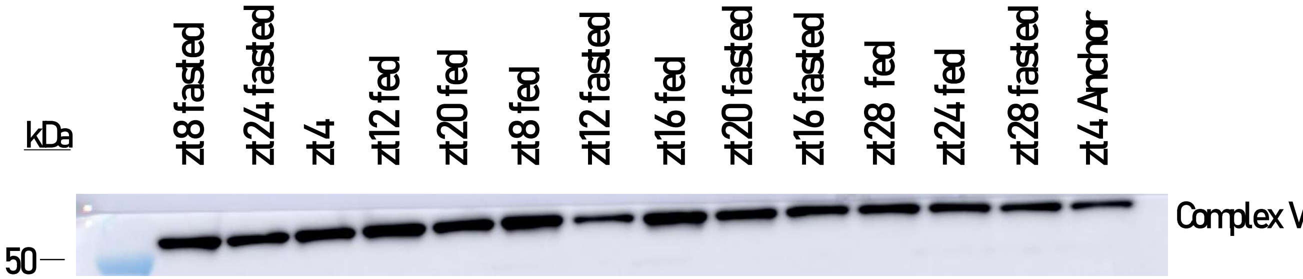
kDa
zt8 fasted
zt24 fasted
zt4
zt12 fed
zt20 fed
zt8 fed
zt12 fasted
zt16 fed
zt20 fasted
zt16 fasted
zt28 fed
zt24 fed
zt28 fasted
zt4 Anchor
50
Complex V

Gel 11

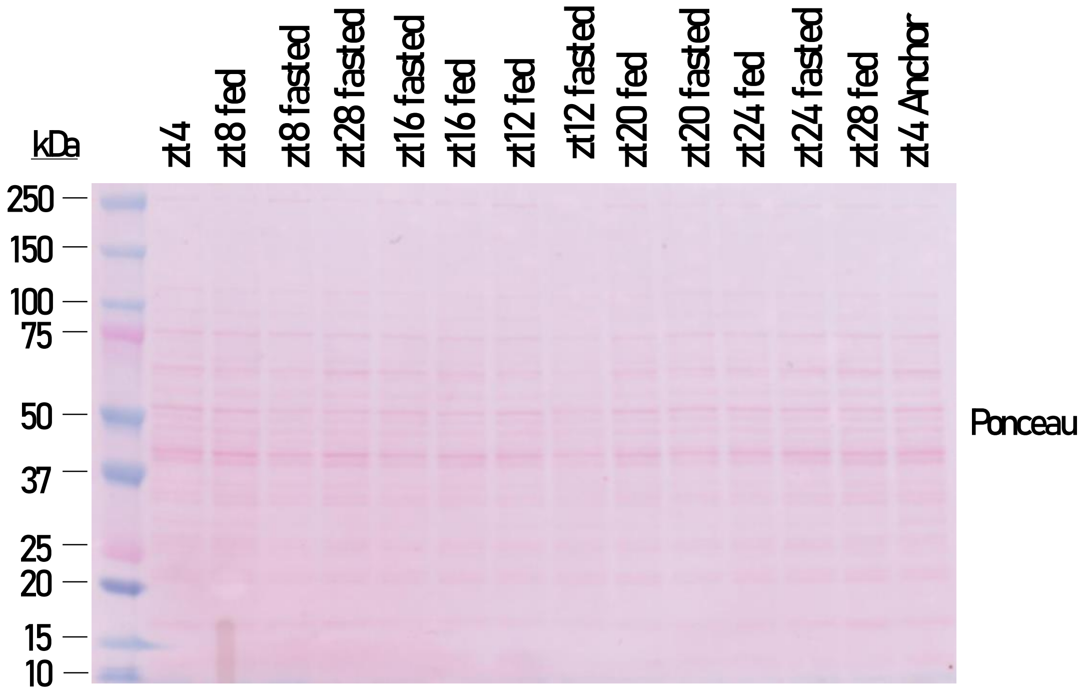

kDa
250
150
100
75
50
37
25
20
15
10
zt4
zt8 fed
zt8 fasted
zt28 fasted
zt16 fasted
zt16 fed
zt12 fed
zt12 fasted
zt20 fed
zt20 fasted
zt24 fed
zt24 fasted
zt28 fed
zt4 Anchor
Ponceau

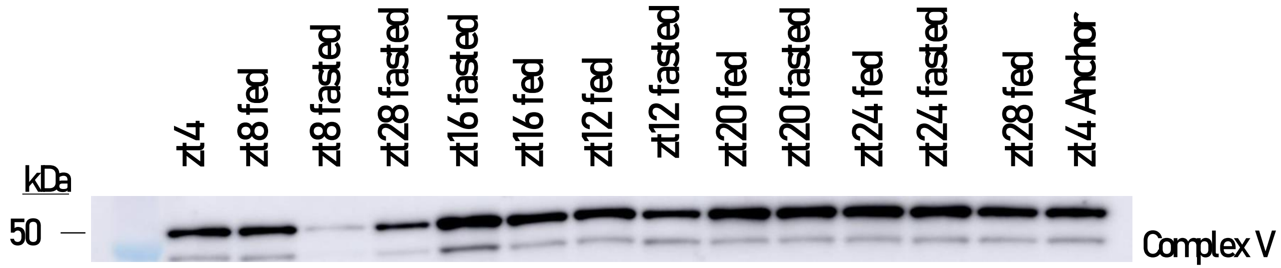

kDa
50
zt4
zt8 fed
zt8 fasted
zt28 fasted
zt16 fasted
zt16 fed
zt12 fed
zt12 fasted
zt20 fed
zt20 fasted
zt24 fed
zt24 fasted
zt28 fed
zt4 Anchor
Complex V

Gel 12

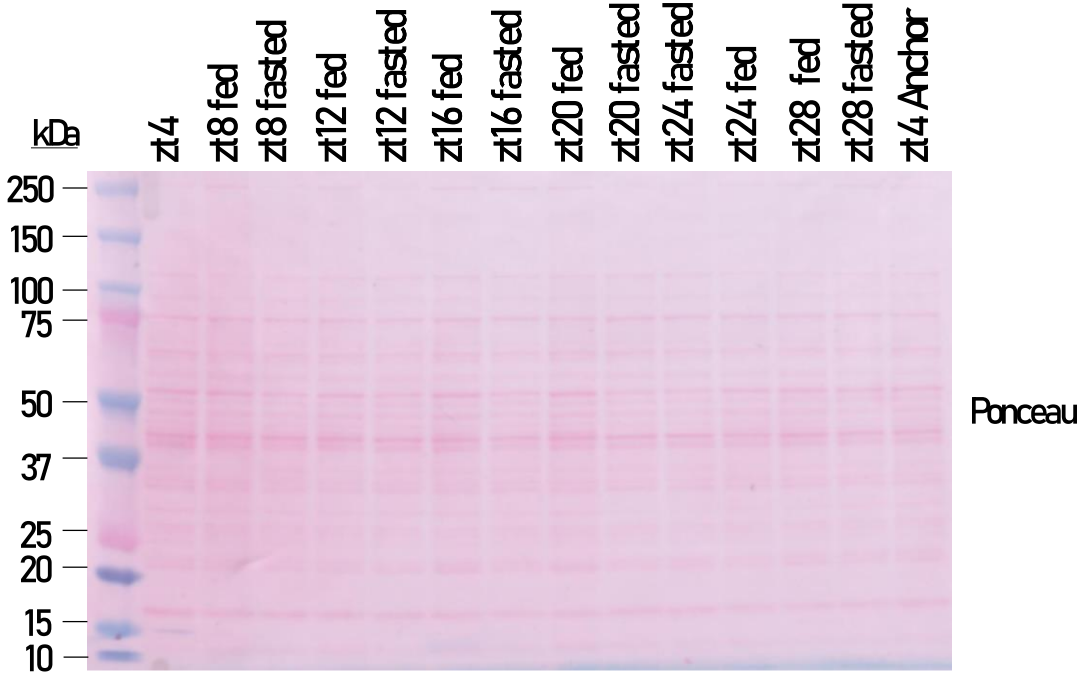
kDa
250
150
100
75
50
37
25
20
15
10
zt4
zt8 fed
zt8 fasted
zt12 fed
zt12 fasted
zt16 fed
zt16 fasted
zt20 fed
zt20 fasted
zt24 fasted
zt24 fed
zt28 fed
zt28 fasted
zt4 Anchor
Ponceau

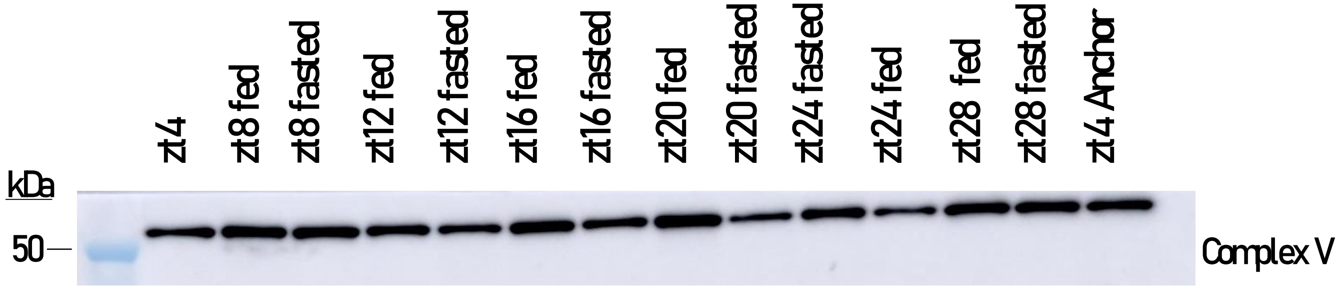
kDa
50
zt4
zt8 fed
zt8 fasted
zt12 fed
zt12 fasted
zt16 fed
zt16 fasted
zt20 fed
zt20 fasted
zt24 fasted
zt24 fed
zt28 fed
zt28 fasted
zt4 Anchor
Complex V
