# Supplementary material for: The interplay between sex, time of day, fasting status, and their impact on cardiac mitochondrial structure, function, and dynamics
Source: Sci Rep. 2023 Dec 7;13:21638. doi: 10.1038/s41598-023-49018-z (PMC10703790; doi:10.1038/s41598-023-49018-z)
Supplement: Supplementary file 3 — Supplementary Figure S1. [file 41598_2023_49018_MOESM3_ESM.pptx]

## Slide 1
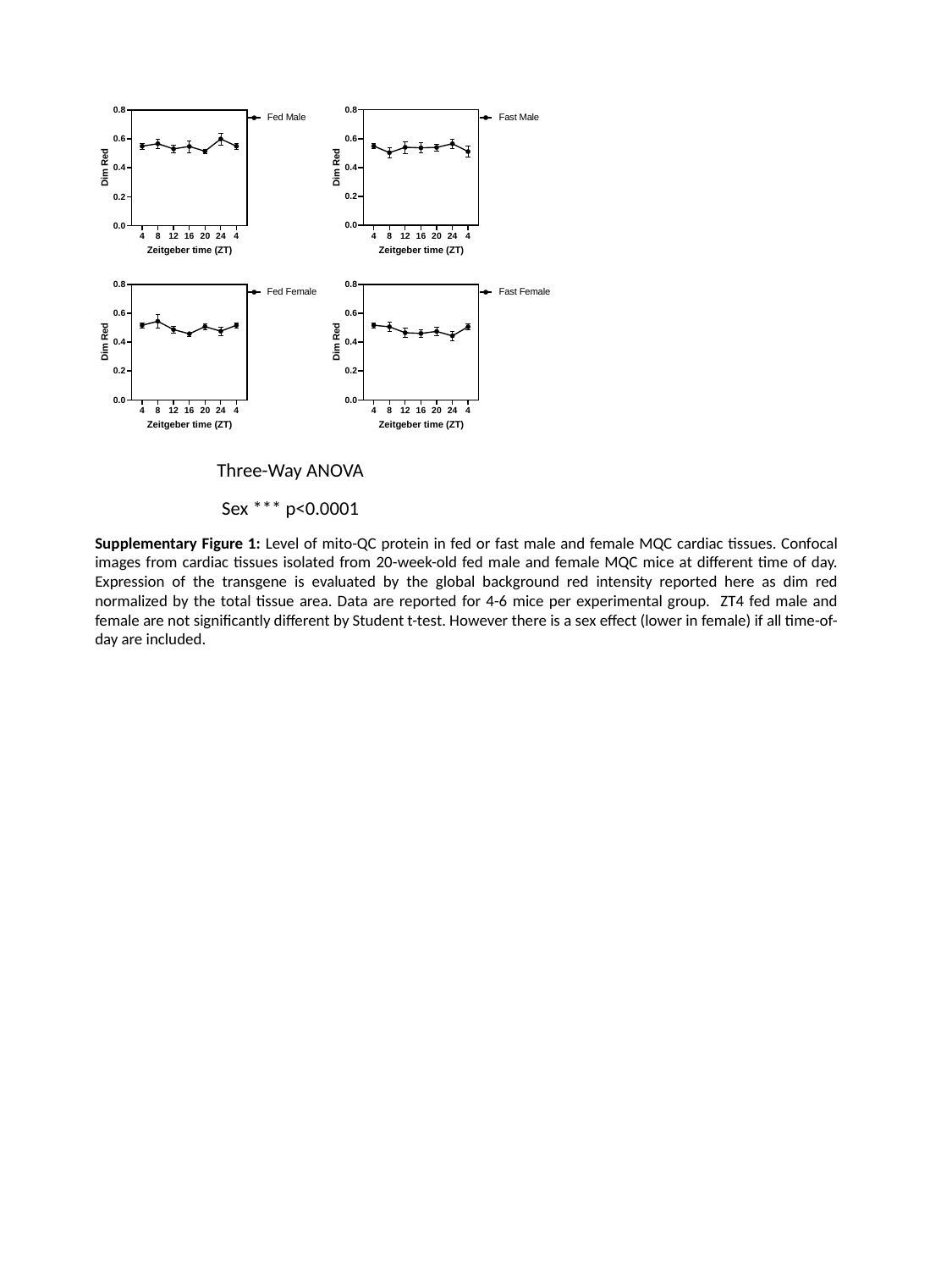

Three-Way ANOVA
Sex *** p<0.0001
Supplementary Figure 1: Level of mito-QC protein in fed or fast male and female MQC cardiac tissues. Confocal images from cardiac tissues isolated from 20-week-old fed male and female MQC mice at different time of day. Expression of the transgene is evaluated by the global background red intensity reported here as dim red normalized by the total tissue area. Data are reported for 4-6 mice per experimental group. ZT4 fed male and female are not significantly different by Student t-test. However there is a sex effect (lower in female) if all time-of-day are included.
